# Quality of life of the Canadian population using the VR-12: population norms for health utility values, summary component scores and domain scores

**DOI:** 10.1007/s11136-023-03536-5

**Published:** 2023-11-08

**Authors:** Logan Trenaman, Daphne Guh, Nick Bansback, Richard Sawatzky, Huiying Sun, Lena Cuthbertson, David G. T. Whitehurst

**Affiliations:** 1 Department of Health Systems and Population Health, School of Public Health, University of Washington, 3980 15th Ave NE, Fourth Floor, Box 351621, Seattle, WA 98195 USA; 2 Centre for Advancing Health Outcomes, Vancouver, BC Canada; 3 School of Population and Public Health, University of British Columbia, Vancouver, BC Canada; 4 School of Nursing, Trinity Western University, Vancouver, BC Canada; 5 British Columbia Office of Patient-Centred Measurement, Ministry of Health/Providence Health Care, Vancouver, BC Canada; 6 Faculty of Health Sciences, Simon Fraser University, Burnaby, BC Canada

**Keywords:** Health-related quality of life, Health utility, Normative value, Population norms, VR-12

## Abstract

**Objectives:**

To estimate Canadian population norms (health utility values, summary component scores and domain scores) for the VR-12.

**Methods:**

English and French speaking Canadians aged 18 and older completed an online survey that included sociodemographic questions and standardized health status instruments, including the VR-12. Responses to the VR-12 were summarized as: (i) a health utility value; (ii) mental and physical component summary scores (MCS and PCS, respectively), and (iii) eight domain scores. Norms were calculated for the full sample and by gender, age group, and province/territory (univariate), and for several multivariate stratifications (e.g., age group and gender). Results were summarized using descriptive statistics, including number of respondents, mean and standard deviation (SD), median and percentiles (25th and 75th), and minimum and maximum.

**Results:**

A total of 6761 people who clicked on the survey link completed the survey (83.4% completion rate), of whom 6741 (99.7%) were included in the analysis. The mean health utility score was 0.698 (SD = 0.216). Mean health utility scores tended to be higher in older age groups, ranging from 0.661 (SD = 0.214) in those aged 18–29 to 0.728 (SD = 0.310) in those aged 80+. Average MCS scores were higher in older age groups, while PCS scores were lower. Females consistently reported lower mean health utility values, summary component scores and domain scores compared with males.

**Conclusions:**

This is the first study to present Canadian norms for the VR-12. Health utility norms can serve as a valuable input for Canadian economic models, while summary component and domain norms can help interpret routinely-collected data.

**Supplementary Information:**

The online version contains supplementary material available at 10.1007/s11136-023-03536-5.

## Introduction

Generic health-related quality of life instruments—measures of self-perceived health status designed to be applicable across diverse groups of respondents, regardless of disease or demographics—have a multitude of applications in research and clinical care. These include as indicators of disease burden and health outcome, as case-mix indicators for risk adjustment, and at the individual patient-level for informing care [1–5]. There is no gold standard of measurement for health-related quality of life, whether generic or disease-specific. For this reason, it is important that instrument-specific resources exist to assist with the interpretation of data. An example of such a resource is the establishment of reference data for population norms, which summarize health outcomes in the general population and across demographic and clinical subgroups. The availability of population norms enables researchers to compare estimates from study samples with a population reference. Such data are useful in multiple contexts, including tracking changes in health outcomes over time [6–9], comparing populations with different health conditions [10], comparing between countries [11, 12], and modelling comparison groups in the evaluation of health technologies in the absence of primary data [13].

The Veterans RAND 12-item Health Survey (VR-12) and associated measures are among the most widely used instruments for assessing respondents’ (patients and non-patients) perceptions of health domains relevant to their quality of life [5, 14]. The VR-12 was developed from the Veterans RAND 36-item Health Survey, which was developed and modified from the original RAND version of the 36-item Health Survey version 1.0 (also known as the MOS SF-36) [15, 16]. Responses to the VR-12 can be used to generate (i) health utility values, (ii) summary component scores for mental health and physical health, and (iii) scores for each of the instrument’s eight domains. (Further details about the VR-12 are provided in the *Methods* section.) Population norms for the VR-12 have been described using data from over 170,000 respondents to the Medicare Health Outcomes Survey in the United States (US) [16] and a sample of 500 Chinese adults in Hong Kong [17]. There are no Canadian population norms for the VR-12. Previous studies have published Canadian population norms for health utility scores generated using other instruments, including the Health Utilities Index Mark 3 (HUI3) [13], EQ-5D-3L (Alberta only) [18], EQ-5D-5L [19, 20], and, for Quebec only, the SF-6Dv2 (albeit with preference weights derived from a representative sample of the United Kingdom (UK) population) [21].

Health utility values are an important input in the economic evaluation of health technologies [13, 22, 23], where they can be used to estimate quality-adjusted life years (QALYs) [24, 25]. A health utility value (also known as a health state value or a preference weight) is an index score anchored on a 0–1 scale, where zero is interpreted as a state equivalent to dead and one is considered ‘full health’. The value provides a representation of a utility, reflecting the description of a health state provided by the respondent (e.g., an individual completing the VR-12) and the value of this health state based on the preferences of a representative sample of the general population [24, 26]. Negative health utility values are possible, reflecting health states worse than dead. In Canada, guidelines for the economic evaluation of health technologies state that health preferences should reflect the general Canadian population [27]. Researchers/analysts are also encouraged to examine potential sources of heterogeneity that may lead to using different parameter-input values (such as health utility values) across subgroups. The provision of norms that reflect societal preferences, by univariate (e.g., age or gender) and multivariate (e.g., age and gender) stratifications, can facilitate this type of analysis [13, 27].

The objective of this study was to estimate Canadian population norms (health utility values, summary component scores and domain scores) for the VR-12, including norms by univariate and multivariate stratifications.

## Methods

The study was approved by the University of British Columbia Behavioural Research Ethics Board (study number H18-00969). The Checklist for Reporting Results of Internet E-Surveys (CHERRIES) was followed in the preparation of this manuscript [28].

### Study design

Data for this study were obtained via an online survey that was designed to serve multiple purposes. Details on the survey development have been reported previously [29]. There were two versions of the survey (Survey A and Survey B). Survey A included: (i) sociodemographic and clinical questions (regarding age, gender, province or territory of residence, education, population group, marital status, household income, and self-reported health conditions), (ii) standardized instruments for measuring health status, including the VR-12 (the focus of the analyses reported herein) as well as other instruments (EQ-5D-5L [30] and PROMIS Global-10 [31], not reported herein) and (iii) a health state valuation exercise using a discrete choice experiment for estimating a Canadian preference-based scoring algorithm for the VR-12 (published elsewhere [29]). In Survey A, the VR-12 was administered after the sociodemographic and clinical questions, and before the health state valuation exercise. The EQ-5D-5L and PROMIS Global-10 were administered after the health state valuation exercise. Survey B included the same sociodemographic and clinical questions and a different set of standardized health status instruments (VR-12, EQ-5D-5L, VR-36 [32]). There was no health state valuation exercise in Survey B. The VR-12, EQ-5D-5L and VR-36 instruments were administered in a fixed order (as listed), after the sociodemographic and clinical questions. The surveys went through two stages of development. The first phase included development and piloting with the study team. The second phase involved the testing of Survey A with focus groups consisting of members of the Canadian general population. Once finalized, the surveys were translated into French. Official translations of the VR-12, EQ-5D-5L, and PROMIS Global-10 were used, with the remaining components of the surveys undergoing forward and backward translation.

### Study participants

A sample of English and French speaking Canadians aged 18 and older that was broadly representative of the Canadian general population was recruited through a market research company (Ipsos Canada). Quota sampling was used to ensure the sample was representative of the Canadian population according to gender, age group (18–29, 30–39, 40–49, 50–59, 60–69, and 70+ years), and geographic location. Eligible market research panelists received an email from the company with an invitation to participate in the study. Those who clicked on this invitation were provided with information about the survey, including the purpose, study team, how their data would be used and stored, and the expected time to complete the survey. Eligible participants were then asked to provide electronic informed consent. Those who consented were randomized to either Survey A or Survey B; participants were able to choose between English and French versions of their assigned survey. Data were collected between January and February 2019. We did not collect any identifiable information beyond a unique study ID, and all data were stored on a secure server. We checked Internet Protocol addresses for duplicates to reduce the likelihood that individuals completed the survey more than once.

### The Veterans RAND 12-item Health Survey (VR-12)

The VR-12 is a 14-item instrument. The first 12 items correspond to eight health domains: general health (GH, one item), physical functioning (PF, two items), role physical (RP, two items), bodily pain (BP, one item), role emotional (RE, two items), vitality (VT, one item), mental health (MH, two items), and social functioning (SF, one item). The remaining two VR-12 items ask about changes in physical and emotional health “*compared to one year ago*”. The response scales for VR-12 items vary, ranging from three-point scales to six-point scales. Details of the wording for all VR-12 items and response options is provided in the Supplementary Material (SM1).

Responses to the VR-12 can be scored in three ways: a health utility value; two summary component scores, the physical component summary (PCS) and mental component summary (MCS) scores; and scores for each of the eight domains. Details of the scoring procedures used in this study for all three approaches are described in the following section. The two items measuring change are for descriptive purposes only and are not used in any scoring procedures.

### Scoring procedures for the VR-12

Health utility values were estimated using a scoring algorithm developed by Bansback and colleagues (using data from the health state valuation exercise in Survey A) [29]. The classification system for deriving VR-12 health utilities for the Canadian population comprises eight attributes, derived from eight VR-12 items (see SM1). The composition of the eight attributes in the classification system for deriving health utilities is different to the eight domains of the VR-12. To avoid confusion, the following naming conventions are used in this paper for the VR-12 classification system attributes: physical functioning (PF_u_), role physical (RP_u_), role emotional (RE_u_), bodily pain (BP_u_), mental health-anxiety (MA_u_), mental health-depression (MD_u_), vitality (VT_u_), and social functioning (SF_u_), with the ‘u’ subscript signifying the connection with the health *utility* values.

The scoring algorithm estimates a disutility for each level within the eight attributes, relative to the ‘best’ level of response (i.e., a ‘level 1’ response, indicating no problems for the respective attribute, has a disutility of zero). For each respondent, a health utility value is calculated as 1.000 (‘full health’) minus the sum of the eight disutilities. Using this algorithm, health utilities can range from -0.589 (the lowest level of response on all eight attributes) to 1.000 (level 1 responses for all eight attributes).

Summary component scores were calculated using algorithms described by Selim and colleagues [16]. The procedure begins with a linear transformation of items from a raw score so that scores range from 0 (worst health status) to 100 (best health status). These transformed items are then multiplied by weights to obtain VR-12 scores that correspond with normed SF-12 scores based on a nationally representative sample of the US population from the Medical Expenditure Survey, which was administered between 2000 and 2002. MCS and PCS scores are expressed as a T-score with a mean of 50 and standard deviation of 10. This is known as norm-based scoring; scores above (below) 50 are interpreted as reporting better (worse) than the average health status of the general US population, and a 10-point difference in scores corresponds to one standard deviation. To obtain norm-based scores for the Canadian population, a further transformation was applied by standardizing the norms to a mean of 50 and standard deviation of 10 using the current sample. This was accomplished by calculating a Z-score based on the following formula:

$$ Z = \frac{x - \mu }{\sigma } $$

where $x$ is the raw score, $\mu$ is the population mean, and $\sigma$ is the population standard deviation. This Z-score is then transformed to a T-score as follows:

$$ {\text{T }} = \left( {Z \times 10} \right) + 50 $$

The eight domain scores were calculated using an extensibility scoring algorithm described by Selim and colleagues [33]. This algorithm includes coefficients for each response category for each item on the VR-12. Domain scores are calculated based on the coefficient for the item(s) corresponding to that domain.

### Statistical analysis

Descriptive statistics were used to summarize the sociodemographic characteristics of survey participants. VR-12 data were weighted to account for unit non-response and non-coverage of the internet sample, with the 2021 Census and 2017/18 Canadian Community Health Survey (CCHS) used as the population standard [34, 35]. An interactive proportional fitting method, also known as raking, was implemented using the SAS Raking Macro—Generation IV [36]. The aim was to match the distribution of five variables in our sample to the known population distribution: gender, geographic location, and education from the Census; and age and self-reported health status from the CCHS. The raking algorithm used the same categories available in the underlying data, and the following categories for age: 18–29, 30–39, 40–49, 50–59, 60–69, 70–79, and 80+ years. The algorithm also considered the cross-classification of age by self-reported health status. The same survey weights were used for all analyses.

Norms for health utility values, summary component scores (MCS and PCS) and domain scores were calculated for the full sample, by male and female gender, by age group (18–29, 30–39, 40–49, 50–59, 60–69, 70–79, and 80 + years), by province/territory (data permitting), and for 13 self-reported health conditions. The question about health conditions asked, “Do you have any of the following health conditions now? (Select all that apply).” The 13 listed health conditions were: anemia or blood disease, back pain, cancer, depression, diabetes, heart disease, high blood pressure, kidney disease, liver disease, lung disease, osteoarthritis /degenerative arthritis, rheumatoid arthritis, and ulcer or stomach disease. Norms are also reported for multivariate stratifications: by age group and gender; by health condition and gender; and by province/territory, age group and gender. All results are presented using descriptive statistics only (i.e., no inferential analyses were performed). Norms are summarized using the following statistics: number of respondents, mean and standard deviation, median and percentiles (25th and 75th), and minimum and maximum. Across all analyses, norms are not reported for cell counts less than five. Analyses were performed using SAS and R Statistical Software version 3.5.2 (Vienna, Austria).

## Results

Of 8110 individuals who clicked on the survey invitation link, 6761 (83.4%) completed the survey and 6741 (99.7% of the 6761) were included in the analysis (12 were excluded based on age and eight excluded because of missing VR-12 data). Sample characteristics for the sample (weighted and unweighted), and the 2021 Canadian census are presented in Table 1. Our sample was broadly representative of the Canadian population on most characteristics but was more highly educated and more likely to report a higher household income.

**Table 1:** Sample characteristics for the full sample (n = 6741), weighted sample, and comparable statistics for the Canadian general population, drawn from Statistics Canada 2021 Census and 2017/18 Canadian Community Health Survey*

	Full sample, n(%)	Weighted sample, %	General population, %
Age group (years)			
18–29	1285 (19.1)	19.1	19.1
30–39	1174 (17.4)	17.8	17.8
40–49	1272 (18.9)	16.2	16.2
50–59	1308 (19.4)	17.6	17.6
60–69	749 (11.1)	15.7	15.7
70–79	859 (12.7)	9.2	9.2
80 and over	94 (1.4)	4.3	4.3
Gender			
Male	3358 (49.8)	48.8	48.8
Female	3370 (50.0)	50.8	50.8
Other	13 (0.2)	0.3	0.3
Province/Territory			
Alberta	721 (10.7)	11.5	11.5
British Columbia	1008 (15.0)	13.5	13.5
Manitoba	280 (4.2)	3.8	3.6
New Brunswick	153 (2.3)	1.7	2.1
Newfoundland and Labrador	90 (1.3)	1.5	1.4
Northwest Territories	10 (0.1)	0.1	0.1
Nova Scotia	239 (3.5)	3.0	2.6
Nunavut	2 (0.0)	0.0	0.1
Ontario	2605 (38.6)	38.5	38.5
Prince Edward Island	41 (0.6)	0.5	0.4
Quebec	1385 (20.5)	23.0	23.0
Saskatchewan	203 (3.0)	2.9	3.1
Yukon	4 (0.1)	0.1	0.1
Education			
No certificate, diploma or degree	235 (3.5)	18.3	18.3
Secondary (high) school diploma or equivalency certificate	1583 (23.5)	26.5	26.5
Apprenticeship or trades certificate or diploma	461 (6.8)	9.8	9.8
College, CEGEP or other non-university certificate or diploma	1645 (24.4)	19.4	19.4
University certificate or diploma below bachelor level	617 (9.2)	2.8	2.8
University certificate, diploma, or degree at bachelor level or above	2200 (32.6)	23.3	23.3
Population group			
White	5410 (80.3)	82.3	70.1
South Asian	235 (3.5)	2.9	6.9
Chinese	465 (6.9)	4.7	4.8
Black	142 (2.1)	2.3	3.8
Filipino	66 (1.0)	0.9	2.6
Latin American	79 (1.2)	1.3	1.6
Arab	51 (0.8)	0.6	1.7
Southeast Asian	65 (1.0)	1.0	1.1
West Asian	32 (0.5)	0.4	1.0
Korean	25 (0.4)	0.2	0.6
Japanese	30 (0.4)	0.4	0.3
First Nation (North American Indian)	97 (1.4)	1.6	2.5
Métis	36 (0.5)	0.5	1.6
Inuit	5 (0.1)	0.1	0.2
Other Indigenous/Aboriginal	36 (0.5)	0.9	0.1
Other	177 (2.6)	2.5	0.5
Marital status			
Never legally married	2185 (32.4)	32.20	29.14
Legally married (and not separated)	2915 (43.2)	41.57	44.31
Separated, but still legally married	190 (2.8)	3.29	2.39
Divorced	483 (7.2)	7.31	6.20
Widowed	234 (3.5)	4.48	5.37
Living with a common-law partner	733 (10.9)	11.02	12.59
Annual household gross income			
$0–$19,999	712 (10.6)	12.9	18.2
$20,000–$49,999	1864 (27.7)	31.0	36.0
$50,000–$99,999	2477 (36.7)	34.2	26.4
$100,000–$149,999	1128 (16.7)	14.6	16.2
$150,000 and over	518 (7.7)	6.5	3.3
Self-reported health status			
Excellent/Very good	2816 (41.8)	59.8	59.8
Good	2615 (38.8)	28.5	28.5
Fair/Poor	1310 (19.4)	11.7	11.7
Survey language			
English	6199 (92.0)	–	–
French	542 (8.0)	–	–

*Numbers do not always sum to the respective totals because of missing data. Missing data were observed for marital status (n = 1) and annual household gross income (n = 42)

### Canadian norms—health utility values

**Fig. 1:**
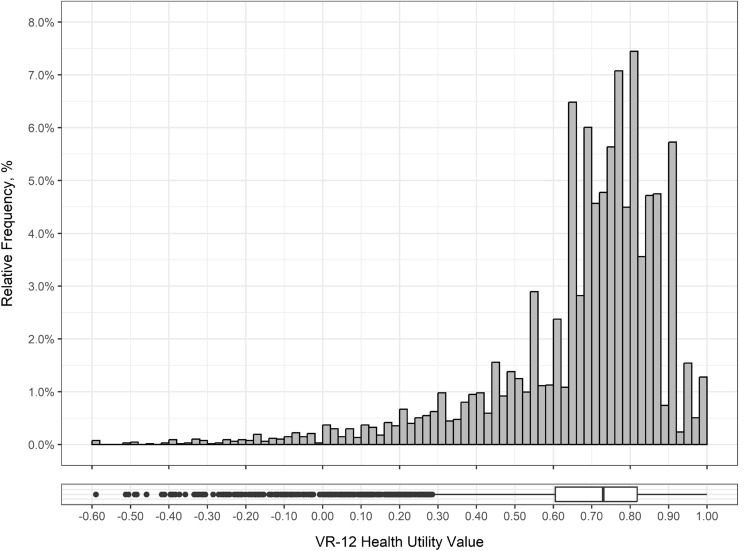
Histogram and boxplot of VR-12 health utility values for the full sample (n = 6741)

A histogram and boxplot of the health utility values for the sample is presented in Fig. 1. The distribution is negatively skewed, with clustering at the upper end of the 0–1 scale. Overall, the mean health utility score in the sample was 0.698 (SD = 0.216). Table 2 presents health utility norms (mean and standard deviation only) for Canadians by age group and gender. Older age groups had higher mean health utility scores, with, for example, means of 0.661 (SD = 0.214) in those aged 18–29 and 0.728 (SD = 0.310) in those aged 80 + . Except for the 80 + age group, mean health utility scores were higher for males compared with females. Detailed statistics summarizing Canadian population health utility norms by age group (univariate) and by gender and age group (multivariate) are reported in SM2. Further stratifications are provided in SM3 by province (univariate), by province and age group (multivariate), and by province, age group and gender (multivariate). Across provinces and territories, mean health utility scores were between 0.682 and 0.703, with the exceptions of Manitoba (0.637), Northwest Territories (0.654), Quebec (0.734) and Newfoundland and Labrador (0.756); no norms are reported for Nunavut or Yukon because of small sample sizes. Northwest Territories and Prince Edward Island were the only regions with higher mean health utility score for females compared with males, although the small sample sizes should be noted (n = 10 and n = 41, respectively).

**Table 2:** Canadian norms for VR-12 health utility values and summary component scores, by gender and age group. Numbers are means (standard deviations)*

	Full sample	Male	Female
VR-12 health utility values			
Full sample	0.698 (0.216)	0.721 (0.190)	0.678 (0.235)
Age group (years)			
18–29	0.661 (0.214)	0.694 (0.189)	0.636 (0.223)
30–39	0.686 (0.221)	0.712 (0.194)	0.661 (0.240)
40–49	0.710 (0.194)	0.724 (0.185)	0.697 (0.202)
50–59	0.697 (0.228)	0.731 (0.199)	0.664 (0.251)
60–69	0.724 (0.231)	0.730 (0.206)	0.720 (0.246)
70–79	0.721 (0.186)	0.752 (0.145)	0.680 (0.230)
80 and over	0.728 (0.310)	0.726 (0.297)	0.732 (0.338)
VR-12 physical component summary scores			
Full sample	50.000 (10.000)	50.275 (9.591)	49.737 (10.370)
Age group (years)			
18–29	53.686 (7.598)	53.846 (7.473)	53.565 (7.658)
30–39	52.155 (8.372)	52.951 (7.568)	51.407 (8.940)
40–49	51.985 (7.975)	52.128 (7.704)	51.852 (8.246)
50–59	49.096 (10.041)	49.972 (9.154)	48.218 (10.855)
60–69	47.053 (12.697)	46.394 (12.341)	47.460 (12.910)
70–79	45.128 (9.608)	46.267 (8.863)	43.628 (10.507)
80 and over	42.165 (19.407)	41.369 (18.912)	43.783 (20.348)
VR-12 mental component summary scores			
Full sample	50.000 (10.000)	51.075 (9.284)	49.074 (10.457)
Age group (years)			
18–29	44.874 (10.609)	46.500 (10.283)	43.655 (10.386)
30–39	48.099 (9.842)	48.933 (9.368)	47.314 (10.217)
40–49	49.511 (9.174)	50.421 (8.805)	48.664 (9.472)
50–59	51.090 (9.181)	52.307 (8.149)	49.871 (10.044)
60–69	53.298 (9.985)	54.229 (8.724)	52.743 (10.678)
70–79	54.928 (6.581)	55.927 (5.934)	53.613 (7.303)
80 and over	55.402 (11.337)	55.460 (11.902)	55.284 (10.333)

*Sample sizes for the ‘full sample’ statistics are reported in Table 1. Within each age group, the sample sizes for males and females, respectively, are: 586 and 690, 18–29; 578 and 593, 30–39; 642 and 630, 40–49; 678 and 630, 50–59; 294 and 454, 60–69; 518 and 341, 70–79; and 62 and 32, 80 and over

**Fig. 2:**
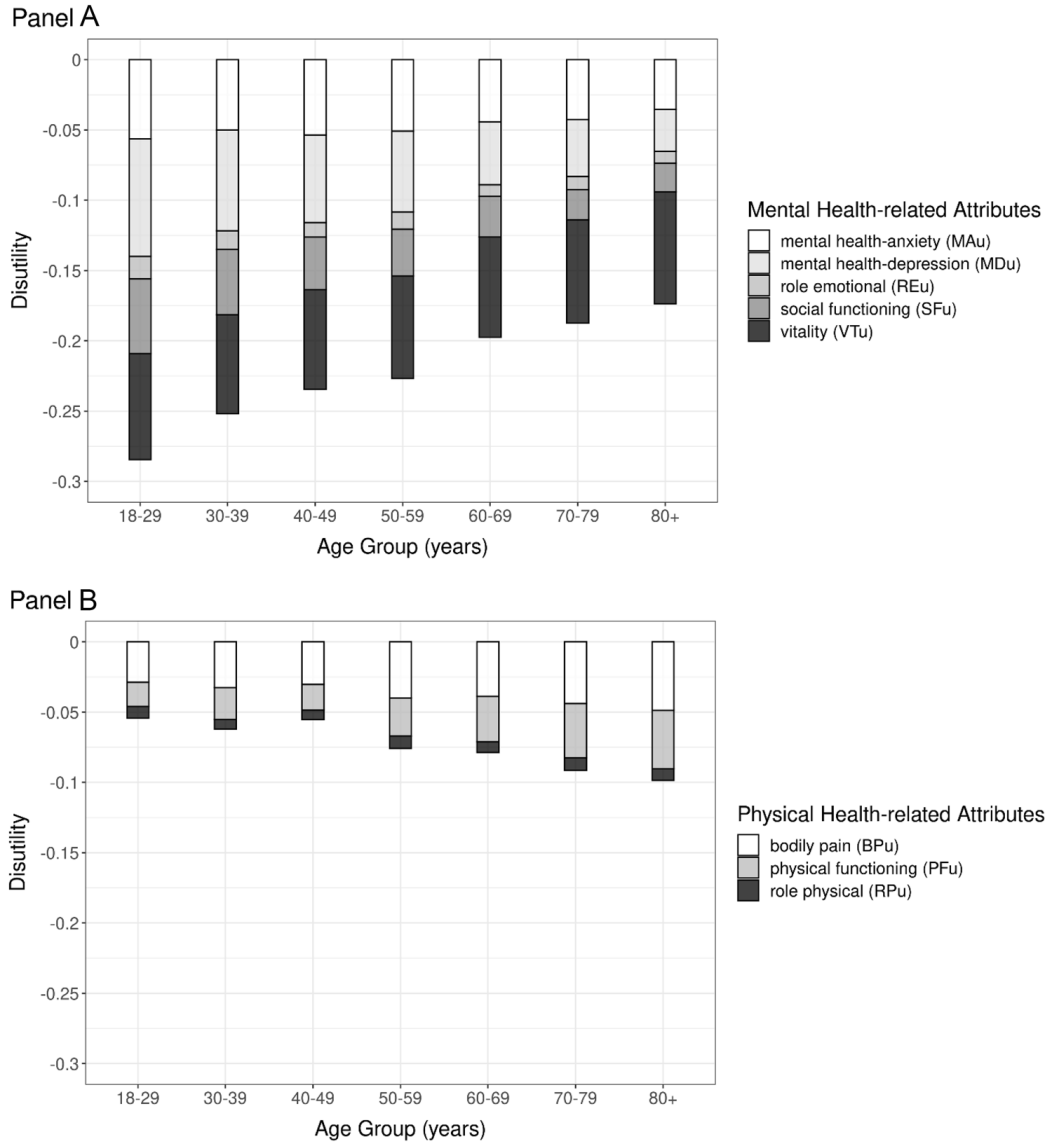
Mean disutilities, by attribute and age group, presented separately for mental health-related (panel A) and physical health-related (panel B) attributes

Mean disutilities, by classification system attribute and age group, are presented in Fig. 2 for mental health-related attributes and physical health-related attributes. With respect to mental health (Fig. 2, panel A), four of the five attributes (the exception being vitality (VT_u_)) show lower mean disutilities in older age groups. This means that, based on the preferences of the Canadian general population, a higher value (i.e., a smaller disutility) is placed on the descriptions of mental health status provided by older individuals. With respect to physical health (Fig. 2, panel B), two of the three physical health-related attributes show higher mean disutilities in older age groups (the exception being role physical (RP_u_))—meaning that, on average, Canadians place a lower value (i.e., higher disutility) on the descriptions of physical health status provided by older individuals.

Detailed health utility norms for 13 self-reported health conditions (univariate), and by self-reported health conditions and gender (multivariate), are reported in SM4. Across the 13 conditions, in the full sample, mean health utility values ranged from 0.682 (SD = 0.212) for those who reported high blood pressure to 0.493 (SD = 0.252) for those with depression.

### Canadian norms—summary component scores and domain scores

**Fig. 3:**
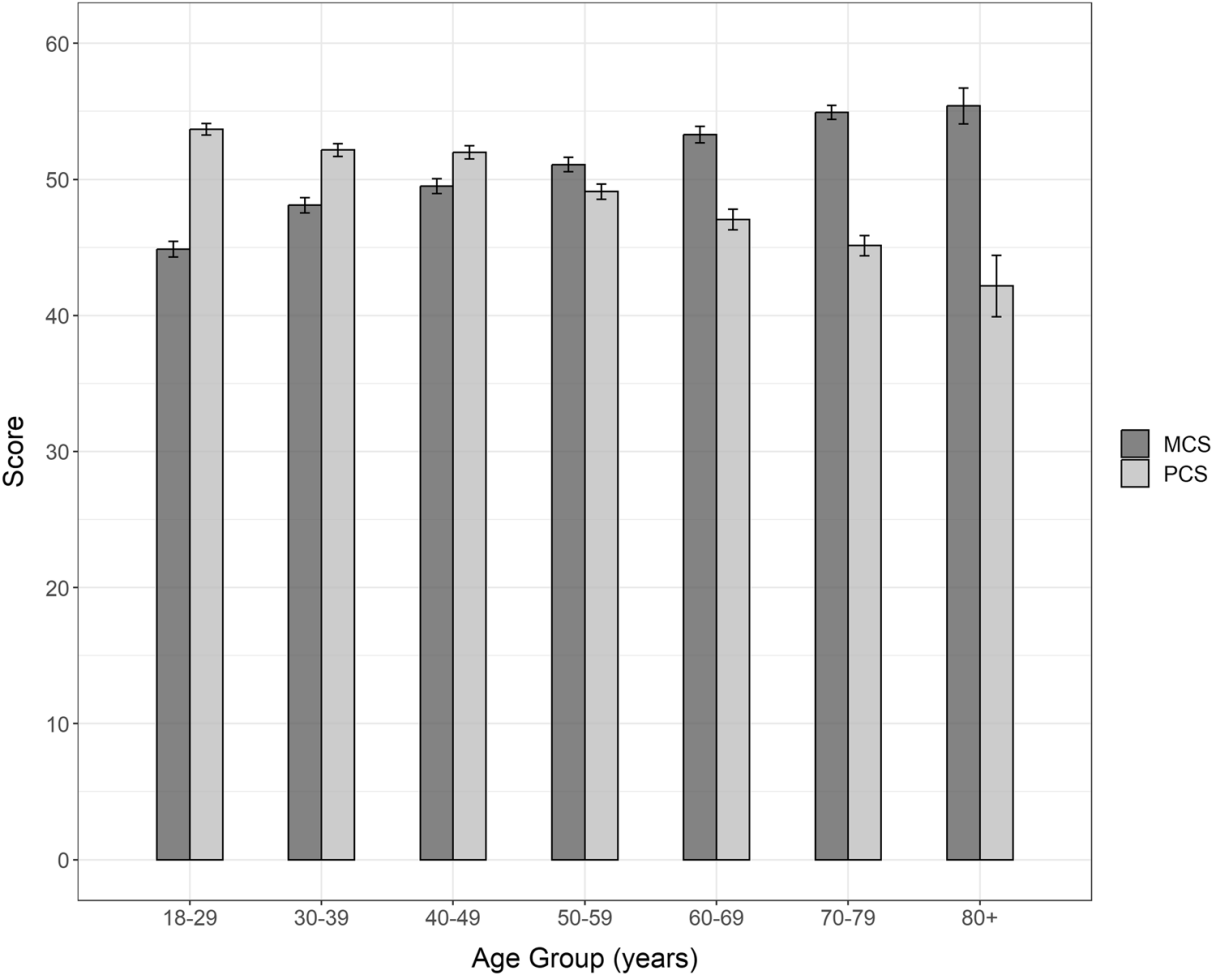
Mean VR-12 mental and physical component summary scores (MCS and PCS, respectively) and 95% confidence intervals, by age group

**Fig. 4:**
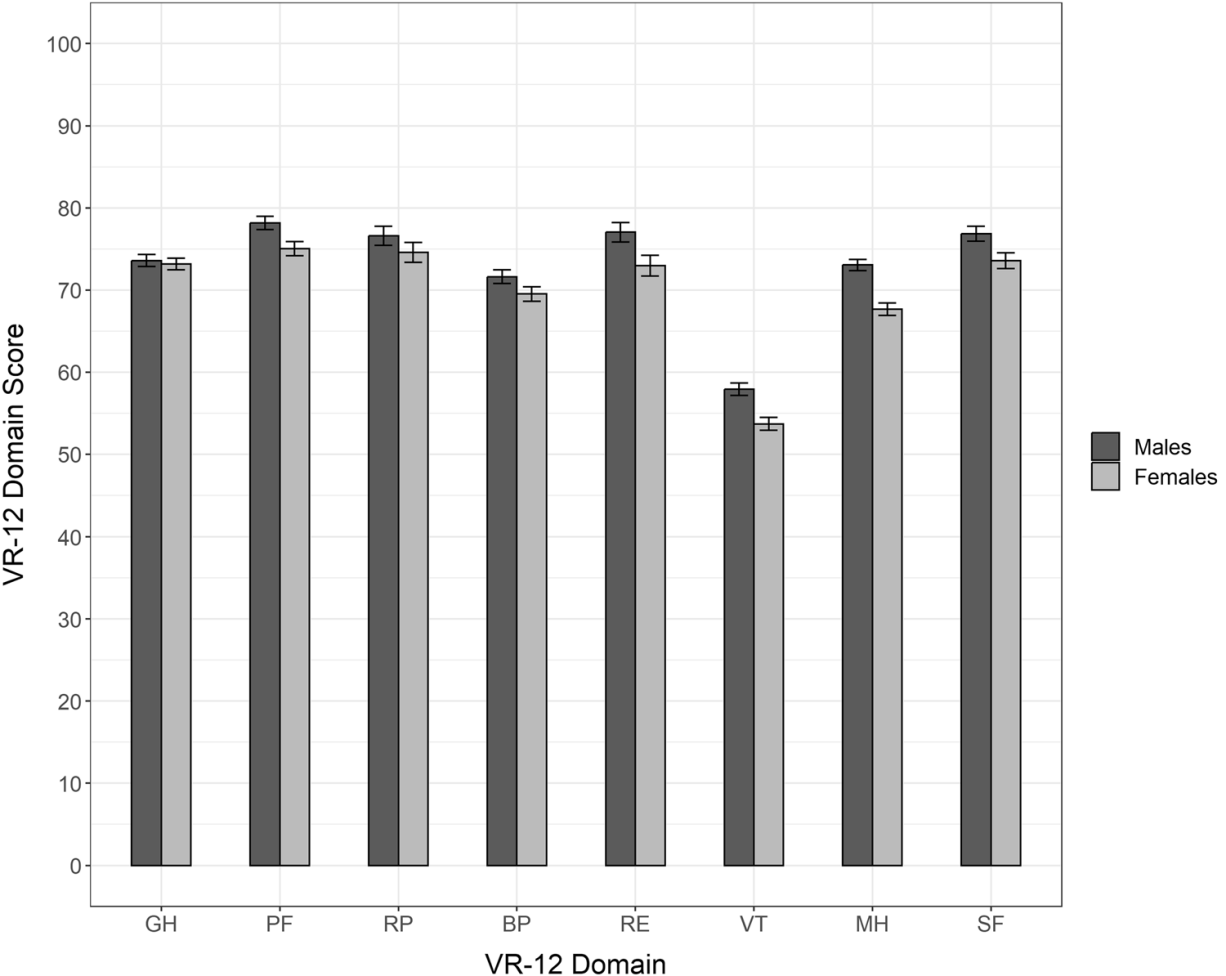
Mean VR-12 domain scores and 95% confidence intervals, by gender. BP indicates bodily pain; GH, general health; MH, mental health; PF, physical functioning; RE, role emotional; RP, role physical; SF, social functioning; VT, vitality

Summary component score norms (mean and standard deviation only) for Canadians by age group and gender are presented in Table 2. Detailed statistics summarizing Canadian population norms for the summary component scores and domain scores, by age group and gender (univariate and multivariate), are reported in SM2. Further stratifications are provided in SM3 by province (univariate), by province and age group (multivariate), and by province, age group and gender (multivariate). Figure 3 presents mean MCS and PCS scores, by age group, showing higher mental health summary scores and lower physical health summary scores in older age groups. Similar observations are made when looking at domain scores, with lower mean scores on domains most relevant to physical health (PF, RP, BP, GH) and higher mean scores on domains most relevant to mental health (VT, SF, MH, RE) in the older age groups (see SM2). The mean domain scores for females were lower compared with males for all eight VR-12 domains (see Fig. 4). Norms for the summary component scores and domain scores for 13 self-reported health conditions (univariate), and by self-reported health conditions and gender (multivariate), are reported in SM4.

## Discussion

This is the first study to present population norms for the VR-12, derived from a representative sample of Canadians. These norms provide insights into the health of Canadians, including how older Canadians tend to report higher mental health scores and lower physical health scores compared with younger Canadians, and how women tend to report lower mental and physical health scores compared with men.

There are many instruments available to measure health-related quality of life, including the VR-12 and related instruments (VR-36, SF-12, SF-36), EQ-5D instruments (EQ-5D-3L and EQ-5D-5L), the Health Utilities Index (HUI), and the PROMIS Global-10. There are Canadian population norms for the HUI3 [13], EQ-5D-3L (Alberta only) [18], EQ-5D-5L [19, 20], SF-12 (Alberta only) [18], SF-6Dv2 (Quebec only, with UK-derived preference weights) [21], and the SF-36 [37]. The Canadian Institute for Health Information (CIHI) recommends that the availability of population norms be a central consideration when choosing between available measures [38]. In reviewing available measures of health-related quality of life regarding effectiveness, meaningfulness, appropriateness, and feasibility criteria, CIHI concluded that the VR/SF instruments scored highly on all criteria relative to other measures. The VR-12 also has a Canadian health utility value set [29]—facilitating the estimation of QALYs from VR-12 data—and, based on the results presented here, population norms.

The mean VR-12 health utility value for Canadians was estimated to be 0.698. There is a large literature on health utility values. However, it is challenging to compare across studies. Different instruments conceptualize health in different ways, which results in variation in the number of component domains and the relative balance between different aspects of health [39]. Different health utility values are to be expected and may reflect underlying preferences [40–42]; however, the variation may also reflect subtle differences in the sociodemographic characteristics of the samples or the elicitation technique used [43–48]. Our estimated mean health utility value for the VR-12 (0.698) is lower than estimates from other studies. For example, studies of the SF-6D from the UK, Australia, Hong Kong, and China, estimated mean values ranging from 0.766 to 0.872 [49–53]. A Canadian study of the HUI estimated a mean health utility value of 0.863 [13], while Canadian, Chinese, and US studies of the EQ-5D-5L estimated mean values of 0.864, 0.946 and 0.851 (from the face-to-face sample), respectively [20, 52, 54]. Conversely, our estimates are similar to Australian mean age- and sex-adjusted utility norms for the Assessment of Quality of Life six-dimension (AQoL-6D) and eight-dimension (AQoL-8D) instruments, which were 0.737 and 0.680, respectively [22]. These findings might be explained by the respective components of the instruments, as the AQoL instruments and the VR-12 include a higher proportion of items related to psychosocial health when compared with variants of the SF-6D and EQ-5D instruments. For example, five of the eight dimensions of the AQoL-8D cover psychosocial health, corresponding to 25 of the 35 items [55]. Five of the eight VR-12 items used to calculate health utility values are related to mental health. A 2021 systematic review found that the SF-12, which is closely related to the VR-12, fully captured three of seven domains of mental health that were highly valued by people with mental health problems, and partially captured one additional domain [56]. By comparison, in the same study, the EQ-5D instruments fully captured one of the seven domains and partially captured two. The systematic review found that other generic (e.g., Quality of Life Index [57]) and condition-specific (e.g., Lancashire Quality of Life Profile [58]) instruments captured more of the domains of mental health that were highly valued by people with mental health problems. Neither of these instruments has a Canadian value set, which would enable their use in economic evaluation. The ability of the VR-12 to independently assess multiple mental health domains may be an advantage. Measures with fewer mental health domains may miss important aspects of mental health or conflate different domains by grouping them together. For example, the one mental health dimension included in the EQ-5D-5L measures two distinct aspects of mental health—anxiety and depression [59].

In our cross-sectional survey, we identified that people in older age groups provide VR-12 responses that correspond with higher mean health utility values. Previous studies have found different patterns with respect to age, including an age-related monotonic decline in health utility values for the SF-6D [50] and AQoL-4D [60] instruments, and a u-shaped utility curve for the AQoL-6D and AQoL-8D instruments [22]. The differences in these trajectories may reflect the items captured by each instrument. Evidence suggests that instruments with a higher proportion of items related to physical health tend to exhibit a monotonic decline, while those with a higher proportion of items related to mental health tend to exhibit a u-shaped curve given older age groups tend to report higher mental health scores [22, 37, 61]. In our study, higher scores in the mental health and social functioning domains in older Canadians seems to be driving the higher health utility scores in older ages. There was some evidence of lower physical health scores in older age groups for the bodily pain, physical functioning, and role physical domains, however these differences were modest in comparison with the age-related increase in scores on the mental health domains.

We observed that females reported lower scores than males. This finding was consistent across health utility values, summary component scores, and domain scores. A study of Medicare beneficiaries in the US that reported norms for the VR-12 also found that females reported lower mean PCS and MCS scores than males [16]. This general observation has been observed for other instruments [22, 23, 37]. It is possible men and women with the same health status may interpret and respond differently to the items (known as differential item functioning). However, a 2017 study of the VR-12, which included over 270,000 respondents, concluded there was no evidence of differential item functioning at the domain level across genders [62]. There were statistically significant differences for some items, although the magnitudes of these differences were considered negligible. This suggests that the observed gender differences may reflect differences in health status. A 2016 systematic review of self-reported health data from 59 countries found that women systematically reported lower health and functional status than males [63]. This effect was present across all age groups and in both low- and high-income countries. The authors suggested that both societal (e.g., gender inequality in employment and education) and biological (e.g., higher rates of chronic conditions in females) factors may have contributed to the findings [63].

To facilitate the use of the VR-12 norms, we have created an interactive website (https://vr12.cheos.ubc.ca/home). This website includes downloadable comma-separated value files of the health utility score, summary component score, and domain score norms by gender, age group, and province. The website also allows users with respondent-level VR-12 data to calculate VR-12 health utility scores, summary component scores, and domain scores; estimate EQ-5D-5L health utility scores based on a mapping (or ‘crosswalk’) algorithm; and obtain normative VR-12 data for their sample that is adjusted for age, gender, and geographic location.

This study has several strengths and limitations. First, our data come from a large sample of nearly 7000 respondents that were selected to be representative of the Canadian population based on age, gender, and geographic location. To further enhance the representation of our sample we used a raking procedure to weight responses to the 2021 Canadian census and 2017/18 Canadian Community Health Survey based on gender, province, education, age group, and health status. A 2010 study in the US compared online and telephone-based administration for estimating population norms for the PROMIS instrument and found that online administration with post-sampling adjustment resulted in a sample comparable to probability-based general population samples [64]. In our study, adjustment resulted in the sample more closely reflecting the characteristics of the Canadian population, particularly with respect to education and health status, although some imbalances remained. For example, our weighted sample overrepresented those who identify as ‘White’ and underrepresented selected groups, including those who identify as ‘South Asian’ and ‘Black,’ and those in the lowest household gross income bracket. Our sampling procedure, which used an online market research panel, may have resulted in other populations being underrepresented in our sample, such as those without access to a computer or new immigrants. In addition, survey respondents were able to choose to complete the survey in either English or French. Consequently, caution is necessary when generalizing these findings to populations who are underrepresented in our sample or to samples where the VR-12 was administered in one language.

## Conclusion

We have estimated Canadian VR-12 population norms for health utility scores, summary component scores, and domain scores in a large, nationally representative population sample. The health utility norms, which are reported by age group, gender, and region, can serve as a valuable input for Canadian economic models, particularly those interested in subgroup analyses. The norms for summary component scores and domain scores provide a reference standard that allows for routinely-collected data to be interpreted in the context of the Canadian population.

### Supplementary Information

Below is the link to the electronic supplementary material.

Supplementary file1 (DOCX 605 kb)
