# Supplementary material for: Quality of life of the Canadian population using the VR-12: population norms for health utility values, summary component scores and domain scores
Source: Qual Life Res. 2023 Nov 8;33(2):453–65. doi: 10.1007/s11136-023-03536-5 (PMC10850034; doi:10.1007/s11136-023-03536-5)
Supplement: Supplementary file 1 — Supplementary file1 (DOCX 605 kb) [file 11136_2023_3536_MOESM1_ESM.docx]

**SUPPLEMENTARY MATERIAL**

**Supplementary Material 1 (pages 4-7)**

**SM1 Table 1.** VR-12 items and response options, and the classification system used for deriving health utility values.

**Supplementary Material 2 (pages 8-16)**

**SM2 Table 1.** Canadian norms for VR-12 health utility values, summary component scores, and domain scores, by age group – full sample.

**SM2 Table 2.** Canadian norms for VR-12 health utility values, summary component scores, and domain scores, by age group – males only.

**SM2 Table 3**. Canadian norms for VR-12 health utility values, summary component scores, and domain scores, by age group – females only.

**Supplementary Material 3 (pages 17-112)**

**SM3 Table 1**. Canadian norms for VR-12 health utility values, summary component scores, and domain scores, by age group – British Columbia.

**SM3 Table 2**. Canadian norms for VR-12 health utility values, summary component scores, and domain scores, by age group – British Columbia, males only.

**SM3 Table 3**. Canadian norms for VR-12 health utility values, summary component scores, and domain scores, by age group – British Columbia, females only.

**SM3 Table 4**. Canadian norms for VR-12 health utility values, summary component scores, and domain scores, by age group – Alberta.

**SM3 Table 5**. Canadian norms for VR-12 health utility values, summary component scores, and domain scores, by age group – Alberta, males only.

**SM3 Table 6**. Canadian norms for VR-12 health utility values, summary component scores, and domain scores, by age group – Alberta, females only.

**SM3 Table 7**. Canadian norms for VR-12 health utility values, summary component scores, and domain scores, by age group – Saskatchewan.

**SM3 Table 8**. Canadian norms for VR-12 health utility values, summary component scores, and domain scores, by age group – Saskatchewan, males only.

**SM3 Table 9**. Canadian norms for VR-12 health utility values, summary component scores, and domain scores, by age group – Saskatchewan, females only.

**SM3 Table 10**. Canadian norms for VR-12 health utility values, summary component scores, and domain scores, by age group – Manitoba.

**SM3 Table 11**. Canadian norms for VR-12 health utility values, summary component scores, and domain scores, by age group – Manitoba, males only.

**SM3 Table 12**. Canadian norms for VR-12 health utility values, summary component scores, and domain scores, by age group – Manitoba, females only.

**SM3 Table 13**. Canadian norms for VR-12 health utility values, summary component scores, and domain scores, by age group – Ontario.

**SM3 Table 14**. Canadian norms for VR-12 health utility values, summary component scores, and domain scores, by age group – Ontario, males only.

**SM3 Table 15**. Canadian norms for VR-12 health utility values, summary component scores, and domain scores, by age group – Ontario, females only.

**SM3 Table 16**. Canadian norms for VR-12 health utility values, summary component scores, and domain scores, by age group – Quebec.

**SM3 Table 17**. Canadian norms for VR-12 health utility values, summary component scores, and domain scores, by age group – Quebec, males only.

**SM3 Table 18**. Canadian norms for VR-12 health utility values, summary component scores, and domain scores, by age group – Quebec, females only.

**SM3 Table 19**. Canadian norms for VR-12 health utility values, summary component scores, and domain scores, by age group – Nova Scotia.

**SM3 Table 20**. Canadian norms for VR-12 health utility values, summary component scores, and domain scores, by age group – Nova Scotia, males only.

**SM3 Table 21**. Canadian norms for VR-12 health utility values, summary component scores, and domain scores, by age group – Nova Scotia, females only.

**SM3 Table 22**. Canadian norms for VR-12 health utility values, summary component scores, and domain scores, by age group – New Brunswick.

**SM3 Table 23**. Canadian norms for VR-12 health utility values, summary component scores, and domain scores, by age group – New Brunswick, males only.

**SM3 Table 24**. Canadian norms for VR-12 health utility values, summary component scores, and domain scores, by age group – New Brunswick, females only.

**SM3 Table 25**. Canadian norms for VR-12 health utility values, summary component scores, and domain scores, by age group – Newfoundland.

**SM3 Table 26**. Canadian norms for VR-12 health utility values, summary component scores, and domain scores, by age group – Newfoundland, males only.

**SM3 Table 27**. Canadian norms for VR-12 health utility values, summary component scores, and domain scores, by age group – Newfoundland, females only.

**SM3 Table 28**. Canadian norms for VR-12 health utility values, summary component scores, and domain scores, by age group – Prince Edward Island.

**SM3 Table 29**. Canadian norms for VR-12 health utility values, summary component scores, and domain scores, by age group – Prince Edward Island, males only.

**SM3 Table 30**. Canadian norms for VR-12 health utility values, summary component scores, and domain scores, by age group – Prince Edward Island, females only.

**SM3 Table 31**. Canadian norms for VR-12 health utility values, summary component scores, and domain scores, by age group – Northwest Territories.

**SM3 Table 32**. Canadian norms for VR-12 health utility values, summary component scores, and domain scores, by age group – Northwest Territories, males only.

**Supplementary Material 4 (pages 113-120)**

**SM4 Table 1**. Canadian norms for VR-12 health utility values and summary component scores, across seven self-reported health conditions (anemia or blood disease, back pain, cancer, depression, diabetes, heart disease, high blood pressure). Values are means (standard deviations).

**SM4 Table 2.** Canadian norms for VR-12 health utility values and summary component scores, across six self-reported health conditions (kidney disease, liver disease, lung disease, osteoarthritis, rheumatoid arthritis, ulcer or stomach disease). Values are means (standard deviations).

**SM4 Table 3**. Canadian norms for VR-12 health utility values, summary component scores, and domain scores, across seven self-reported health conditions (anemia or blood disease, back pain, cancer, depression, diabetes, heart disease, and high blood pressure), by gender.

**SM4 Table 4**. Canadian norms for VR-12 health utility values, summary component scores, and domain scores, across six self-reported health conditions (kidney disease, liver disease, lung disease, osteoarthritis, rheumatoid arthritis, ulcer or stomach disease), by gender.

**SM1 Table 1.** VR-12 items and response options, and the classification system used for deriving health utility values.*

| VR-12 items and response options | | Corresponding attributes and levels of the VR-12 classification system used to derive health utilities using the scoring algorithm developed by Bansback and colleagues (Bansback, *et al*., 2022)^†^ |
| --- | --- | --- |
| Q1. In general, would you say your health is: | Excellent | - |
|  | Very good | - |
|  | Good | - |
|  | Fair | - |
|  | Poor | - |
| Q2. The following questions are about activities you might do during a typical day. Does your health now limit you in these activities? If so, how much?   1. Moderate activities, such as moving a table, pushing a vacuum cleaner, bowling or playing golf? | Yes, limited a lot | Moderate activities are limited a lot |
|  | Yes, limited a little | Moderate activities are limited a little |
|  | No, not limited at all | Moderate activities are not limited at all |
|  |  | |
| 1. Climbing several flights of stairs? | Yes, limited a lot | - |
|  | Yes, limited a little | - |
|  | No, not limited at all | - |
| Q3. During the past 4 weeks, have you had any of the following problems with your work or other regular daily activities as a result of your physical health?   1. Accomplished less than you would like. | No, none of the time | Accomplish less than you would like as a result of your physical health none of the time |
|  | Yes, a little of the time | Accomplish less than you would like as a result of your physical health a little of the time |
|  | Yes, some of the time | Accomplish less than you would like as a result of your physical health some of the time |
|  | Yes, most of the time | Accomplish less than you would like as a result of your physical health most of the time |
|  | Yes, all of the time | Accomplish less than you would like as a result of your physical health all of the time |
| 1. Were limited in the kind of work or other activities. | No, none of the time | - |
|  | Yes, a little of the time | - |
|  | Yes, some of the time | - |
|  | Yes, most of the time | - |
|  | Yes, all of the time | - |
| Q4. During the past 4 weeks, have you had any of the following problems with your work or other regular daily activities as a result of any emotional problems (such as feeling depressed or anxious)?   1. Accomplished less than you would like. | No, none of the time | Accomplish less than you would like as a result of your emotional problems none of the time |
|  | Yes, a little of the time | Accomplish less than you would like as a result of your emotional problems a little of the time |
|  | Yes, some of the time | Accomplish less than you would like as a result of your emotional problems some of the time |
|  | Yes, most of the time | Accomplish less than you would like as a result of your emotional problems most of the time |
|  | Yes, all of the time | Accomplish less than you would like as a result of your emotional problems all of the time |
| 1. Didn’t do work or other activities as carefully as usual. | No, none of the time | - |
|  | Yes, a little of the time | - |
|  | Yes, some of the time | - |
|  | Yes, most of the time | - |
|  | Yes, all of the time | - |
| Q5. During the past 4 weeks, how much did pain interfere with your normal work (including both work outside the home and housework)? | Not at all | Pain does not interfere with your normal work |
|  | A little bit | Pain interferes with your normal work a little bit |
|  | Moderately | Pain interferes with your normal work moderately |
|  | Quite a bit | Pain interferes with your normal work quite a bit |
|  | Extremely | Pain interferes with your normal work extremely |
| Q6. How much of the time during the past 4 weeks:   1. Have you felt calm and peaceful? | All of the time | Feel calm and peaceful all of the time |
|  | Most of the time | Feel calm and peaceful most of the time |
|  | A good bit of the time | - |
|  | Some of the time | Feel calm and peaceful some of the time |
|  | A little of the time | Feel calm and peaceful a little of the time |
|  | None of the time | MA5: Feel calm and peaceful none of the time |
| 1. Did you have a lot of energy? | All of the time | Have a lot of energy all of the time |
|  | Most of the time | Have a lot of energy a most of the time |
|  | A good bit of the time | - |
|  | Some of the time | Have a lot of energy some of the time |
|  | A little of the time | Have a lot of energy a little of the time |
|  | None of the time | Have a lot of energy none of the time |
| 1. Have you felt downhearted and blue? | All of the time | Feel downhearted and blue all of the time |
|  | Most of the time | Feel downhearted and blue most of the time |
|  | A good bit of the time | - |
|  | Some of the time | Feel downhearted and blue some of the time |
|  | A little of the time | Feel downhearted and blue a little of the time |
|  | None of the time | Feel downhearted and blue none of the time |
| Q7. During the past 4 weeks, how much of the time has your physical health or emotional problems interfered with your social activities (like visiting with friends, relatives, etc.)? | All of the time | Health interferes with social activities all of the time |
|  | Most of the time | Health interferes with social activities most of the time |
|  | Some of the time | Health interferes with social activities some of the time |
|  | A little of the time | Health interferes with social activities a little of the time |
|  | None of the time | Health interferes with social activities none of the time |
| Q8. Compared to one year ago, how would you rate your physical health in general now? | Much better | - |
|  | Slightly better | - |
|  | About the same | - |
|  | Slightly worse | - |
|  | Much worse | - |
| Q9. Compared to one year ago, how would you rate your emotional problems (such as feeling anxious, depressed or irritable) now? | Much better | - |
|  | Slightly better | - |
|  | About the same | - |
|  | Slightly worse | - |
|  | Much worse | - |

^*^ The 14 items (across eight questions) align with the VR-12 domains as follows: general health (GH), question 1/item 1; physical functioning (PF), question 2a/item 2 and question 2b/item 3; role physical (RP), question 3a/item 4 and question 3b/item 5; bodily pain (BP), question 5/item 8; role emotional (RE), question 4a/item 6 and question 4b/item 7; vitality (VT), question 6b/item 10; mental health (MH), question 6a/item 9 and question 6c/item 11; and social functioning (SF), question 7/item 12.

^†^ The composition of the eight attributes in the classification system for deriving health utilities is different to the eight domains of the VR-12. The following naming conventions are used in the paper for the VR-12 classification system attributes: physical functioning (PF_u_), role physical (RP_u_), role emotional (RE_u_), bodily pain (BP_u_), mental health-anxiety (MA_u_), mental health-depression (MD_u_), vitality (VT_u_), and social functioning (SF_u_).

**Reference**: Bansback N, Trenaman L, Mulhern BJ, Norman R, Metcalfe R, Sawatzky R, Brazier JE, Rowen D, Whitehurst DGT. Estimation of a Canadian preference-based scoring algorithm for the Veterans RAND 12-Item Health Survey (VR-12): a population survey using a discrete-choice experiment. *CMAJ Open* 2022; 10(3): E589

**SM2 Table 1.** Canadian norms for VR-12 health utility values, summary component scores, and domain scores, by age group – full sample.

|  | All participants (n=6741) | 18 to 29 (n=1285) | 30 to 39 (n=1174) | 40 to 49 (n=1272) | 50 to 59 (n=1308) | 60 to 69 (n=749) | 70 to 79 (n=859) | 80 and over (n=94) |
| --- | --- | --- | --- | --- | --- | --- | --- | --- |
| *VR-12 health utility values* |  |  |  |  |  |  |  |  |
| Mean | 0.698 | 0.661 | 0.686 | 0.710 | 0.697 | 0.724 | 0.721 | 0.728 |
| Standard deviation | 0.216 | 0.214 | 0.221 | 0.194 | 0.228 | 0.231 | 0.186 | 0.310 |
| Median | 0.749 | 0.708 | 0.740 | 0.765 | 0.765 | 0.777 | 0.777 | 0.758 |
| 25th percentile | 0.644 | 0.575 | 0.615 | 0.644 | 0.644 | 0.644 | 0.644 | 0.696 |
| 75th percentile | 0.832 | 0.799 | 0.818 | 0.852 | 0.852 | 0.852 | 0.852 | 0.830 |
| Minimum | -0.590 | -0.396 | -0.419 | -0.590 | -0.590 | -0.393 | -0.590 | 0.097 |
| Maximum | 1.000 | 1.000 | 1.000 | 1.000 | 1.000 | 1.000 | 1.000 | 0.952 |
| *VR-12 summary component score: PCS* |  |  |  |  |  |  |  |  |
| Mean | 50.000 | 53.686 | 52.155 | 51.985 | 49.096 | 47.053 | 45.128 | 42.165 |
| Standard deviation | 10.000 | 7.598 | 8.372 | 7.975 | 10.041 | 12.697 | 9.608 | 19.407 |
| Median | 53.024 | 55.769 | 54.704 | 54.488 | 52.570 | 49.729 | 47.504 | 42.029 |
| 25th percentile | 44.808 | 49.641 | 47.632 | 48.289 | 43.677 | 40.380 | 38.036 | 35.826 |
| 75th percentile | 57.387 | 58.563 | 57.908 | 57.896 | 56.824 | 56.229 | 54.857 | 50.659 |
| Minimum | 4.409 | 9.897 | 9.897 | 10.494 | 5.715 | 12.719 | 7.468 | 4.409 |
| Maximum | 73.129 | 70.390 | 66.466 | 73.129 | 67.108 | 68.411 | 65.528 | 59.961 |
| *VR-12 summary component score: MCS* |  |  |  |  |  |  |  |  |
| Mean | 50.000 | 44.874 | 48.099 | 49.511 | 51.090 | 53.298 | 54.928 | 55.402 |
| Standard deviation | 10.000 | 10.609 | 9.842 | 9.174 | 9.181 | 9.985 | 6.581 | 11.337 |
| Median | 52.293 | 46.248 | 48.825 | 52.357 | 53.361 | 55.480 | 56.980 | 57.518 |
| 25th percentile | 44.219 | 37.710 | 42.195 | 43.910 | 46.315 | 48.577 | 52.323 | 51.999 |
| 75th percentile | 57.666 | 52.916 | 55.756 | 56.980 | 58.364 | 59.588 | 60.120 | 60.516 |
| Minimum | 8.848 | 8.848 | 14.330 | 8.883 | 10.612 | 17.768 | 18.033 | 34.123 |
| Maximum | 70.517 | 67.290 | 67.325 | 68.555 | 70.087 | 67.974 | 70.517 | 65.043 |
| *VR-12 domain scores* |  |  |  |  |  |  |  |  |
| PF    Mean | 76.577 | 81.573 | 80.683 | 81.579 | 75.218 | 71.067 | 67.507 | 63.736 |
| Standard deviation | 24.355 | 19.887 | 21.317 | 19.462 | 25.053 | 31.591 | 24.166 | 45.696 |
| Median | 93.485 | 93.485 | 93.485 | 93.485 | 93.485 | 80.375 | 80.375 | 58.290 |
| 25th percentile | 58.290 | 71.400 | 71.400 | 71.400 | 58.290 | 58.290 | 58.290 | 58.290 |
| 75th percentile | 93.485 | 93.485 | 93.485 | 93.485 | 93.485 | 93.485 | 93.485 | 93.485 |
| Minimum | 7.765 | 7.765 | 7.765 | 7.765 | 7.765 | 7.765 | 7.765 | 7.765 |
| Maximum | 93.485 | 93.485 | 93.485 | 93.485 | 93.485 | 93.485 | 93.485 | 93.485 |
| RP   Mean | 75.476 | 79.882 | 78.295 | 81.207 | 75.073 | 71.796 | 67.370 | 55.224 |
| Standard deviation | 34.961 | 30.705 | 32.879 | 29.498 | 34.482 | 43.817 | 33.493 | 69.994 |
| Median | 90.425 | 100.690 | 100.690 | 100.690 | 90.425 | 89.670 | 89.670 | 79.405 |
| 25th percentile | 54.725 | 79.405 | 79.405 | 79.405 | 54.725 | 44.460 | 9.775 | 9.775 |
| 75th percentile | 100.690 | 100.690 | 100.690 | 100.690 | 100.690 | 100.690 | 100.690 | 90.425 |
| Minimum | -3.670 | -3.670 | -3.670 | -3.670 | -3.670 | -3.670 | -3.670 | -3.670 |
| Maximum | 100.690 | 100.690 | 100.690 | 100.690 | 100.690 | 100.690 | 100.690 | 100.690 |
| BP   Mean | 70.491 | 73.336 | 72.104 | 72.893 | 68.961 | 68.352 | 66.523 | 64.729 |
| Standard deviation | 25.141 | 23.916 | 26.186 | 22.475 | 24.702 | 29.388 | 21.651 | 44.791 |
| Median | 56.250 | 95.200 | 95.200 | 95.200 | 56.250 | 56.250 | 56.250 | 56.250 |
| 25th percentile | 56.250 | 56.250 | 56.250 | 56.250 | 56.250 | 56.250 | 43.890 | 43.890 |
| 75th percentile | 95.200 | 95.200 | 95.200 | 95.200 | 95.200 | 95.200 | 95.200 | 95.200 |
| Minimum | 2.280 | 2.280 | 2.280 | 2.280 | 2.280 | 2.280 | 2.280 | 2.280 |
| Maximum | 95.200 | 95.200 | 95.200 | 95.200 | 95.200 | 95.200 | 95.200 | 95.200 |
| GH   Mean | 73.305 | 78.115 | 77.171 | 75.087 | 71.318 | 69.486 | 67.892 | 62.950 |
| Standard deviation | 21.140 | 19.374 | 19.204 | 18.701 | 19.925 | 27.052 | 19.243 | 39.916 |
| Median | 83.710 | 83.710 | 83.710 | 83.710 | 83.710 | 83.710 | 61.500 | 61.500 |
| 25th percentile | 61.500 | 61.500 | 61.500 | 61.500 | 61.500 | 61.500 | 61.500 | 37.500 |
| 75th percentile | 83.710 | 83.710 | 83.710 | 83.710 | 83.710 | 83.710 | 83.710 | 83.710 |
| Minimum | 0.580 | 0.580 | 0.580 | 0.580 | 0.580 | 0.580 | 0.580 | 0.580 |
| Maximum | 101.840 | 101.840 | 101.840 | 101.840 | 101.840 | 101.840 | 101.840 | 101.840 |
| VT    Mean | 55.688 | 54.294 | 57.081 | 56.485 | 56.010 | 56.723 | 54.945 | 49.650 |
| Standard deviation | 22.732 | 24.080 | 22.209 | 19.829 | 21.545 | 27.118 | 19.329 | 42.106 |
| Median | 54.550 | 54.550 | 54.550 | 54.550 | 54.550 | 54.550 | 54.550 | 45.900 |
| 25th percentile | 45.900 | 45.900 | 45.900 | 45.900 | 45.900 | 45.900 | 45.900 | 31.930 |
| 75th percentile | 79.250 | 79.250 | 79.250 | 79.250 | 79.250 | 79.250 | 79.250 | 79.250 |
| Minimum | 1.800 | 1.800 | 1.800 | 1.800 | 1.800 | 1.800 | 1.800 | 1.800 |
| Maximum | 95.340 | 95.340 | 95.340 | 95.340 | 95.340 | 95.340 | 95.340 | 95.340 |
| SF     Mean | 75.078 | 65.663 | 69.978 | 75.168 | 77.884 | 80.568 | 85.469 | 83.858 |
| Standard deviation | 27.896 | 29.036 | 29.710 | 26.135 | 25.631 | 29.473 | 19.972 | 37.625 |
| Median | 99.320 | 66.870 | 66.870 | 99.320 | 99.320 | 99.320 | 99.320 | 99.320 |
| 25th percentile | 48.870 | 48.870 | 48.870 | 48.870 | 66.870 | 66.870 | 66.870 | 66.870 |
| 75th percentile | 99.320 | 99.320 | 99.320 | 99.320 | 99.320 | 99.320 | 99.320 | 99.320 |
| Minimum | 2.780 | 2.780 | 2.780 | 2.780 | 2.780 | 2.780 | 2.780 | 24.910 |
| Maximum | 99.320 | 99.320 | 99.320 | 99.320 | 99.320 | 99.320 | 99.320 | 99.320 |
| MH   Mean | 70.204 | 60.966 | 67.297 | 68.493 | 72.013 | 75.933 | 78.601 | 83.366 |
| Standard deviation | 21.992 | 22.785 | 21.720 | 20.538 | 21.106 | 22.905 | 15.706 | 25.573 |
| Median | 74.865 | 60.685 | 69.490 | 74.865 | 74.865 | 83.670 | 83.670 | 92.650 |
| 25th percentile | 55.620 | 48.020 | 51.325 | 51.325 | 60.685 | 65.505 | 69.490 | 74.485 |
| 75th percentile | 83.845 | 83.670 | 83.670 | 83.670 | 89.615 | 92.650 | 92.650 | 92.650 |
| Minimum | -1.030 | -1.030 | -1.030 | -1.030 | -1.030 | -1.030 | -1.030 | 26.385 |
| Maximum | 98.595 | 98.595 | 98.595 | 98.595 | 98.595 | 98.595 | 98.595 | 98.595 |
| RE     Mean | 74.794 | 61.376 | 70.906 | 78.402 | 77.037 | 82.319 | 85.586 | 77.164 |
| Standard deviation | 36.353 | 39.066 | 38.127 | 32.329 | 35.096 | 37.170 | 26.067 | 55.178 |
| Median | 81.810 | 68.370 | 81.810 | 104.125 | 104.125 | 104.125 | 104.125 | 81.810 |
| 25th percentile | 54.690 | 29.335 | 42.775 | 54.690 | 54.690 | 68.370 | 77.005 | 54.690 |
| 75th percentile | 104.125 | 104.125 | 104.125 | 104.125 | 104.125 | 104.125 | 104.125 | 104.125 |
| Minimum | -33.415 | -33.415 | -33.415 | -33.415 | -33.415 | -33.415 | -33.415 | -13.835 |
| Maximum | 104.125 | 104.125 | 104.125 | 104.125 | 104.125 | 104.125 | 104.125 | 104.125 |

BP, bodily pain; GH, general health; MCS, mental component summary; MH, mental health; PCS, physical component summary; PF, physical functioning; RE, role emotional; RP, role physical; VT, vitality; SF, social functioning

**SM2 Table 2.** Canadian norms for VR-12 health utility values, summary component scores, and domain scores, by age group – males only.

|  | All participants (n=3358) | 18 to 29 (n=586) | 30 to 39 (n=578) | 40 to 49 (n=642) | 50 to 59 (n=678) | 60 to 69 (n=294) | 70 to 79 (n=518) | 80 and over (n=62) |
| --- | --- | --- | --- | --- | --- | --- | --- | --- |
| *VR-12 health utility values* |  |  |  |  |  |  |  |  |
| Mean | 0.721 | 0.694 | 0.712 | 0.724 | 0.731 | 0.730 | 0.752 | 0.726 |
| Standard deviation | 0.190 | 0.189 | 0.194 | 0.185 | 0.199 | 0.206 | 0.145 | 0.297 |
| Median | 0.765 | 0.727 | 0.749 | 0.778 | 0.778 | 0.768 | 0.787 | 0.758 |
| 25th percentile | 0.645 | 0.610 | 0.644 | 0.655 | 0.696 | 0.655 | 0.697 | 0.680 |
| 75th percentile | 0.847 | 0.818 | 0.818 | 0.852 | 0.859 | 0.852 | 0.879 | 0.830 |
| Minimum | -0.590 | -0.263 | -0.358 | -0.590 | -0.590 | 0.011 | -0.083 | 0.308 |
| Maximum | 1.000 | 1.000 | 1.000 | 1.000 | 1.000 | 1.000 | 1.000 | 0.952 |
| *VR-12 summary component score: PCS* |  |  |  |  |  |  |  |  |
| Mean | 50.275 | 53.846 | 52.951 | 52.128 | 49.972 | 46.394 | 46.267 | 41.369 |
| Standard deviation | 9.591 | 7.473 | 7.568 | 7.704 | 9.154 | 12.341 | 8.863 | 18.912 |
| Median | 53.423 | 55.818 | 55.699 | 54.885 | 53.423 | 49.120 | 49.383 | 42.029 |
| 25th percentile | 45.458 | 50.266 | 49.030 | 48.860 | 45.018 | 40.753 | 39.691 | 33.687 |
| 75th percentile | 57.299 | 58.519 | 57.889 | 57.763 | 57.155 | 54.704 | 54.968 | 49.465 |
| Minimum | 10.492 | 21.206 | 19.963 | 10.494 | 10.492 | 15.114 | 10.844 | 18.755 |
| Maximum | 73.129 | 69.530 | 66.466 | 73.129 | 66.529 | 63.342 | 65.528 | 59.100 |
| *VR-12 summary component score: MCS* |  |  |  |  |  |  |  |  |
| Mean | 51.075 | 46.500 | 48.933 | 50.421 | 52.307 | 54.229 | 55.927 | 55.460 |
| Standard deviation | 9.284 | 10.283 | 9.368 | 8.805 | 8.149 | 8.724 | 5.934 | 11.902 |
| Median | 53.220 | 47.132 | 49.834 | 53.398 | 53.858 | 56.171 | 57.693 | 58.285 |
| 25th percentile | 45.405 | 40.465 | 43.312 | 44.793 | 48.097 | 50.552 | 53.418 | 51.999 |
| 75th percentile | 58.376 | 54.526 | 55.788 | 57.498 | 58.928 | 59.608 | 60.478 | 60.644 |
| Minimum | 8.883 | 11.897 | 17.082 | 8.883 | 10.612 | 27.976 | 18.033 | 34.123 |
| Maximum | 70.517 | 65.964 | 67.325 | 68.555 | 70.087 | 67.226 | 70.517 | 65.043 |
| *VR-12 domain scores* |  |  |  |  |  |  |  |  |
| PF     Mean | 78.171 | 82.681 | 83.334 | 82.612 | 78.840 | 70.249 | 71.330 | 61.778 |
| Standard deviation | 23.246 | 21.035 | 19.846 | 19.058 | 21.432 | 30.694 | 21.861 | 45.829 |
| Median | 93.485 | 93.485 | 93.485 | 93.485 | 93.485 | 80.375 | 80.375 | 58.290 |
| 25th percentile | 58.290 | 80.375 | 80.375 | 80.375 | 71.400 | 58.290 | 58.290 | 36.595 |
| 75th percentile | 93.485 | 93.485 | 93.485 | 93.485 | 93.485 | 93.485 | 93.485 | 80.375 |
| Minimum | 7.765 | 7.765 | 7.765 | 7.765 | 7.765 | 7.765 | 7.765 | 7.765 |
| Maximum | 93.485 | 93.485 | 93.485 | 93.485 | 93.485 | 93.485 | 93.485 | 93.485 |
| RP     Mean | 76.604 | 80.464 | 79.670 | 82.425 | 77.112 | 72.084 | 71.622 | 55.428 |
| Standard deviation | 34.018 | 31.695 | 32.239 | 28.032 | 33.566 | 42.228 | 30.164 | 70.196 |
| Median | 100.690 | 100.690 | 100.690 | 100.690 | 100.690 | 89.670 | 89.670 | 79.405 |
| 25th percentile | 55.740 | 79.405 | 79.405 | 79.405 | 79.405 | 44.720 | 44.720 | 9.775 |
| 75th percentile | 100.690 | 100.690 | 100.690 | 100.690 | 100.690 | 100.690 | 100.690 | 89.670 |
| Minimum | -3.670 | -3.670 | -3.670 | -3.670 | -3.670 | -3.670 | -3.670 | 0.490 |
| Maximum | 100.690 | 100.690 | 100.690 | 100.690 | 100.690 | 100.690 | 100.690 | 100.690 |
| BP     Mean | 71.615 | 74.269 | 72.864 | 73.746 | 70.785 | 68.256 | 70.318 | 64.967 |
| Standard deviation | 24.357 | 24.894 | 25.905 | 21.474 | 23.097 | 27.663 | 20.401 | 45.065 |
| Median | 56.250 | 95.200 | 95.200 | 95.200 | 56.250 | 56.250 | 56.250 | 56.250 |
| 25th percentile | 56.250 | 56.250 | 56.250 | 56.250 | 56.250 | 56.250 | 56.250 | 43.890 |
| 75th percentile | 95.200 | 95.200 | 95.200 | 95.200 | 95.200 | 95.200 | 95.200 | 95.200 |
| Minimum | 2.280 | 2.280 | 2.280 | 2.280 | 2.280 | 2.280 | 2.280 | 31.500 |
| Maximum | 95.200 | 95.200 | 95.200 | 95.200 | 95.200 | 95.200 | 95.200 | 95.200 |
| GH     Mean | 73.584 | 80.479 | 79.149 | 74.772 | 72.188 | 66.007 | 68.355 | 59.664 |
| Standard deviation | 21.301 | 18.861 | 18.461 | 19.036 | 19.279 | 28.170 | 18.814 | 40.221 |
| Median | 83.710 | 83.710 | 83.710 | 83.710 | 83.710 | 61.500 | 83.710 | 61.500 |
| 25th percentile | 61.500 | 61.500 | 61.500 | 61.500 | 61.500 | 61.500 | 61.500 | 37.500 |
| 75th percentile | 83.710 | 83.710 | 83.710 | 83.710 | 83.710 | 83.710 | 83.710 | 83.710 |
| Minimum | 0.580 | 0.580 | 0.580 | 0.580 | 0.580 | 0.580 | 0.580 | 0.580 |
| Maximum | 101.840 | 101.840 | 101.840 | 101.840 | 101.840 | 101.840 | 101.840 | 101.840 |
| VT     Mean | 57.925 | 58.777 | 60.237 | 57.884 | 59.355 | 57.331 | 56.512 | 47.558 |
| Standard deviation | 21.825 | 23.168 | 21.574 | 18.450 | 20.376 | 25.603 | 19.040 | 42.644 |
| Median | 54.550 | 54.550 | 54.550 | 54.550 | 54.550 | 54.550 | 54.550 | 45.900 |
| 25th percentile | 45.900 | 45.900 | 45.900 | 45.900 | 45.900 | 45.900 | 45.900 | 31.930 |
| 75th percentile | 79.250 | 79.250 | 79.250 | 79.250 | 79.250 | 79.250 | 79.250 | 79.250 |
| Minimum | 1.800 | 1.800 | 1.800 | 1.800 | 1.800 | 1.800 | 1.800 | 1.800 |
| Maximum | 95.340 | 95.340 | 95.340 | 95.340 | 95.340 | 95.340 | 95.340 | 95.340 |
| SF     Mean | 76.839 | 67.406 | 71.950 | 77.068 | 80.357 | 81.516 | 87.112 | 83.236 |
| Standard deviation | 26.720 | 30.342 | 28.618 | 25.049 | 23.928 | 26.889 | 17.989 | 37.290 |
| Median | 99.320 | 66.870 | 66.870 | 99.320 | 99.320 | 99.320 | 99.320 | 99.320 |
| 25th percentile | 66.870 | 48.870 | 48.870 | 66.870 | 66.870 | 66.870 | 66.870 | 66.870 |
| 75th percentile | 99.320 | 99.320 | 99.320 | 99.320 | 99.320 | 99.320 | 99.320 | 99.320 |
| Minimum | 2.780 | 2.780 | 2.780 | 2.780 | 2.780 | 2.780 | 2.780 | 48.870 |
| Maximum | 99.320 | 99.320 | 99.320 | 99.320 | 99.320 | 99.320 | 99.320 | 99.320 |
| MH     Mean | 73.053 | 65.145 | 69.921 | 70.677 | 74.995 | 77.419 | 81.243 | 85.294 |
| Standard deviation | 20.293 | 22.493 | 20.203 | 19.650 | 18.632 | 20.916 | 13.666 | 23.131 |
| Median | 83.670 | 65.505 | 74.865 | 74.865 | 83.670 | 83.670 | 83.670 | 92.650 |
| 25th percentile | 60.685 | 51.325 | 55.620 | 55.620 | 65.505 | 65.505 | 74.865 | 74.865 |
| 75th percentile | 92.650 | 83.670 | 83.670 | 83.845 | 92.650 | 92.650 | 92.650 | 92.650 |
| Minimum | -1.030 | -1.030 | -1.030 | -1.030 | -1.030 | -1.030 | 6.005 | 31.800 |
| Maximum | 98.595 | 98.595 | 98.595 | 98.595 | 98.595 | 98.595 | 98.595 | 98.595 |
| RE     Mean | 77.036 | 64.133 | 71.661 | 80.418 | 80.968 | 85.845 | 89.949 | 73.437 |
| Standard deviation | 34.904 | 40.717 | 37.136 | 30.146 | 32.830 | 32.982 | 21.603 | 60.399 |
| Median | 104.125 | 77.005 | 81.810 | 104.125 | 104.125 | 104.125 | 104.125 | 77.005 |
| 25th percentile | 54.690 | 41.250 | 42.775 | 54.690 | 54.690 | 77.005 | 81.810 | 42.775 |
| 75th percentile | 104.125 | 104.125 | 104.125 | 104.125 | 104.125 | 104.125 | 104.125 | 104.125 |
| Minimum | -33.415 | -33.415 | -33.415 | -33.415 | -33.415 | -33.415 | -33.415 | -13.835 |
| Maximum | 104.125 | 104.125 | 104.125 | 104.125 | 104.125 | 104.125 | 104.125 | 104.125 |

BP, bodily pain; GH, general health; MCS, mental component summary; MH, mental health; PCS, physical component summary; PF, physical functioning; RE, role emotional; RP, role physical; VT, vitality; SF, social functioning

**SM2 Table 3**. Canadian norms for VR-12 health utility values, summary component scores, and domain scores, by age group – females only.

|  | All participants (n=3370) | 18 to 29 (n=690) | 30 to 39 (n=593) | 40 to 49 (n=630) | 50 to 59 (n=630) | 60 to 69 (n=454) | 70 to 79 (n=341) | 80 and over (n=32) |
| --- | --- | --- | --- | --- | --- | --- | --- | --- |
| *VR-12 health utility values* |  |  |  |  |  |  |  |  |
| Mean | 0.678 | 0.636 | 0.661 | 0.697 | 0.664 | 0.720 | 0.680 | 0.732 |
| Standard deviation | 0.235 | 0.223 | 0.240 | 0.202 | 0.251 | 0.246 | 0.230 | 0.338 |
| Median | 0.740 | 0.697 | 0.730 | 0.749 | 0.739 | 0.777 | 0.768 | 0.757 |
| 25th percentile | 0.605 | 0.553 | 0.567 | 0.644 | 0.605 | 0.644 | 0.615 | 0.697 |
| 75th percentile | 0.818 | 0.789 | 0.818 | 0.839 | 0.827 | 0.852 | 0.842 | 0.879 |
| Minimum | -0.590 | -0.396 | -0.419 | -0.590 | -0.590 | -0.393 | -0.590 | 0.097 |
| Maximum | 1.000 | 1.000 | 1.000 | 1.000 | 1.000 | 1.000 | 1.000 | 0.952 |
| *VR-12 summary component score: PCS* |  |  |  |  |  |  |  |  |
| Mean | 49.737 | 53.565 | 51.407 | 51.852 | 48.218 | 47.460 | 43.628 | 43.783 |
| Standard deviation | 10.370 | 7.658 | 8.940 | 8.246 | 10.855 | 12.910 | 10.507 | 20.348 |
| Median | 52.814 | 55.769 | 53.949 | 54.229 | 52.066 | 49.954 | 45.215 | 42.022 |
| 25th percentile | 44.091 | 49.421 | 46.192 | 47.970 | 42.338 | 40.009 | 35.360 | 37.812 |
| 75th percentile | 57.577 | 58.737 | 57.908 | 57.908 | 56.824 | 56.824 | 54.507 | 54.838 |
| Minimum | 4.409 | 9.897 | 9.897 | 15.070 | 5.715 | 12.719 | 7.468 | 4.409 |
| Maximum | 70.390 | 70.390 | 65.678 | 65.783 | 67.108 | 68.411 | 61.267 | 59.961 |
| *VR-12 summary component score: MCS* |  |  |  |  |  |  |  |  |
| Mean | 49.074 | 43.655 | 47.314 | 48.664 | 49.871 | 52.743 | 53.613 | 55.284 |
| Standard deviation | 10.457 | 10.386 | 10.217 | 9.472 | 10.044 | 10.678 | 7.303 | 10.333 |
| Median | 51.270 | 45.588 | 47.909 | 51.488 | 52.189 | 55.070 | 55.526 | 55.058 |
| 25th percentile | 43.225 | 36.662 | 40.514 | 42.800 | 44.169 | 46.818 | 50.362 | 51.770 |
| 75th percentile | 56.980 | 52.023 | 55.540 | 56.732 | 56.980 | 59.588 | 59.576 | 59.588 |
| Minimum | 8.848 | 8.848 | 14.330 | 18.705 | 14.148 | 17.768 | 18.832 | 39.544 |
| Maximum | 68.956 | 67.290 | 66.386 | 62.711 | 67.832 | 67.974 | 68.956 | 64.318 |
| *VR-12 domain scores* |  |  |  |  |  |  |  |  |
| PF     Mean | 75.049 | 80.551 | 78.068 | 80.617 | 71.590 | 71.572 | 62.473 | 67.717 |
| Standard deviation | 25.319 | 18.790 | 22.300 | 19.837 | 28.019 | 32.203 | 26.680 | 45.369 |
| Median | 93.485 | 93.485 | 93.485 | 93.485 | 80.375 | 80.375 | 71.400 | 58.290 |
| 25th percentile | 58.290 | 71.400 | 58.290 | 71.400 | 58.290 | 58.290 | 36.595 | 58.290 |
| 75th percentile | 93.485 | 93.485 | 93.485 | 93.485 | 93.485 | 93.485 | 93.485 | 93.485 |
| Minimum | 7.765 | 7.765 | 7.765 | 7.765 | 7.765 | 7.765 | 7.765 | 7.765 |
| Maximum | 93.485 | 93.485 | 93.485 | 93.485 | 93.485 | 93.485 | 93.485 | 93.485 |
| RP     Mean | 74.575 | 79.983 | 77.200 | 80.072 | 73.031 | 71.780 | 61.768 | 54.808 |
| Standard deviation | 35.690 | 29.101 | 33.211 | 30.904 | 35.357 | 44.754 | 37.466 | 70.715 |
| Median | 89.670 | 90.425 | 89.670 | 100.690 | 89.670 | 89.670 | 79.405 | 79.405 |
| 25th percentile | 54.725 | 79.405 | 55.740 | 79.405 | 51.210 | 44.460 | 9.775 | 9.775 |
| 75th percentile | 100.690 | 100.690 | 100.690 | 100.690 | 100.690 | 100.690 | 100.690 | 100.690 |
| Minimum | -3.670 | -3.670 | -3.670 | -3.670 | -3.670 | -3.670 | -3.670 | -3.670 |
| Maximum | 100.690 | 100.690 | 100.690 | 100.690 | 100.690 | 100.690 | 100.690 | 100.690 |
| BP     Mean | 69.511 | 72.867 | 71.481 | 72.098 | 67.134 | 68.437 | 61.525 | 64.245 |
| Standard deviation | 25.851 | 23.006 | 26.378 | 23.444 | 26.219 | 30.505 | 22.700 | 44.959 |
| Median | 56.250 | 95.200 | 95.200 | 56.250 | 56.250 | 56.250 | 56.250 | 56.250 |
| 25th percentile | 56.250 | 56.250 | 56.250 | 56.250 | 56.250 | 56.250 | 43.890 | 43.890 |
| 75th percentile | 95.200 | 95.200 | 95.200 | 95.200 | 95.200 | 95.200 | 95.200 | 95.200 |
| Minimum | 2.280 | 2.280 | 2.280 | 2.280 | 2.280 | 2.280 | 2.280 | 2.280 |
| Maximum | 95.200 | 95.200 | 95.200 | 95.200 | 95.200 | 95.200 | 95.200 | 95.200 |
| GH     Mean | 73.176 | 76.511 | 75.313 | 75.380 | 70.447 | 71.519 | 67.281 | 69.631 |
| Standard deviation | 20.762 | 18.344 | 19.610 | 18.365 | 20.577 | 26.047 | 19.893 | 37.284 |
| Median | 83.710 | 83.710 | 83.710 | 83.710 | 83.710 | 83.710 | 61.500 | 61.500 |
| 25th percentile | 61.500 | 61.500 | 61.500 | 61.500 | 61.500 | 61.500 | 61.500 | 61.500 |
| 75th percentile | 83.710 | 83.710 | 83.710 | 83.710 | 83.710 | 83.710 | 83.710 | 83.710 |
| Minimum | 0.580 | 0.580 | 0.580 | 0.580 | 0.580 | 0.580 | 0.580 | 0.580 |
| Maximum | 101.840 | 101.840 | 101.840 | 101.840 | 101.840 | 101.840 | 101.840 | 101.840 |
| VT     Mean | 53.694 | 50.535 | 53.998 | 55.181 | 52.660 | 56.398 | 52.879 | 53.904 |
| Standard deviation | 23.311 | 23.761 | 22.373 | 21.081 | 22.284 | 28.097 | 19.637 | 40.703 |
| Median | 54.550 | 45.900 | 54.550 | 54.550 | 54.550 | 54.550 | 54.550 | 45.900 |
| 25th percentile | 45.900 | 31.930 | 45.900 | 45.900 | 45.900 | 45.900 | 31.930 | 31.930 |
| 75th percentile | 79.250 | 79.250 | 79.250 | 79.250 | 79.250 | 79.250 | 79.250 | 79.250 |
| Minimum | 1.800 | 1.800 | 1.800 | 1.800 | 1.800 | 1.800 | 1.800 | 1.800 |
| Maximum | 95.340 | 95.340 | 95.340 | 95.340 | 95.340 | 95.340 | 95.340 | 95.340 |
| SF     Mean | 73.557 | 64.516 | 68.101 | 73.398 | 75.406 | 79.974 | 83.304 | 85.122 |
| Standard deviation | 28.814 | 27.540 | 30.606 | 27.109 | 27.153 | 31.051 | 22.538 | 38.771 |
| Median | 66.870 | 66.870 | 66.870 | 66.870 | 99.320 | 99.320 | 99.320 | 99.320 |
| 25th percentile | 48.870 | 48.870 | 48.870 | 48.870 | 66.870 | 66.870 | 66.870 | 66.870 |
| 75th percentile | 99.320 | 99.320 | 99.320 | 99.320 | 99.320 | 99.320 | 99.320 | 99.320 |
| Minimum | 2.780 | 2.780 | 2.780 | 2.780 | 2.780 | 2.780 | 2.780 | 24.910 |
| Maximum | 99.320 | 99.320 | 99.320 | 99.320 | 99.320 | 99.320 | 99.320 | 99.320 |
| MH     Mean | 67.674 | 57.514 | 64.834 | 66.458 | 69.027 | 75.053 | 75.120 | 79.448 |
| Standard deviation | 23.080 | 21.973 | 22.740 | 21.240 | 23.135 | 24.084 | 17.935 | 28.948 |
| Median | 74.865 | 60.685 | 65.505 | 74.485 | 74.865 | 83.670 | 83.670 | 83.670 |
| 25th percentile | 51.325 | 41.685 | 51.045 | 51.325 | 51.325 | 65.505 | 65.505 | 69.490 |
| 75th percentile | 83.670 | 74.865 | 83.670 | 83.670 | 83.670 | 92.650 | 92.650 | 92.650 |
| Minimum | -1.030 | -1.030 | -1.030 | -1.030 | -1.030 | -1.030 | -1.030 | 26.385 |
| Maximum | 98.595 | 98.595 | 98.595 | 98.595 | 98.595 | 98.595 | 98.595 | 98.595 |
| RE     Mean | 72.951 | 59.878 | 70.260 | 76.524 | 73.099 | 80.224 | 79.838 | 84.741 |
| Standard deviation | 37.369 | 36.691 | 39.132 | 34.339 | 37.016 | 39.484 | 30.968 | 41.106 |
| Median | 81.810 | 65.090 | 81.810 | 104.125 | 81.810 | 104.125 | 104.125 | 104.125 |
| 25th percentile | 54.690 | 29.335 | 42.775 | 54.690 | 54.690 | 54.690 | 54.690 | 68.370 |
| 75th percentile | 104.125 | 104.125 | 104.125 | 104.125 | 104.125 | 104.125 | 104.125 | 104.125 |
| Minimum | -33.415 | -33.415 | -33.415 | -33.415 | -33.415 | -33.415 | -33.415 | 41.250 |
| Maximum | 104.125 | 104.125 | 104.125 | 104.125 | 104.125 | 104.125 | 104.125 | 104.125 |

BP, bodily pain; GH, general health; MCS, mental component summary; MH, mental health; PCS, physical component summary; PF, physical functioning; RE, role emotional; RP, role physical; VT, vitality; SF, social functioning

**SM3 Table 1**. Canadian norms for VR-12 health utility values, summary component scores, and domain scores, by age group – British Columbia.

|  | All participants (n=1008) | 18 to 29 (n=169) | 30 to 39 (n=172) | 40 to 49 (n=176) | 50 to 59 (n=183) | 60 to 69 (n=116) | 70 to 79 (n=174) | 80 and over (n=18) |
| --- | --- | --- | --- | --- | --- | --- | --- | --- |
| *VR-12 health utility values* |  |  |  |  |  |  |  |  |
| Mean | 0.699 | 0.693 | 0.631 | 0.706 | 0.733 | 0.714 | 0.719 | 0.722 |
| Standard deviation | 0.209 | 0.153 | 0.265 | 0.177 | 0.188 | 0.242 | 0.186 | 0.376 |
| Median | 0.755 | 0.726 | 0.746 | 0.746 | 0.765 | 0.778 | 0.777 | 0.725 |
| 25th percentile | 0.644 | 0.644 | 0.525 | 0.674 | 0.696 | 0.644 | 0.684 | 0.680 |
| 75th percentile | 0.839 | 0.799 | 0.818 | 0.839 | 0.852 | 0.879 | 0.852 | 0.952 |
| Minimum | -0.590 | -0.065 | -0.390 | -0.505 | -0.312 | -0.358 | -0.590 | 0.097 |
| Maximum | 1.000 | 1.000 | 1.000 | 1.000 | 1.000 | 1.000 | 1.000 | 0.952 |
| *VR-12 summary component score* |  |  |  |  |  |  |  |  |
| PCS   Mean | 49.893 | 54.089 | 50.268 | 53.460 | 51.023 | 46.534 | 44.440 | 44.167 |
| Standard deviation | 9.879 | 6.768 | 9.241 | 6.991 | 8.698 | 14.155 | 9.141 | 17.032 |
| Median | 53.645 | 55.874 | 53.387 | 55.412 | 54.350 | 51.997 | 45.816 | 42.600 |
| 25th percentile | 44.025 | 49.936 | 44.796 | 51.436 | 46.935 | 38.369 | 38.074 | 36.603 |
| 75th percentile | 57.477 | 58.769 | 57.763 | 58.613 | 57.407 | 56.824 | 54.838 | 55.963 |
| Minimum | 11.729 | 31.921 | 17.395 | 20.038 | 11.729 | 15.114 | 15.165 | 22.304 |
| Maximum | 70.847 | 69.054 | 64.283 | 70.847 | 66.529 | 63.342 | 64.354 | 57.155 |
| MCS  Mean | 50.481 | 45.860 | 47.285 | 48.271 | 51.828 | 53.986 | 55.531 | 55.296 |
| Standard deviation | 9.282 | 8.258 | 9.790 | 9.044 | 8.435 | 9.274 | 6.666 | 12.147 |
| Median | 52.757 | 46.222 | 47.118 | 51.476 | 52.983 | 56.980 | 57.899 | 57.812 |
| 25th percentile | 43.975 | 39.630 | 38.969 | 41.302 | 48.758 | 50.200 | 52.838 | 52.125 |
| 75th percentile | 58.068 | 53.166 | 56.219 | 55.622 | 58.396 | 59.588 | 60.667 | 61.487 |
| Minimum | 13.413 | 18.089 | 22.647 | 13.413 | 20.903 | 17.768 | 18.832 | 35.101 |
| Maximum | 70.517 | 62.699 | 67.325 | 62.711 | 67.133 | 67.133 | 70.517 | 61.499 |
| *VR-12 domain scores* |  |  |  |  |  |  |  |  |
| PF     Mean | 75.928 | 79.266 | 75.792 | 84.154 | 81.499 | 68.666 | 66.353 | 67.916 |
| Standard deviation | 23.670 | 20.636 | 23.384 | 16.011 | 19.347 | 33.267 | 22.038 | 47.254 |
| Median | 93.485 | 93.485 | 93.485 | 93.485 | 93.485 | 80.375 | 71.400 | 80.375 |
| 25th percentile | 58.290 | 58.290 | 58.290 | 80.375 | 71.400 | 58.290 | 58.290 | 58.290 |
| 75th percentile | 93.485 | 93.485 | 93.485 | 93.485 | 93.485 | 93.485 | 93.485 | 93.485 |
| Minimum | 7.765 | 7.765 | 7.765 | 7.765 | 7.765 | 7.765 | 7.765 | 7.765 |
| Maximum | 93.485 | 93.485 | 93.485 | 93.485 | 93.485 | 93.485 | 93.485 | 93.485 |
| RP     Mean | 75.946 | 83.748 | 72.339 | 85.201 | 79.445 | 69.637 | 65.225 | 68.282 |
| Standard deviation | 33.436 | 23.492 | 36.867 | 26.736 | 30.908 | 44.307 | 31.026 | 60.899 |
| Median | 100.690 | 100.690 | 89.670 | 100.690 | 100.690 | 90.425 | 79.405 | 79.405 |
| 25th percentile | 55.740 | 79.405 | 44.720 | 79.405 | 79.405 | 44.460 | 9.775 | 44.720 |
| 75th percentile | 100.690 | 100.690 | 100.690 | 100.690 | 100.690 | 100.690 | 100.690 | 100.690 |
| Minimum | -3.670 | 0.490 | -3.670 | -3.670 | -3.670 | -3.670 | -3.670 | 0.490 |
| Maximum | 100.690 | 100.690 | 100.690 | 100.690 | 100.690 | 100.690 | 100.690 | 100.690 |
| BP     Mean | 70.692 | 73.764 | 67.205 | 74.548 | 74.931 | 70.310 | 64.542 | 62.843 |
| Standard deviation | 24.330 | 22.470 | 27.992 | 20.010 | 22.256 | 30.646 | 19.207 | 41.840 |
| Median | 56.250 | 95.200 | 56.250 | 95.200 | 95.200 | 95.200 | 56.250 | 56.250 |
| 25th percentile | 56.250 | 56.250 | 56.250 | 56.250 | 56.250 | 43.890 | 56.250 | 43.890 |
| 75th percentile | 95.200 | 95.200 | 95.200 | 95.200 | 95.200 | 95.200 | 95.200 | 95.200 |
| Minimum | 2.280 | 2.280 | 2.280 | 2.280 | 2.280 | 2.280 | 2.280 | 31.500 |
| Maximum | 95.200 | 95.200 | 95.200 | 95.200 | 95.200 | 95.200 | 95.200 | 95.200 |
| GH     Mean | 74.211 | 80.249 | 74.563 | 75.923 | 72.448 | 72.904 | 69.249 | 69.945 |
| Standard deviation | 19.977 | 18.392 | 18.882 | 17.001 | 18.066 | 29.021 | 17.855 | 26.348 |
| Median | 83.710 | 83.710 | 83.710 | 83.710 | 83.710 | 83.710 | 83.710 | 61.500 |
| 25th percentile | 61.500 | 61.500 | 61.500 | 61.500 | 61.500 | 61.500 | 61.500 | 61.500 |
| 75th percentile | 83.710 | 101.840 | 83.710 | 83.710 | 83.710 | 83.710 | 83.710 | 83.710 |
| Minimum | 0.580 | 0.580 | 0.580 | 0.580 | 0.580 | 0.580 | 0.580 | 37.500 |
| Maximum | 101.840 | 101.840 | 101.840 | 101.840 | 101.840 | 101.840 | 101.840 | 101.840 |
| VT     Mean | 55.541 | 56.603 | 56.212 | 54.963 | 55.984 | 57.150 | 54.127 | 49.105 |
| Standard deviation | 21.374 | 19.100 | 19.208 | 19.068 | 20.477 | 28.053 | 19.213 | 47.430 |
| Median | 54.550 | 54.550 | 54.550 | 54.550 | 54.550 | 54.550 | 54.550 | 45.900 |
| 25th percentile | 45.900 | 45.900 | 45.900 | 45.900 | 45.900 | 45.900 | 45.900 | 31.930 |
| 75th percentile | 79.250 | 79.250 | 79.250 | 79.250 | 79.250 | 79.250 | 79.250 | 79.250 |
| Minimum | 1.800 | 1.800 | 1.800 | 1.800 | 1.800 | 1.800 | 1.800 | 1.800 |
| Maximum | 95.340 | 95.340 | 95.340 | 95.340 | 95.340 | 95.340 | 95.340 | 79.250 |
| SF      Mean | 75.724 | 72.235 | 67.151 | 73.022 | 77.899 | 79.682 | 86.163 | 80.131 |
| Standard deviation | 25.587 | 25.443 | 28.340 | 24.690 | 23.563 | 28.726 | 18.762 | 34.055 |
| Median | 99.320 | 66.870 | 66.870 | 66.870 | 99.320 | 99.320 | 99.320 | 66.870 |
| 25th percentile | 48.870 | 48.870 | 48.870 | 48.870 | 66.870 | 66.870 | 66.870 | 66.870 |
| 75th percentile | 99.320 | 99.320 | 99.320 | 99.320 | 99.320 | 99.320 | 99.320 | 99.320 |
| Minimum | 2.780 | 2.780 | 2.780 | 2.780 | 2.780 | 2.780 | 2.780 | 48.870 |
| Maximum | 99.320 | 99.320 | 99.320 | 99.320 | 99.320 | 99.320 | 99.320 | 99.320 |
| MH   Mean | 70.875 | 62.824 | 65.322 | 66.212 | 73.557 | 76.099 | 79.678 | 82.867 |
| Standard deviation | 20.482 | 17.749 | 20.962 | 20.555 | 20.400 | 20.241 | 14.664 | 35.714 |
| Median | 74.865 | 60.685 | 65.505 | 74.865 | 83.670 | 83.670 | 83.670 | 92.650 |
| 25th percentile | 59.850 | 51.045 | 49.110 | 51.325 | 65.505 | 65.505 | 74.485 | 74.865 |
| 75th percentile | 83.845 | 74.865 | 83.670 | 83.670 | 92.650 | 83.845 | 92.650 | 98.595 |
| Minimum | -1.030 | 6.005 | -1.030 | -1.030 | -1.030 | -1.030 | -1.030 | 26.385 |
| Maximum | 98.595 | 98.595 | 98.595 | 98.595 | 98.595 | 98.595 | 98.595 | 98.595 |
| RE     Mean | 76.906 | 59.883 | 65.823 | 77.232 | 86.499 | 86.966 | 85.624 | 84.203 |
| Standard deviation | 34.657 | 38.190 | 39.805 | 30.895 | 27.227 | 34.159 | 24.414 | 54.696 |
| Median | 104.125 | 54.690 | 77.005 | 81.810 | 104.125 | 104.125 | 104.125 | 104.125 |
| 25th percentile | 54.690 | 29.335 | 41.250 | 54.690 | 81.810 | 81.810 | 77.005 | 68.370 |
| 75th percentile | 104.125 | 104.125 | 104.125 | 104.125 | 104.125 | 104.125 | 104.125 | 104.125 |
| Minimum | -33.415 | -27.180 | -33.415 | -33.415 | -33.415 | -33.415 | -33.415 | -13.835 |
| Maximum | 104.125 | 104.125 | 104.125 | 104.125 | 104.125 | 104.125 | 104.125 | 104.125 |

BP, bodily pain; GH, general health; MCS, mental component summary; MH, mental health; PCS, physical component summary; PF, physical functioning; RE, role emotional; RP, role physical; VT, vitality; SF, social functioning

**SM3 Table 2**. Canadian norms for VR-12 health utility values, summary component scores, and domain scores, by age group – British Columbia, males only.

|  | All participants (n=496) | 18 to 29 (n=68) | 30 to 39 (n=90) | 40 to 49 (n=88) | 50 to 59 (n=94) | 60 to 69 (n=42) | 70 to 79 (n=102) | 80 and over (n=12) |
| --- | --- | --- | --- | --- | --- | --- | --- | --- |
| *VR-12 health utility values* |  |  |  |  |  |  |  |  |
| Mean | 0.730 | 0.727 | 0.681 | 0.735 | 0.765 | 0.687 | 0.752 | 0.780 |
| Standard deviation | 0.176 | 0.157 | 0.206 | 0.157 | 0.159 | 0.250 | 0.128 | 0.264 |
| Median | 0.766 | 0.765 | 0.755 | 0.768 | 0.787 | 0.759 | 0.778 | 0.759 |
| 25th percentile | 0.688 | 0.670 | 0.554 | 0.696 | 0.746 | 0.566 | 0.707 | 0.705 |
| 75th percentile | 0.839 | 0.818 | 0.818 | 0.839 | 0.861 | 0.800 | 0.852 | 0.952 |
| Minimum | -0.505 | 0.215 | 0.044 | -0.505 | -0.109 | 0.071 | 0.124 | 0.308 |
| Maximum | 1.000 | 1.000 | 1.000 | 1.000 | 1.000 | 1.000 | 1.000 | 0.952 |
| *VR-12 summary component score* |  |  |  |  |  |  |  |  |
| PCS   Mean | 50.216 | 54.594 | 52.272 | 54.145 | 52.327 | 43.528 | 45.174 | 44.783 |
| Standard deviation | 9.939 | 6.451 | 7.520 | 5.563 | 8.034 | 17.023 | 9.483 | 17.061 |
| Median | 54.108 | 55.874 | 56.216 | 54.759 | 56.166 | 51.997 | 47.914 | 42.600 |
| 25th percentile | 44.951 | 54.277 | 47.521 | 52.490 | 47.304 | 34.738 | 38.101 | 35.826 |
| 75th percentile | 57.299 | 58.266 | 57.810 | 57.791 | 57.686 | 54.390 | 55.354 | 55.963 |
| Minimum | 15.114 | 31.921 | 23.883 | 27.971 | 20.187 | 15.114 | 15.165 | 31.487 |
| Maximum | 70.847 | 64.679 | 63.902 | 70.847 | 66.529 | 63.342 | 64.354 | 56.025 |
| MCS  Mean | 52.100 | 48.432 | 47.582 | 50.661 | 52.918 | 54.998 | 56.694 | 56.763 |
| Standard deviation | 8.686 | 7.885 | 9.981 | 7.786 | 8.404 | 7.327 | 5.870 | 11.395 |
| Median | 54.215 | 48.353 | 47.597 | 53.184 | 53.160 | 57.678 | 57.961 | 60.021 |
| 25th percentile | 46.813 | 41.625 | 41.026 | 46.223 | 49.578 | 51.732 | 53.837 | 52.125 |
| 75th percentile | 58.911 | 55.768 | 56.219 | 56.980 | 59.422 | 59.896 | 61.499 | 61.499 |
| Minimum | 13.413 | 26.855 | 22.647 | 13.413 | 24.159 | 36.629 | 26.529 | 35.101 |
| Maximum | 70.517 | 62.699 | 67.325 | 62.711 | 67.133 | 62.917 | 70.517 | 61.499 |
| *VR-12 domain scores* |  |  |  |  |  |  |  |  |
| PF     Mean | 77.521 | 78.209 | 80.977 | 88.166 | 84.748 | 62.760 | 71.037 | 66.456 |
| Standard deviation | 22.997 | 24.607 | 19.304 | 11.610 | 14.853 | 35.256 | 21.722 | 47.528 |
| Median | 93.485 | 93.485 | 93.485 | 93.485 | 93.485 | 58.290 | 80.375 | 80.375 |
| 25th percentile | 58.290 | 58.290 | 71.400 | 93.485 | 80.375 | 58.290 | 58.290 | 29.460 |
| 75th percentile | 93.485 | 93.485 | 93.485 | 93.485 | 93.485 | 93.485 | 93.485 | 93.485 |
| Minimum | 7.765 | 7.765 | 7.765 | 7.765 | 29.460 | 7.765 | 7.765 | 29.460 |
| Maximum | 93.485 | 93.485 | 93.485 | 93.485 | 93.485 | 93.485 | 93.485 | 93.485 |
| RP     Mean | 77.953 | 82.704 | 77.011 | 88.970 | 83.190 | 66.978 | 66.105 | 76.616 |
| Standard deviation | 32.078 | 25.820 | 31.101 | 19.266 | 30.357 | 49.428 | 31.967 | 52.083 |
| Median | 100.690 | 100.690 | 89.670 | 100.690 | 100.690 | 89.670 | 79.405 | 79.405 |
| 25th percentile | 79.405 | 55.740 | 54.725 | 79.405 | 79.405 | 9.775 | 9.775 | 79.405 |
| 75th percentile | 100.690 | 100.690 | 100.690 | 100.690 | 100.690 | 100.690 | 100.690 | 100.690 |
| Minimum | -3.670 | 0.490 | -3.670 | -3.670 | -3.670 | -3.670 | -3.670 | 5.020 |
| Maximum | 100.690 | 100.690 | 100.690 | 100.690 | 100.690 | 100.690 | 100.690 | 100.690 |
| BP     Mean | 72.972 | 81.234 | 72.606 | 75.488 | 77.045 | 65.504 | 66.823 | 66.581 |
| Standard deviation | 23.109 | 22.518 | 22.316 | 17.501 | 22.536 | 34.321 | 17.581 | 41.729 |
| Median | 95.200 | 95.200 | 56.250 | 95.200 | 95.200 | 56.250 | 56.250 | 56.250 |
| 25th percentile | 56.250 | 56.250 | 56.250 | 56.250 | 56.250 | 43.890 | 56.250 | 43.890 |
| 75th percentile | 95.200 | 95.200 | 95.200 | 95.200 | 95.200 | 95.200 | 95.200 | 95.200 |
| Minimum | 2.280 | 2.280 | 2.280 | 31.500 | 2.280 | 2.280 | 2.280 | 31.500 |
| Maximum | 95.200 | 95.200 | 95.200 | 95.200 | 95.200 | 95.200 | 95.200 | 95.200 |
| GH    Mean | 74.191 | 82.997 | 75.463 | 75.700 | 74.667 | 68.578 | 69.132 | 69.029 |
| Standard deviation | 21.576 | 20.613 | 19.001 | 15.007 | 18.779 | 37.448 | 20.549 | 26.044 |
| Median | 83.710 | 83.710 | 83.710 | 83.710 | 83.710 | 83.710 | 83.710 | 61.500 |
| 25th percentile | 61.500 | 83.710 | 61.500 | 61.500 | 61.500 | 61.500 | 61.500 | 61.500 |
| 75th percentile | 83.710 | 101.840 | 83.710 | 83.710 | 83.710 | 101.840 | 83.710 | 83.710 |
| Minimum | 0.580 | 0.580 | 0.580 | 0.580 | 0.580 | 0.580 | 0.580 | 37.500 |
| Maximum | 101.840 | 101.840 | 101.840 | 101.840 | 101.840 | 101.840 | 101.840 | 83.710 |
| VT     Mean | 57.625 | 62.025 | 58.086 | 58.100 | 59.979 | 57.716 | 52.033 | 51.684 |
| Standard deviation | 21.454 | 20.070 | 19.301 | 16.706 | 19.756 | 28.094 | 19.989 | 51.582 |
| Median | 54.550 | 54.550 | 54.550 | 54.550 | 54.550 | 54.550 | 54.550 | 54.550 |
| 25th percentile | 45.900 | 45.900 | 45.900 | 45.900 | 54.550 | 45.900 | 31.930 | 31.930 |
| 75th percentile | 79.250 | 79.250 | 79.250 | 79.250 | 79.250 | 79.250 | 79.250 | 79.250 |
| Minimum | 1.800 | 1.800 | 1.800 | 1.800 | 1.800 | 1.800 | 1.800 | 1.800 |
| Maximum | 95.340 | 95.340 | 95.340 | 95.340 | 95.340 | 95.340 | 95.340 | 79.250 |
| SF      Mean | 78.619 | 76.923 | 66.947 | 79.844 | 80.745 | 76.754 | 90.408 | 82.603 |
| Standard deviation | 25.045 | 28.299 | 29.093 | 20.364 | 22.709 | 29.914 | 16.081 | 34.843 |
| Median | 99.320 | 99.320 | 66.870 | 99.320 | 99.320 | 66.870 | 99.320 | 99.320 |
| 25th percentile | 66.870 | 66.870 | 48.870 | 66.870 | 66.870 | 66.870 | 99.320 | 66.870 |
| 75th percentile | 99.320 | 99.320 | 99.320 | 99.320 | 99.320 | 99.320 | 99.320 | 99.320 |
| Minimum | 2.780 | 2.780 | 2.780 | 2.780 | 2.780 | 2.780 | 2.780 | 48.870 |
| Maximum | 99.320 | 99.320 | 99.320 | 99.320 | 99.320 | 99.320 | 99.320 | 99.320 |
| MH    Mean | 74.722 | 68.144 | 65.559 | 73.008 | 75.948 | 76.871 | 82.762 | 89.299 |
| Standard deviation | 19.170 | 17.272 | 21.405 | 18.191 | 20.499 | 16.724 | 11.625 | 22.882 |
| Median | 83.670 | 69.490 | 65.505 | 83.670 | 83.670 | 83.670 | 83.845 | 92.650 |
| 25th percentile | 65.505 | 55.055 | 49.110 | 65.505 | 65.505 | 65.505 | 74.865 | 83.845 |
| 75th percentile | 92.650 | 83.670 | 83.670 | 83.670 | 92.650 | 83.845 | 92.650 | 98.595 |
| Minimum | -1.030 | 21.060 | -1.030 | -1.030 | -1.030 | 41.440 | 21.060 | 41.440 |
| Maximum | 98.595 | 98.595 | 98.595 | 98.595 | 98.595 | 98.595 | 98.595 | 98.595 |
| RE     Mean | 80.714 | 64.718 | 69.526 | 79.561 | 89.488 | 90.781 | 91.241 | 81.454 |
| Standard deviation | 32.786 | 45.036 | 33.610 | 26.969 | 26.084 | 30.509 | 19.929 | 63.223 |
| Median | 104.125 | 81.810 | 77.005 | 104.125 | 104.125 | 104.125 | 104.125 | 104.125 |
| 25th percentile | 54.690 | 41.250 | 41.285 | 54.690 | 81.810 | 81.810 | 81.810 | 54.690 |
| 75th percentile | 104.125 | 104.125 | 104.125 | 104.125 | 104.125 | 104.125 | 104.125 | 104.125 |
| Minimum | -33.415 | -27.180 | -27.180 | -33.415 | -33.415 | 2.250 | -13.835 | -13.835 |
| Maximum | 104.125 | 104.125 | 104.125 | 104.125 | 104.125 | 104.125 | 104.125 | 104.125 |

BP, bodily pain; GH, general health; MCS, mental component summary; MH, mental health; PCS, physical component summary; PF, physical functioning; RE, role emotional; RP, role physical; VT, vitality; SF, social functioning

**SM3 Table 3**. Canadian norms for VR-12 health utility values, summary component scores, and domain scores, by age group – British Columbia, females only.

|  | All participants (n=509) | 18 to 29 (n=100) | 30 to 39 (n=81) | 40 to 49 (n=88) | 50 to 59 (n=89) | 60 to 69 (n=73) | 70 to 79 (n=72) | 80 and over (n=6) |
| --- | --- | --- | --- | --- | --- | --- | --- | --- |
| *VR-12 health utility values* |  |  |  |  |  |  |  |  |
| Mean | 0.668 | 0.666 | 0.579 | 0.682 | 0.689 | 0.731 | 0.668 | 0.581 |
| Standard deviation | 0.234 | 0.148 | 0.313 | 0.193 | 0.209 | 0.239 | 0.243 | 0.491 |
| Median | 0.730 | 0.696 | 0.727 | 0.740 | 0.755 | 0.783 | 0.737 | 0.644 |
| 25th percentile | 0.615 | 0.635 | 0.300 | 0.644 | 0.644 | 0.644 | 0.644 | 0.300 |
| 75th percentile | 0.829 | 0.759 | 0.818 | 0.818 | 0.832 | 0.913 | 0.852 | 0.716 |
| Minimum | -0.590 | -0.065 | -0.390 | -0.322 | -0.312 | -0.358 | -0.590 | 0.097 |
| Maximum | 1.000 | 0.960 | 0.913 | 1.000 | 1.000 | 0.960 | 1.000 | 0.952 |
| *VR-12 summary component score* |  |  |  |  |  |  |  |  |
| PCS   Mean | 49.526 | 53.502 | 48.011 | 52.902 | 49.202 | 48.560 | 43.338 | 42.657 |
| Standard deviation | 9.787 | 6.967 | 10.468 | 8.169 | 9.170 | 11.861 | 8.629 | 18.330 |
| Median | 53.355 | 55.313 | 50.493 | 55.944 | 53.540 | 53.355 | 45.215 | 41.708 |
| 25th percentile | 42.975 | 47.367 | 39.253 | 48.792 | 45.404 | 39.562 | 37.396 | 36.603 |
| 75th percentile | 57.807 | 58.833 | 57.119 | 59.236 | 56.824 | 57.550 | 53.024 | 47.483 |
| Minimum | 11.729 | 33.275 | 17.395 | 20.038 | 11.729 | 21.540 | 16.031 | 22.304 |
| Maximum | 69.054 | 69.054 | 62.009 | 63.531 | 61.768 | 58.973 | 58.016 | 57.155 |
| MCS  Mean | 48.870 | 43.913 | 47.051 | 46.322 | 50.307 | 53.252 | 53.785 | 51.701 |
| Standard deviation | 9.570 | 8.105 | 9.632 | 9.815 | 8.344 | 10.220 | 7.500 | 12.640 |
| Median | 51.287 | 43.887 | 46.563 | 50.153 | 52.206 | 56.300 | 55.837 | 53.508 |
| 25th percentile | 40.626 | 39.390 | 36.717 | 37.677 | 44.917 | 46.635 | 48.917 | 48.278 |
| 75th percentile | 56.980 | 49.853 | 56.869 | 55.058 | 56.980 | 59.576 | 59.588 | 53.826 |
| Minimum | 17.768 | 18.089 | 23.507 | 24.095 | 20.903 | 17.768 | 18.832 | 39.544 |
| Maximum | 67.133 | 60.138 | 62.516 | 62.699 | 64.353 | 67.133 | 65.217 | 61.487 |
| *VR-12 domain scores* |  |  |  |  |  |  |  |  |
| PF     Mean | 74.199 | 79.488 | 70.072 | 80.883 | 76.963 | 72.487 | 59.316 | 71.494 |
| Standard deviation | 24.225 | 17.453 | 26.352 | 18.963 | 22.695 | 31.811 | 21.527 | 50.663 |
| Median | 93.485 | 93.485 | 80.375 | 93.485 | 93.485 | 93.485 | 58.290 | 93.485 |
| 25th percentile | 58.290 | 58.290 | 36.595 | 71.400 | 71.400 | 58.290 | 51.545 | 58.290 |
| 75th percentile | 93.485 | 93.485 | 93.485 | 93.485 | 93.485 | 93.485 | 80.375 | 93.485 |
| Minimum | 7.765 | 7.765 | 7.765 | 7.765 | 7.765 | 7.765 | 7.765 | 7.765 |
| Maximum | 93.485 | 93.485 | 93.485 | 93.485 | 93.485 | 93.485 | 93.485 | 93.485 |
| RP     Mean | 73.903 | 83.837 | 67.103 | 82.127 | 74.216 | 72.347 | 63.903 | 47.854 |
| Standard deviation | 34.455 | 21.770 | 42.149 | 32.371 | 31.102 | 40.602 | 29.834 | 69.471 |
| Median | 90.425 | 89.670 | 100.690 | 100.690 | 89.670 | 90.425 | 79.405 | 44.460 |
| 25th percentile | 49.970 | 79.405 | 9.775 | 79.405 | 54.725 | 44.460 | 9.775 | 9.775 |
| 75th percentile | 100.690 | 100.690 | 100.690 | 100.690 | 100.690 | 100.690 | 100.690 | 79.405 |
| Minimum | -3.670 | 0.885 | -3.670 | -3.645 | -3.670 | -3.670 | -3.670 | 0.490 |
| Maximum | 100.690 | 100.690 | 100.690 | 100.690 | 100.690 | 100.690 | 100.690 | 100.690 |
| BP     Mean | 68.503 | 68.478 | 61.184 | 73.782 | 71.979 | 73.520 | 61.114 | 53.682 |
| Standard deviation | 25.326 | 21.016 | 32.441 | 22.315 | 21.833 | 28.176 | 21.157 | 41.903 |
| Median | 56.250 | 56.250 | 56.250 | 95.200 | 95.200 | 95.200 | 56.250 | 56.250 |
| 25th percentile | 56.250 | 56.250 | 43.890 | 56.250 | 56.250 | 56.250 | 43.890 | 31.500 |
| 75th percentile | 95.200 | 95.200 | 95.200 | 95.200 | 95.200 | 95.200 | 95.200 | 56.250 |
| Minimum | 2.280 | 31.500 | 2.280 | 2.280 | 2.280 | 2.280 | 2.280 | 31.500 |
| Maximum | 95.200 | 95.200 | 95.200 | 95.200 | 95.200 | 95.200 | 95.200 | 95.200 |
| GH    Mean | 74.215 | 77.860 | 73.674 | 76.105 | 69.351 | 75.771 | 69.425 | 72.190 |
| Standard deviation | 18.333 | 16.619 | 18.917 | 18.871 | 17.037 | 22.777 | 13.268 | 29.090 |
| Median | 83.710 | 83.710 | 83.710 | 83.710 | 61.500 | 83.710 | 61.500 | 61.500 |
| 25th percentile | 61.500 | 61.500 | 61.500 | 61.500 | 61.500 | 61.500 | 61.500 | 61.500 |
| 75th percentile | 83.710 | 83.710 | 83.710 | 83.710 | 83.710 | 83.710 | 83.710 | 83.710 |
| Minimum | 0.580 | 0.580 | 0.580 | 0.580 | 0.580 | 0.580 | 37.500 | 61.500 |
| Maximum | 101.840 | 101.840 | 101.840 | 101.840 | 101.840 | 101.840 | 101.840 | 101.840 |
| VT     Mean | 53.533 | 52.677 | 54.254 | 52.405 | 50.406 | 57.004 | 57.272 | 42.787 |
| Standard deviation | 21.180 | 17.598 | 19.140 | 20.958 | 20.384 | 28.362 | 17.925 | 40.268 |
| Median | 54.550 | 54.550 | 54.550 | 54.550 | 45.900 | 54.550 | 54.550 | 45.900 |
| 25th percentile | 45.900 | 45.900 | 45.900 | 31.930 | 31.930 | 31.930 | 45.900 | 31.930 |
| 75th percentile | 79.250 | 54.550 | 79.250 | 79.250 | 79.250 | 79.250 | 79.250 | 45.900 |
| Minimum | 1.800 | 1.800 | 1.800 | 1.800 | 1.800 | 1.800 | 1.800 | 1.800 |
| Maximum | 95.340 | 95.340 | 95.340 | 95.340 | 95.340 | 95.340 | 95.340 | 79.250 |
| SF      Mean | 72.721 | 68.666 | 67.161 | 67.460 | 73.926 | 81.132 | 79.785 | 74.072 |
| Standard deviation | 25.849 | 23.025 | 27.727 | 27.384 | 24.154 | 28.113 | 21.207 | 33.356 |
| Median | 66.870 | 66.870 | 66.870 | 66.870 | 66.870 | 99.320 | 99.320 | 66.870 |
| 25th percentile | 48.870 | 48.870 | 48.870 | 48.870 | 48.870 | 66.870 | 66.870 | 66.870 |
| 75th percentile | 99.320 | 99.320 | 99.320 | 99.320 | 99.320 | 99.320 | 99.320 | 99.320 |
| Minimum | 2.780 | 2.780 | 2.780 | 2.780 | 2.780 | 2.780 | 2.780 | 48.870 |
| Maximum | 99.320 | 99.320 | 99.320 | 99.320 | 99.320 | 99.320 | 99.320 | 99.320 |
| MH   Mean | 67.030 | 58.597 | 65.354 | 60.671 | 70.220 | 75.487 | 75.044 | 67.105 |
| Standard deviation | 21.060 | 17.283 | 20.433 | 21.397 | 20.064 | 22.201 | 17.622 | 45.868 |
| Median | 74.865 | 60.685 | 65.505 | 65.505 | 83.670 | 83.670 | 83.670 | 74.865 |
| 25th percentile | 51.045 | 41.685 | 49.110 | 41.685 | 51.325 | 74.865 | 65.505 | 42.075 |
| 75th percentile | 83.670 | 74.865 | 83.845 | 74.865 | 83.670 | 92.650 | 92.650 | 83.670 |
| Minimum | -1.030 | 6.005 | 6.005 | -1.030 | -1.030 | -1.030 | -1.030 | 26.385 |
| Maximum | 98.595 | 92.650 | 98.595 | 98.595 | 98.595 | 98.595 | 98.595 | 98.595 |
| RE     Mean | 73.019 | 55.573 | 61.980 | 75.332 | 82.325 | 84.310 | 77.185 | 90.941 |
| Standard deviation | 36.096 | 32.689 | 45.811 | 34.432 | 28.147 | 36.150 | 28.588 | 34.354 |
| Median | 81.810 | 54.690 | 77.005 | 81.810 | 104.125 | 104.125 | 104.125 | 104.125 |
| 25th percentile | 54.690 | 14.165 | 41.250 | 54.690 | 77.005 | 77.005 | 54.690 | 68.370 |
| 75th percentile | 104.125 | 104.125 | 104.125 | 104.125 | 104.125 | 104.125 | 104.125 | 104.125 |
| Minimum | -33.415 | -20.070 | -33.415 | -20.070 | -33.415 | -33.415 | -33.415 | 54.690 |
| Maximum | 104.125 | 104.125 | 104.125 | 104.125 | 104.125 | 104.125 | 104.125 | 104.125 |

BP, bodily pain; GH, general health; MCS, mental component summary; MH, mental health; PCS, physical component summary; PF, physical functioning; RE, role emotional; RP, role physical; VT, vitality; SF, social functioning

**SM3 Table 4**. Canadian norms for VR-12 health utility values, summary component scores, and domain scores, by age group – Alberta.

|  | All participants (n=721) | 18 to 29 (n=163) | 30 to 39 (n=140) | 40 to 49 (n=120) | 50 to 59 (n=118) | 60 to 69 (n=77) | 70 to 79 (n=95) | 80 and over (n=8) |
| --- | --- | --- | --- | --- | --- | --- | --- | --- |
| *VR-12 health utility values* |  |  |  |  |  |  |  |  |
| Mean | 0.699 | 0.664 | 0.664 | 0.721 | 0.704 | 0.727 | 0.752 | 0.722 |
| Standard deviation | 0.229 | 0.255 | 0.244 | 0.211 | 0.238 | 0.222 | 0.154 | 0.229 |
| Median | 0.760 | 0.746 | 0.736 | 0.818 | 0.746 | 0.779 | 0.799 | 0.757 |
| 25th percentile | 0.627 | 0.615 | 0.553 | 0.644 | 0.608 | 0.644 | 0.664 | 0.555 |
| 75th percentile | 0.839 | 0.799 | 0.818 | 0.852 | 0.879 | 0.818 | 0.852 | 0.771 |
| Minimum | -0.590 | -0.396 | -0.167 | 0.014 | -0.590 | 0.102 | -0.083 | 0.497 |
| Maximum | 1.000 | 1.000 | 0.960 | 1.000 | 1.000 | 1.000 | 1.000 | 0.952 |
| *VR-12 summary component score* |  |  |  |  |  |  |  |  |
| PCS   Mean | 50.292 | 53.625 | 51.629 | 53.316 | 47.049 | 47.871 | 46.849 | 39.674 |
| Standard deviation | 10.216 | 8.712 | 9.019 | 7.142 | 10.677 | 13.078 | 9.434 | 19.320 |
| Median | 53.012 | 55.615 | 53.847 | 55.251 | 49.623 | 50.244 | 49.721 | 37.812 |
| 25th percentile | 45.194 | 51.166 | 44.461 | 49.797 | 38.799 | 45.199 | 40.022 | 26.399 |
| 75th percentile | 57.433 | 59.233 | 58.499 | 58.161 | 56.468 | 54.928 | 56.427 | 53.136 |
| Minimum | 9.897 | 9.897 | 10.759 | 26.339 | 11.837 | 21.803 | 17.868 | 25.152 |
| Maximum | 73.129 | 63.999 | 65.027 | 73.129 | 66.977 | 60.656 | 59.631 | 59.961 |
| MCS  Mean | 49.596 | 45.148 | 46.242 | 49.176 | 53.336 | 52.525 | 55.562 | 55.384 |
| Standard deviation | 10.803 | 10.734 | 11.198 | 11.276 | 9.098 | 10.926 | 5.555 | 5.065 |
| Median | 51.715 | 46.530 | 47.909 | 53.858 | 57.501 | 54.576 | 56.980 | 53.776 |
| 25th percentile | 43.835 | 41.508 | 40.447 | 43.867 | 47.228 | 48.725 | 52.349 | 52.689 |
| 75th percentile | 57.701 | 52.171 | 55.477 | 56.980 | 59.588 | 58.376 | 60.178 | 58.239 |
| Minimum | 8.883 | 12.946 | 18.750 | 8.883 | 19.683 | 27.486 | 30.067 | 52.617 |
| Maximum | 70.087 | 63.670 | 64.871 | 68.555 | 70.087 | 63.397 | 67.076 | 61.499 |
| *VR-12 domain scores* |  |  |  |  |  |  |  |  |
| PF     Mean | 77.206 | 81.938 | 78.915 | 83.923 | 72.007 | 74.251 | 69.092 | 60.655 |
| Standard deviation | 24.978 | 22.288 | 23.761 | 19.034 | 27.777 | 29.323 | 24.021 | 44.562 |
| Median | 93.485 | 93.485 | 93.485 | 93.485 | 93.485 | 80.375 | 80.375 | 58.290 |
| 25th percentile | 58.290 | 80.375 | 71.400 | 80.375 | 58.290 | 58.290 | 58.290 | 29.460 |
| 75th percentile | 93.485 | 93.485 | 93.485 | 93.485 | 93.485 | 93.485 | 93.485 | 80.375 |
| Minimum | 7.765 | 7.765 | 7.765 | 7.765 | 7.765 | 7.765 | 7.765 | 7.765 |
| Maximum | 93.485 | 93.485 | 93.485 | 93.485 | 93.485 | 93.485 | 93.485 | 93.485 |
| RP     Mean | 76.212 | 81.241 | 76.811 | 80.660 | 73.281 | 75.543 | 71.714 | 37.133 |
| Standard deviation | 35.213 | 31.240 | 33.119 | 29.135 | 36.511 | 44.980 | 32.918 | 67.190 |
| Median | 89.670 | 100.690 | 89.670 | 100.690 | 90.425 | 89.670 | 90.425 | 9.775 |
| 25th percentile | 54.725 | 79.405 | 51.210 | 79.405 | 44.720 | 79.405 | 44.460 | 5.245 |
| 75th percentile | 100.690 | 100.690 | 100.690 | 100.690 | 100.690 | 100.690 | 100.690 | 79.405 |
| Minimum | -3.670 | -3.670 | -3.670 | -3.670 | -3.670 | -3.670 | -3.670 | 5.245 |
| Maximum | 100.690 | 100.690 | 100.690 | 100.690 | 100.690 | 100.690 | 100.690 | 100.690 |
| BP     Mean | 70.249 | 75.540 | 69.105 | 75.229 | 66.962 | 65.018 | 71.262 | 54.851 |
| Standard deviation | 25.489 | 25.831 | 25.936 | 22.303 | 25.191 | 29.110 | 20.609 | 39.777 |
| Median | 56.250 | 95.200 | 56.250 | 95.200 | 56.250 | 56.250 | 56.250 | 43.890 |
| 25th percentile | 56.250 | 56.250 | 43.890 | 56.250 | 43.890 | 56.250 | 56.250 | 43.890 |
| 75th percentile | 95.200 | 95.200 | 95.200 | 95.200 | 95.200 | 95.200 | 95.200 | 95.200 |
| Minimum | 2.280 | 2.280 | 2.280 | 2.280 | 2.280 | 31.500 | 31.500 | 31.500 |
| Maximum | 95.200 | 95.200 | 95.200 | 95.200 | 95.200 | 95.200 | 95.200 | 95.200 |
| GH    Mean | 73.356 | 74.458 | 74.980 | 80.452 | 68.789 | 70.135 | 68.924 | 72.842 |
| Standard deviation | 21.959 | 19.924 | 22.303 | 17.451 | 21.387 | 32.361 | 17.350 | 25.624 |
| Median | 83.710 | 83.710 | 83.710 | 83.710 | 61.500 | 83.710 | 61.500 | 61.500 |
| 25th percentile | 61.500 | 61.500 | 61.500 | 61.500 | 61.500 | 61.500 | 61.500 | 61.500 |
| 75th percentile | 83.710 | 83.710 | 83.710 | 83.710 | 83.710 | 83.710 | 83.710 | 83.710 |
| Minimum | 0.580 | 0.580 | 0.580 | 0.580 | 0.580 | 0.580 | 37.500 | 61.500 |
| Maximum | 101.840 | 101.840 | 101.840 | 101.840 | 101.840 | 101.840 | 101.840 | 101.840 |
| VT     Mean | 56.027 | 52.985 | 54.290 | 60.258 | 56.124 | 56.786 | 59.488 | 53.563 |
| Standard deviation | 24.184 | 21.788 | 22.818 | 25.199 | 26.295 | 29.654 | 19.363 | 34.114 |
| Median | 54.550 | 54.550 | 54.550 | 54.550 | 54.550 | 54.550 | 54.550 | 45.900 |
| 25th percentile | 45.900 | 45.900 | 45.900 | 45.900 | 45.900 | 45.900 | 45.900 | 31.930 |
| 75th percentile | 79.250 | 79.250 | 79.250 | 79.250 | 79.250 | 79.250 | 79.250 | 79.250 |
| Minimum | 1.800 | 1.800 | 1.800 | 1.800 | 1.800 | 1.800 | 1.800 | 31.930 |
| Maximum | 95.340 | 95.340 | 95.340 | 95.340 | 95.340 | 95.340 | 95.340 | 79.250 |
| SF      Mean | 73.772 | 64.595 | 63.302 | 75.009 | 81.139 | 81.622 | 88.815 | 81.467 |
| Standard deviation | 29.509 | 29.855 | 33.923 | 27.468 | 24.373 | 30.579 | 15.099 | 33.890 |
| Median | 66.870 | 66.870 | 66.870 | 66.870 | 99.320 | 99.320 | 99.320 | 99.320 |
| 25th percentile | 48.870 | 48.870 | 48.870 | 66.870 | 66.870 | 66.870 | 66.870 | 66.870 |
| 75th percentile | 99.320 | 99.320 | 99.320 | 99.320 | 99.320 | 99.320 | 99.320 | 99.320 |
| Minimum | 2.780 | 2.780 | 2.780 | 2.780 | 2.780 | 24.910 | 24.910 | 48.870 |
| Maximum | 99.320 | 99.320 | 99.320 | 99.320 | 99.320 | 99.320 | 99.320 | 99.320 |
| MH   Mean | 69.070 | 62.579 | 63.613 | 66.497 | 75.635 | 74.073 | 77.715 | 82.619 |
| Standard deviation | 24.090 | 22.566 | 24.344 | 24.386 | 23.338 | 29.043 | 15.541 | 17.889 |
| Median | 74.865 | 60.685 | 65.505 | 74.865 | 83.670 | 83.670 | 83.670 | 74.865 |
| 25th percentile | 51.325 | 51.045 | 51.045 | 51.325 | 65.505 | 55.620 | 65.505 | 74.865 |
| 75th percentile | 83.845 | 74.865 | 83.670 | 83.670 | 92.650 | 92.650 | 92.650 | 92.650 |
| Minimum | -1.030 | -1.030 | 6.005 | -1.030 | -1.030 | -1.030 | 21.060 | 65.505 |
| Maximum | 98.595 | 98.595 | 98.595 | 98.595 | 98.595 | 98.595 | 98.595 | 98.595 |
| RE     Mean | 74.478 | 63.133 | 66.455 | 78.198 | 81.494 | 80.618 | 90.657 | 76.619 |
| Standard deviation | 37.290 | 39.427 | 40.729 | 34.747 | 33.999 | 37.559 | 23.329 | 52.369 |
| Median | 81.810 | 68.370 | 81.810 | 104.125 | 104.125 | 81.810 | 104.125 | 81.810 |
| 25th percentile | 54.690 | 41.250 | 29.335 | 54.690 | 54.690 | 54.690 | 81.810 | 68.370 |
| 75th percentile | 104.125 | 104.125 | 104.125 | 104.125 | 104.125 | 104.125 | 104.125 | 104.125 |
| Minimum | -33.415 | -33.415 | -20.070 | -33.415 | -33.415 | -33.415 | -20.070 | 14.165 |
| Maximum | 104.125 | 104.125 | 104.125 | 104.125 | 104.125 | 104.125 | 104.125 | 104.125 |

BP, bodily pain; GH, general health; MCS, mental component summary; MH, mental health; PCS, physical component summary; PF, physical functioning; RE, role emotional; RP, role physical; VT, vitality; SF, social functioning

**SM3 Table 5.** Canadian norms for VR-12 health utility values, summary component scores, and domain scores, by age group – Alberta, males only.

|  | All participants (n=338) | 18 to 29 (n=67) | 30 to 39 (n=58) | 40 to 49 (n=68) | 50 to 59 (n=56) | 60 to 69 (n=29) | 70 to 79 (n=56) | 80 and over (n=4) |
| --- | --- | --- | --- | --- | --- | --- | --- | --- |
| *VR-12 health utility values* |  |  |  |  |  |  |  |  |
| Mean | 0.721 | 0.694 | 0.707 | 0.724 | 0.739 | 0.712 | 0.771 | - |
| Standard deviation | 0.214 | 0.238 | 0.210 | 0.219 | 0.232 | 0.215 | 0.157 | - |
| Median | 0.765 | 0.765 | 0.746 | 0.818 | 0.776 | 0.750 | 0.818 | - |
| 25th percentile | 0.644 | 0.579 | 0.644 | 0.645 | 0.697 | 0.606 | 0.704 | - |
| 75th percentile | 0.852 | 0.858 | 0.832 | 0.852 | 0.913 | 0.802 | 0.912 | - |
| Minimum | -0.590 | -0.174 | -0.167 | 0.051 | -0.590 | 0.108 | -0.083 | - |
| Maximum | 1.000 | 0.952 | 0.960 | 1.000 | 1.000 | 1.000 | 1.000 | - |
| *VR-12 summary component score* |  |  |  |  |  |  |  |  |
| PCS   Mean | 50.685 | 55.476 | 52.358 | 53.152 | 46.733 | 46.831 | 47.494 | - |
| Standard deviation | 10.295 | 7.026 | 9.084 | 7.752 | 10.693 | 15.438 | 8.954 | - |
| Median | 54.585 | 56.105 | 56.134 | 56.571 | 49.623 | 52.687 | 51.199 | - |
| 25th percentile | 44.683 | 52.891 | 44.017 | 47.083 | 38.196 | 43.213 | 42.773 | - |
| 75th percentile | 57.763 | 59.759 | 58.769 | 58.216 | 56.824 | 54.704 | 55.921 | - |
| Minimum | 15.290 | 29.218 | 23.330 | 26.339 | 15.290 | 21.803 | 17.868 | - |
| Maximum | 73.129 | 63.999 | 65.027 | 73.129 | 58.728 | 59.070 | 58.491 | - |
| MCS  Mean | 50.902 | 46.097 | 47.733 | 50.338 | 55.171 | 53.164 | 56.405 | - |
| Standard deviation | 10.328 | 11.195 | 10.155 | 11.477 | 8.302 | 9.172 | 5.306 | - |
| Median | 53.858 | 45.611 | 48.825 | 53.858 | 58.568 | 55.070 | 57.832 | - |
| 25th percentile | 44.742 | 43.750 | 40.970 | 44.024 | 54.017 | 46.574 | 55.375 | - |
| 75th percentile | 58.376 | 55.254 | 55.768 | 57.678 | 59.588 | 58.062 | 60.478 | - |
| Minimum | 8.883 | 16.451 | 25.684 | 8.883 | 20.625 | 36.243 | 30.067 | - |
| Maximum | 70.087 | 61.499 | 64.871 | 68.555 | 70.087 | 62.731 | 63.903 | - |
| *VR-12 domain scores* |  |  |  |  |  |  |  |  |
| PF     Mean | 78.475 | 86.080 | 81.085 | 81.644 | 73.798 | 72.536 | 72.854 | - |
| Standard deviation | 24.801 | 19.195 | 23.397 | 21.784 | 25.480 | 36.101 | 22.422 | - |
| Median | 93.485 | 93.485 | 93.485 | 93.485 | 93.485 | 93.485 | 93.485 | - |
| 25th percentile | 58.290 | 93.485 | 71.400 | 80.375 | 58.290 | 58.290 | 58.290 | - |
| 75th percentile | 93.485 | 93.485 | 93.485 | 93.485 | 93.485 | 93.485 | 93.485 | - |
| Minimum | 7.765 | 7.765 | 7.765 | 7.765 | 7.765 | 7.765 | 7.765 | - |
| Maximum | 93.485 | 93.485 | 93.485 | 93.485 | 93.485 | 93.485 | 93.485 | - |
| RP     Mean | 76.413 | 86.678 | 74.553 | 81.098 | 71.250 | 71.400 | 74.278 | - |
| Standard deviation | 36.650 | 28.656 | 37.200 | 31.922 | 40.937 | 48.297 | 31.335 | - |
| Median | 100.690 | 100.690 | 90.425 | 100.690 | 100.690 | 89.670 | 100.690 | - |
| 25th percentile | 55.740 | 79.405 | 55.740 | 79.405 | 40.165 | 54.725 | 44.460 | - |
| 75th percentile | 100.690 | 100.690 | 100.690 | 100.690 | 100.690 | 100.690 | 100.690 | - |
| Minimum | -3.670 | -3.670 | -3.645 | -3.670 | -3.670 | 0.465 | -3.670 | - |
| Maximum | 100.690 | 100.690 | 100.690 | 100.690 | 100.690 | 100.690 | 100.690 | - |
| BP     Mean | 72.372 | 81.755 | 69.682 | 77.109 | 68.368 | 61.460 | 72.998 | - |
| Standard deviation | 25.244 | 23.382 | 27.107 | 21.455 | 27.261 | 28.453 | 19.630 | - |
| Median | 95.200 | 95.200 | 56.250 | 95.200 | 56.250 | 56.250 | 95.200 | - |
| 25th percentile | 56.250 | 56.250 | 43.890 | 56.250 | 43.890 | 56.250 | 56.250 | - |
| 75th percentile | 95.200 | 95.200 | 95.200 | 95.200 | 95.200 | 56.250 | 95.200 | - |
| Minimum | 2.280 | 2.280 | 2.280 | 2.280 | 2.280 | 31.500 | 31.500 | - |
| Maximum | 95.200 | 95.200 | 95.200 | 95.200 | 95.200 | 95.200 | 95.200 | - |
| GH    Mean | 73.876 | 75.977 | 80.070 | 81.344 | 70.178 | 62.148 | 69.754 | - |
| Standard deviation | 21.273 | 17.155 | 17.533 | 15.115 | 21.049 | 39.222 | 17.402 | - |
| Median | 83.710 | 83.710 | 83.710 | 83.710 | 83.710 | 83.710 | 61.500 | - |
| 25th percentile | 61.500 | 61.500 | 61.500 | 83.710 | 61.500 | 37.500 | 61.500 | - |
| 75th percentile | 83.710 | 83.710 | 83.710 | 83.710 | 83.710 | 83.710 | 83.710 | - |
| Minimum | 0.580 | 37.500 | 0.580 | 37.500 | 0.580 | 0.580 | 37.500 | - |
| Maximum | 101.840 | 101.840 | 101.840 | 101.840 | 101.840 | 83.710 | 101.840 | - |
| VT     Mean | 59.007 | 53.744 | 60.926 | 64.269 | 58.333 | 60.404 | 59.267 | - |
| Standard deviation | 22.934 | 20.838 | 22.034 | 20.149 | 27.027 | 31.664 | 17.853 | - |
| Median | 54.550 | 54.550 | 54.550 | 79.250 | 79.250 | 79.250 | 54.550 | - |
| 25th percentile | 45.900 | 45.900 | 45.900 | 45.900 | 45.900 | 45.900 | 45.900 | - |
| 75th percentile | 79.250 | 79.250 | 79.250 | 79.250 | 79.250 | 79.250 | 79.250 | - |
| Minimum | 1.800 | 1.800 | 1.800 | 1.800 | 1.800 | 1.800 | 1.800 | - |
| Maximum | 95.340 | 95.340 | 95.340 | 95.340 | 95.340 | 95.340 | 95.340 | - |
| SF      Mean | 77.816 | 69.922 | 70.245 | 77.350 | 83.270 | 83.161 | 89.243 | - |
| Standard deviation | 27.645 | 30.939 | 26.921 | 31.059 | 24.642 | 28.988 | 14.760 | - |
| Median | 99.320 | 66.870 | 66.870 | 99.320 | 99.320 | 99.320 | 99.320 | - |
| 25th percentile | 66.870 | 48.870 | 48.870 | 66.870 | 66.870 | 66.870 | 66.870 | - |
| 75th percentile | 99.320 | 99.320 | 99.320 | 99.320 | 99.320 | 99.320 | 99.320 | - |
| Minimum | 2.780 | 2.780 | 2.780 | 2.780 | 2.780 | 24.910 | 48.870 | - |
| Maximum | 99.320 | 99.320 | 99.320 | 99.320 | 99.320 | 99.320 | 99.320 | - |
| MH   Mean | 72.207 | 65.455 | 66.913 | 68.533 | 80.101 | 74.382 | 82.570 | - |
| Standard deviation | 22.420 | 24.642 | 20.845 | 23.563 | 19.519 | 26.659 | 13.325 | - |
| Median | 83.670 | 65.505 | 69.490 | 74.865 | 83.670 | 83.670 | 83.845 | - |
| 25th percentile | 59.850 | 51.325 | 51.325 | 51.325 | 74.865 | 60.685 | 74.865 | - |
| 75th percentile | 89.615 | 83.845 | 83.670 | 83.670 | 92.650 | 83.845 | 92.650 | - |
| Minimum | -1.030 | -1.030 | 16.745 | -1.030 | -1.030 | -1.030 | 21.060 | - |
| Maximum | 98.595 | 98.595 | 98.595 | 98.595 | 98.595 | 98.595 | 98.595 | - |
| RE     Mean | 76.829 | 66.530 | 64.953 | 80.663 | 88.224 | 82.403 | 90.314 | - |
| Standard deviation | 36.280 | 40.191 | 38.934 | 33.920 | 32.474 | 36.329 | 22.993 | - |
| Median | 104.125 | 68.370 | 54.690 | 104.125 | 104.125 | 104.125 | 104.125 | - |
| 25th percentile | 54.690 | 54.690 | 29.335 | 54.690 | 104.125 | 54.690 | 81.810 | - |
| 75th percentile | 104.125 | 104.125 | 104.125 | 104.125 | 104.125 | 104.125 | 104.125 | - |
| Minimum | -33.415 | -33.415 | -20.070 | -33.415 | -33.415 | 29.335 | -20.070 | - |
| Maximum | 104.125 | 104.125 | 104.125 | 104.125 | 104.125 | 104.125 | 104.125 | - |

BP, bodily pain; GH, general health; MCS, mental component summary; MH, mental health; PCS, physical component summary; PF, physical functioning; RE, role emotional; RP, role physical; VT, vitality; SF, social functioning

**SM3 Table 6**. Canadian norms for VR-12 health utility values, summary component scores, and domain scores, by age group – Alberta, females only.

|  | All participants (n=381) | 18 to 29 (n=95) | 30 to 39 (n=81) | 40 to 49 (n=52) | 50 to 59 (n=62) | 60 to 69 (n=48) | 70 to 79 (n=39) | 80 and over (n=4) |
| --- | --- | --- | --- | --- | --- | --- | --- | --- |
| *VR-12 health utility values* |  |  |  |  |  |  |  |  |
| Mean | 0.682 | 0.644 | 0.640 | 0.718 | 0.666 | 0.736 | 0.730 | - |
| Standard deviation | 0.238 | 0.263 | 0.256 | 0.202 | 0.241 | 0.227 | 0.148 | - |
| Median | 0.749 | 0.746 | 0.696 | 0.791 | 0.705 | 0.779 | 0.790 | - |
| 25th percentile | 0.605 | 0.644 | 0.536 | 0.644 | 0.547 | 0.655 | 0.573 | - |
| 75th percentile | 0.832 | 0.799 | 0.799 | 0.837 | 0.863 | 0.818 | 0.852 | - |
| Minimum | -0.396 | -0.396 | -0.120 | 0.014 | -0.383 | 0.102 | 0.297 | - |
| Maximum | 1.000 | 1.000 | 0.960 | 0.913 | 1.000 | 0.919 | 0.960 | - |
| *VR-12 summary component score* |  |  |  |  |  |  |  |  |
| PCS   Mean | 50.016 | 52.065 | 51.382 | 53.490 | 47.386 | 48.509 | 46.068 | - |
| Standard deviation | 10.061 | 9.524 | 8.394 | 6.326 | 10.741 | 11.518 | 10.160 | - |
| Median | 52.710 | 55.209 | 53.012 | 53.914 | 51.142 | 49.954 | 49.144 | - |
| 25th percentile | 45.377 | 47.026 | 44.966 | 52.546 | 41.229 | 46.072 | 36.483 | - |
| 75th percentile | 57.258 | 59.085 | 58.499 | 57.571 | 55.963 | 55.354 | 57.908 | - |
| Minimum | 9.897 | 9.897 | 23.294 | 28.666 | 11.837 | 25.564 | 22.305 | - |
| Maximum | 66.977 | 62.414 | 63.680 | 64.238 | 66.977 | 60.656 | 59.631 | - |
| MCS  Mean | 48.562 | 44.552 | 45.254 | 47.957 | 51.382 | 52.133 | 54.542 | - |
| Standard deviation | 11.043 | 10.227 | 11.895 | 10.974 | 9.491 | 11.924 | 5.835 | - |
| Median | 49.871 | 46.958 | 47.402 | 50.296 | 53.602 | 52.846 | 56.980 | - |
| 25th percentile | 42.090 | 40.022 | 39.061 | 40.628 | 46.742 | 48.725 | 50.220 | - |
| 75th percentile | 56.980 | 50.935 | 55.477 | 56.732 | 59.576 | 59.588 | 59.576 | - |
| Minimum | 12.946 | 12.946 | 18.750 | 20.761 | 19.683 | 27.486 | 39.119 | - |
| Maximum | 67.076 | 63.670 | 61.499 | 61.714 | 65.191 | 63.397 | 67.076 | - |
| *VR-12 domain scores* |  |  |  |  |  |  |  |  |
| PF     Mean | 76.220 | 78.355 | 77.881 | 86.316 | 70.099 | 75.304 | 64.540 | - |
| Standard deviation | 25.040 | 23.886 | 23.470 | 14.478 | 29.804 | 24.672 | 25.860 | - |
| Median | 80.375 | 93.485 | 93.485 | 93.485 | 93.485 | 80.375 | 71.400 | - |
| 25th percentile | 71.400 | 71.400 | 71.400 | 93.485 | 58.290 | 71.400 | 36.595 | - |
| 75th percentile | 93.485 | 93.485 | 93.485 | 93.485 | 93.485 | 80.375 | 93.485 | - |
| Minimum | 7.765 | 7.765 | 7.765 | 7.765 | 7.765 | 7.765 | 7.765 | - |
| Maximum | 93.485 | 93.485 | 93.485 | 93.485 | 93.485 | 93.485 | 93.485 | - |
| RP     Mean | 76.308 | 77.309 | 78.807 | 80.200 | 75.443 | 78.086 | 68.613 | - |
| Standard deviation | 33.708 | 31.738 | 29.431 | 25.328 | 32.216 | 43.029 | 35.285 | - |
| Median | 89.670 | 89.670 | 89.670 | 89.670 | 89.670 | 89.670 | 89.670 | - |
| 25th percentile | 54.725 | 54.725 | 51.210 | 79.405 | 79.405 | 79.405 | 9.775 | - |
| 75th percentile | 100.690 | 100.690 | 100.690 | 100.690 | 100.690 | 100.690 | 100.690 | - |
| Minimum | -3.670 | -3.670 | 0.490 | 0.490 | -3.670 | -3.670 | 0.465 | - |
| Maximum | 100.690 | 100.690 | 100.690 | 100.690 | 100.690 | 100.690 | 100.690 | - |
| BP     Mean | 68.493 | 69.935 | 69.116 | 73.255 | 65.464 | 67.203 | 69.161 | - |
| Standard deviation | 25.493 | 26.443 | 24.779 | 23.400 | 23.308 | 29.419 | 22.052 | - |
| Median | 56.250 | 56.250 | 56.250 | 56.250 | 56.250 | 56.250 | 56.250 | - |
| 25th percentile | 56.250 | 56.250 | 56.250 | 56.250 | 43.890 | 56.250 | 43.890 | - |
| 75th percentile | 95.200 | 95.200 | 95.200 | 95.200 | 95.200 | 95.200 | 95.200 | - |
| Minimum | 2.280 | 2.280 | 31.500 | 31.500 | 2.280 | 31.500 | 31.500 | - |
| Maximum | 95.200 | 95.200 | 95.200 | 95.200 | 95.200 | 95.200 | 95.200 | - |
| GH    Mean | 73.095 | 73.523 | 72.004 | 79.516 | 67.309 | 75.039 | 67.920 | - |
| Standard deviation | 22.385 | 21.470 | 24.003 | 20.214 | 21.772 | 25.761 | 17.460 | - |
| Median | 83.710 | 83.710 | 83.710 | 83.710 | 61.500 | 83.710 | 83.710 | - |
| 25th percentile | 61.500 | 61.500 | 61.500 | 61.500 | 61.500 | 61.500 | 61.500 | - |
| 75th percentile | 83.710 | 83.710 | 83.710 | 101.840 | 83.710 | 83.710 | 83.710 | - |
| Minimum | 0.580 | 0.580 | 0.580 | 0.580 | 0.580 | 37.500 | 37.500 | - |
| Maximum | 101.840 | 101.840 | 101.840 | 101.840 | 101.840 | 101.840 | 101.840 | - |
| VT     Mean | 53.710 | 52.860 | 50.144 | 56.047 | 53.771 | 54.565 | 59.755 | - |
| Standard deviation | 24.815 | 22.052 | 22.024 | 30.168 | 25.652 | 28.309 | 21.587 | - |
| Median | 54.550 | 54.550 | 45.900 | 54.550 | 54.550 | 45.900 | 79.250 | - |
| 25th percentile | 45.900 | 45.900 | 45.900 | 31.930 | 31.930 | 45.900 | 31.930 | - |
| 75th percentile | 79.250 | 79.250 | 54.550 | 79.250 | 79.250 | 79.250 | 79.250 | - |
| Minimum | 1.800 | 1.800 | 1.800 | 1.800 | 1.800 | 1.800 | 1.800 | - |
| Maximum | 95.340 | 95.340 | 95.340 | 95.340 | 95.340 | 79.250 | 95.340 | - |
| SF      Mean | 70.617 | 60.611 | 58.999 | 72.550 | 78.871 | 80.678 | 88.298 | - |
| Standard deviation | 30.428 | 28.039 | 37.393 | 21.914 | 24.147 | 31.738 | 15.755 | - |
| Median | 66.870 | 66.870 | 66.870 | 66.870 | 99.320 | 99.320 | 99.320 | - |
| 25th percentile | 48.870 | 48.870 | 24.910 | 66.870 | 66.870 | 66.870 | 66.870 | - |
| 75th percentile | 99.320 | 99.320 | 99.320 | 99.320 | 99.320 | 99.320 | 99.320 | - |
| Minimum | 2.780 | 2.780 | 2.780 | 24.910 | 2.780 | 24.910 | 24.910 | - |
| Maximum | 99.320 | 99.320 | 99.320 | 99.320 | 99.320 | 99.320 | 99.320 | - |
| MH   Mean | 66.571 | 60.512 | 61.528 | 64.360 | 70.881 | 73.882 | 71.842 | - |
| Standard deviation | 25.116 | 20.560 | 26.517 | 25.462 | 25.739 | 30.666 | 16.999 | - |
| Median | 69.490 | 60.685 | 65.505 | 74.865 | 83.670 | 83.670 | 83.670 | - |
| 25th percentile | 51.325 | 51.045 | 48.020 | 41.685 | 55.620 | 55.620 | 55.620 | - |
| 75th percentile | 83.845 | 74.865 | 83.670 | 83.670 | 92.650 | 92.650 | 83.670 | - |
| Minimum | -1.030 | -1.030 | 6.005 | -1.030 | 6.005 | 21.060 | 41.440 | - |
| Maximum | 98.595 | 98.595 | 98.595 | 92.650 | 98.595 | 98.595 | 98.595 | - |
| RE     Mean | 72.621 | 60.692 | 67.372 | 75.609 | 74.327 | 79.523 | 91.071 | - |
| Standard deviation | 38.069 | 38.797 | 42.401 | 35.933 | 34.326 | 38.589 | 24.101 | - |
| Median | 81.810 | 68.370 | 81.810 | 81.810 | 104.125 | 81.810 | 104.125 | - |
| 25th percentile | 54.690 | 41.250 | 41.250 | 41.250 | 54.690 | 54.690 | 104.125 | - |
| 75th percentile | 104.125 | 104.125 | 104.125 | 104.125 | 104.125 | 104.125 | 104.125 | - |
| Minimum | -33.415 | -33.415 | -20.070 | -33.415 | -33.415 | -33.415 | 2.250 | - |
| Maximum | 104.125 | 104.125 | 104.125 | 104.125 | 104.125 | 104.125 | 104.125 | - |

BP, bodily pain; GH, general health; MCS, mental component summary; MH, mental health; PCS, physical component summary; PF, physical functioning; RE, role emotional; RP, role physical; VT, vitality; SF, social functioning

**SM3 Table 7**. Canadian norms for VR-12 health utility values, summary component scores, and domain scores, by age group – Saskatchewan.

|  | All participants (n=203) | 18 to 29 (n=41) | 30 to 39 (n=34) | 40 to 49 (n=39) | 50 to 59 (n=39) | 60 to 69 (n=21) | 70 to 79 (n=26) | 80 and over (n=3) |
| --- | --- | --- | --- | --- | --- | --- | --- | --- |
| *VR-12 health utility values* |  |  |  |  |  |  |  |  |
| Mean | 0.699 | 0.640 | 0.667 | 0.700 | 0.696 | 0.724 | 0.784 | - |
| Standard deviation | 0.209 | 0.242 | 0.187 | 0.222 | 0.201 | 0.214 | 0.131 | - |
| Median | 0.730 | 0.716 | 0.716 | 0.778 | 0.778 | 0.808 | 0.852 | - |
| 25th percentile | 0.625 | 0.601 | 0.477 | 0.644 | 0.644 | 0.580 | 0.668 | - |
| 75th percentile | 0.852 | 0.765 | 0.818 | 0.852 | 0.879 | 0.839 | 0.879 | - |
| Minimum | -0.412 | -0.103 | -0.033 | 0.007 | -0.412 | 0.152 | 0.355 | - |
| Maximum | 1.000 | 0.960 | 0.913 | 1.000 | 0.913 | 0.913 | 0.960 | - |
| *VR-12 summary component score* |  |  |  |  |  |  |  |  |
| PCS   Mean | 49.947 | 56.149 | 50.092 | 50.894 | 46.941 | 43.959 | 49.052 | - |
| Standard deviation | 9.978 | 6.274 | 7.118 | 9.943 | 8.621 | 15.055 | 8.405 | - |
| Median | 53.547 | 57.258 | 49.660 | 53.715 | 52.254 | 49.693 | 54.752 | - |
| 25th percentile | 46.313 | 54.469 | 47.114 | 45.521 | 42.053 | 33.228 | 42.892 | - |
| 75th percentile | 56.824 | 59.688 | 55.607 | 58.625 | 56.216 | 53.024 | 55.446 | - |
| Minimum | 9.897 | 29.550 | 9.897 | 20.271 | 17.138 | 24.880 | 24.500 | - |
| Maximum | 70.390 | 70.390 | 59.730 | 61.809 | 57.258 | 58.183 | 59.100 | - |
| MCS  Mean | 50.246 | 42.435 | 47.240 | 49.300 | 51.930 | 58.049 | 54.770 | - |
| Standard deviation | 10.739 | 12.657 | 10.523 | 8.278 | 6.848 | 8.470 | 6.108 | - |
| Median | 53.107 | 44.370 | 48.217 | 52.634 | 54.390 | 58.928 | 55.110 | - |
| 25th percentile | 44.346 | 40.035 | 40.928 | 42.663 | 46.917 | 57.114 | 52.323 | - |
| 75th percentile | 58.928 | 49.191 | 57.164 | 55.070 | 59.588 | 59.588 | 60.821 | - |
| Minimum | 12.358 | 12.358 | 23.912 | 30.364 | 22.636 | 28.494 | 35.408 | - |
| Maximum | 67.226 | 60.789 | 64.854 | 62.711 | 65.243 | 67.226 | 64.049 | - |
| *VR-12 domain scores* |  |  |  |  |  |  |  |  |
| PF     Mean | 77.529 | 89.336 | 79.602 | 77.917 | 72.570 | 62.693 | 80.201 | - |
| Standard deviation | 23.820 | 10.232 | 17.630 | 24.208 | 21.177 | 41.953 | 17.993 | - |
| Median | 93.485 | 93.485 | 93.485 | 93.485 | 80.375 | 80.375 | 93.485 | - |
| 25th percentile | 71.400 | 93.485 | 71.400 | 71.400 | 58.290 | 49.705 | 71.400 | - |
| 75th percentile | 93.485 | 93.485 | 93.485 | 93.485 | 93.485 | 93.485 | 93.485 | - |
| Minimum | 7.765 | 29.460 | 7.765 | 7.765 | 7.765 | 7.765 | 7.765 | - |
| Maximum | 93.485 | 93.485 | 93.485 | 93.485 | 93.485 | 93.485 | 93.485 | - |
| RP     Mean | 76.596 | 80.552 | 74.643 | 81.079 | 75.546 | 67.305 | 77.188 | - |
| Standard deviation | 35.111 | 29.246 | 33.007 | 33.733 | 27.594 | 58.808 | 30.242 | - |
| Median | 100.690 | 100.690 | 89.670 | 100.690 | 90.425 | 100.690 | 100.690 | - |
| 25th percentile | 54.725 | 54.725 | 54.725 | 79.405 | 79.405 | 9.775 | 44.720 | - |
| 75th percentile | 100.690 | 100.690 | 100.690 | 100.690 | 100.690 | 100.690 | 100.690 | - |
| Minimum | -3.670 | 5.020 | -3.670 | -3.670 | -3.670 | -3.670 | -3.670 | - |
| Maximum | 100.690 | 100.690 | 100.690 | 100.690 | 100.690 | 100.690 | 100.690 | - |
| BP     Mean | 71.820 | 75.720 | 63.165 | 74.152 | 64.709 | 73.472 | 72.544 | - |
| Standard deviation | 23.944 | 22.812 | 22.967 | 24.985 | 20.373 | 28.250 | 22.923 | - |
| Median | 56.250 | 95.200 | 56.250 | 95.200 | 56.250 | 56.250 | 95.200 | - |
| 25th percentile | 56.250 | 56.250 | 43.890 | 56.250 | 56.250 | 56.250 | 43.890 | - |
| 75th percentile | 95.200 | 95.200 | 95.200 | 95.200 | 95.200 | 95.200 | 95.200 | - |
| Minimum | 2.280 | 31.500 | 2.280 | 2.280 | 2.280 | 31.500 | 31.500 | - |
| Maximum | 95.200 | 95.200 | 95.200 | 95.200 | 95.200 | 95.200 | 95.200 | - |
| GH    Mean | 70.276 | 79.103 | 74.452 | 71.578 | 65.064 | 64.786 | 64.344 | - |
| Standard deviation | 19.980 | 13.269 | 15.882 | 25.646 | 14.746 | 24.339 | 18.245 | - |
| Median | 83.710 | 83.710 | 83.710 | 83.710 | 61.500 | 61.500 | 61.500 | - |
| 25th percentile | 61.500 | 83.710 | 61.500 | 61.500 | 61.500 | 61.500 | 61.500 | - |
| 75th percentile | 83.710 | 83.710 | 83.710 | 83.710 | 83.710 | 83.710 | 83.710 | - |
| Minimum | 0.580 | 0.580 | 0.580 | 0.580 | 0.580 | 37.500 | 0.580 | - |
| Maximum | 101.840 | 101.840 | 101.840 | 101.840 | 83.710 | 101.840 | 101.840 | - |
| VT     Mean | 52.799 | 46.952 | 50.270 | 55.782 | 48.744 | 56.503 | 61.574 | - |
| Standard deviation | 22.557 | 24.641 | 19.670 | 20.710 | 17.216 | 28.972 | 24.512 | - |
| Median | 54.550 | 45.900 | 45.900 | 54.550 | 54.550 | 54.550 | 54.550 | - |
| 25th percentile | 45.900 | 31.930 | 45.900 | 45.900 | 31.930 | 54.550 | 45.900 | - |
| 75th percentile | 79.250 | 54.550 | 54.550 | 79.250 | 54.550 | 79.250 | 79.250 | - |
| Minimum | 1.800 | 1.800 | 1.800 | 1.800 | 1.800 | 1.800 | 1.800 | - |
| Maximum | 95.340 | 95.340 | 79.250 | 95.340 | 79.250 | 79.250 | 95.340 | - |
| SF      Mean | 76.191 | 60.222 | 67.658 | 77.258 | 81.333 | 89.405 | 89.497 | - |
| Standard deviation | 26.248 | 29.393 | 25.856 | 24.329 | 18.739 | 23.307 | 17.113 | - |
| Median | 99.320 | 48.870 | 66.870 | 99.320 | 99.320 | 99.320 | 99.320 | - |
| 25th percentile | 48.870 | 48.870 | 48.870 | 48.870 | 66.870 | 66.870 | 66.870 | - |
| 75th percentile | 99.320 | 99.320 | 99.320 | 99.320 | 99.320 | 99.320 | 99.320 | - |
| Minimum | 2.780 | 2.780 | 2.780 | 24.910 | 2.780 | 24.910 | 2.780 | - |
| Maximum | 99.320 | 99.320 | 99.320 | 99.320 | 99.320 | 99.320 | 99.320 | - |
| MH   Mean | 70.478 | 55.949 | 64.568 | 70.034 | 72.660 | 82.109 | 80.578 | - |
| Standard deviation | 22.675 | 26.123 | 22.172 | 19.890 | 16.816 | 18.509 | 14.268 | - |
| Median | 74.865 | 51.325 | 60.685 | 74.865 | 74.865 | 83.670 | 83.670 | - |
| 25th percentile | 55.620 | 49.545 | 51.325 | 60.685 | 60.685 | 74.865 | 74.865 | - |
| 75th percentile | 92.650 | 74.865 | 92.650 | 83.670 | 92.650 | 92.650 | 92.650 | - |
| Minimum | -1.030 | -1.030 | 16.745 | 6.005 | 6.005 | 26.385 | 31.800 | - |
| Maximum | 98.595 | 92.650 | 98.595 | 98.595 | 92.650 | 98.595 | 98.595 | - |
| RE     Mean | 77.394 | 61.772 | 70.312 | 71.401 | 80.487 | 98.058 | 84.890 | - |
| Standard deviation | 36.727 | 42.998 | 39.401 | 38.935 | 25.660 | 25.138 | 26.135 | - |
| Median | 104.125 | 65.090 | 104.125 | 104.125 | 104.125 | 104.125 | 104.125 | - |
| 25th percentile | 54.690 | 54.690 | 41.250 | 42.775 | 54.690 | 104.125 | 54.690 | - |
| 75th percentile | 104.125 | 104.125 | 104.125 | 104.125 | 104.125 | 104.125 | 104.125 | - |
| Minimum | -33.415 | -13.835 | -13.835 | -33.415 | -20.070 | -33.415 | 13.250 | - |
| Maximum | 104.125 | 104.125 | 104.125 | 104.125 | 104.125 | 104.125 | 104.125 | - |

BP, bodily pain; GH, general health; MCS, mental component summary; MH, mental health; PCS, physical component summary; PF, physical functioning; RE, role emotional; RP, role physical; VT, vitality; SF, social functioning

**SM3 Table 8**. Canadian norms for VR-12 health utility values, summary component scores, and domain scores, by age group – Saskatchewan, males only.

|  | All participants (n=106) | 18 to 29 (n=24) | 30 to 39 (n=15) | 40 to 49 (n=19) | 50 to 59 (n=23) | 60 to 69 (n=11) | 70 to 79 (n=12) | 80 and over (n=2) |
| --- | --- | --- | --- | --- | --- | --- | --- | --- |
| *VR-12 health utility values* |  |  |  |  |  |  |  |  |
| Mean | 0.692 | 0.662 | 0.685 | 0.657 | 0.729 | 0.661 | 0.798 | - |
| Standard deviation | 0.221 | 0.249 | 0.140 | 0.251 | 0.233 | 0.253 | 0.116 | - |
| Median | 0.727 | 0.716 | 0.716 | 0.746 | 0.810 | 0.594 | 0.852 | - |
| 25th percentile | 0.615 | 0.635 | 0.553 | 0.644 | 0.706 | 0.580 | 0.784 | - |
| 75th percentile | 0.852 | 0.765 | 0.817 | 0.818 | 0.913 | 0.818 | 0.852 | - |
| Minimum | -0.412 | 0.162 | 0.347 | 0.007 | -0.412 | 0.152 | 0.355 | - |
| Maximum | 1.000 | 0.960 | 0.879 | 1.000 | 0.913 | 0.913 | 0.913 | - |
| *VR-12 summary component score* |  |  |  |  |  |  |  |  |
| PCS   Mean | 49.250 | 56.314 | 48.795 | 47.206 | 49.279 | 39.767 | 50.894 | - |
| Standard deviation | 11.140 | 5.140 | 5.390 | 11.430 | 8.332 | 17.994 | 8.857 | - |
| Median | 54.390 | 56.468 | 49.030 | 51.436 | 54.759 | 38.304 | 54.796 | - |
| 25th percentile | 47.614 | 54.469 | 44.439 | 43.364 | 42.503 | 24.917 | 53.179 | - |
| 75th percentile | 56.824 | 58.888 | 55.607 | 56.571 | 56.824 | 54.229 | 56.216 | - |
| Minimum | 20.271 | 29.550 | 28.795 | 20.271 | 22.941 | 24.880 | 24.500 | - |
| Maximum | 61.057 | 61.057 | 56.071 | 60.325 | 57.258 | 56.824 | 59.100 | - |
| MCS  Mean | 50.607 | 43.589 | 47.862 | 50.006 | 52.342 | 58.251 | 55.523 | - |
| Standard deviation | 10.934 | 13.105 | 8.848 | 7.342 | 7.798 | 11.118 | 4.677 | - |
| Median | 53.502 | 44.370 | 48.217 | 52.646 | 54.390 | 59.440 | 55.110 | - |
| 25th percentile | 44.370 | 40.760 | 44.420 | 43.445 | 51.270 | 57.114 | 55.110 | - |
| 75th percentile | 58.396 | 49.191 | 57.164 | 55.070 | 59.588 | 67.226 | 58.376 | - |
| Minimum | 17.914 | 17.914 | 27.721 | 33.114 | 22.636 | 28.494 | 35.408 | - |
| Maximum | 67.226 | 60.789 | 64.854 | 62.711 | 63.883 | 67.226 | 64.049 | - |
| *VR-12 domain scores* |  |  |  |  |  |  |  |  |
| PF     Mean | 74.380 | 89.907 | 76.163 | 68.008 | 77.529 | 47.649 | 83.290 | - |
| Standard deviation | 28.038 | 11.047 | 18.294 | 28.719 | 20.877 | 47.156 | 18.462 | - |
| Median | 93.485 | 93.485 | 93.485 | 80.375 | 93.485 | 58.290 | 93.485 | - |
| 25th percentile | 58.290 | 93.485 | 58.290 | 58.290 | 58.290 | 7.765 | 93.485 | - |
| 75th percentile | 93.485 | 93.485 | 93.485 | 93.485 | 93.485 | 80.375 | 93.485 | - |
| Minimum | 7.765 | 29.460 | 7.765 | 7.765 | 7.765 | 7.765 | 29.460 | - |
| Maximum | 93.485 | 93.485 | 93.485 | 93.485 | 93.485 | 93.485 | 93.485 | - |
| RP     Mean | 74.752 | 83.294 | 69.126 | 77.988 | 78.980 | 51.564 | 84.350 | - |
| Standard deviation | 37.891 | 29.500 | 31.107 | 33.852 | 29.161 | 67.627 | 28.463 | - |
| Median | 100.690 | 100.690 | 79.405 | 100.690 | 100.690 | 79.405 | 100.690 | - |
| 25th percentile | 54.725 | 54.725 | 44.720 | 79.405 | 79.405 | -3.670 | 79.405 | - |
| 75th percentile | 100.690 | 100.690 | 100.690 | 100.690 | 100.690 | 100.690 | 100.690 | - |
| Minimum | -3.670 | 5.020 | 0.465 | -3.670 | 0.465 | -3.670 | -3.670 | - |
| Maximum | 100.690 | 100.690 | 100.690 | 100.690 | 100.690 | 100.690 | 100.690 | - |
| BP     Mean | 73.258 | 74.608 | 63.933 | 69.159 | 69.628 | 76.312 | 81.123 | - |
| Standard deviation | 23.523 | 24.040 | 19.109 | 21.912 | 21.565 | 32.351 | 20.443 | - |
| Median | 95.200 | 56.250 | 56.250 | 56.250 | 56.250 | 95.200 | 95.200 | - |
| 25th percentile | 56.250 | 56.250 | 43.890 | 56.250 | 56.250 | 56.250 | 56.250 | - |
| 75th percentile | 95.200 | 95.200 | 95.200 | 95.200 | 95.200 | 95.200 | 95.200 | - |
| Minimum | 2.280 | 31.500 | 43.890 | 31.500 | 2.280 | 31.500 | 31.500 | - |
| Maximum | 95.200 | 95.200 | 95.200 | 95.200 | 95.200 | 95.200 | 95.200 | - |
| GH    Mean | 68.845 | 80.860 | 71.270 | 62.944 | 68.707 | 63.708 | 59.048 | - |
| Standard deviation | 22.187 | 11.074 | 15.344 | 28.187 | 14.288 | 31.541 | 22.587 | - |
| Median | 83.710 | 83.710 | 83.710 | 83.710 | 61.500 | 61.500 | 61.500 | - |
| 25th percentile | 61.500 | 83.710 | 61.500 | 37.500 | 61.500 | 37.500 | 37.500 | - |
| 75th percentile | 83.710 | 83.710 | 83.710 | 83.710 | 83.710 | 83.710 | 83.710 | - |
| Minimum | 0.580 | 61.500 | 0.580 | 0.580 | 0.580 | 37.500 | 0.580 | - |
| Maximum | 101.840 | 101.840 | 83.710 | 101.840 | 83.710 | 101.840 | 101.840 | - |
| VT     Mean | 53.248 | 50.047 | 47.560 | 53.200 | 52.821 | 56.576 | 69.897 | - |
| Standard deviation | 22.711 | 27.298 | 16.561 | 18.622 | 17.435 | 35.305 | 18.427 | - |
| Median | 54.550 | 54.550 | 54.550 | 45.900 | 54.550 | 54.550 | 79.250 | - |
| 25th percentile | 45.900 | 45.900 | 45.900 | 31.930 | 45.900 | 45.900 | 54.550 | - |
| 75th percentile | 79.250 | 54.550 | 54.550 | 79.250 | 79.250 | 79.250 | 79.250 | - |
| Minimum | 1.800 | 1.800 | 1.800 | 31.930 | 1.800 | 1.800 | 1.800 | - |
| Maximum | 95.340 | 95.340 | 79.250 | 95.340 | 79.250 | 79.250 | 95.340 | - |
| SF      Mean | 75.828 | 58.047 | 73.689 | 77.256 | 86.422 | 88.523 | 91.243 | - |
| Standard deviation | 26.922 | 29.416 | 21.767 | 22.161 | 20.709 | 26.080 | 18.578 | - |
| Median | 99.320 | 48.870 | 66.870 | 99.320 | 99.320 | 99.320 | 99.320 | - |
| 25th percentile | 48.870 | 48.870 | 66.870 | 48.870 | 99.320 | 66.870 | 99.320 | - |
| 75th percentile | 99.320 | 66.870 | 99.320 | 99.320 | 99.320 | 99.320 | 99.320 | - |
| Minimum | 2.780 | 24.910 | 24.910 | 24.910 | 2.780 | 24.910 | 2.780 | - |
| Maximum | 99.320 | 99.320 | 99.320 | 99.320 | 99.320 | 99.320 | 99.320 | - |
| MH   Mean | 69.320 | 57.383 | 65.652 | 66.790 | 73.183 | 77.470 | 80.624 | - |
| Standard deviation | 23.457 | 28.415 | 19.833 | 20.557 | 18.357 | 22.458 | 11.352 | - |
| Median | 74.865 | 51.325 | 65.505 | 69.490 | 74.865 | 83.670 | 83.670 | - |
| 25th percentile | 51.325 | 51.325 | 55.620 | 55.620 | 60.685 | 74.865 | 74.865 | - |
| 75th percentile | 89.615 | 74.865 | 92.650 | 83.670 | 92.650 | 83.670 | 92.650 | - |
| Minimum | 6.005 | 6.005 | 16.745 | 21.060 | 6.005 | 26.385 | 31.800 | - |
| Maximum | 98.595 | 92.650 | 98.595 | 98.595 | 92.650 | 92.650 | 98.595 | - |
| RE     Mean | 79.289 | 68.862 | 63.125 | 74.278 | 82.801 | 96.145 | 90.955 | - |
| Standard deviation | 35.777 | 44.400 | 39.016 | 28.726 | 28.726 | 33.849 | 23.030 | - |
| Median | 104.125 | 65.090 | 54.690 | 81.810 | 104.125 | 104.125 | 104.125 | - |
| 25th percentile | 54.690 | 54.690 | 29.335 | 54.690 | 54.690 | 104.125 | 104.125 | - |
| 75th percentile | 104.125 | 104.125 | 104.125 | 104.125 | 104.125 | 104.125 | 104.125 | - |
| Minimum | -33.415 | -13.835 | -13.835 | 2.250 | -20.070 | -33.415 | 13.250 | - |
| Maximum | 104.125 | 104.125 | 104.125 | 104.125 | 104.125 | 104.125 | 104.125 | - |

BP, bodily pain; GH, general health; MCS, mental component summary; MH, mental health; PCS, physical component summary; PF, physical functioning; RE, role emotional; RP, role physical; VT, vitality; SF, social functioning

**SM3 Table 9**. Canadian norms for VR-12 health utility values, summary component scores, and domain scores, by age group – Saskatchewan, females only.

|  | All participants (n=97) | 18 to 29 (n=17) | 30 to 39 (n=19) | 40 to 49 (n=20) | 50 to 59 (n=16) | 60 to 69 (n=10) | 70 to 79 (n=14) | 80 and over (n=1) |
| --- | --- | --- | --- | --- | --- | --- | --- | --- |
| *VR-12 health utility values* |  |  |  |  |  |  |  |  |
| Mean | 0.707 | 0.581 | 0.656 | 0.733 | 0.641 | 0.802 | 0.774 | - |
| Standard deviation | 0.196 | 0.232 | 0.221 | 0.192 | 0.142 | 0.110 | 0.147 | - |
| Median | 0.746 | 0.625 | 0.726 | 0.818 | 0.674 | 0.839 | 0.722 | - |
| 25th percentile | 0.625 | 0.554 | 0.477 | 0.696 | 0.596 | 0.697 | 0.644 | - |
| 75th percentile | 0.852 | 0.746 | 0.861 | 0.852 | 0.758 | 0.839 | 0.913 | - |
| Minimum | -0.103 | -0.103 | -0.033 | 0.253 | 0.250 | 0.619 | 0.438 | - |
| Maximum | 0.960 | 0.852 | 0.913 | 0.960 | 0.879 | 0.913 | 0.960 | - |
| *VR-12 summary component score* |  |  |  |  |  |  |  |  |
| PCS   Mean | 50.794 | 55.717 | 50.942 | 53.736 | 42.985 | 49.112 | 47.816 | - |
| Standard deviation | 8.518 | 7.761 | 8.270 | 7.444 | 8.509 | 8.006 | 8.102 | - |
| Median | 53.024 | 58.456 | 51.540 | 56.691 | 44.691 | 50.828 | 51.038 | - |
| 25th percentile | 45.195 | 49.090 | 47.114 | 53.426 | 37.553 | 43.480 | 42.892 | - |
| 75th percentile | 56.824 | 61.047 | 56.824 | 58.625 | 54.229 | 50.828 | 55.446 | - |
| Minimum | 9.897 | 40.270 | 9.897 | 33.582 | 17.138 | 31.770 | 26.518 | - |
| Maximum | 70.390 | 70.390 | 59.730 | 61.809 | 56.713 | 58.183 | 56.824 | - |
| MCS  Mean | 49.808 | 39.414 | 46.833 | 48.757 | 51.232 | 57.802 | 54.265 | - |
| Standard deviation | 10.564 | 11.989 | 11.904 | 9.236 | 5.402 | 4.679 | 7.253 | - |
| Median | 52.752 | 40.942 | 48.600 | 52.634 | 50.467 | 58.928 | 58.376 | - |
| 25th percentile | 44.346 | 25.111 | 36.665 | 42.663 | 45.478 | 57.716 | 46.028 | - |
| 75th percentile | 58.928 | 49.752 | 59.588 | 54.556 | 57.417 | 58.928 | 60.821 | - |
| Minimum | 12.358 | 12.358 | 23.912 | 30.364 | 38.436 | 49.366 | 42.059 | - |
| Maximum | 65.243 | 56.867 | 61.126 | 60.789 | 65.243 | 64.854 | 60.987 | - |
| *VR-12 domain scores* |  |  |  |  |  |  |  |  |
| PF     Mean | 81.353 | 87.841 | 81.854 | 85.552 | 64.177 | 81.188 | 78.127 | - |
| Standard deviation | 17.593 | 9.158 | 17.212 | 16.022 | 20.824 | 20.303 | 17.984 | - |
| Median | 93.485 | 93.485 | 93.485 | 93.485 | 58.290 | 93.485 | 80.375 | - |
| 25th percentile | 71.400 | 80.375 | 71.400 | 80.375 | 58.290 | 58.290 | 71.400 | - |
| 75th percentile | 93.485 | 93.485 | 93.485 | 93.485 | 93.485 | 93.485 | 93.485 | - |
| Minimum | 7.765 | 58.290 | 7.765 | 7.765 | 7.765 | 49.705 | 7.765 | - |
| Maximum | 93.485 | 93.485 | 93.485 | 93.485 | 93.485 | 93.485 | 93.485 | - |
| RP     Mean | 78.833 | 73.370 | 78.256 | 83.460 | 69.734 | 86.658 | 72.380 | - |
| Standard deviation | 31.862 | 28.838 | 34.793 | 34.309 | 25.521 | 38.443 | 31.868 | - |
| Median | 100.690 | 89.670 | 90.425 | 100.690 | 79.405 | 100.690 | 90.425 | - |
| 25th percentile | 79.405 | 49.970 | 79.405 | 89.670 | 54.725 | 89.670 | 44.720 | - |
| 75th percentile | 100.690 | 100.690 | 100.690 | 100.690 | 100.690 | 100.690 | 100.690 | - |
| Minimum | -3.670 | 9.775 | -3.670 | -3.670 | -3.670 | 5.020 | 0.490 | - |
| Maximum | 100.690 | 100.690 | 100.690 | 100.690 | 100.690 | 100.690 | 100.690 | - |
| BP     Mean | 70.074 | 78.635 | 62.662 | 77.998 | 56.384 | 69.981 | 66.785 | - |
| Standard deviation | 24.414 | 21.472 | 26.122 | 27.562 | 17.525 | 23.959 | 23.958 | - |
| Median | 56.250 | 95.200 | 56.250 | 95.200 | 56.250 | 56.250 | 56.250 | - |
| 25th percentile | 56.250 | 56.250 | 43.890 | 56.250 | 43.890 | 56.250 | 43.890 | - |
| 75th percentile | 95.200 | 95.200 | 95.200 | 95.200 | 56.250 | 95.200 | 95.200 | - |
| Minimum | 2.280 | 31.500 | 2.280 | 2.280 | 2.280 | 56.250 | 31.500 | - |
| Maximum | 95.200 | 95.200 | 95.200 | 95.200 | 95.200 | 95.200 | 95.200 | - |
| GH    Mean | 72.012 | 74.501 | 76.536 | 78.229 | 58.897 | 66.111 | 67.898 | - |
| Standard deviation | 17.219 | 15.526 | 16.368 | 21.472 | 14.741 | 14.343 | 13.301 | - |
| Median | 83.710 | 83.710 | 83.710 | 83.710 | 61.500 | 61.500 | 61.500 | - |
| 25th percentile | 61.500 | 61.500 | 61.500 | 61.500 | 61.500 | 61.500 | 61.500 | - |
| 75th percentile | 83.710 | 83.710 | 83.710 | 83.710 | 61.500 | 61.500 | 83.710 | - |
| Minimum | 0.580 | 0.580 | 0.580 | 0.580 | 0.580 | 37.500 | 37.500 | - |
| Maximum | 101.840 | 101.840 | 101.840 | 101.840 | 83.710 | 83.710 | 83.710 | - |
| VT     Mean | 52.254 | 38.848 | 52.045 | 57.772 | 41.844 | 56.414 | 55.986 | - |
| Standard deviation | 22.495 | 19.387 | 22.085 | 22.808 | 16.193 | 21.918 | 28.108 | - |
| Median | 54.550 | 45.900 | 45.900 | 54.550 | 45.900 | 54.550 | 54.550 | - |
| 25th percentile | 45.900 | 31.930 | 45.900 | 45.900 | 31.930 | 54.550 | 31.930 | - |
| 75th percentile | 79.250 | 45.900 | 79.250 | 79.250 | 54.550 | 54.550 | 79.250 | - |
| Minimum | 1.800 | 1.800 | 1.800 | 1.800 | 1.800 | 1.800 | 1.800 | - |
| Maximum | 95.340 | 79.250 | 79.250 | 95.340 | 79.250 | 79.250 | 95.340 | - |
| SF      Mean | 76.632 | 65.917 | 63.708 | 77.260 | 72.721 | 90.490 | 88.325 | - |
| Standard deviation | 25.626 | 29.682 | 28.570 | 26.806 | 13.926 | 21.162 | 16.361 | - |
| Median | 99.320 | 66.870 | 66.870 | 99.320 | 66.870 | 99.320 | 99.320 | - |
| 25th percentile | 66.870 | 24.910 | 48.870 | 48.870 | 66.870 | 99.320 | 66.870 | - |
| 75th percentile | 99.320 | 99.320 | 99.320 | 99.320 | 99.320 | 99.320 | 99.320 | - |
| Minimum | 2.780 | 2.780 | 2.780 | 24.910 | 24.910 | 48.870 | 48.870 | - |
| Maximum | 99.320 | 99.320 | 99.320 | 99.320 | 99.320 | 99.320 | 99.320 | - |
| MH   Mean | 71.884 | 52.192 | 63.858 | 72.533 | 71.775 | 87.811 | 80.546 | - |
| Standard deviation | 21.836 | 23.024 | 24.372 | 19.403 | 14.880 | 10.155 | 16.806 | - |
| Median | 83.670 | 51.325 | 59.850 | 83.670 | 74.865 | 92.650 | 83.670 | - |
| 25th percentile | 59.850 | 31.800 | 51.045 | 60.685 | 51.325 | 83.670 | 74.865 | - |
| 75th percentile | 92.650 | 74.865 | 92.650 | 83.670 | 92.650 | 92.650 | 92.650 | - |
| Minimum | -1.030 | -1.030 | 21.060 | 6.005 | 41.440 | 69.490 | 31.800 | - |
| Maximum | 98.595 | 83.670 | 92.650 | 92.650 | 92.650 | 98.595 | 92.650 | - |
| RE     Mean | 75.093 | 43.206 | 75.020 | 69.183 | 76.570 | 100.410 | 80.818 | - |
| Standard deviation | 37.807 | 37.629 | 40.050 | 47.315 | 21.073 | 10.703 | 28.694 | - |
| Median | 104.125 | 42.775 | 104.125 | 104.125 | 81.810 | 104.125 | 104.125 | - |
| 25th percentile | 54.690 | 2.250 | 41.250 | 29.335 | 54.690 | 104.125 | 41.250 | - |
| 75th percentile | 104.125 | 81.810 | 104.125 | 104.125 | 104.125 | 104.125 | 104.125 | - |
| Minimum | -33.415 | -13.835 | 2.250 | -33.415 | -3.985 | 81.810 | 29.335 | - |
| Maximum | 104.125 | 104.125 | 104.125 | 104.125 | 104.125 | 104.125 | 104.125 | - |

BP, bodily pain; GH, general health; MCS, mental component summary; MH, mental health; PCS, physical component summary; PF, physical functioning; RE, role emotional; RP, role physical; VT, vitality; SF, social functioning

**SM3 Table 10**. Canadian norms for VR-12 health utility values, summary component scores, and domain scores, by age group – Manitoba.

|  | All participants (n=280) | 18 to 29 (n=42) | 30 to 39 (n=48) | 40 to 49 (n=67) | 50 to 59 (n=63) | 60 to 69 (n=26) | 70 to 79 (n=30) | 80 and over (n=4) |
| --- | --- | --- | --- | --- | --- | --- | --- | --- |
| *VR-12 health utility values* |  |  |  |  |  |  |  |  |
| Mean | 0.637 | 0.550 | 0.587 | 0.654 | 0.644 | 0.677 | 0.666 | - |
| Standard deviation | 0.233 | 0.237 | 0.240 | 0.171 | 0.279 | 0.172 | 0.227 | - |
| Median | 0.697 | 0.587 | 0.644 | 0.674 | 0.716 | 0.697 | 0.756 | - |
| 25th percentile | 0.508 | 0.353 | 0.468 | 0.583 | 0.569 | 0.547 | 0.696 | - |
| 75th percentile | 0.818 | 0.746 | 0.746 | 0.808 | 0.852 | 0.802 | 0.818 | - |
| Minimum | -0.590 | 0.174 | -0.159 | -0.590 | -0.383 | 0.376 | -0.334 | - |
| Maximum | 1.000 | 1.000 | 1.000 | 1.000 | 0.913 | 0.919 | 1.000 | - |
| *VR-12 summary component score* |  |  |  |  |  |  |  |  |
| PCS   Mean | 48.782 | 54.781 | 48.257 | 50.708 | 47.041 | 47.700 | 43.191 | - |
| Standard deviation | 9.636 | 8.147 | 10.133 | 7.772 | 9.748 | 11.065 | 8.731 | - |
| Median | 49.570 | 56.824 | 46.215 | 53.626 | 49.815 | 47.181 | 43.176 | - |
| 25th percentile | 41.173 | 44.642 | 44.958 | 45.294 | 37.897 | 43.375 | 39.063 | - |
| 75th percentile | 57.299 | 61.409 | 56.927 | 58.086 | 56.824 | 54.885 | 54.642 | - |
| Minimum | 11.078 | 38.217 | 19.963 | 17.643 | 11.078 | 18.784 | 15.591 | - |
| Maximum | 68.411 | 64.968 | 66.466 | 63.279 | 60.689 | 68.411 | 58.016 | - |
| MCS  Mean | 47.013 | 39.150 | 45.536 | 45.805 | 49.346 | 48.156 | 52.972 | - |
| Standard deviation | 10.681 | 11.136 | 9.067 | 8.422 | 10.631 | 13.335 | 6.207 | - |
| Median | 48.170 | 35.691 | 44.385 | 48.170 | 52.491 | 53.482 | 52.211 | - |
| 25th percentile | 39.746 | 31.719 | 40.953 | 41.486 | 46.315 | 39.219 | 51.223 | - |
| 75th percentile | 57.000 | 48.333 | 50.499 | 52.057 | 58.376 | 57.381 | 59.576 | - |
| Minimum | 10.612 | 22.543 | 18.964 | 23.617 | 10.612 | 19.089 | 27.665 | - |
| Maximum | 65.145 | 62.699 | 62.699 | 62.751 | 64.211 | 60.856 | 65.145 | - |
| *VR-12 domain scores* |  |  |  |  |  |  |  |  |
| PF     Mean | 71.678 | 81.002 | 65.714 | 74.552 | 71.162 | 68.290 | 60.444 | - |
| Standard deviation | 25.406 | 18.824 | 33.264 | 19.493 | 24.972 | 32.018 | 22.371 | - |
| Median | 80.375 | 93.485 | 80.375 | 80.375 | 80.375 | 80.375 | 58.290 | - |
| 25th percentile | 58.290 | 58.290 | 36.595 | 58.290 | 58.290 | 29.460 | 58.290 | - |
| 75th percentile | 93.485 | 93.485 | 93.485 | 93.485 | 93.485 | 93.485 | 80.375 | - |
| Minimum | 7.765 | 29.460 | 7.765 | 7.765 | 7.765 | 7.765 | 7.765 | - |
| Maximum | 93.485 | 93.485 | 93.485 | 93.485 | 93.485 | 93.485 | 93.485 | - |
| RP     Mean | 72.507 | 74.623 | 66.087 | 81.234 | 73.621 | 74.530 | 70.054 | - |
| Standard deviation | 33.720 | 34.849 | 41.458 | 27.839 | 32.707 | 32.809 | 27.212 | - |
| Median | 89.670 | 100.690 | 89.670 | 100.690 | 89.670 | 79.405 | 79.405 | - |
| 25th percentile | 44.720 | 54.725 | 9.775 | 79.405 | 51.210 | 54.725 | 44.720 | - |
| 75th percentile | 100.690 | 100.690 | 100.690 | 100.690 | 100.690 | 100.690 | 100.690 | - |
| Minimum | -3.670 | 9.775 | -3.670 | -3.670 | -3.670 | 0.465 | -3.670 | - |
| Maximum | 100.690 | 100.690 | 100.690 | 100.690 | 100.690 | 100.690 | 100.690 | - |
| BP     Mean | 67.014 | 74.000 | 61.657 | 71.287 | 64.482 | 63.862 | 70.780 | - |
| Standard deviation | 22.783 | 20.442 | 25.827 | 19.727 | 22.923 | 25.519 | 20.807 | - |
| Median | 56.250 | 56.250 | 56.250 | 56.250 | 56.250 | 56.250 | 95.200 | - |
| 25th percentile | 56.250 | 56.250 | 43.890 | 56.250 | 43.890 | 43.890 | 56.250 | - |
| 75th percentile | 95.200 | 95.200 | 95.200 | 95.200 | 95.200 | 95.200 | 95.200 | - |
| Minimum | 2.280 | 43.890 | 2.280 | 2.280 | 2.280 | 31.500 | 2.280 | - |
| Maximum | 95.200 | 95.200 | 95.200 | 95.200 | 95.200 | 95.200 | 95.200 | - |
| GH    Mean | 67.737 | 66.930 | 78.992 | 66.449 | 61.642 | 68.920 | 54.167 | - |
| Standard deviation | 22.934 | 28.636 | 19.089 | 16.951 | 23.633 | 16.689 | 26.818 | - |
| Median | 61.500 | 83.710 | 83.710 | 61.500 | 61.500 | 61.500 | 61.500 | - |
| 25th percentile | 61.500 | 61.500 | 61.500 | 61.500 | 37.500 | 61.500 | 37.500 | - |
| 75th percentile | 83.710 | 83.710 | 83.710 | 83.710 | 83.710 | 83.710 | 83.710 | - |
| Minimum | 0.580 | 0.580 | 0.580 | 0.580 | 0.580 | 37.500 | 0.580 | - |
| Maximum | 101.840 | 101.840 | 101.840 | 101.840 | 101.840 | 83.710 | 101.840 | - |
| VT     Mean | 50.236 | 57.402 | 55.092 | 47.218 | 48.855 | 42.347 | 47.748 | - |
| Standard deviation | 22.857 | 30.453 | 21.850 | 15.365 | 23.221 | 26.926 | 19.303 | - |
| Median | 45.900 | 54.550 | 54.550 | 45.900 | 45.900 | 45.900 | 45.900 | - |
| 25th percentile | 31.930 | 31.930 | 45.900 | 31.930 | 31.930 | 31.930 | 31.930 | - |
| 75th percentile | 54.550 | 95.340 | 54.550 | 54.550 | 54.550 | 54.550 | 79.250 | - |
| Minimum | 1.800 | 1.800 | 1.800 | 1.800 | 1.800 | 1.800 | 1.800 | - |
| Maximum | 95.340 | 95.340 | 95.340 | 95.340 | 95.340 | 79.250 | 95.340 | - |
| SF      Mean | 66.008 | 48.911 | 49.955 | 66.993 | 74.458 | 75.242 | 80.006 | - |
| Standard deviation | 32.461 | 33.405 | 38.848 | 26.399 | 27.760 | 25.907 | 24.255 | - |
| Median | 66.870 | 48.870 | 48.870 | 66.870 | 99.320 | 66.870 | 99.320 | - |
| 25th percentile | 48.870 | 2.780 | 2.780 | 48.870 | 48.870 | 66.870 | 66.870 | - |
| 75th percentile | 99.320 | 66.870 | 66.870 | 99.320 | 99.320 | 99.320 | 99.320 | - |
| Minimum | 2.780 | 2.780 | 2.780 | 2.780 | 2.780 | 24.910 | 2.780 | - |
| Maximum | 99.320 | 99.320 | 99.320 | 99.320 | 99.320 | 99.320 | 99.320 | - |
| MH   Mean | 65.277 | 46.594 | 68.816 | 60.102 | 68.663 | 67.456 | 73.427 | - |
| Standard deviation | 25.014 | 26.722 | 24.822 | 19.806 | 25.229 | 25.328 | 15.585 | - |
| Median | 74.485 | 41.685 | 74.865 | 65.505 | 83.670 | 74.865 | 74.865 | - |
| 25th percentile | 51.045 | 21.060 | 51.325 | 41.685 | 51.325 | 51.325 | 59.850 | - |
| 75th percentile | 83.845 | 69.490 | 92.650 | 74.865 | 92.650 | 83.670 | 92.650 | - |
| Minimum | -1.030 | 14.025 | 6.005 | -1.030 | -1.030 | 21.060 | 6.005 | - |
| Maximum | 98.595 | 98.595 | 98.595 | 98.595 | 98.595 | 98.595 | 98.595 | - |
| RE     Mean | 65.396 | 58.504 | 48.768 | 69.829 | 72.314 | 64.683 | 84.009 | - |
| Standard deviation | 38.248 | 41.520 | 42.555 | 30.365 | 35.691 | 48.562 | 27.087 | - |
| Median | 77.005 | 81.810 | 41.250 | 81.810 | 81.810 | 81.810 | 104.125 | - |
| 25th percentile | 29.335 | 2.250 | 14.165 | 42.775 | 54.690 | 14.165 | 77.005 | - |
| 75th percentile | 104.125 | 104.125 | 81.810 | 104.125 | 104.125 | 104.125 | 104.125 | - |
| Minimum | -33.415 | -13.835 | -33.415 | -33.415 | -33.415 | -13.835 | -33.415 | - |
| Maximum | 104.125 | 104.125 | 104.125 | 104.125 | 104.125 | 104.125 | 104.125 | - |

BP, bodily pain; GH, general health; MCS, mental component summary; MH, mental health; PCS, physical component summary; PF, physical functioning; RE, role emotional; RP, role physical; VT, vitality; SF, social functioning

**SM3 Table 11**. Canadian norms for VR-12 health utility values, summary component scores, and domain scores, by age group – Manitoba, males only.

|  | All participants (n=146) | 18 to 29 (n=22) | 30 to 39 (n=27) | 40 to 49 (n=41) | 50 to 59 (n=28) | 60 to 69 (n=9) | 70 to 79 (n=17) | 80 and over (n=2) |
| --- | --- | --- | --- | --- | --- | --- | --- | --- |
| *VR-12 health utility values* |  |  |  |  |  |  |  |  |
| Mean | 0.681 | 0.593 | 0.664 | 0.687 | 0.611 | 0.825 | 0.715 | - |
| Standard deviation | 0.224 | 0.223 | 0.235 | 0.161 | 0.314 | 0.067 | 0.122 | - |
| Median | 0.756 | 0.587 | 0.746 | 0.674 | 0.746 | 0.829 | 0.756 | - |
| 25th percentile | 0.587 | 0.353 | 0.544 | 0.644 | 0.569 | 0.799 | 0.706 | - |
| 75th percentile | 0.829 | 0.832 | 0.818 | 0.818 | 0.879 | 0.873 | 0.808 | - |
| Minimum | -0.590 | 0.353 | 0.105 | -0.590 | -0.123 | 0.713 | 0.268 | - |
| Maximum | 1.000 | 1.000 | 1.000 | 1.000 | 0.913 | 0.919 | 0.952 | - |
| *VR-12 summary component score* |  |  |  |  |  |  |  |  |
| PCS   Mean | 49.951 | 58.206 | 52.016 | 50.652 | 46.448 | 51.654 | 42.578 | - |
| Standard deviation | 9.456 | 7.393 | 8.547 | 7.363 | 11.070 | 5.233 | 6.796 | - |
| Median | 52.911 | 60.562 | 55.351 | 52.219 | 49.815 | 53.423 | 40.689 | - |
| 25th percentile | 40.689 | 55.542 | 45.982 | 45.062 | 35.681 | 47.181 | 40.689 | - |
| 75th percentile | 57.763 | 64.968 | 57.763 | 58.161 | 56.824 | 55.354 | 45.198 | - |
| Minimum | 11.078 | 38.217 | 19.963 | 21.960 | 11.078 | 41.890 | 20.962 | - |
| Maximum | 66.466 | 64.968 | 66.466 | 63.279 | 60.689 | 57.791 | 56.340 | - |
| MCS  Mean | 49.345 | 42.194 | 47.791 | 47.299 | 49.342 | 56.850 | 53.708 | - |
| Standard deviation | 10.139 | 11.532 | 9.759 | 6.321 | 12.453 | 5.119 | 5.018 | - |
| Median | 51.685 | 36.569 | 49.183 | 47.537 | 54.438 | 59.536 | 52.211 | - |
| 25th percentile | 42.532 | 32.550 | 39.746 | 42.260 | 47.037 | 54.611 | 52.211 | - |
| 75th percentile | 59.516 | 50.569 | 57.512 | 53.846 | 59.588 | 60.287 | 59.807 | - |
| Minimum | 10.612 | 22.543 | 18.964 | 23.617 | 10.612 | 45.728 | 41.514 | - |
| Maximum | 65.145 | 62.699 | 62.699 | 62.751 | 64.211 | 60.528 | 65.145 | - |
| *VR-12 domain scores* |  |  |  |  |  |  |  |  |
| PF     Mean | 77.342 | 87.755 | 78.385 | 74.532 | 72.422 | 85.245 | 60.898 | - |
| Standard deviation | 21.885 | 14.905 | 25.166 | 19.688 | 25.490 | 14.034 | 20.603 | - |
| Median | 93.485 | 93.485 | 93.485 | 93.485 | 80.375 | 93.485 | 58.290 | - |
| 25th percentile | 58.290 | 93.485 | 71.400 | 58.290 | 58.290 | 71.400 | 58.290 | - |
| 75th percentile | 93.485 | 93.485 | 93.485 | 93.485 | 93.485 | 93.485 | 80.375 | - |
| Minimum | 7.765 | 29.460 | 7.765 | 7.765 | 7.765 | 58.290 | 7.765 | - |
| Maximum | 93.485 | 93.485 | 93.485 | 93.485 | 93.485 | 93.485 | 93.485 | - |
| RP     Mean | 76.994 | 91.157 | 72.389 | 82.634 | 72.176 | 91.007 | 73.504 | - |
| Standard deviation | 30.667 | 21.224 | 33.817 | 28.040 | 33.859 | 24.033 | 20.943 | - |
| Median | 100.690 | 100.690 | 100.690 | 100.690 | 89.670 | 100.690 | 79.405 | - |
| 25th percentile | 49.970 | 100.690 | 45.835 | 79.405 | 54.725 | 79.405 | 79.405 | - |
| 75th percentile | 100.690 | 100.690 | 100.690 | 100.690 | 100.690 | 100.690 | 89.670 | - |
| Minimum | -3.670 | 9.775 | -3.670 | -3.645 | -3.670 | 5.245 | 5.245 | - |
| Maximum | 100.690 | 100.690 | 100.690 | 100.690 | 100.690 | 100.690 | 100.690 | - |
| BP     Mean | 72.125 | 80.244 | 73.241 | 72.187 | 66.864 | 77.164 | 73.071 | - |
| Standard deviation | 21.934 | 19.816 | 21.900 | 19.532 | 26.189 | 23.249 | 18.143 | - |
| Median | 56.250 | 95.200 | 95.200 | 56.250 | 56.250 | 95.200 | 56.250 | - |
| 25th percentile | 56.250 | 56.250 | 56.250 | 56.250 | 31.500 | 56.250 | 56.250 | - |
| 75th percentile | 95.200 | 95.200 | 95.200 | 95.200 | 95.200 | 95.200 | 95.200 | - |
| Minimum | 2.280 | 43.890 | 31.500 | 2.280 | 31.500 | 43.890 | 31.500 | - |
| Maximum | 95.200 | 95.200 | 95.200 | 95.200 | 95.200 | 95.200 | 95.200 | - |
| GH    Mean | 68.162 | 79.307 | 77.317 | 67.173 | 60.738 | 73.301 | 43.756 | - |
| Standard deviation | 23.987 | 14.609 | 22.191 | 16.687 | 29.633 | 18.123 | 30.088 | - |
| Median | 61.500 | 83.710 | 83.710 | 61.500 | 83.710 | 83.710 | 61.500 | - |
| 25th percentile | 61.500 | 61.500 | 61.500 | 61.500 | 37.500 | 61.500 | 0.580 | - |
| 75th percentile | 83.710 | 83.710 | 101.840 | 83.710 | 83.710 | 83.710 | 83.710 | - |
| Minimum | 0.580 | 37.500 | 0.580 | 0.580 | 0.580 | 37.500 | 0.580 | - |
| Maximum | 101.840 | 101.840 | 101.840 | 101.840 | 101.840 | 83.710 | 101.840 | - |
| VT     Mean | 55.806 | 76.253 | 62.266 | 49.051 | 46.865 | 61.222 | 42.897 | - |
| Standard deviation | 21.300 | 22.871 | 20.961 | 16.998 | 18.633 | 17.875 | 14.622 | - |
| Median | 54.550 | 79.250 | 54.550 | 45.900 | 45.900 | 54.550 | 31.930 | - |
| 25th percentile | 45.900 | 54.550 | 54.550 | 31.930 | 45.900 | 54.550 | 31.930 | - |
| 75th percentile | 79.250 | 95.340 | 79.250 | 54.550 | 54.550 | 79.250 | 54.550 | - |
| Minimum | 1.800 | 31.930 | 1.800 | 1.800 | 1.800 | 31.930 | 1.800 | - |
| Maximum | 95.340 | 95.340 | 95.340 | 95.340 | 79.250 | 79.250 | 79.250 | - |
| SF      Mean | 71.271 | 45.552 | 67.482 | 69.963 | 70.991 | 90.277 | 84.758 | - |
| Standard deviation | 31.371 | 41.616 | 31.015 | 19.698 | 32.641 | 15.829 | 20.273 | - |
| Median | 99.320 | 48.870 | 66.870 | 66.870 | 99.320 | 99.320 | 99.320 | - |
| 25th percentile | 48.870 | 2.780 | 48.870 | 48.870 | 48.870 | 66.870 | 66.870 | - |
| 75th percentile | 99.320 | 99.320 | 99.320 | 99.320 | 99.320 | 99.320 | 99.320 | - |
| Minimum | 2.780 | 2.780 | 2.780 | 2.780 | 2.780 | 66.870 | 24.910 | - |
| Maximum | 99.320 | 99.320 | 99.320 | 99.320 | 99.320 | 99.320 | 99.320 | - |
| MH   Mean | 67.540 | 48.427 | 64.443 | 62.772 | 70.721 | 84.900 | 72.618 | - |
| Standard deviation | 24.726 | 31.405 | 23.273 | 16.723 | 26.484 | 15.583 | 11.538 | - |
| Median | 74.865 | 51.045 | 69.490 | 60.685 | 83.670 | 92.650 | 74.865 | - |
| 25th percentile | 51.045 | 14.025 | 51.045 | 49.110 | 51.325 | 83.670 | 59.850 | - |
| 75th percentile | 92.650 | 83.670 | 83.670 | 83.670 | 92.650 | 92.650 | 83.845 | - |
| Minimum | -1.030 | 14.025 | 6.005 | -1.030 | -1.030 | 51.325 | 49.110 | - |
| Maximum | 98.595 | 98.595 | 98.595 | 98.595 | 98.595 | 98.595 | 98.595 | - |
| RE     Mean | 78.602 | 81.283 | 68.335 | 76.397 | 73.227 | 97.928 | 92.473 | - |
| Standard deviation | 30.911 | 34.372 | 30.635 | 25.380 | 42.422 | 11.730 | 16.130 | - |
| Median | 104.125 | 104.125 | 77.005 | 81.810 | 104.125 | 104.125 | 104.125 | - |
| 25th percentile | 54.690 | 54.690 | 41.250 | 54.690 | 54.690 | 81.810 | 81.810 | - |
| 75th percentile | 104.125 | 104.125 | 104.125 | 104.125 | 104.125 | 104.125 | 104.125 | - |
| Minimum | -33.415 | -13.835 | 2.250 | 7.930 | -33.415 | 77.005 | 42.775 | - |
| Maximum | 104.125 | 104.125 | 104.125 | 104.125 | 104.125 | 104.125 | 104.125 | - |

BP, bodily pain; GH, general health; MCS, mental component summary; MH, mental health; PCS, physical component summary; PF, physical functioning; RE, role emotional; RP, role physical; VT, vitality; SF, social functioning

**SM3 Table 12**. Canadian norms for VR-12 health utility values, summary component scores, and domain scores, by age group – Manitoba, females only.

|  | All participants (n=132) | 18 to 29 (n=19) | 30 to 39 (n=20) | 40 to 49 (n=26) | 50 to 59 (n=35) | 60 to 69 (n=17) | 70 to 79 (n=13) | 80 and over (n=2) |
| --- | --- | --- | --- | --- | --- | --- | --- | --- |
| *VR-12 health utility values* |  |  |  |  |  |  |  |  |
| Mean | 0.610 | 0.618 | 0.531 | 0.605 | 0.668 | 0.614 | 0.585 | - |
| Standard deviation | 0.224 | 0.169 | 0.236 | 0.181 | 0.250 | 0.160 | 0.315 | - |
| Median | 0.682 | 0.730 | 0.468 | 0.682 | 0.696 | 0.645 | 0.800 | - |
| 25th percentile | 0.468 | 0.508 | 0.468 | 0.349 | 0.616 | 0.547 | 0.388 | - |
| 75th percentile | 0.765 | 0.746 | 0.706 | 0.779 | 0.852 | 0.727 | 0.852 | - |
| Minimum | -0.383 | 0.190 | -0.159 | 0.111 | -0.383 | 0.376 | -0.334 | - |
| Maximum | 1.000 | 0.913 | 0.799 | 0.832 | 0.913 | 0.829 | 1.000 | - |
| *VR-12 summary component score* |  |  |  |  |  |  |  |  |
| PCS   Mean | 47.841 | 53.194 | 45.836 | 50.793 | 47.488 | 46.018 | 44.213 | - |
| Standard deviation | 9.775 | 6.494 | 11.323 | 8.527 | 8.691 | 12.822 | 11.037 | - |
| Median | 49.354 | 55.816 | 45.263 | 53.878 | 49.570 | 43.375 | 49.804 | - |
| 25th percentile | 41.568 | 44.642 | 42.956 | 45.294 | 37.897 | 40.282 | 25.772 | - |
| 75th percentile | 56.174 | 58.245 | 51.288 | 57.582 | 54.838 | 50.415 | 57.763 | - |
| Minimum | 15.591 | 38.991 | 24.883 | 17.643 | 31.417 | 18.784 | 15.591 | - |
| Maximum | 68.411 | 64.910 | 61.145 | 62.328 | 58.877 | 68.411 | 58.016 | - |
| MCS  Mean | 45.644 | 39.378 | 44.591 | 43.563 | 49.349 | 44.458 | 51.747 | - |
| Standard deviation | 10.166 | 8.087 | 7.531 | 10.804 | 9.106 | 14.176 | 7.634 | - |
| Median | 46.315 | 40.482 | 44.385 | 48.170 | 52.491 | 41.104 | 56.619 | - |
| 25th percentile | 39.219 | 30.751 | 43.231 | 25.843 | 45.298 | 39.219 | 46.614 | - |
| 75th percentile | 53.858 | 47.362 | 47.507 | 51.954 | 57.512 | 56.890 | 59.576 | - |
| Minimum | 19.089 | 25.215 | 26.058 | 24.586 | 20.580 | 19.089 | 27.665 | - |
| Maximum | 62.699 | 59.608 | 58.937 | 60.652 | 59.608 | 60.856 | 62.699 | - |
| *VR-12 domain scores* |  |  |  |  |  |  |  |  |
| PF     Mean | 66.291 | 78.326 | 55.778 | 74.583 | 70.214 | 61.077 | 59.689 | - |
| Standard deviation | 27.910 | 18.966 | 38.951 | 19.568 | 24.886 | 35.508 | 25.357 | - |
| Median | 80.375 | 93.485 | 36.595 | 80.375 | 80.375 | 58.290 | 71.400 | - |
| 25th percentile | 36.595 | 80.375 | 36.595 | 71.400 | 51.545 | 29.460 | 7.765 | - |
| 75th percentile | 93.485 | 93.485 | 93.485 | 93.485 | 93.485 | 93.485 | 93.485 | - |
| Minimum | 7.765 | 29.460 | 7.765 | 29.460 | 7.765 | 7.765 | 7.765 | - |
| Maximum | 93.485 | 93.485 | 93.485 | 93.485 | 93.485 | 93.485 | 93.485 | - |
| RP     Mean | 71.317 | 71.415 | 65.529 | 79.133 | 74.708 | 67.522 | 64.306 | - |
| Standard deviation | 34.072 | 29.189 | 47.980 | 27.968 | 32.215 | 34.119 | 34.316 | - |
| Median | 89.670 | 79.405 | 89.670 | 89.670 | 89.670 | 79.405 | 100.690 | - |
| 25th percentile | 54.725 | 54.725 | 9.775 | 79.405 | 44.460 | 54.725 | 0.490 | - |
| 75th percentile | 100.690 | 100.690 | 90.425 | 100.690 | 100.690 | 89.670 | 100.690 | - |
| Minimum | -3.670 | 9.775 | -3.670 | -3.670 | 0.465 | 0.465 | -3.670 | - |
| Maximum | 100.690 | 100.690 | 100.690 | 100.690 | 100.690 | 100.690 | 100.690 | - |
| BP     Mean | 62.613 | 70.348 | 54.721 | 69.938 | 62.689 | 58.204 | 66.962 | - |
| Standard deviation | 22.742 | 18.967 | 27.156 | 20.363 | 20.169 | 24.454 | 24.399 | - |
| Median | 56.250 | 56.250 | 56.250 | 56.250 | 56.250 | 56.250 | 95.200 | - |
| 25th percentile | 43.890 | 43.890 | 43.890 | 56.250 | 56.250 | 43.890 | 31.500 | - |
| 75th percentile | 95.200 | 95.200 | 56.250 | 95.200 | 95.200 | 56.250 | 95.200 | - |
| Minimum | 2.280 | 43.890 | 2.280 | 31.500 | 2.280 | 31.500 | 2.280 | - |
| Maximum | 95.200 | 95.200 | 95.200 | 95.200 | 95.200 | 95.200 | 95.200 | - |
| GH    Mean | 69.895 | 70.974 | 81.377 | 65.363 | 62.322 | 67.056 | 71.515 | - |
| Standard deviation | 17.897 | 17.665 | 13.516 | 17.649 | 17.894 | 15.962 | 16.846 | - |
| Median | 61.500 | 83.710 | 83.710 | 61.500 | 61.500 | 61.500 | 61.500 | - |
| 25th percentile | 61.500 | 61.500 | 83.710 | 61.500 | 37.500 | 61.500 | 61.500 | - |
| 75th percentile | 83.710 | 83.710 | 83.710 | 83.710 | 83.710 | 83.710 | 83.710 | - |
| Minimum | 0.580 | 37.500 | 37.500 | 0.580 | 37.500 | 37.500 | 37.500 | - |
| Maximum | 101.840 | 101.840 | 101.840 | 101.840 | 101.840 | 83.710 | 101.840 | - |
| VT     Mean | 46.455 | 47.108 | 50.672 | 44.468 | 50.354 | 34.318 | 55.832 | - |
| Standard deviation | 22.021 | 15.383 | 21.994 | 12.289 | 26.511 | 25.934 | 23.700 | - |
| Median | 45.900 | 45.900 | 54.550 | 45.900 | 45.900 | 31.930 | 79.250 | - |
| 25th percentile | 31.930 | 31.930 | 45.900 | 31.930 | 31.930 | 1.800 | 31.930 | - |
| 75th percentile | 54.550 | 54.550 | 54.550 | 45.900 | 79.250 | 45.900 | 79.250 | - |
| Minimum | 1.800 | 1.800 | 1.800 | 1.800 | 1.800 | 1.800 | 1.800 | - |
| Maximum | 95.340 | 79.250 | 95.340 | 79.250 | 95.340 | 79.250 | 95.340 | - |
| SF      Mean | 61.592 | 54.347 | 37.683 | 62.541 | 77.067 | 68.847 | 72.089 | - |
| Standard deviation | 33.092 | 22.082 | 42.950 | 34.540 | 23.342 | 27.069 | 28.676 | - |
| Median | 66.870 | 66.870 | 48.870 | 66.870 | 66.870 | 66.870 | 99.320 | - |
| 25th percentile | 48.870 | 48.870 | 2.780 | 24.910 | 66.870 | 48.870 | 48.870 | - |
| 75th percentile | 99.320 | 66.870 | 66.870 | 99.320 | 99.320 | 99.320 | 99.320 | - |
| Minimum | 2.780 | 2.780 | 2.780 | 2.780 | 2.780 | 24.910 | 2.780 | - |
| Maximum | 99.320 | 99.320 | 99.320 | 99.320 | 99.320 | 99.320 | 99.320 | - |
| MH   Mean | 65.172 | 52.879 | 74.449 | 56.097 | 67.114 | 60.036 | 74.774 | - |
| Standard deviation | 23.772 | 16.677 | 23.775 | 23.815 | 24.468 | 24.985 | 20.200 | - |
| Median | 69.490 | 51.325 | 74.865 | 65.505 | 74.865 | 51.325 | 83.670 | - |
| 25th percentile | 51.325 | 30.945 | 65.505 | 40.565 | 51.325 | 51.325 | 69.490 | - |
| 75th percentile | 83.670 | 69.490 | 92.650 | 74.865 | 83.670 | 83.670 | 92.650 | - |
| Minimum | -1.030 | 14.025 | 21.060 | 6.005 | -1.030 | 21.060 | 6.005 | - |
| Maximum | 98.595 | 92.650 | 92.650 | 92.650 | 92.650 | 92.650 | 98.595 | - |
| RE     Mean | 54.979 | 44.365 | 35.501 | 59.980 | 71.627 | 50.542 | 69.907 | - |
| Standard deviation | 40.135 | 28.704 | 49.946 | 35.724 | 29.880 | 51.803 | 35.535 | - |
| Median | 54.690 | 29.335 | 14.165 | 54.690 | 68.370 | 29.335 | 104.125 | - |
| 25th percentile | 14.165 | 29.335 | 14.165 | 13.345 | 54.690 | 14.165 | 29.335 | - |
| 75th percentile | 104.125 | 81.810 | 77.005 | 104.125 | 104.125 | 104.125 | 104.125 | - |
| Minimum | -33.415 | -3.985 | -33.415 | -33.415 | -33.415 | -13.835 | -33.415 | - |
| Maximum | 104.125 | 104.125 | 104.125 | 104.125 | 104.125 | 104.125 | 104.125 | - |

BP, bodily pain; GH, general health; MCS, mental component summary; MH, mental health; PCS, physical component summary; PF, physical functioning; RE, role emotional; RP, role physical; VT, vitality; SF, social functioning

**SM3 Table 13**. Canadian norms for VR-12 health utility values, summary component scores, and domain scores, by age group – Ontario.

|  | All participants (n=2605) | 18 to 29 (n=516) | 30 to 39 (n=429) | 40 to 49 (n=496) | 50 to 59 (n=474) | 60 to 69 (n=275) | 70 to 79 (n=368) | 80 and over (n=47) |
| --- | --- | --- | --- | --- | --- | --- | --- | --- |
| *VR-12 health utility values* |  |  |  |  |  |  |  |  |
| Mean | 0.681 | 0.639 | 0.696 | 0.689 | 0.658 | 0.703 | 0.703 | 0.725 |
| Standard deviation | 0.227 | 0.225 | 0.215 | 0.205 | 0.252 | 0.250 | 0.195 | 0.315 |
| Median | 0.740 | 0.696 | 0.740 | 0.746 | 0.746 | 0.768 | 0.777 | 0.764 |
| 25th percentile | 0.606 | 0.525 | 0.630 | 0.627 | 0.610 | 0.644 | 0.644 | 0.697 |
| 75th percentile | 0.818 | 0.779 | 0.822 | 0.839 | 0.818 | 0.818 | 0.852 | 0.830 |
| Minimum | -0.513 | -0.263 | -0.419 | -0.242 | -0.513 | -0.393 | -0.134 | 0.231 |
| Maximum | 1.000 | 1.000 | 1.000 | 1.000 | 1.000 | 1.000 | 1.000 | 0.952 |
| *VR-12 summary component score* |  |  |  |  |  |  |  |  |
| PCS   Mean | 49.274 | 52.661 | 52.098 | 50.904 | 49.145 | 46.462 | 44.825 | 41.692 |
| Standard deviation | 10.299 | 7.919 | 8.162 | 8.612 | 10.355 | 12.591 | 9.852 | 21.283 |
| Median | 52.122 | 55.098 | 54.759 | 53.976 | 52.164 | 47.922 | 47.330 | 42.617 |
| 25th percentile | 43.317 | 47.754 | 48.056 | 47.691 | 44.230 | 40.441 | 36.419 | 35.803 |
| 75th percentile | 57.047 | 58.264 | 57.763 | 57.077 | 56.913 | 55.849 | 55.354 | 50.189 |
| Minimum | 4.409 | 19.499 | 10.489 | 10.494 | 5.715 | 12.719 | 10.844 | 4.409 |
| Maximum | 68.528 | 68.528 | 65.678 | 64.732 | 67.108 | 62.714 | 65.528 | 59.833 |
| MCS  Mean | 49.568 | 44.314 | 48.974 | 49.199 | 48.998 | 52.651 | 54.356 | 54.930 |
| Standard deviation | 10.290 | 11.095 | 9.703 | 9.176 | 10.078 | 10.195 | 7.054 | 11.713 |
| Median | 51.967 | 45.699 | 50.348 | 51.947 | 51.513 | 54.576 | 56.466 | 56.507 |
| 25th percentile | 43.524 | 37.051 | 43.222 | 43.010 | 43.876 | 46.446 | 51.781 | 51.111 |
| 75th percentile | 57.373 | 52.846 | 56.268 | 56.986 | 56.968 | 59.429 | 59.998 | 59.783 |
| Minimum | 11.897 | 11.897 | 14.330 | 18.715 | 14.148 | 17.963 | 18.033 | 41.784 |
| Maximum | 67.974 | 67.290 | 64.897 | 66.008 | 64.130 | 67.974 | 67.596 | 65.043 |
| *VR-12 domain scores* |  |  |  |  |  |  |  |  |
| PF     Mean | 74.490 | 79.111 | 80.897 | 79.188 | 73.421 | 69.268 | 66.115 | 60.990 |
| Standard deviation | 24.852 | 21.300 | 19.026 | 20.806 | 25.106 | 31.695 | 24.828 | 48.716 |
| Median | 93.485 | 93.485 | 93.485 | 93.485 | 80.375 | 80.375 | 71.400 | 58.290 |
| 25th percentile | 58.290 | 71.400 | 71.400 | 71.400 | 58.290 | 58.290 | 58.290 | 36.595 |
| 75th percentile | 93.485 | 93.485 | 93.485 | 93.485 | 93.485 | 93.485 | 93.485 | 93.485 |
| Minimum | 7.765 | 7.765 | 7.765 | 7.765 | 7.765 | 7.765 | 7.765 | 7.765 |
| Maximum | 93.485 | 93.485 | 93.485 | 93.485 | 93.485 | 93.485 | 93.485 | 93.485 |
| RP     Mean | 73.871 | 76.060 | 79.554 | 79.576 | 74.333 | 72.096 | 68.162 | 50.656 |
| Standard deviation | 35.526 | 33.624 | 30.476 | 30.562 | 34.097 | 42.129 | 33.535 | 76.469 |
| Median | 89.670 | 89.670 | 100.690 | 100.690 | 89.670 | 89.670 | 89.670 | 79.405 |
| 25th percentile | 51.210 | 55.740 | 79.405 | 79.405 | 54.725 | 44.720 | 44.460 | 9.775 |
| 75th percentile | 100.690 | 100.690 | 100.690 | 100.690 | 100.690 | 100.690 | 100.690 | 89.670 |
| Minimum | -3.670 | -3.670 | -3.670 | -3.670 | -3.670 | -3.670 | -3.670 | -3.670 |
| Maximum | 100.690 | 100.690 | 100.690 | 100.690 | 100.690 | 100.690 | 100.690 | 100.690 |
| BP     Mean | 68.933 | 70.928 | 72.464 | 70.226 | 67.947 | 66.446 | 64.170 | 66.878 |
| Standard deviation | 25.636 | 23.895 | 26.820 | 22.612 | 25.508 | 29.883 | 22.245 | 47.714 |
| Median | 56.250 | 56.250 | 95.200 | 56.250 | 56.250 | 56.250 | 56.250 | 56.250 |
| 25th percentile | 56.250 | 56.250 | 56.250 | 56.250 | 56.250 | 56.250 | 43.890 | 43.890 |
| 75th percentile | 95.200 | 95.200 | 95.200 | 95.200 | 95.200 | 95.200 | 95.200 | 95.200 |
| Minimum | 2.280 | 2.280 | 2.280 | 2.280 | 2.280 | 2.280 | 2.280 | 2.280 |
| Maximum | 95.200 | 95.200 | 95.200 | 95.200 | 95.200 | 95.200 | 95.200 | 95.200 |
| GH    Mean | 72.083 | 77.923 | 76.786 | 71.920 | 71.188 | 67.917 | 68.932 | 59.468 |
| Standard deviation | 21.691 | 18.590 | 19.454 | 19.493 | 20.252 | 26.952 | 19.628 | 46.698 |
| Median | 83.710 | 83.710 | 83.710 | 83.710 | 83.710 | 61.500 | 61.500 | 61.500 |
| 25th percentile | 61.500 | 61.500 | 61.500 | 61.500 | 61.500 | 61.500 | 61.500 | 37.500 |
| 75th percentile | 83.710 | 83.710 | 83.710 | 83.710 | 83.710 | 83.710 | 83.710 | 83.710 |
| Minimum | 0.580 | 0.580 | 0.580 | 0.580 | 0.580 | 0.580 | 0.580 | 0.580 |
| Maximum | 101.840 | 101.840 | 101.840 | 101.840 | 101.840 | 101.840 | 101.840 | 101.840 |
| VT     Mean | 54.596 | 53.310 | 59.992 | 54.518 | 53.383 | 54.641 | 53.873 | 49.475 |
| Standard deviation | 23.178 | 25.389 | 23.543 | 19.419 | 21.207 | 25.673 | 18.677 | 47.500 |
| Median | 54.550 | 54.550 | 54.550 | 54.550 | 54.550 | 45.900 | 54.550 | 45.900 |
| 25th percentile | 45.900 | 45.900 | 45.900 | 45.900 | 45.900 | 45.900 | 45.900 | 31.930 |
| 75th percentile | 79.250 | 79.250 | 79.250 | 79.250 | 79.250 | 79.250 | 79.250 | 79.250 |
| Minimum | 1.800 | 1.800 | 1.800 | 1.800 | 1.800 | 1.800 | 1.800 | 1.800 |
| Maximum | 95.340 | 95.340 | 95.340 | 95.340 | 95.340 | 95.340 | 95.340 | 95.340 |
| SF      Mean | 74.067 | 64.058 | 72.566 | 73.418 | 74.975 | 78.296 | 83.665 | 82.958 |
| Standard deviation | 28.435 | 28.423 | 28.081 | 26.963 | 28.364 | 31.485 | 20.903 | 42.047 |
| Median | 66.870 | 66.870 | 66.870 | 66.870 | 99.320 | 99.320 | 99.320 | 99.320 |
| 25th percentile | 48.870 | 48.870 | 48.870 | 48.870 | 48.870 | 66.870 | 66.870 | 66.870 |
| 75th percentile | 99.320 | 99.320 | 99.320 | 99.320 | 99.320 | 99.320 | 99.320 | 99.320 |
| Minimum | 2.780 | 2.780 | 2.780 | 2.780 | 2.780 | 2.780 | 2.780 | 24.910 |
| Maximum | 99.320 | 99.320 | 99.320 | 99.320 | 99.320 | 99.320 | 99.320 | 99.320 |
| MH   Mean | 69.365 | 59.788 | 68.735 | 67.844 | 68.101 | 74.226 | 78.058 | 82.603 |
| Standard deviation | 22.379 | 23.528 | 21.365 | 20.188 | 22.483 | 23.410 | 16.521 | 23.323 |
| Median | 74.865 | 60.685 | 74.865 | 74.865 | 74.865 | 74.865 | 83.670 | 89.615 |
| 25th percentile | 51.325 | 41.685 | 51.325 | 51.325 | 51.325 | 65.505 | 65.505 | 69.490 |
| 75th percentile | 83.845 | 83.670 | 83.670 | 83.845 | 83.670 | 92.650 | 92.650 | 92.650 |
| Minimum | -1.030 | -1.030 | -1.030 | -1.030 | -1.030 | -1.030 | 6.005 | 51.325 |
| Maximum | 98.595 | 98.595 | 98.595 | 98.595 | 98.595 | 98.595 | 98.595 | 98.595 |
| RE     Mean | 72.102 | 57.146 | 71.712 | 77.414 | 69.712 | 81.376 | 82.555 | 74.251 |
| Standard deviation | 37.811 | 39.925 | 37.733 | 32.440 | 39.363 | 38.415 | 28.152 | 56.986 |
| Median | 81.810 | 54.690 | 81.810 | 104.125 | 81.810 | 104.125 | 104.125 | 77.005 |
| 25th percentile | 42.775 | 29.335 | 54.690 | 54.690 | 41.285 | 54.690 | 68.370 | 42.775 |
| 75th percentile | 104.125 | 104.125 | 104.125 | 104.125 | 104.125 | 104.125 | 104.125 | 104.125 |
| Minimum | -33.415 | -33.415 | -33.415 | -33.415 | -33.415 | -33.415 | -33.415 | -3.985 |
| Maximum | 104.125 | 104.125 | 104.125 | 104.125 | 104.125 | 104.125 | 104.125 | 104.125 |

BP, bodily pain; GH, general health; MCS, mental component summary; MH, mental health; PCS, physical component summary; PF, physical functioning; RE, role emotional; RP, role physical; VT, vitality; SF, social functioning

**SM3 Table 14**. Canadian norms for VR-12 health utility values, summary component scores, and domain scores, by age group – Ontario, males only.

|  | All participants (n=1261) | 18 to 29 (n=231) | 30 to 39 (n=205) | 40 to 49 (n=228) | 50 to 59 (n=235) | 60 to 69 (n=101) | 70 to 79 (n=229) | 80 and over (n=32) |
| --- | --- | --- | --- | --- | --- | --- | --- | --- |
| *VR-12 health utility values* |  |  |  |  |  |  |  |  |
| Mean | 0.702 | 0.680 | 0.705 | 0.682 | 0.701 | 0.732 | 0.729 | 0.704 |
| Standard deviation | 0.192 | 0.180 | 0.192 | 0.197 | 0.204 | 0.209 | 0.156 | 0.304 |
| Median | 0.745 | 0.710 | 0.736 | 0.746 | 0.752 | 0.778 | 0.769 | 0.758 |
| 25th percentile | 0.644 | 0.570 | 0.644 | 0.606 | 0.644 | 0.664 | 0.644 | 0.588 |
| 75th percentile | 0.829 | 0.789 | 0.822 | 0.820 | 0.839 | 0.840 | 0.873 | 0.829 |
| Minimum | -0.513 | -0.263 | -0.358 | -0.218 | -0.513 | 0.011 | -0.077 | 0.388 |
| Maximum | 1.000 | 1.000 | 1.000 | 0.960 | 1.000 | 1.000 | 1.000 | 0.952 |
| *VR-12 summary component score* |  |  |  |  |  |  |  |  |
| PCS   Mean | 49.280 | 52.166 | 52.918 | 50.537 | 49.575 | 47.488 | 45.443 | 40.409 |
| Standard deviation | 9.616 | 8.425 | 7.283 | 8.792 | 8.993 | 9.286 | 8.577 | 19.234 |
| Median | 51.453 | 54.838 | 55.699 | 54.006 | 52.562 | 47.637 | 46.971 | 43.116 |
| 25th percentile | 43.346 | 46.893 | 49.217 | 46.313 | 45.206 | 43.983 | 37.758 | 27.157 |
| 75th percentile | 56.824 | 58.388 | 57.863 | 56.949 | 57.055 | 54.704 | 54.395 | 47.060 |
| Minimum | 10.492 | 25.774 | 28.850 | 10.494 | 10.492 | 19.175 | 10.844 | 23.854 |
| Maximum | 68.528 | 68.528 | 64.708 | 64.732 | 63.819 | 59.701 | 65.528 | 59.100 |
| MCS  Mean | 50.366 | 46.083 | 48.809 | 48.924 | 50.839 | 53.468 | 55.128 | 54.412 |
| Standard deviation | 9.299 | 10.422 | 9.063 | 8.717 | 8.159 | 9.153 | 6.541 | 11.737 |
| Median | 52.305 | 47.132 | 49.601 | 51.790 | 53.003 | 55.218 | 57.146 | 52.928 |
| 25th percentile | 44.413 | 40.770 | 43.287 | 40.936 | 45.969 | 48.203 | 52.544 | 51.111 |
| 75th percentile | 58.202 | 52.971 | 55.768 | 56.968 | 57.931 | 59.112 | 60.376 | 60.388 |
| Minimum | 11.897 | 11.897 | 20.278 | 20.505 | 20.559 | 27.976 | 18.033 | 41.784 |
| Maximum | 67.596 | 65.964 | 64.897 | 66.008 | 64.130 | 64.985 | 67.596 | 65.043 |
| *VR-12 domain scores* |  |  |  |  |  |  |  |  |
| PF     Mean | 75.659 | 78.994 | 83.449 | 79.021 | 76.250 | 73.738 | 68.695 | 58.234 |
| Standard deviation | 23.667 | 23.901 | 17.969 | 21.491 | 22.196 | 23.848 | 21.693 | 46.558 |
| Median | 93.485 | 93.485 | 93.485 | 93.485 | 93.485 | 80.375 | 80.375 | 58.290 |
| 25th percentile | 58.290 | 71.400 | 71.400 | 71.400 | 58.290 | 58.290 | 58.290 | 36.595 |
| 75th percentile | 93.485 | 93.485 | 93.485 | 93.485 | 93.485 | 93.485 | 93.485 | 80.375 |
| Minimum | 7.765 | 7.765 | 7.765 | 7.765 | 7.765 | 7.765 | 7.765 | 7.765 |
| Maximum | 93.485 | 93.485 | 93.485 | 93.485 | 93.485 | 93.485 | 93.485 | 93.485 |
| RP     Mean | 73.981 | 75.054 | 79.583 | 79.407 | 75.148 | 78.451 | 70.555 | 48.630 |
| Standard deviation | 34.529 | 36.593 | 29.713 | 29.565 | 32.512 | 34.210 | 29.392 | 74.205 |
| Median | 89.670 | 89.670 | 100.690 | 100.690 | 100.690 | 89.670 | 79.405 | 44.720 |
| 25th percentile | 51.210 | 55.740 | 54.725 | 79.405 | 54.725 | 79.405 | 44.720 | 5.245 |
| 75th percentile | 100.690 | 100.690 | 100.690 | 100.690 | 100.690 | 100.690 | 100.690 | 79.405 |
| Minimum | -3.670 | -3.670 | -3.670 | -3.670 | -3.670 | -3.670 | -3.670 | 0.490 |
| Maximum | 100.690 | 100.690 | 100.690 | 100.690 | 100.690 | 100.690 | 100.690 | 100.690 |
| BP     Mean | 68.894 | 70.536 | 70.761 | 68.653 | 68.880 | 68.465 | 66.278 | 66.067 |
| Standard deviation | 25.035 | 25.797 | 28.650 | 21.586 | 22.793 | 25.814 | 21.341 | 46.863 |
| Median | 56.250 | 56.250 | 95.200 | 56.250 | 56.250 | 56.250 | 56.250 | 56.250 |
| 25th percentile | 56.250 | 56.250 | 56.250 | 56.250 | 56.250 | 56.250 | 56.250 | 43.890 |
| 75th percentile | 95.200 | 95.200 | 95.200 | 95.200 | 95.200 | 95.200 | 95.200 | 95.200 |
| Minimum | 2.280 | 2.280 | 2.280 | 2.280 | 2.280 | 31.500 | 2.280 | 31.500 |
| Maximum | 95.200 | 95.200 | 95.200 | 95.200 | 95.200 | 95.200 | 95.200 | 95.200 |
| GH    Mean | 72.176 | 81.197 | 79.862 | 69.425 | 70.310 | 66.264 | 68.830 | 55.518 |
| Standard deviation | 21.926 | 19.090 | 19.274 | 21.423 | 18.892 | 24.313 | 17.121 | 44.934 |
| Median | 83.710 | 83.710 | 83.710 | 83.710 | 83.710 | 61.500 | 61.500 | 61.500 |
| 25th percentile | 61.500 | 61.500 | 61.500 | 61.500 | 61.500 | 61.500 | 61.500 | 37.500 |
| 75th percentile | 83.710 | 101.840 | 83.710 | 83.710 | 83.710 | 83.710 | 83.710 | 83.710 |
| Minimum | 0.580 | 0.580 | 0.580 | 0.580 | 0.580 | 0.580 | 0.580 | 0.580 |
| Maximum | 101.840 | 101.840 | 101.840 | 101.840 | 101.840 | 101.840 | 101.840 | 101.840 |
| VT     Mean | 56.464 | 57.385 | 63.793 | 53.812 | 57.310 | 54.616 | 55.805 | 45.944 |
| Standard deviation | 22.043 | 24.060 | 22.054 | 18.522 | 19.053 | 22.609 | 18.156 | 46.156 |
| Median | 54.550 | 54.550 | 54.550 | 54.550 | 54.550 | 45.900 | 54.550 | 45.900 |
| 25th percentile | 45.900 | 45.900 | 45.900 | 45.900 | 45.900 | 45.900 | 45.900 | 31.930 |
| 75th percentile | 79.250 | 79.250 | 79.250 | 79.250 | 79.250 | 79.250 | 79.250 | 79.250 |
| Minimum | 1.800 | 1.800 | 1.800 | 1.800 | 1.800 | 1.800 | 1.800 | 1.800 |
| Maximum | 95.340 | 95.340 | 95.340 | 95.340 | 95.340 | 95.340 | 95.340 | 95.340 |
| SF      Mean | 74.764 | 65.658 | 70.509 | 72.578 | 80.013 | 80.125 | 83.054 | 80.362 |
| Standard deviation | 27.158 | 29.110 | 28.858 | 25.789 | 24.724 | 28.498 | 20.026 | 40.000 |
| Median | 66.870 | 66.870 | 66.870 | 66.870 | 99.320 | 99.320 | 99.320 | 99.320 |
| 25th percentile | 48.870 | 48.870 | 48.870 | 48.870 | 66.870 | 66.870 | 66.870 | 66.870 |
| 75th percentile | 99.320 | 99.320 | 99.320 | 99.320 | 99.320 | 99.320 | 99.320 | 99.320 |
| Minimum | 2.780 | 2.780 | 2.780 | 2.780 | 2.780 | 2.780 | 2.780 | 48.870 |
| Maximum | 99.320 | 99.320 | 99.320 | 99.320 | 99.320 | 99.320 | 99.320 | 99.320 |
| MH   Mean | 72.204 | 65.082 | 71.275 | 66.975 | 71.509 | 77.169 | 80.244 | 83.107 |
| Standard deviation | 20.216 | 21.746 | 19.161 | 19.949 | 18.685 | 22.681 | 14.741 | 21.874 |
| Median | 74.865 | 65.795 | 74.865 | 74.865 | 83.670 | 83.670 | 83.670 | 89.615 |
| 25th percentile | 60.685 | 51.325 | 60.685 | 51.325 | 55.620 | 65.505 | 74.865 | 65.505 |
| 75th percentile | 89.615 | 83.670 | 83.670 | 83.845 | 83.670 | 92.650 | 92.650 | 92.650 |
| Minimum | -1.030 | -1.030 | -1.030 | 6.005 | 6.005 | 21.060 | 6.005 | 60.685 |
| Maximum | 98.595 | 98.595 | 98.595 | 98.595 | 98.595 | 98.595 | 98.595 | 98.595 |
| RE     Mean | 72.234 | 57.510 | 66.300 | 76.317 | 74.895 | 85.879 | 86.128 | 70.271 |
| Standard deviation | 36.567 | 42.165 | 39.736 | 30.359 | 34.919 | 32.151 | 23.725 | 58.722 |
| Median | 81.810 | 54.690 | 68.370 | 104.125 | 104.125 | 104.125 | 104.125 | 77.005 |
| 25th percentile | 42.775 | 29.335 | 42.775 | 54.690 | 54.690 | 77.005 | 77.005 | 41.285 |
| 75th percentile | 104.125 | 104.125 | 104.125 | 104.125 | 104.125 | 104.125 | 104.125 | 104.125 |
| Minimum | -33.415 | -33.415 | -33.415 | -33.415 | -33.415 | -20.070 | -33.415 | -3.985 |
| Maximum | 104.125 | 104.125 | 104.125 | 104.125 | 104.125 | 104.125 | 104.125 | 104.125 |

BP, bodily pain; GH, general health; MCS, mental component summary; MH, mental health; PCS, physical component summary; PF, physical functioning; RE, role emotional; RP, role physical; VT, vitality; SF, social functioning

**SM3 Table 15**. Canadian norms for VR-12 health utility values, summary component scores, and domain scores, by age group – Ontario, females only.

|  | All participants (n=1342) | 18 to 29 (n=283) | 30 to 39 (n=224) | 40 to 49 (n=268) | 50 to 59 (n=239) | 60 to 69 (n=174) | 70 to 79 (n=139) | 80 and over (n=15) |
| --- | --- | --- | --- | --- | --- | --- | --- | --- |
| *VR-12 health utility values* |  |  |  |  |  |  |  |  |
| Mean | 0.664 | 0.604 | 0.688 | 0.695 | 0.624 | 0.689 | 0.668 | 0.764 |
| Standard deviation | 0.253 | 0.247 | 0.235 | 0.212 | 0.288 | 0.270 | 0.244 | 0.334 |
| Median | 0.736 | 0.696 | 0.746 | 0.749 | 0.736 | 0.758 | 0.778 | 0.777 |
| 25th percentile | 0.573 | 0.456 | 0.615 | 0.644 | 0.556 | 0.600 | 0.521 | 0.697 |
| 75th percentile | 0.818 | 0.779 | 0.822 | 0.852 | 0.818 | 0.818 | 0.827 | 0.913 |
| Minimum | -0.484 | -0.258 | -0.419 | -0.242 | -0.484 | -0.393 | -0.134 | 0.231 |
| Maximum | 1.000 | 1.000 | 1.000 | 1.000 | 1.000 | 1.000 | 1.000 | 0.950 |
| *VR-12 summary component score* |  |  |  |  |  |  |  |  |
| PCS   Mean | 49.228 | 53.001 | 51.280 | 51.180 | 48.811 | 45.946 | 44.007 | 44.024 |
| Standard deviation | 10.893 | 7.437 | 8.837 | 8.462 | 11.547 | 14.144 | 11.644 | 25.190 |
| Median | 52.409 | 55.542 | 54.034 | 53.837 | 52.066 | 48.063 | 47.504 | 42.018 |
| 25th percentile | 43.131 | 49.184 | 46.192 | 47.715 | 44.224 | 37.816 | 33.197 | 38.572 |
| 75th percentile | 57.246 | 58.232 | 57.763 | 57.077 | 56.824 | 56.824 | 55.843 | 55.653 |
| Minimum | 4.409 | 19.499 | 10.489 | 15.070 | 5.715 | 12.719 | 15.956 | 4.409 |
| Maximum | 67.108 | 66.389 | 65.678 | 64.566 | 67.108 | 62.714 | 61.267 | 59.833 |
| MCS  Mean | 48.943 | 42.883 | 49.138 | 49.406 | 47.567 | 52.240 | 53.334 | 55.871 |
| Standard deviation | 11.005 | 11.139 | 10.272 | 9.561 | 11.473 | 10.744 | 7.751 | 11.835 |
| Median | 51.752 | 44.671 | 50.808 | 52.151 | 51.110 | 53.968 | 55.308 | 56.507 |
| 25th percentile | 43.219 | 33.505 | 42.195 | 43.919 | 42.340 | 45.640 | 51.781 | 50.307 |
| 75th percentile | 56.986 | 52.627 | 56.980 | 57.187 | 55.756 | 59.588 | 59.576 | 59.608 |
| Minimum | 14.148 | 14.151 | 14.330 | 18.715 | 14.148 | 17.963 | 27.192 | 43.640 |
| Maximum | 67.974 | 67.290 | 64.089 | 62.711 | 63.031 | 67.974 | 65.313 | 64.318 |
| *VR-12 domain scores* |  |  |  |  |  |  |  |  |
| PF     Mean | 73.394 | 78.983 | 78.351 | 79.314 | 71.220 | 67.020 | 62.704 | 65.997 |
| Standard deviation | 25.872 | 18.961 | 19.682 | 20.246 | 27.514 | 35.208 | 29.061 | 53.278 |
| Median | 80.375 | 93.485 | 93.485 | 93.485 | 80.375 | 80.375 | 71.400 | 58.290 |
| 25th percentile | 58.290 | 71.400 | 58.290 | 58.290 | 58.290 | 58.290 | 36.595 | 58.290 |
| 75th percentile | 93.485 | 93.485 | 93.485 | 93.485 | 93.485 | 93.485 | 93.485 | 93.485 |
| Minimum | 7.765 | 7.765 | 7.765 | 7.765 | 7.765 | 7.765 | 7.765 | 7.765 |
| Maximum | 93.485 | 93.485 | 93.485 | 93.485 | 93.485 | 93.485 | 93.485 | 93.485 |
| RP     Mean | 73.734 | 76.864 | 79.525 | 79.703 | 73.700 | 68.900 | 64.999 | 54.336 |
| Standard deviation | 36.469 | 31.136 | 31.224 | 31.439 | 35.642 | 45.703 | 39.352 | 83.273 |
| Median | 89.670 | 89.670 | 100.690 | 100.690 | 89.670 | 89.670 | 89.670 | 79.405 |
| 25th percentile | 51.210 | 54.725 | 79.405 | 79.405 | 54.725 | 44.460 | 9.775 | 9.775 |
| 75th percentile | 100.690 | 100.690 | 100.690 | 100.690 | 100.690 | 100.690 | 100.690 | 100.690 |
| Minimum | -3.670 | -3.670 | -3.670 | -3.670 | -3.670 | -3.670 | -3.670 | -3.670 |
| Maximum | 100.690 | 100.690 | 100.690 | 100.690 | 100.690 | 100.690 | 100.690 | 100.690 |
| BP     Mean | 69.026 | 71.663 | 74.163 | 71.408 | 67.221 | 65.430 | 61.383 | 68.353 |
| Standard deviation | 26.201 | 22.237 | 24.988 | 23.429 | 27.948 | 32.009 | 23.494 | 51.026 |
| Median | 56.250 | 56.250 | 95.200 | 56.250 | 56.250 | 56.250 | 56.250 | 56.250 |
| 25th percentile | 56.250 | 56.250 | 56.250 | 56.250 | 56.250 | 43.890 | 43.890 | 56.250 |
| 75th percentile | 95.200 | 95.200 | 95.200 | 95.200 | 95.200 | 95.200 | 95.200 | 95.200 |
| Minimum | 2.280 | 2.280 | 2.280 | 2.280 | 2.280 | 2.280 | 2.280 | 2.280 |
| Maximum | 95.200 | 95.200 | 95.200 | 95.200 | 95.200 | 95.200 | 95.200 | 95.200 |
| GH    Mean | 71.948 | 74.623 | 73.717 | 73.794 | 71.871 | 68.748 | 69.066 | 66.645 |
| Standard deviation | 21.477 | 17.705 | 19.211 | 17.525 | 21.521 | 28.385 | 23.243 | 48.648 |
| Median | 83.710 | 83.710 | 83.710 | 83.710 | 83.710 | 83.710 | 83.710 | 61.500 |
| 25th percentile | 61.500 | 61.500 | 61.500 | 61.500 | 61.500 | 61.500 | 61.500 | 61.500 |
| 75th percentile | 83.710 | 83.710 | 83.710 | 83.710 | 83.710 | 83.710 | 83.710 | 83.710 |
| Minimum | 0.580 | 0.580 | 0.580 | 0.580 | 0.580 | 0.580 | 0.580 | 0.580 |
| Maximum | 101.840 | 101.840 | 101.840 | 101.840 | 101.840 | 101.840 | 101.840 | 101.840 |
| VT     Mean | 52.967 | 49.420 | 56.199 | 55.049 | 50.329 | 54.653 | 51.320 | 55.892 |
| Standard deviation | 24.097 | 25.995 | 24.334 | 20.171 | 22.699 | 27.357 | 19.324 | 49.340 |
| Median | 54.550 | 45.900 | 54.550 | 54.550 | 54.550 | 54.550 | 54.550 | 45.900 |
| 25th percentile | 45.900 | 31.930 | 45.900 | 45.900 | 31.930 | 45.900 | 31.930 | 31.930 |
| 75th percentile | 79.250 | 79.250 | 79.250 | 79.250 | 54.550 | 79.250 | 54.550 | 79.250 |
| Minimum | 1.800 | 1.800 | 1.800 | 1.800 | 1.800 | 1.800 | 1.800 | 1.800 |
| Maximum | 95.340 | 95.340 | 95.340 | 95.340 | 95.340 | 95.340 | 95.340 | 95.340 |
| SF      Mean | 73.588 | 62.977 | 74.619 | 74.050 | 71.056 | 77.376 | 84.472 | 87.675 |
| Standard deviation | 29.462 | 27.419 | 27.272 | 27.957 | 31.019 | 33.118 | 22.326 | 46.092 |
| Median | 66.870 | 66.870 | 99.320 | 99.320 | 66.870 | 99.320 | 99.320 | 99.320 |
| 25th percentile | 48.870 | 48.870 | 48.870 | 48.870 | 48.870 | 66.870 | 66.870 | 99.320 |
| 75th percentile | 99.320 | 99.320 | 99.320 | 99.320 | 99.320 | 99.320 | 99.320 | 99.320 |
| Minimum | 2.780 | 2.780 | 2.780 | 2.780 | 2.780 | 2.780 | 2.780 | 24.910 |
| Maximum | 99.320 | 99.320 | 99.320 | 99.320 | 99.320 | 99.320 | 99.320 | 99.320 |
| MH   Mean | 66.982 | 55.045 | 66.201 | 68.497 | 65.450 | 72.746 | 75.167 | 81.686 |
| Standard deviation | 23.880 | 23.733 | 22.985 | 20.405 | 25.398 | 23.681 | 18.828 | 26.880 |
| Median | 74.865 | 55.620 | 74.485 | 74.865 | 74.865 | 74.865 | 83.670 | 83.845 |
| 25th percentile | 51.325 | 31.800 | 49.545 | 51.325 | 51.045 | 60.685 | 60.685 | 69.490 |
| 75th percentile | 83.670 | 74.865 | 83.670 | 83.845 | 83.670 | 83.845 | 92.650 | 92.650 |
| Minimum | -1.030 | 6.005 | -1.030 | -1.030 | -1.030 | -1.030 | 16.745 | 51.325 |
| Maximum | 98.595 | 98.595 | 98.595 | 98.595 | 98.595 | 98.595 | 98.595 | 98.595 |
| RE     Mean | 72.247 | 57.803 | 77.112 | 78.239 | 65.682 | 79.111 | 77.831 | 81.482 |
| Standard deviation | 38.714 | 37.332 | 35.131 | 34.147 | 42.928 | 41.434 | 33.831 | 51.979 |
| Median | 81.810 | 54.690 | 104.125 | 104.125 | 81.810 | 104.125 | 104.125 | 104.125 |
| 25th percentile | 42.775 | 29.335 | 54.690 | 54.690 | 29.335 | 54.690 | 54.690 | 54.690 |
| 75th percentile | 104.125 | 104.125 | 104.125 | 104.125 | 104.125 | 104.125 | 104.125 | 104.125 |
| Minimum | -33.415 | -33.415 | -33.415 | -33.415 | -33.415 | -33.415 | -33.415 | 41.250 |
| Maximum | 104.125 | 104.125 | 104.125 | 104.125 | 104.125 | 104.125 | 104.125 | 104.125 |

BP, bodily pain; GH, general health; MCS, mental component summary; MH, mental health; PCS, physical component summary; PF, physical functioning; RE, role emotional; RP, role physical; VT, vitality; SF, social functioning

**SM3 Table 16**. Canadian norms for VR-12 health utility values, summary component scores, and domain scores, by age group – Quebec.

|  | All participants (n=1385) | 18 to 29 (n=283) | 30 to 39 (n=266) | 40 to 49 (n=285) | 50 to 59 (n=302) | 60 to 69 (n=148) | 70 to 79 (n=96) | 80 and over (n=5) |
| --- | --- | --- | --- | --- | --- | --- | --- | --- |
| *VR-12 health utility values* |  |  |  |  |  |  |  |  |
| Mean | 0.734 | 0.702 | 0.718 | 0.751 | 0.732 | 0.767 | 0.754 | 0.844 |
| Standard deviation | 0.190 | 0.185 | 0.192 | 0.169 | 0.210 | 0.189 | 0.180 | 0.121 |
| Median | 0.768 | 0.746 | 0.749 | 0.793 | 0.778 | 0.799 | 0.789 | 0.879 |
| 25th percentile | 0.678 | 0.610 | 0.678 | 0.696 | 0.696 | 0.696 | 0.697 | 0.839 |
| 75th percentile | 0.852 | 0.818 | 0.818 | 0.852 | 0.852 | 0.879 | 0.879 | 0.883 |
| Minimum | -0.489 | -0.139 | -0.383 | -0.305 | -0.489 | 0.061 | 0.203 | 0.717 |
| Maximum | 1.000 | 1.000 | 1.000 | 1.000 | 1.000 | 1.000 | 1.000 | 0.919 |
| *VR-12 summary component score* |  |  |  |  |  |  |  |  |
| PCS   Mean | 51.603 | 54.915 | 53.995 | 52.636 | 49.531 | 47.798 | 45.970 | 50.042 |
| Standard deviation | 9.363 | 6.583 | 7.390 | 7.579 | 10.930 | 11.366 | 10.237 | 9.578 |
| Median | 54.344 | 56.824 | 56.247 | 54.885 | 53.500 | 49.365 | 49.017 | 52.746 |
| 25th percentile | 47.632 | 51.924 | 50.687 | 49.019 | 45.377 | 41.148 | 39.757 | 45.648 |
| 75th percentile | 57.768 | 58.744 | 58.413 | 57.899 | 57.155 | 55.963 | 54.229 | 54.480 |
| Minimum | 13.059 | 20.035 | 21.086 | 16.904 | 13.059 | 16.988 | 21.969 | 42.029 |
| Maximum | 70.042 | 70.042 | 65.605 | 65.783 | 64.573 | 63.207 | 63.575 | 56.216 |
| MCS  Mean | 50.823 | 46.672 | 48.499 | 51.361 | 52.432 | 54.445 | 55.260 | 59.351 |
| Standard deviation | 9.511 | 10.261 | 9.693 | 8.659 | 8.065 | 9.113 | 6.566 | 2.548 |
| Median | 52.634 | 49.543 | 49.339 | 53.316 | 53.812 | 56.980 | 55.788 | 59.088 |
| 25th percentile | 45.691 | 39.409 | 44.190 | 46.225 | 48.021 | 50.552 | 53.823 | 58.376 |
| 75th percentile | 57.657 | 53.858 | 55.058 | 57.823 | 57.893 | 59.763 | 60.120 | 60.516 |
| Minimum | 8.848 | 8.848 | 17.082 | 15.291 | 18.311 | 30.455 | 37.173 | 57.393 |
| Maximum | 67.832 | 62.699 | 66.386 | 67.113 | 67.832 | 64.291 | 65.941 | 61.653 |
| *VR-12 domain scores* |  |  |  |  |  |  |  |  |
| PF     Mean | 81.288 | 85.218 | 85.403 | 84.743 | 78.080 | 74.070 | 71.661 | 83.952 |
| Standard deviation | 22.555 | 15.772 | 18.343 | 17.903 | 26.520 | 29.669 | 27.751 | 24.219 |
| Median | 93.485 | 93.485 | 93.485 | 93.485 | 93.485 | 80.375 | 80.375 | 93.485 |
| 25th percentile | 80.375 | 80.375 | 80.375 | 80.375 | 71.400 | 58.290 | 58.290 | 80.375 |
| 75th percentile | 93.485 | 93.485 | 93.485 | 93.485 | 93.485 | 93.485 | 93.485 | 93.485 |
| Minimum | 7.765 | 7.765 | 7.765 | 7.765 | 7.765 | 7.765 | 7.765 | 58.290 |
| Maximum | 93.485 | 93.485 | 93.485 | 93.485 | 93.485 | 93.485 | 93.485 | 93.485 |
| RP     Mean | 77.662 | 85.503 | 81.029 | 81.157 | 73.768 | 69.679 | 62.907 | 77.124 |
| Standard deviation | 35.931 | 26.389 | 33.217 | 30.767 | 39.427 | 46.476 | 42.457 | 40.920 |
| Median | 100.690 | 100.690 | 100.690 | 100.690 | 100.690 | 90.425 | 89.670 | 89.670 |
| 25th percentile | 55.740 | 79.405 | 79.405 | 79.405 | 44.720 | 44.460 | 9.775 | 55.740 |
| 75th percentile | 100.690 | 100.690 | 100.690 | 100.690 | 100.690 | 100.690 | 100.690 | 100.690 |
| Minimum | -3.670 | -3.670 | -3.670 | -3.670 | -3.670 | 0.490 | 0.490 | 44.720 |
| Maximum | 100.690 | 100.690 | 100.690 | 100.690 | 100.690 | 100.690 | 100.690 | 100.690 |
| BP     Mean | 73.559 | 77.104 | 76.989 | 75.225 | 68.757 | 70.501 | 68.815 | 75.966 |
| Standard deviation | 25.644 | 24.245 | 24.668 | 24.294 | 26.809 | 27.791 | 25.003 | 39.385 |
| Median | 95.200 | 95.200 | 95.200 | 95.200 | 56.250 | 56.250 | 56.250 | 95.200 |
| 25th percentile | 56.250 | 56.250 | 56.250 | 56.250 | 56.250 | 56.250 | 56.250 | 56.250 |
| 75th percentile | 95.200 | 95.200 | 95.200 | 95.200 | 95.200 | 95.200 | 95.200 | 95.200 |
| Minimum | 2.280 | 2.280 | 2.280 | 2.280 | 2.280 | 31.500 | 31.500 | 43.890 |
| Maximum | 95.200 | 95.200 | 95.200 | 95.200 | 95.200 | 95.200 | 95.200 | 95.200 |
| GH    Mean | 76.691 | 81.573 | 80.140 | 78.825 | 73.827 | 69.652 | 68.289 | 82.395 |
| Standard deviation | 20.339 | 19.155 | 17.869 | 18.107 | 21.079 | 24.201 | 19.714 | 21.329 |
| Median | 83.710 | 83.710 | 83.710 | 83.710 | 83.710 | 83.710 | 83.710 | 83.710 |
| 25th percentile | 61.500 | 83.710 | 61.500 | 61.500 | 61.500 | 61.500 | 61.500 | 83.710 |
| 75th percentile | 83.710 | 101.840 | 83.710 | 83.710 | 83.710 | 83.710 | 83.710 | 83.710 |
| Minimum | 0.580 | 0.580 | 0.580 | 0.580 | 0.580 | 0.580 | 0.580 | 61.500 |
| Maximum | 101.840 | 101.840 | 101.840 | 101.840 | 101.840 | 101.840 | 101.840 | 101.840 |
| VT     Mean | 59.040 | 56.312 | 56.776 | 60.949 | 60.518 | 62.360 | 57.246 | 59.435 |
| Standard deviation | 22.310 | 23.911 | 22.681 | 19.077 | 21.063 | 27.263 | 19.395 | 17.042 |
| Median | 54.550 | 54.550 | 54.550 | 54.550 | 54.550 | 54.550 | 54.550 | 54.550 |
| 25th percentile | 45.900 | 45.900 | 45.900 | 45.900 | 45.900 | 45.900 | 45.900 | 54.550 |
| 75th percentile | 79.250 | 79.250 | 79.250 | 79.250 | 79.250 | 79.250 | 79.250 | 54.550 |
| Minimum | 1.800 | 1.800 | 1.800 | 1.800 | 1.800 | 1.800 | 31.930 | 54.550 |
| Maximum | 95.340 | 95.340 | 95.340 | 95.340 | 95.340 | 95.340 | 95.340 | 79.250 |
| SF      Mean | 78.108 | 70.297 | 73.618 | 79.797 | 80.393 | 84.254 | 87.999 | 99.320 |
| Standard deviation | 27.326 | 30.587 | 28.078 | 25.441 | 24.298 | 26.142 | 22.546 | 0.000 |
| Median | 99.320 | 66.870 | 66.870 | 99.320 | 99.320 | 99.320 | 99.320 | 99.320 |
| 25th percentile | 66.870 | 48.870 | 66.870 | 66.870 | 66.870 | 66.870 | 99.320 | 99.320 |
| 75th percentile | 99.320 | 99.320 | 99.320 | 99.320 | 99.320 | 99.320 | 99.320 | 99.320 |
| Minimum | 2.780 | 2.780 | 2.780 | 2.780 | 2.780 | 2.780 | 24.910 | 99.320 |
| Maximum | 99.320 | 99.320 | 99.320 | 99.320 | 99.320 | 99.320 | 99.320 | 99.320 |
| MH   Mean | 71.883 | 64.434 | 67.418 | 72.592 | 74.781 | 79.086 | 79.479 | 91.794 |
| Standard deviation | 21.100 | 22.099 | 21.817 | 19.445 | 18.459 | 21.659 | 16.532 | 8.127 |
| Median | 74.865 | 65.795 | 69.490 | 74.865 | 74.865 | 83.670 | 83.670 | 92.650 |
| 25th percentile | 59.850 | 49.545 | 51.325 | 59.850 | 65.505 | 69.490 | 65.505 | 92.650 |
| 75th percentile | 83.845 | 83.670 | 83.670 | 89.615 | 89.615 | 92.650 | 92.650 | 92.650 |
| Minimum | -1.030 | -1.030 | -1.030 | 6.005 | -1.030 | 6.005 | 30.945 | 83.670 |
| Maximum | 98.595 | 98.595 | 98.595 | 98.595 | 98.595 | 98.595 | 98.595 | 98.595 |
| RE     Mean | 78.627 | 69.709 | 76.484 | 82.709 | 79.917 | 81.924 | 87.264 | 94.081 |
| Standard deviation | 34.757 | 36.497 | 35.730 | 32.659 | 34.935 | 34.152 | 27.368 | 21.237 |
| Median | 104.125 | 77.005 | 81.810 | 104.125 | 104.125 | 104.125 | 104.125 | 104.125 |
| 25th percentile | 54.690 | 54.690 | 54.690 | 54.690 | 54.690 | 68.370 | 77.005 | 81.810 |
| 75th percentile | 104.125 | 104.125 | 104.125 | 104.125 | 104.125 | 104.125 | 104.125 | 104.125 |
| Minimum | -33.415 | -33.415 | -33.415 | -33.415 | -33.415 | 2.250 | 2.250 | 77.005 |
| Maximum | 104.125 | 104.125 | 104.125 | 104.125 | 104.125 | 104.125 | 104.125 | 104.125 |

BP, bodily pain; GH, general health; MCS, mental component summary; MH, mental health; PCS, physical component summary; PF, physical functioning; RE, role emotional; RP, role physical; VT, vitality; SF, social functioning

**SM3 Table 17**. Canadian norms for VR-12 health utility values, summary component scores, and domain scores, by age group – Quebec, males only.

|  | All participants (n=749) | 18 to 29 (n=139) | 30 to 39 (n=141) | 40 to 49 (n=154) | 50 to 59 (n=184) | 60 to 69 (n=71) | 70 to 79 (n=56) | 80 and over (n=4) |
| --- | --- | --- | --- | --- | --- | --- | --- | --- |
| *VR-12 health utility values* |  |  |  |  |  |  |  |  |
| Mean | 0.755 | 0.721 | 0.734 | 0.768 | 0.760 | 0.772 | 0.809 | - |
| Standard deviation | 0.176 | 0.176 | 0.190 | 0.167 | 0.180 | 0.179 | 0.117 | - |
| Median | 0.789 | 0.765 | 0.765 | 0.818 | 0.799 | 0.800 | 0.839 | - |
| 25th percentile | 0.696 | 0.645 | 0.696 | 0.698 | 0.716 | 0.697 | 0.738 | - |
| 75th percentile | 0.860 | 0.832 | 0.818 | 0.873 | 0.860 | 0.883 | 0.891 | - |
| Minimum | -0.489 | -0.065 | -0.257 | -0.070 | -0.489 | 0.061 | 0.388 | - |
| Maximum | 1.000 | 1.000 | 1.000 | 1.000 | 1.000 | 0.952 | 1.000 | - |
| *VR-12 summary component score* |  |  |  |  |  |  |  |  |
| PCS   Mean | 52.186 | 54.474 | 53.752 | 53.081 | 50.910 | 47.760 | 50.097 | - |
| Standard deviation | 8.315 | 6.258 | 7.349 | 6.610 | 9.536 | 10.658 | 8.164 | - |
| Median | 54.480 | 55.818 | 55.582 | 55.921 | 54.546 | 50.911 | 51.427 | - |
| 25th percentile | 48.856 | 51.919 | 50.687 | 49.722 | 47.909 | 42.297 | 48.406 | - |
| 75th percentile | 57.729 | 58.016 | 58.241 | 57.425 | 57.573 | 54.838 | 55.164 | - |
| Minimum | 13.059 | 28.941 | 21.086 | 26.504 | 13.059 | 16.988 | 24.303 | - |
| Maximum | 69.530 | 69.530 | 64.902 | 61.264 | 64.573 | 63.207 | 63.575 | - |
| MCS  Mean | 51.829 | 47.859 | 49.722 | 52.298 | 53.169 | 55.143 | 56.682 | - |
| Standard deviation | 9.050 | 9.761 | 9.440 | 9.107 | 7.268 | 8.961 | 5.360 | - |
| Median | 53.846 | 50.051 | 50.081 | 54.733 | 53.858 | 57.393 | 58.521 | - |
| 25th percentile | 47.260 | 39.924 | 45.134 | 48.138 | 49.473 | 50.790 | 54.285 | - |
| 75th percentile | 58.376 | 55.399 | 56.230 | 59.588 | 58.381 | 61.403 | 60.801 | - |
| Minimum | 15.291 | 20.563 | 17.082 | 15.291 | 18.311 | 31.149 | 37.173 | - |
| Maximum | 67.113 | 62.699 | 64.280 | 67.113 | 65.389 | 64.291 | 65.639 | - |
| *VR-12 domain scores* |  |  |  |  |  |  |  |  |
| PF     Mean | 83.233 | 86.205 | 85.594 | 86.491 | 81.509 | 73.234 | 80.607 | - |
| Standard deviation | 20.320 | 15.588 | 19.362 | 15.338 | 21.825 | 29.588 | 20.458 | - |
| Median | 93.485 | 93.485 | 93.485 | 93.485 | 93.485 | 80.375 | 93.485 | - |
| 25th percentile | 80.375 | 80.375 | 80.375 | 93.485 | 80.375 | 58.290 | 80.375 | - |
| 75th percentile | 93.485 | 93.485 | 93.485 | 93.485 | 93.485 | 93.485 | 93.485 | - |
| Minimum | 7.765 | 7.765 | 7.765 | 7.765 | 7.765 | 7.765 | 7.765 | - |
| Maximum | 93.485 | 93.485 | 93.485 | 93.485 | 93.485 | 93.485 | 93.485 | - |
| RP     Mean | 80.175 | 83.593 | 82.215 | 82.579 | 78.154 | 71.230 | 78.325 | - |
| Standard deviation | 33.230 | 26.736 | 34.496 | 29.136 | 36.389 | 41.244 | 31.086 | - |
| Median | 100.690 | 100.690 | 100.690 | 100.690 | 100.690 | 89.670 | 90.425 | - |
| 25th percentile | 79.405 | 79.405 | 79.405 | 79.405 | 79.405 | 44.460 | 79.405 | - |
| 75th percentile | 100.690 | 100.690 | 100.690 | 100.690 | 100.690 | 100.690 | 100.690 | - |
| Minimum | -3.670 | -3.645 | 0.465 | -3.670 | -3.670 | 0.490 | 0.490 | - |
| Maximum | 100.690 | 100.690 | 100.690 | 100.690 | 100.690 | 100.690 | 100.690 | - |
| BP     Mean | 75.023 | 74.496 | 76.038 | 77.898 | 72.102 | 71.195 | 79.265 | - |
| Standard deviation | 24.108 | 24.932 | 25.080 | 23.473 | 23.397 | 24.535 | 21.456 | - |
| Median | 95.200 | 95.200 | 95.200 | 95.200 | 56.250 | 56.250 | 95.200 | - |
| 25th percentile | 56.250 | 56.250 | 56.250 | 56.250 | 56.250 | 56.250 | 56.250 | - |
| 75th percentile | 95.200 | 95.200 | 95.200 | 95.200 | 95.200 | 95.200 | 95.200 | - |
| Minimum | 2.280 | 2.280 | 2.280 | 2.280 | 2.280 | 31.500 | 31.500 | - |
| Maximum | 95.200 | 95.200 | 95.200 | 95.200 | 95.200 | 95.200 | 95.200 | - |
| GH    Mean | 77.341 | 80.782 | 79.937 | 79.786 | 75.652 | 67.806 | 73.026 | - |
| Standard deviation | 19.514 | 20.291 | 17.762 | 17.308 | 18.747 | 24.368 | 17.613 | - |
| Median | 83.710 | 83.710 | 83.710 | 83.710 | 83.710 | 83.710 | 83.710 | - |
| 25th percentile | 61.500 | 83.710 | 61.500 | 83.710 | 61.500 | 61.500 | 61.500 | - |
| 75th percentile | 83.710 | 83.710 | 83.710 | 83.710 | 83.710 | 83.710 | 83.710 | - |
| Minimum | 0.580 | 0.580 | 0.580 | 0.580 | 0.580 | 0.580 | 0.580 | - |
| Maximum | 101.840 | 101.840 | 101.840 | 101.840 | 101.840 | 101.840 | 101.840 | - |
| VT     Mean | 61.664 | 60.979 | 58.689 | 62.106 | 63.651 | 61.940 | 64.722 | - |
| Standard deviation | 21.075 | 21.136 | 22.866 | 18.569 | 20.620 | 25.350 | 18.037 | - |
| Median | 54.550 | 54.550 | 54.550 | 54.550 | 54.550 | 79.250 | 79.250 | - |
| 25th percentile | 45.900 | 45.900 | 45.900 | 45.900 | 45.900 | 45.900 | 45.900 | - |
| 75th percentile | 79.250 | 79.250 | 79.250 | 79.250 | 79.250 | 79.250 | 79.250 | - |
| Minimum | 1.800 | 1.800 | 1.800 | 1.800 | 1.800 | 1.800 | 31.930 | - |
| Maximum | 95.340 | 95.340 | 95.340 | 95.340 | 95.340 | 95.340 | 95.340 | - |
| SF      Mean | 79.574 | 72.287 | 74.823 | 80.470 | 81.306 | 85.242 | 93.207 | - |
| Standard deviation | 26.238 | 30.254 | 28.085 | 25.891 | 22.750 | 24.186 | 14.421 | - |
| Median | 99.320 | 66.870 | 66.870 | 99.320 | 99.320 | 99.320 | 99.320 | - |
| 25th percentile | 66.870 | 48.870 | 66.870 | 66.870 | 66.870 | 66.870 | 99.320 | - |
| 75th percentile | 99.320 | 99.320 | 99.320 | 99.320 | 99.320 | 99.320 | 99.320 | - |
| Minimum | 2.780 | 2.780 | 2.780 | 2.780 | 2.780 | 2.780 | 48.870 | - |
| Maximum | 99.320 | 99.320 | 99.320 | 99.320 | 99.320 | 99.320 | 99.320 | - |
| MH   Mean | 74.710 | 67.282 | 70.892 | 75.896 | 77.609 | 79.727 | 82.468 | - |
| Standard deviation | 19.524 | 21.751 | 20.599 | 18.707 | 15.818 | 20.513 | 14.281 | - |
| Median | 83.670 | 69.490 | 74.865 | 83.670 | 83.670 | 83.670 | 83.845 | - |
| 25th percentile | 60.685 | 49.110 | 55.055 | 65.505 | 69.490 | 69.490 | 83.670 | - |
| 75th percentile | 92.650 | 83.670 | 83.670 | 92.650 | 92.650 | 92.650 | 92.650 | - |
| Minimum | -1.030 | 14.025 | -1.030 | 6.005 | -1.030 | 30.945 | 31.800 | - |
| Maximum | 98.595 | 98.595 | 98.595 | 98.595 | 98.595 | 98.595 | 98.595 | - |
| RE     Mean | 81.251 | 70.639 | 78.890 | 85.025 | 82.415 | 85.337 | 93.948 | - |
| Standard deviation | 33.388 | 35.846 | 35.265 | 32.323 | 32.920 | 32.001 | 19.363 | - |
| Median | 104.125 | 81.810 | 104.125 | 104.125 | 104.125 | 104.125 | 104.125 | - |
| 25th percentile | 54.690 | 54.690 | 54.690 | 81.810 | 54.690 | 77.005 | 81.810 | - |
| 75th percentile | 104.125 | 104.125 | 104.125 | 104.125 | 104.125 | 104.125 | 104.125 | - |
| Minimum | -33.415 | -33.415 | -33.415 | -33.415 | -33.415 | 2.250 | 26.690 | - |
| Maximum | 104.125 | 104.125 | 104.125 | 104.125 | 104.125 | 104.125 | 104.125 | - |

BP, bodily pain; GH, general health; MCS, mental component summary; MH, mental health; PCS, physical component summary; PF, physical functioning; RE, role emotional; RP, role physical; VT, vitality; SF, social functioning

**SM3 Table 18**. Canadian norms for VR-12 health utility values, summary component scores, and domain scores, by age group – Quebec, females only.

|  | All participants (n=632) | 18 to 29 (n=140) | 30 to 39 (n=125) | 40 to 49 (n=131) | 50 to 59 (n=118) | 60 to 69 (n=77) | 70 to 79 (n=40) | 80 and over (n=1) |
| --- | --- | --- | --- | --- | --- | --- | --- | --- |
| *VR-12 health utility values* |  |  |  |  |  |  |  |  |
| Mean | 0.714 | 0.693 | 0.699 | 0.732 | 0.695 | 0.762 | 0.693 | - |
| Standard deviation | 0.201 | 0.182 | 0.193 | 0.170 | 0.246 | 0.199 | 0.225 | - |
| Median | 0.749 | 0.730 | 0.746 | 0.765 | 0.759 | 0.792 | 0.768 | - |
| 25th percentile | 0.644 | 0.608 | 0.640 | 0.674 | 0.644 | 0.688 | 0.644 | - |
| 75th percentile | 0.832 | 0.799 | 0.812 | 0.852 | 0.839 | 0.879 | 0.839 | - |
| Minimum | -0.383 | -0.139 | -0.383 | -0.305 | -0.161 | 0.256 | 0.203 | - |
| Maximum | 1.000 | 1.000 | 1.000 | 1.000 | 1.000 | 1.000 | 0.952 | - |
| *VR-12 summary component score* |  |  |  |  |  |  |  |  |
| PCS   Mean | 50.996 | 55.473 | 54.283 | 52.134 | 47.704 | 47.825 | 41.398 | - |
| Standard deviation | 10.447 | 6.790 | 7.454 | 8.576 | 12.579 | 12.051 | 10.574 | - |
| Median | 54.229 | 57.675 | 56.320 | 53.855 | 52.562 | 48.771 | 39.797 | - |
| 25th percentile | 46.076 | 51.924 | 52.151 | 47.413 | 40.583 | 39.001 | 35.360 | - |
| 75th percentile | 57.872 | 58.897 | 58.603 | 58.594 | 56.824 | 56.315 | 47.028 | - |
| Minimum | 13.883 | 20.035 | 26.447 | 16.904 | 13.883 | 21.265 | 21.969 | - |
| Maximum | 70.042 | 70.042 | 65.605 | 65.783 | 62.626 | 62.465 | 61.051 | - |
| MCS  Mean | 49.840 | 45.961 | 47.052 | 50.301 | 51.457 | 53.956 | 53.685 | - |
| Standard deviation | 9.784 | 10.326 | 9.780 | 8.000 | 9.096 | 9.259 | 7.657 | - |
| Median | 51.621 | 48.473 | 47.656 | 52.055 | 53.812 | 56.480 | 55.526 | - |
| 25th percentile | 44.882 | 39.518 | 41.247 | 45.072 | 46.301 | 49.002 | 49.741 | - |
| 75th percentile | 56.980 | 52.450 | 52.994 | 56.968 | 57.184 | 59.763 | 58.396 | - |
| Minimum | 8.848 | 8.848 | 17.620 | 24.013 | 28.148 | 30.455 | 38.802 | - |
| Maximum | 67.832 | 62.699 | 66.386 | 62.699 | 67.832 | 63.618 | 65.941 | - |
| *VR-12 domain scores* |  |  |  |  |  |  |  |  |
| PF     Mean | 79.244 | 84.567 | 85.177 | 82.764 | 73.535 | 74.656 | 61.748 | - |
| Standard deviation | 24.810 | 15.974 | 17.196 | 20.388 | 31.962 | 29.916 | 32.450 | - |
| Median | 93.485 | 93.485 | 93.485 | 93.485 | 93.485 | 80.375 | 80.375 | - |
| 25th percentile | 80.375 | 80.375 | 80.375 | 80.375 | 58.290 | 58.290 | 29.460 | - |
| 75th percentile | 93.485 | 93.485 | 93.485 | 93.485 | 93.485 | 93.485 | 80.375 | - |
| Minimum | 7.765 | 7.765 | 7.765 | 7.765 | 7.765 | 7.765 | 7.765 | - |
| Maximum | 93.485 | 93.485 | 93.485 | 93.485 | 93.485 | 93.485 | 93.485 | - |
| RP     Mean | 75.144 | 88.257 | 79.625 | 79.548 | 67.957 | 68.593 | 45.823 | - |
| Standard deviation | 38.640 | 24.712 | 31.784 | 32.615 | 43.106 | 51.055 | 48.344 | - |
| Median | 90.425 | 100.690 | 89.670 | 100.690 | 89.670 | 90.425 | 9.775 | - |
| 25th percentile | 54.725 | 79.405 | 79.405 | 79.405 | 40.165 | 39.705 | 9.775 | - |
| 75th percentile | 100.690 | 100.690 | 100.690 | 100.690 | 100.690 | 100.690 | 100.690 | - |
| Minimum | -3.670 | -3.670 | -3.670 | -3.670 | -3.670 | 0.490 | 0.490 | - |
| Maximum | 100.690 | 100.690 | 100.690 | 100.690 | 100.690 | 100.690 | 100.690 | - |
| BP     Mean | 72.074 | 79.986 | 78.114 | 72.201 | 64.324 | 70.016 | 57.237 | - |
| Standard deviation | 27.298 | 23.300 | 24.238 | 24.959 | 30.857 | 30.634 | 23.583 | - |
| Median | 95.200 | 95.200 | 95.200 | 56.250 | 56.250 | 56.250 | 56.250 | - |
| 25th percentile | 56.250 | 56.250 | 56.250 | 56.250 | 43.890 | 43.890 | 43.890 | - |
| 75th percentile | 95.200 | 95.200 | 95.200 | 95.200 | 95.200 | 95.200 | 56.250 | - |
| Minimum | 2.280 | 2.280 | 2.280 | 2.280 | 2.280 | 31.500 | 31.500 | - |
| Maximum | 95.200 | 95.200 | 95.200 | 95.200 | 95.200 | 95.200 | 95.200 | - |
| GH    Mean | 76.167 | 83.226 | 80.379 | 77.736 | 71.409 | 70.945 | 63.041 | - |
| Standard deviation | 21.071 | 16.500 | 18.058 | 19.010 | 24.111 | 24.070 | 21.071 | - |
| Median | 83.710 | 83.710 | 83.710 | 83.710 | 83.710 | 83.710 | 61.500 | - |
| 25th percentile | 61.500 | 83.710 | 61.500 | 61.500 | 61.500 | 61.500 | 37.500 | - |
| 75th percentile | 83.710 | 101.840 | 83.710 | 83.710 | 83.710 | 83.710 | 83.710 | - |
| Minimum | 0.580 | 0.580 | 0.580 | 0.580 | 0.580 | 37.500 | 37.500 | - |
| Maximum | 101.840 | 101.840 | 101.840 | 101.840 | 101.840 | 101.840 | 101.840 | - |
| VT     Mean | 56.374 | 52.653 | 54.512 | 59.640 | 56.367 | 62.654 | 48.962 | - |
| Standard deviation | 23.236 | 25.429 | 22.315 | 19.643 | 20.993 | 29.075 | 17.175 | - |
| Median | 54.550 | 54.550 | 54.550 | 54.550 | 54.550 | 54.550 | 54.550 | - |
| 25th percentile | 45.900 | 31.930 | 45.900 | 45.900 | 45.900 | 45.900 | 31.930 | - |
| 75th percentile | 79.250 | 79.250 | 79.250 | 79.250 | 79.250 | 79.250 | 54.550 | - |
| Minimum | 1.800 | 1.800 | 1.800 | 1.800 | 1.800 | 1.800 | 31.930 | - |
| Maximum | 95.340 | 95.340 | 95.340 | 95.340 | 95.340 | 95.340 | 79.250 | - |
| SF      Mean | 76.724 | 69.224 | 72.192 | 79.037 | 79.185 | 83.561 | 82.227 | - |
| Standard deviation | 28.354 | 30.668 | 28.104 | 24.978 | 26.578 | 27.950 | 29.429 | - |
| Median | 99.320 | 66.870 | 66.870 | 99.320 | 99.320 | 99.320 | 99.320 | - |
| 25th percentile | 66.870 | 48.870 | 48.870 | 66.870 | 66.870 | 66.870 | 66.870 | - |
| 75th percentile | 99.320 | 99.320 | 99.320 | 99.320 | 99.320 | 99.320 | 99.320 | - |
| Minimum | 2.780 | 2.780 | 2.780 | 2.780 | 2.780 | 24.910 | 24.910 | - |
| Maximum | 99.320 | 99.320 | 99.320 | 99.320 | 99.320 | 99.320 | 99.320 | - |
| MH   Mean | 69.043 | 62.595 | 63.307 | 68.854 | 71.033 | 78.638 | 76.166 | - |
| Standard deviation | 22.168 | 21.491 | 22.394 | 19.666 | 21.359 | 22.781 | 18.765 | - |
| Median | 74.865 | 65.505 | 65.505 | 69.490 | 74.865 | 83.670 | 83.670 | - |
| 25th percentile | 51.325 | 49.545 | 51.325 | 51.325 | 60.685 | 69.490 | 65.505 | - |
| 75th percentile | 83.670 | 74.865 | 74.865 | 83.670 | 83.670 | 92.650 | 92.650 | - |
| Minimum | -1.030 | -1.030 | -1.030 | 14.025 | 16.745 | 6.005 | 30.945 | - |
| Maximum | 98.595 | 98.595 | 98.595 | 98.595 | 98.595 | 98.595 | 98.595 | - |
| RE     Mean | 76.116 | 70.155 | 73.636 | 80.089 | 76.607 | 79.534 | 79.859 | - |
| Standard deviation | 35.787 | 36.202 | 36.147 | 32.971 | 37.711 | 35.924 | 34.147 | - |
| Median | 81.810 | 77.005 | 81.810 | 104.125 | 104.125 | 81.810 | 104.125 | - |
| 25th percentile | 54.690 | 54.690 | 42.775 | 54.690 | 54.690 | 54.690 | 41.250 | - |
| 75th percentile | 104.125 | 104.125 | 104.125 | 104.125 | 104.125 | 104.125 | 104.125 | - |
| Minimum | -33.415 | -33.415 | -33.415 | -27.180 | -33.415 | 2.250 | 2.250 | - |
| Maximum | 104.125 | 104.125 | 104.125 | 104.125 | 104.125 | 104.125 | 104.125 | - |

BP, bodily pain; GH, general health; MCS, mental component summary; MH, mental health; PCS, physical component summary; PF, physical functioning; RE, role emotional; RP, role physical; VT, vitality; SF, social functioning

**SM3 Table 19**. Canadian norms for VR-12 health utility values, summary component scores, and domain scores, by age group – Nova Scotia.

|  | All participants (n=239) | 18 to 29 (n=29) | 30 to 39 (n=40) | 40 to 49 (n=40) | 50 to 59 (n=52) | 60 to 69 (n=36) | 70 to 79 (n=37) | 80 and over (n=5) |
| --- | --- | --- | --- | --- | --- | --- | --- | --- |
| *VR-12 health utility values* |  |  |  |  |  |  |  |  |
| Mean | 0.692 | 0.662 | 0.729 | 0.669 | 0.736 | 0.697 | 0.681 | 0.556 |
| Standard deviation | 0.196 | 0.199 | 0.127 | 0.214 | 0.170 | 0.231 | 0.207 | 0.291 |
| Median | 0.750 | 0.736 | 0.769 | 0.778 | 0.791 | 0.678 | 0.777 | 0.630 |
| 25th percentile | 0.615 | 0.564 | 0.615 | 0.644 | 0.706 | 0.630 | 0.644 | 0.388 |
| 75th percentile | 0.839 | 0.837 | 0.839 | 0.818 | 0.852 | 0.860 | 0.842 | 0.717 |
| Minimum | -0.590 | 0.127 | -0.174 | -0.590 | -0.093 | -0.177 | -0.080 | 0.308 |
| Maximum | 1.000 | 0.952 | 1.000 | 0.919 | 0.913 | 1.000 | 0.942 | 0.737 |
| *VR-12 summary component score* |  |  |  |  |  |  |  |  |
| PCS   Mean | 47.280 | 51.580 | 50.572 | 51.632 | 48.414 | 46.002 | 41.656 | 33.176 |
| Standard deviation | 10.837 | 8.513 | 7.499 | 7.574 | 9.593 | 13.292 | 11.315 | 17.488 |
| Median | 51.439 | 52.939 | 51.990 | 54.745 | 48.362 | 51.535 | 43.793 | 30.624 |
| 25th percentile | 41.400 | 48.075 | 47.281 | 51.039 | 41.756 | 32.956 | 33.071 | 30.624 |
| 75th percentile | 56.824 | 57.299 | 56.824 | 57.077 | 57.908 | 56.707 | 52.814 | 42.177 |
| Minimum | 7.468 | 21.206 | 19.643 | 17.412 | 16.738 | 18.292 | 7.468 | 18.755 |
| Maximum | 64.464 | 64.464 | 62.387 | 60.576 | 59.788 | 59.022 | 59.756 | 49.636 |
| MCS  Mean | 51.342 | 45.712 | 50.061 | 47.110 | 54.625 | 50.575 | 56.405 | 54.881 |
| Standard deviation | 9.047 | 10.547 | 6.618 | 8.546 | 8.850 | 9.028 | 5.418 | 17.789 |
| Median | 54.576 | 46.033 | 52.738 | 48.659 | 56.980 | 52.872 | 57.213 | 57.198 |
| 25th percentile | 43.958 | 37.618 | 43.410 | 43.120 | 49.136 | 43.520 | 55.131 | 53.343 |
| 75th percentile | 57.471 | 57.471 | 56.466 | 56.466 | 59.588 | 55.676 | 60.246 | 62.815 |
| Minimum | 18.705 | 19.036 | 29.279 | 18.705 | 30.367 | 20.102 | 30.533 | 34.123 |
| Maximum | 68.956 | 61.487 | 62.711 | 60.929 | 67.355 | 65.767 | 68.956 | 62.815 |
| *VR-12 domain scores* |  |  |  |  |  |  |  |  |
| PF     Mean | 68.830 | 80.704 | 79.249 | 77.327 | 64.318 | 65.861 | 59.641 | 48.360 |
| Standard deviation | 28.379 | 18.071 | 23.046 | 20.398 | 33.956 | 33.677 | 25.926 | 36.730 |
| Median | 80.375 | 93.485 | 93.485 | 93.485 | 80.375 | 80.375 | 58.290 | 58.290 |
| 25th percentile | 58.290 | 58.290 | 80.375 | 58.290 | 29.460 | 29.460 | 29.460 | 58.290 |
| 75th percentile | 93.485 | 93.485 | 93.485 | 93.485 | 93.485 | 93.485 | 93.485 | 58.290 |
| Minimum | 7.765 | 7.765 | 7.765 | 7.765 | 7.765 | 7.765 | 7.765 | 7.765 |
| Maximum | 93.485 | 93.485 | 93.485 | 93.485 | 93.485 | 93.485 | 93.485 | 58.290 |
| RP     Mean | 72.683 | 68.287 | 79.961 | 82.507 | 82.259 | 64.950 | 63.622 | 52.709 |
| Standard deviation | 34.810 | 34.638 | 28.718 | 25.422 | 32.553 | 44.754 | 32.336 | 65.710 |
| Median | 90.425 | 89.670 | 100.690 | 100.690 | 100.690 | 79.405 | 79.405 | 79.405 |
| 25th percentile | 49.970 | 49.970 | 79.405 | 79.405 | 79.405 | 9.775 | 9.775 | 9.775 |
| 75th percentile | 100.690 | 100.690 | 100.690 | 100.690 | 100.690 | 100.690 | 100.690 | 79.405 |
| Minimum | -3.670 | -3.670 | -3.670 | -3.670 | -3.670 | -3.670 | -3.670 | 0.490 |
| Maximum | 100.690 | 100.690 | 100.690 | 100.690 | 100.690 | 100.690 | 100.690 | 79.405 |
| BP     Mean | 70.053 | 73.763 | 67.308 | 70.707 | 75.790 | 69.588 | 72.751 | 43.252 |
| Standard deviation | 24.270 | 23.500 | 18.931 | 20.755 | 22.515 | 30.032 | 22.388 | 40.365 |
| Median | 56.250 | 95.200 | 56.250 | 56.250 | 95.200 | 95.200 | 95.200 | 31.500 |
| 25th percentile | 56.250 | 56.250 | 43.890 | 56.250 | 56.250 | 43.890 | 56.250 | 31.500 |
| 75th percentile | 95.200 | 95.200 | 95.200 | 95.200 | 95.200 | 95.200 | 95.200 | 56.250 |
| Minimum | 2.280 | 31.500 | 2.280 | 2.280 | 2.280 | 2.280 | 2.280 | 31.500 |
| Maximum | 95.200 | 95.200 | 95.200 | 95.200 | 95.200 | 95.200 | 95.200 | 95.200 |
| GH    Mean | 68.607 | 69.881 | 76.289 | 73.834 | 74.034 | 63.164 | 62.983 | 46.800 |
| Standard deviation | 20.142 | 22.544 | 14.297 | 12.572 | 16.113 | 27.060 | 19.428 | 30.332 |
| Median | 83.710 | 83.710 | 83.710 | 83.710 | 83.710 | 61.500 | 61.500 | 37.500 |
| 25th percentile | 61.500 | 61.500 | 61.500 | 61.500 | 61.500 | 37.500 | 37.500 | 37.500 |
| 75th percentile | 83.710 | 83.710 | 83.710 | 83.710 | 83.710 | 83.710 | 83.710 | 61.500 |
| Minimum | 0.580 | 0.580 | 0.580 | 37.500 | 0.580 | 0.580 | 0.580 | 37.500 |
| Maximum | 101.840 | 101.840 | 101.840 | 101.840 | 83.710 | 101.840 | 101.840 | 83.710 |
| VT     Mean | 53.998 | 62.101 | 54.846 | 47.034 | 63.976 | 50.360 | 45.939 | 37.235 |
| Standard deviation | 22.052 | 24.264 | 15.806 | 13.673 | 21.473 | 24.973 | 23.273 | 30.926 |
| Median | 45.900 | 45.900 | 54.550 | 45.900 | 79.250 | 45.900 | 45.900 | 45.900 |
| 25th percentile | 45.900 | 45.900 | 45.900 | 31.930 | 54.550 | 31.930 | 31.930 | 31.930 |
| 75th percentile | 79.250 | 95.340 | 79.250 | 54.550 | 79.250 | 79.250 | 79.250 | 45.900 |
| Minimum | 1.800 | 31.930 | 1.800 | 1.800 | 1.800 | 1.800 | 1.800 | 1.800 |
| Maximum | 95.340 | 95.340 | 95.340 | 79.250 | 79.250 | 95.340 | 95.340 | 54.550 |
| SF      Mean | 75.444 | 58.016 | 73.120 | 73.489 | 80.486 | 76.438 | 83.176 | 84.838 |
| Standard deviation | 26.022 | 27.191 | 25.014 | 26.051 | 26.637 | 24.727 | 19.902 | 36.866 |
| Median | 99.320 | 66.870 | 66.870 | 99.320 | 99.320 | 66.870 | 99.320 | 99.320 |
| 25th percentile | 48.870 | 24.910 | 48.870 | 48.870 | 48.870 | 48.870 | 48.870 | 66.870 |
| 75th percentile | 99.320 | 66.870 | 99.320 | 99.320 | 99.320 | 99.320 | 99.320 | 99.320 |
| Minimum | 2.780 | 2.780 | 2.780 | 2.780 | 2.780 | 24.910 | 24.910 | 48.870 |
| Maximum | 99.320 | 99.320 | 99.320 | 99.320 | 99.320 | 99.320 | 99.320 | 99.320 |
| MH   Mean | 72.567 | 62.974 | 70.301 | 63.822 | 79.251 | 72.266 | 80.154 | 76.434 |
| Standard deviation | 19.109 | 20.564 | 14.597 | 20.955 | 17.083 | 19.388 | 14.582 | 37.974 |
| Median | 74.865 | 60.685 | 74.865 | 65.505 | 83.670 | 74.865 | 83.670 | 83.670 |
| 25th percentile | 60.685 | 51.325 | 51.325 | 51.325 | 74.865 | 55.620 | 74.865 | 74.865 |
| 75th percentile | 83.845 | 83.670 | 83.670 | 83.845 | 92.650 | 83.845 | 92.650 | 92.650 |
| Minimum | -1.030 | 21.060 | 23.910 | -1.030 | 16.745 | 14.025 | 6.005 | 31.800 |
| Maximum | 98.595 | 98.595 | 98.595 | 98.595 | 98.595 | 98.595 | 98.595 | 92.650 |
| RE     Mean | 76.743 | 63.170 | 79.575 | 71.283 | 84.125 | 69.007 | 95.576 | 72.260 |
| Standard deviation | 33.125 | 39.933 | 26.016 | 29.870 | 30.303 | 43.334 | 18.880 | 51.317 |
| Median | 104.125 | 54.690 | 104.125 | 81.810 | 104.125 | 81.810 | 104.125 | 77.005 |
| 25th percentile | 54.690 | 41.250 | 54.690 | 41.250 | 68.370 | 29.335 | 104.125 | 54.690 |
| 75th percentile | 104.125 | 104.125 | 104.125 | 104.125 | 104.125 | 104.125 | 104.125 | 104.125 |
| Minimum | -33.415 | -13.835 | -33.415 | -33.415 | -13.835 | -20.070 | 2.250 | 29.335 |
| Maximum | 104.125 | 104.125 | 104.125 | 104.125 | 104.125 | 104.125 | 104.125 | 104.125 |

BP, bodily pain; GH, general health; MCS, mental component summary; MH, mental health; PCS, physical component summary; PF, physical functioning; RE, role emotional; RP, role physical; VT, vitality; SF, social functioning

**SM3 Table 20**. Canadian norms for VR-12 health utility values, summary component scores, and domain scores, by age group – Nova Scotia, males only.

|  | All participants (n=116) | 18 to 29 (n=13) | 30 to 39 (n=20) | 40 to 49 (n=17) | 50 to 59 (n=26) | 60 to 69 (n=14) | 70 to 79 (n=23) | 80 and over (n=3) |
| --- | --- | --- | --- | --- | --- | --- | --- | --- |
| *VR-12 health utility values* |  |  |  |  |  |  |  |  |
| Mean | 0.695 | 0.741 | 0.704 | 0.788 | 0.703 | 0.656 | 0.716 | - |
| Standard deviation | 0.178 | 0.157 | 0.140 | 0.103 | 0.165 | 0.204 | 0.191 | - |
| Median | 0.746 | 0.818 | 0.768 | 0.818 | 0.746 | 0.644 | 0.808 | - |
| 25th percentile | 0.605 | 0.564 | 0.605 | 0.736 | 0.644 | 0.516 | 0.457 | - |
| 75th percentile | 0.837 | 0.837 | 0.839 | 0.839 | 0.839 | 0.879 | 0.863 | - |
| Minimum | -0.174 | 0.344 | -0.174 | 0.267 | -0.043 | 0.212 | 0.014 | - |
| Maximum | 0.952 | 0.952 | 0.852 | 0.913 | 0.913 | 0.923 | 0.942 | - |
| *VR-12 summary component score* |  |  |  |  |  |  |  |  |
| PCS   Mean | 46.027 | 53.234 | 48.987 | 54.230 | 47.908 | 39.910 | 43.522 | - |
| Standard deviation | 11.411 | 6.812 | 7.906 | 5.774 | 8.444 | 12.503 | 11.233 | - |
| Median | 49.763 | 52.939 | 50.737 | 56.096 | 48.362 | 33.375 | 48.063 | - |
| 25th percentile | 35.868 | 52.939 | 43.676 | 52.814 | 45.746 | 32.956 | 32.837 | - |
| 75th percentile | 55.563 | 57.155 | 55.921 | 57.077 | 56.824 | 51.493 | 53.423 | - |
| Minimum | 18.462 | 21.206 | 22.622 | 26.998 | 18.462 | 24.370 | 21.165 | - |
| Maximum | 62.098 | 62.098 | 58.491 | 60.214 | 59.788 | 56.468 | 59.756 | - |
| MCS  Mean | 51.728 | 51.117 | 49.865 | 51.330 | 50.745 | 48.719 | 57.162 | - |
| Standard deviation | 8.253 | 8.964 | 7.627 | 7.001 | 7.411 | 8.148 | 5.574 | - |
| Median | 55.254 | 55.768 | 55.497 | 51.307 | 51.877 | 47.569 | 60.051 | - |
| 25th percentile | 43.774 | 41.878 | 43.039 | 47.740 | 42.361 | 40.250 | 55.131 | - |
| 75th percentile | 58.239 | 57.471 | 56.466 | 56.466 | 58.622 | 55.254 | 60.246 | - |
| Minimum | 20.766 | 31.724 | 29.279 | 20.766 | 30.367 | 40.250 | 30.533 | - |
| Maximum | 63.429 | 61.487 | 59.559 | 60.929 | 63.429 | 59.608 | 62.516 | - |
| *VR-12 domain scores* |  |  |  |  |  |  |  |  |
| PF     Mean | 70.141 | 84.462 | 76.320 | 88.384 | 73.793 | 52.757 | 63.111 | - |
| Standard deviation | 28.475 | 20.628 | 28.603 | 12.367 | 21.233 | 34.643 | 28.074 | - |
| Median | 80.375 | 93.485 | 93.485 | 93.485 | 80.375 | 58.290 | 80.375 | - |
| 25th percentile | 58.290 | 80.375 | 80.375 | 93.485 | 58.290 | 29.460 | 29.460 | - |
| 75th percentile | 93.485 | 93.485 | 93.485 | 93.485 | 93.485 | 93.485 | 93.485 | - |
| Minimum | 7.765 | 7.765 | 7.765 | 29.460 | 7.765 | 7.765 | 7.765 | - |
| Maximum | 93.485 | 93.485 | 93.485 | 93.485 | 93.485 | 93.485 | 93.485 | - |
| RP     Mean | 67.887 | 75.396 | 74.353 | 89.627 | 74.675 | 47.312 | 64.811 | - |
| Standard deviation | 37.311 | 33.174 | 36.360 | 22.158 | 30.392 | 46.298 | 35.049 | - |
| Median | 79.405 | 100.690 | 100.690 | 100.690 | 90.425 | 44.720 | 79.405 | - |
| 25th percentile | 44.720 | 49.970 | 44.720 | 100.690 | 79.405 | 5.020 | 5.020 | - |
| 75th percentile | 100.690 | 100.690 | 100.690 | 100.690 | 100.690 | 79.405 | 100.690 | - |
| Minimum | -3.670 | -3.670 | -3.670 | 9.775 | -3.670 | 0.490 | -3.670 | - |
| Maximum | 100.690 | 100.690 | 100.690 | 100.690 | 100.690 | 100.690 | 100.690 | - |
| BP     Mean | 66.417 | 82.008 | 59.931 | 77.213 | 63.197 | 56.804 | 83.724 | - |
| Standard deviation | 24.532 | 22.469 | 19.164 | 18.758 | 17.704 | 29.779 | 17.041 | - |
| Median | 56.250 | 95.200 | 56.250 | 95.200 | 56.250 | 43.890 | 95.200 | - |
| 25th percentile | 43.890 | 56.250 | 43.890 | 56.250 | 56.250 | 43.890 | 56.250 | - |
| 75th percentile | 95.200 | 95.200 | 56.250 | 95.200 | 95.200 | 95.200 | 95.200 | - |
| Minimum | 2.280 | 43.890 | 2.280 | 43.890 | 31.500 | 2.280 | 31.500 | - |
| Maximum | 95.200 | 95.200 | 95.200 | 95.200 | 95.200 | 95.200 | 95.200 | - |
| GH    Mean | 66.977 | 78.775 | 78.574 | 77.497 | 68.463 | 52.031 | 65.928 | - |
| Standard deviation | 21.053 | 17.059 | 16.466 | 12.845 | 16.766 | 23.746 | 17.546 | - |
| Median | 61.500 | 83.710 | 83.710 | 83.710 | 83.710 | 61.500 | 61.500 | - |
| 25th percentile | 61.500 | 61.500 | 61.500 | 61.500 | 61.500 | 37.500 | 37.500 | - |
| 75th percentile | 83.710 | 83.710 | 83.710 | 83.710 | 83.710 | 61.500 | 83.710 | - |
| Minimum | 0.580 | 37.500 | 37.500 | 37.500 | 0.580 | 0.580 | 37.500 | - |
| Maximum | 101.840 | 101.840 | 101.840 | 101.840 | 83.710 | 83.710 | 101.840 | - |
| VT     Mean | 52.345 | 72.350 | 54.311 | 51.613 | 58.950 | 40.723 | 46.296 | - |
| Standard deviation | 23.306 | 27.491 | 12.616 | 9.089 | 19.483 | 24.219 | 25.677 | - |
| Median | 45.900 | 79.250 | 54.550 | 54.550 | 54.550 | 31.930 | 45.900 | - |
| 25th percentile | 45.900 | 45.900 | 45.900 | 45.900 | 45.900 | 31.930 | 1.800 | - |
| 75th percentile | 79.250 | 95.340 | 54.550 | 54.550 | 79.250 | 45.900 | 79.250 | - |
| Minimum | 1.800 | 31.930 | 31.930 | 31.930 | 1.800 | 1.800 | 1.800 | - |
| Maximum | 95.340 | 95.340 | 95.340 | 79.250 | 79.250 | 79.250 | 95.340 | - |
| SF      Mean | 73.135 | 63.178 | 69.933 | 86.065 | 68.252 | 67.273 | 84.866 | - |
| Standard deviation | 26.255 | 29.245 | 31.059 | 21.841 | 25.321 | 23.767 | 18.635 | - |
| Median | 66.870 | 66.870 | 99.320 | 99.320 | 66.870 | 66.870 | 99.320 | - |
| 25th percentile | 48.870 | 48.870 | 48.870 | 66.870 | 48.870 | 48.870 | 48.870 | - |
| 75th percentile | 99.320 | 66.870 | 99.320 | 99.320 | 99.320 | 99.320 | 99.320 | - |
| Minimum | 2.780 | 24.910 | 2.780 | 2.780 | 2.780 | 48.870 | 48.870 | - |
| Maximum | 99.320 | 99.320 | 99.320 | 99.320 | 99.320 | 99.320 | 99.320 | - |
| MH   Mean | 73.846 | 73.373 | 70.162 | 71.237 | 74.456 | 69.731 | 82.540 | - |
| Standard deviation | 17.300 | 17.795 | 14.596 | 15.446 | 14.425 | 17.274 | 16.585 | - |
| Median | 74.865 | 83.670 | 74.865 | 69.490 | 74.865 | 74.485 | 92.650 | - |
| 25th percentile | 60.685 | 60.685 | 51.325 | 64.600 | 60.685 | 51.325 | 74.865 | - |
| 75th percentile | 83.845 | 83.670 | 83.670 | 83.845 | 92.650 | 83.845 | 92.650 | - |
| Minimum | 6.005 | 40.305 | 41.440 | 21.060 | 41.440 | 51.325 | 6.005 | - |
| Maximum | 98.595 | 98.595 | 83.845 | 92.650 | 98.595 | 98.595 | 98.595 | - |
| RE     Mean | 78.392 | 82.893 | 79.317 | 89.090 | 72.298 | 55.548 | 98.196 | - |
| Standard deviation | 33.040 | 32.946 | 32.161 | 24.241 | 30.659 | 45.204 | 16.467 | - |
| Median | 104.125 | 104.125 | 104.125 | 104.125 | 81.810 | 54.690 | 104.125 | - |
| 25th percentile | 54.690 | 54.690 | 54.690 | 77.005 | 41.250 | 14.165 | 104.125 | - |
| 75th percentile | 104.125 | 104.125 | 104.125 | 104.125 | 104.125 | 104.125 | 104.125 | - |
| Minimum | -33.415 | -13.835 | -33.415 | -13.835 | -13.835 | -13.835 | 14.165 | - |
| Maximum | 104.125 | 104.125 | 104.125 | 104.125 | 104.125 | 104.125 | 104.125 | - |

BP, bodily pain; GH, general health; MCS, mental component summary; MH, mental health; PCS, physical component summary; PF, physical functioning; RE, role emotional; RP, role physical; VT, vitality; SF, social functioning

**SM3 Table 21**. Canadian norms for VR-12 health utility values, summary component scores, and domain scores, by age group – Nova Scotia, females only.

|  | All participants (n=123) | 18 to 29 (n=16) | 30 to 39 (n=20) | 40 to 49 (n=23) | 50 to 59 (n=26) | 60 to 69 (n=22) | 70 to 79 (n=14) | 80 and over (n=2) |
| --- | --- | --- | --- | --- | --- | --- | --- | --- |
| *VR-12 health utility values* |  |  |  |  |  |  |  |  |
| Mean | 0.688 | 0.522 | 0.766 | 0.530 | 0.756 | 0.727 | 0.613 | - |
| Standard deviation | 0.213 | 0.184 | 0.109 | 0.236 | 0.175 | 0.247 | 0.230 | - |
| Median | 0.769 | 0.644 | 0.769 | 0.696 | 0.791 | 0.818 | 0.717 | - |
| 25th percentile | 0.644 | 0.365 | 0.706 | 0.322 | 0.779 | 0.668 | 0.644 | - |
| 75th percentile | 0.852 | 0.696 | 0.869 | 0.818 | 0.852 | 0.860 | 0.777 | - |
| Minimum | -0.590 | 0.127 | 0.350 | -0.590 | -0.093 | -0.177 | -0.080 | - |
| Maximum | 1.000 | 0.799 | 1.000 | 0.919 | 0.913 | 1.000 | 0.913 | - |
| *VR-12 summary component score* |  |  |  |  |  |  |  |  |
| PCS   Mean | 48.666 | 48.676 | 52.928 | 48.593 | 48.707 | 50.598 | 38.002 | - |
| Standard deviation | 10.172 | 9.458 | 6.887 | 8.291 | 10.777 | 12.031 | 11.353 | - |
| Median | 52.367 | 51.360 | 54.704 | 52.367 | 52.773 | 56.707 | 41.358 | - |
| 25th percentile | 41.756 | 36.160 | 51.101 | 44.145 | 41.756 | 49.665 | 33.788 | - |
| 75th percentile | 57.908 | 58.931 | 60.590 | 56.759 | 57.908 | 58.371 | 42.886 | - |
| Minimum | 7.468 | 30.784 | 19.643 | 17.412 | 16.738 | 18.292 | 7.468 | - |
| Maximum | 64.464 | 64.464 | 62.387 | 60.576 | 59.253 | 59.022 | 58.016 | - |
| MCS  Mean | 50.915 | 36.222 | 50.352 | 42.174 | 56.878 | 51.975 | 54.922 | - |
| Standard deviation | 9.757 | 7.110 | 5.626 | 8.419 | 9.383 | 9.477 | 5.169 | - |
| Median | 53.343 | 33.929 | 49.616 | 45.955 | 56.980 | 53.003 | 57.213 | - |
| 25th percentile | 44.809 | 32.238 | 46.115 | 36.180 | 53.878 | 46.751 | 54.828 | - |
| 75th percentile | 57.213 | 41.483 | 54.576 | 51.931 | 67.355 | 57.219 | 57.530 | - |
| Minimum | 18.705 | 19.036 | 30.794 | 18.705 | 33.743 | 20.102 | 36.156 | - |
| Maximum | 68.956 | 60.204 | 62.711 | 59.074 | 67.355 | 65.767 | 68.956 | - |
| *VR-12 domain scores* |  |  |  |  |  |  |  |  |
| PF     Mean | 67.379 | 74.106 | 83.602 | 64.390 | 58.814 | 75.747 | 52.847 | - |
| Standard deviation | 28.348 | 14.977 | 15.913 | 21.652 | 42.438 | 30.035 | 22.042 | - |
| Median | 80.375 | 71.400 | 93.485 | 80.375 | 80.375 | 93.485 | 58.290 | - |
| 25th percentile | 58.290 | 58.290 | 80.375 | 29.460 | 7.765 | 71.400 | 29.460 | - |
| 75th percentile | 93.485 | 93.485 | 93.485 | 93.485 | 93.485 | 93.485 | 58.290 | - |
| Minimum | 7.765 | 29.460 | 7.765 | 7.765 | 7.765 | 7.765 | 7.765 | - |
| Maximum | 93.485 | 93.485 | 93.485 | 93.485 | 93.485 | 93.485 | 93.485 | - |
| RP     Mean | 77.990 | 55.806 | 88.295 | 74.178 | 86.663 | 78.257 | 61.295 | - |
| Standard deviation | 31.767 | 34.621 | 17.371 | 26.894 | 34.263 | 39.589 | 28.492 | - |
| Median | 100.690 | 79.405 | 100.690 | 100.690 | 100.690 | 100.690 | 79.405 | - |
| 25th percentile | 79.405 | 9.775 | 79.405 | 54.725 | 100.690 | 79.405 | 44.460 | - |
| 75th percentile | 100.690 | 100.690 | 100.690 | 100.690 | 100.690 | 100.690 | 79.405 | - |
| Minimum | -3.670 | 0.490 | 0.490 | -3.670 | -3.670 | -3.670 | -3.670 | - |
| Maximum | 100.690 | 100.690 | 100.690 | 100.690 | 100.690 | 100.690 | 100.690 | - |
| BP     Mean | 74.079 | 59.287 | 78.270 | 63.095 | 83.104 | 79.233 | 51.264 | - |
| Standard deviation | 23.609 | 20.274 | 15.777 | 21.289 | 23.317 | 26.850 | 21.886 | - |
| Median | 95.200 | 56.250 | 95.200 | 56.250 | 95.200 | 95.200 | 56.250 | - |
| 25th percentile | 56.250 | 43.890 | 56.250 | 56.250 | 56.250 | 56.250 | 43.890 | - |
| 75th percentile | 95.200 | 95.200 | 95.200 | 95.200 | 95.200 | 95.200 | 56.250 | - |
| Minimum | 2.280 | 31.500 | 31.500 | 2.280 | 2.280 | 2.280 | 2.280 | - |
| Maximum | 95.200 | 95.200 | 95.200 | 95.200 | 95.200 | 95.200 | 95.200 | - |
| GH    Mean | 70.410 | 54.265 | 72.894 | 69.548 | 77.269 | 71.563 | 57.216 | - |
| Standard deviation | 19.202 | 21.554 | 11.703 | 11.955 | 14.626 | 26.351 | 22.230 | - |
| Median | 83.710 | 61.500 | 83.710 | 83.710 | 83.710 | 83.710 | 61.500 | - |
| 25th percentile | 61.500 | 61.500 | 61.500 | 61.500 | 83.710 | 61.500 | 61.500 | - |
| 75th percentile | 83.710 | 61.500 | 83.710 | 83.710 | 83.710 | 83.710 | 61.500 | - |
| Minimum | 0.580 | 0.580 | 0.580 | 37.500 | 0.580 | 0.580 | 0.580 | - |
| Maximum | 101.840 | 83.710 | 83.710 | 83.710 | 83.710 | 101.840 | 101.840 | - |
| VT     Mean | 55.827 | 44.105 | 55.640 | 41.677 | 66.895 | 57.631 | 45.239 | - |
| Standard deviation | 20.774 | 12.673 | 18.788 | 15.631 | 23.084 | 23.319 | 19.589 | - |
| Median | 54.550 | 45.900 | 54.550 | 45.900 | 79.250 | 54.550 | 45.900 | - |
| 25th percentile | 45.900 | 31.930 | 31.930 | 31.930 | 54.550 | 45.900 | 31.930 | - |
| 75th percentile | 79.250 | 45.900 | 79.250 | 45.900 | 79.250 | 79.250 | 54.550 | - |
| Minimum | 1.800 | 31.930 | 1.800 | 1.800 | 1.800 | 1.800 | 1.800 | - |
| Maximum | 95.340 | 79.250 | 95.340 | 79.250 | 79.250 | 95.340 | 79.250 | - |
| SF      Mean | 78.000 | 48.954 | 77.856 | 58.776 | 87.591 | 83.353 | 79.866 | - |
| Standard deviation | 25.717 | 24.724 | 17.243 | 25.542 | 25.282 | 23.440 | 22.345 | - |
| Median | 99.320 | 48.870 | 66.870 | 66.870 | 99.320 | 99.320 | 99.320 | - |
| 25th percentile | 66.870 | 24.910 | 66.870 | 24.910 | 99.320 | 66.870 | 66.870 | - |
| 75th percentile | 99.320 | 66.870 | 99.320 | 99.320 | 99.320 | 99.320 | 99.320 | - |
| Minimum | 2.780 | 2.780 | 24.910 | 2.780 | 24.910 | 24.910 | 24.910 | - |
| Maximum | 99.320 | 99.320 | 99.320 | 99.320 | 99.320 | 99.320 | 99.320 | - |
| MH   Mean | 71.150 | 44.716 | 70.507 | 55.146 | 82.036 | 74.178 | 75.481 | - |
| Standard deviation | 20.669 | 13.873 | 14.976 | 23.110 | 19.015 | 20.799 | 10.176 | - |
| Median | 74.865 | 51.325 | 74.865 | 65.505 | 83.670 | 74.865 | 74.865 | - |
| 25th percentile | 60.685 | 21.060 | 51.325 | 41.440 | 83.670 | 65.505 | 74.865 | - |
| 75th percentile | 83.845 | 60.685 | 89.615 | 74.865 | 98.595 | 92.650 | 83.670 | - |
| Minimum | -1.030 | 21.060 | 23.910 | -1.030 | 16.745 | 14.025 | 41.685 | - |
| Maximum | 98.595 | 83.670 | 98.595 | 98.595 | 98.595 | 98.595 | 98.595 | - |
| RE     Mean | 74.916 | 28.542 | 79.959 | 50.451 | 90.994 | 79.162 | 90.448 | - |
| Standard deviation | 33.263 | 29.538 | 18.835 | 27.308 | 27.876 | 40.077 | 22.459 | - |
| Median | 104.125 | 29.335 | 81.810 | 41.250 | 104.125 | 104.125 | 104.125 | - |
| 25th percentile | 54.690 | 2.250 | 54.690 | 29.335 | 81.810 | 77.005 | 104.125 | - |
| 75th percentile | 104.125 | 41.285 | 104.125 | 104.125 | 104.125 | 104.125 | 104.125 | - |
| Minimum | -33.415 | -13.835 | 14.165 | -33.415 | 2.250 | -20.070 | 2.250 | - |
| Maximum | 104.125 | 104.125 | 104.125 | 104.125 | 104.125 | 104.125 | 104.125 | - |

BP, bodily pain; GH, general health; MCS, mental component summary; MH, mental health; PCS, physical component summary; PF, physical functioning; RE, role emotional; RP, role physical; VT, vitality; SF, social functioning

**SM3 Table 22**. Canadian norms for VR-12 health utility values, summary component scores, and domain scores, by age group – New Brunswick.

|  | All participants (n=153) | 18 to 29 (n=18) | 30 to 39 (n=19) | 40 to 49 (n=25) | 50 to 59 (n=47) | 60 to 69 (n=23) | 70 to 79 (n=18) | 80 and over (n=3) |
| --- | --- | --- | --- | --- | --- | --- | --- | --- |
| *VR-12 health utility values* |  |  |  |  |  |  |  |  |
| Mean | 0.683 | 0.601 | 0.690 | 0.725 | 0.720 | 0.648 | 0.797 | - |
| Standard deviation | 0.178 | 0.226 | 0.123 | 0.209 | 0.109 | 0.256 | 0.094 | - |
| Median | 0.746 | 0.696 | 0.736 | 0.839 | 0.747 | 0.769 | 0.822 | - |
| 25th percentile | 0.606 | 0.301 | 0.644 | 0.696 | 0.664 | 0.519 | 0.750 | - |
| 75th percentile | 0.812 | 0.778 | 0.799 | 0.879 | 0.821 | 0.799 | 0.913 | - |
| Minimum | -0.334 | 0.213 | 0.287 | -0.334 | -0.024 | -0.186 | 0.358 | - |
| Maximum | 0.952 | 0.913 | 0.861 | 0.952 | 0.913 | 0.913 | 0.952 | - |
| *VR-12 summary component score* |  |  |  |  |  |  |  |  |
| PCS   Mean | 48.747 | 54.140 | 52.893 | 51.364 | 47.843 | 46.385 | 46.830 | - |
| Standard deviation | 8.345 | 4.536 | 5.691 | 6.763 | 5.896 | 12.542 | 5.366 | - |
| Median | 52.612 | 54.370 | 55.090 | 53.075 | 49.775 | 46.118 | 49.009 | - |
| 25th percentile | 42.513 | 53.634 | 49.721 | 51.362 | 43.996 | 38.999 | 41.147 | - |
| 75th percentile | 56.229 | 56.247 | 58.342 | 56.824 | 53.067 | 57.299 | 51.990 | - |
| Minimum | 14.977 | 43.180 | 14.977 | 20.710 | 26.211 | 19.202 | 21.529 | - |
| Maximum | 63.474 | 63.474 | 59.336 | 61.137 | 59.471 | 59.623 | 57.509 | - |
| MCS  Mean | 49.857 | 41.827 | 47.443 | 51.438 | 52.250 | 50.520 | 57.637 | - |
| Standard deviation | 7.699 | 9.569 | 6.417 | 7.649 | 4.692 | 8.761 | 3.498 | - |
| Median | 51.505 | 40.479 | 47.780 | 56.980 | 53.503 | 51.505 | 58.443 | - |
| 25th percentile | 44.576 | 30.812 | 42.804 | 46.682 | 47.633 | 46.548 | 54.250 | - |
| 75th percentile | 56.358 | 50.412 | 54.611 | 59.588 | 56.201 | 55.768 | 60.987 | - |
| Minimum | 21.948 | 25.610 | 30.499 | 29.736 | 23.905 | 21.948 | 43.967 | - |
| Maximum | 64.834 | 59.588 | 62.516 | 61.487 | 62.848 | 62.458 | 64.834 | - |
| *VR-12 domain scores* |  |  |  |  |  |  |  |  |
| PF     Mean | 76.616 | 88.755 | 82.074 | 83.444 | 72.779 | 73.852 | 72.905 | - |
| Standard deviation | 20.017 | 9.826 | 14.902 | 16.279 | 16.174 | 28.760 | 15.340 | - |
| Median | 93.485 | 93.485 | 93.485 | 93.485 | 80.375 | 80.375 | 80.375 | - |
| 25th percentile | 58.290 | 93.485 | 71.400 | 80.375 | 58.290 | 58.290 | 58.290 | - |
| 75th percentile | 93.485 | 93.485 | 93.485 | 93.485 | 93.485 | 93.485 | 93.485 | - |
| Minimum | 7.765 | 58.290 | 7.765 | 7.765 | 7.765 | 7.765 | 7.765 | - |
| Maximum | 93.485 | 93.485 | 93.485 | 93.485 | 93.485 | 93.485 | 93.485 | - |
| RP     Mean | 72.951 | 69.702 | 78.303 | 83.788 | 77.303 | 68.902 | 82.257 | - |
| Standard deviation | 28.983 | 28.472 | 23.297 | 24.752 | 22.234 | 39.575 | 18.783 | - |
| Median | 89.670 | 79.405 | 89.670 | 100.690 | 89.670 | 79.405 | 100.690 | - |
| 25th percentile | 49.970 | 54.725 | 54.725 | 79.405 | 79.405 | 44.460 | 79.405 | - |
| 75th percentile | 100.690 | 89.670 | 100.690 | 100.690 | 100.690 | 100.690 | 100.690 | - |
| Minimum | -3.670 | 9.775 | -3.670 | -3.670 | -3.670 | 0.490 | 0.490 | - |
| Maximum | 100.690 | 100.690 | 100.690 | 100.690 | 100.690 | 100.690 | 100.690 | - |
| BP     Mean | 66.831 | 65.863 | 77.482 | 70.920 | 66.259 | 63.149 | 69.793 | - |
| Standard deviation | 20.503 | 17.614 | 17.298 | 19.455 | 15.980 | 29.787 | 14.023 | - |
| Median | 56.250 | 56.250 | 95.200 | 56.250 | 56.250 | 56.250 | 56.250 | - |
| 25th percentile | 56.250 | 56.250 | 56.250 | 56.250 | 56.250 | 31.500 | 56.250 | - |
| 75th percentile | 95.200 | 95.200 | 95.200 | 95.200 | 95.200 | 95.200 | 95.200 | - |
| Minimum | 2.280 | 43.890 | 31.500 | 2.280 | 31.500 | 31.500 | 31.500 | - |
| Maximum | 95.200 | 95.200 | 95.200 | 95.200 | 95.200 | 95.200 | 95.200 | - |
| GH    Mean | 68.194 | 74.580 | 70.739 | 72.640 | 67.289 | 66.937 | 60.900 | - |
| Standard deviation | 16.683 | 14.784 | 11.900 | 14.754 | 12.105 | 24.188 | 17.221 | - |
| Median | 61.500 | 83.710 | 83.710 | 83.710 | 61.500 | 61.500 | 61.500 | - |
| 25th percentile | 61.500 | 61.500 | 61.500 | 61.500 | 61.500 | 61.500 | 61.500 | - |
| 75th percentile | 83.710 | 83.710 | 83.710 | 83.710 | 83.710 | 83.710 | 83.710 | - |
| Minimum | 0.580 | 37.500 | 37.500 | 0.580 | 0.580 | 0.580 | 0.580 | - |
| Maximum | 101.840 | 101.840 | 83.710 | 101.840 | 101.840 | 101.840 | 83.710 | - |
| VT     Mean | 52.895 | 41.481 | 60.327 | 55.656 | 52.356 | 54.268 | 63.332 | - |
| Standard deviation | 19.132 | 27.801 | 17.918 | 17.662 | 13.778 | 22.408 | 12.895 | - |
| Median | 54.550 | 45.900 | 79.250 | 54.550 | 45.900 | 54.550 | 79.250 | - |
| 25th percentile | 45.900 | 1.800 | 45.900 | 45.900 | 45.900 | 45.900 | 54.550 | - |
| 75th percentile | 79.250 | 54.550 | 79.250 | 79.250 | 79.250 | 79.250 | 79.250 | - |
| Minimum | 1.800 | 1.800 | 1.800 | 1.800 | 1.800 | 1.800 | 31.930 | - |
| Maximum | 79.250 | 79.250 | 79.250 | 79.250 | 79.250 | 79.250 | 79.250 | - |
| SF      Mean | 74.082 | 62.517 | 67.063 | 78.536 | 85.068 | 66.183 | 91.040 | - |
| Standard deviation | 22.995 | 21.218 | 16.922 | 19.584 | 14.695 | 37.367 | 11.855 | - |
| Median | 66.870 | 48.870 | 66.870 | 99.320 | 99.320 | 66.870 | 99.320 | - |
| 25th percentile | 48.870 | 48.870 | 48.870 | 66.870 | 66.870 | 24.910 | 99.320 | - |
| 75th percentile | 99.320 | 66.870 | 66.870 | 99.320 | 99.320 | 99.320 | 99.320 | - |
| Minimum | 2.780 | 24.910 | 24.910 | 2.780 | 2.780 | 2.780 | 24.910 | - |
| Maximum | 99.320 | 99.320 | 99.320 | 99.320 | 99.320 | 99.320 | 99.320 | - |
| MH   Mean | 70.582 | 54.266 | 65.272 | 73.157 | 72.848 | 73.157 | 83.775 | - |
| Standard deviation | 18.661 | 30.626 | 14.827 | 18.323 | 13.749 | 18.007 | 9.358 | - |
| Median | 74.865 | 65.505 | 69.490 | 83.670 | 83.670 | 74.865 | 92.650 | - |
| 25th percentile | 65.505 | 16.745 | 51.045 | 64.600 | 60.685 | 69.490 | 74.485 | - |
| 75th percentile | 83.670 | 74.865 | 74.865 | 92.650 | 83.845 | 83.670 | 92.650 | - |
| Minimum | 6.005 | 6.005 | 21.060 | 6.005 | 23.910 | 6.005 | 55.620 | - |
| Maximum | 98.595 | 98.595 | 92.650 | 98.595 | 98.595 | 92.650 | 98.595 | - |
| RE     Mean | 73.109 | 57.230 | 69.361 | 83.580 | 78.673 | 77.233 | 96.186 | - |
| Standard deviation | 29.215 | 29.691 | 22.068 | 25.079 | 20.111 | 33.592 | 13.577 | - |
| Median | 81.810 | 54.690 | 54.690 | 104.125 | 81.810 | 81.810 | 104.125 | - |
| 25th percentile | 54.690 | 29.335 | 54.690 | 54.690 | 54.690 | 68.370 | 104.125 | - |
| 75th percentile | 104.125 | 81.810 | 104.125 | 104.125 | 104.125 | 104.125 | 104.125 | - |
| Minimum | -33.415 | -13.835 | 2.250 | -20.070 | -33.415 | -13.835 | 29.335 | - |
| Maximum | 104.125 | 104.125 | 104.125 | 104.125 | 104.125 | 104.125 | 104.125 | - |

BP, bodily pain; GH, general health; MCS, mental component summary; MH, mental health; PCS, physical component summary; PF, physical functioning; RE, role emotional; RP, role physical; VT, vitality; SF, social functioning

**SM3 Table 23**. Canadian norms for VR-12 health utility values, summary component scores, and domain scores, by age group – New Brunswick, males only.

|  | All participants (n=70) | 18 to 29 (n=7) | 30 to 39 (n=7) | 40 to 49 (n=18) | 50 to 59 (n=18) | 60 to 69 (n=7) | 70 to 79 (n=11) | 80 and over (n=2) |
| --- | --- | --- | --- | --- | --- | --- | --- | --- |
| *VR-12 health utility values* |  |  |  |  |  |  |  |  |
| Mean | 0.752 | 0.726 | 0.738 | 0.808 | 0.736 | 0.777 | 0.822 | - |
| Standard deviation | 0.113 | 0.090 | 0.117 | 0.094 | 0.073 | 0.139 | 0.055 | - |
| Median | 0.768 | 0.696 | 0.736 | 0.839 | 0.749 | 0.799 | 0.822 | - |
| 25th percentile | 0.696 | 0.696 | 0.706 | 0.716 | 0.678 | 0.696 | 0.750 | - |
| 75th percentile | 0.852 | 0.778 | 0.812 | 0.903 | 0.821 | 0.913 | 0.879 | - |
| Minimum | 0.331 | 0.606 | 0.331 | 0.486 | 0.553 | 0.459 | 0.707 | - |
| Maximum | 0.952 | 0.913 | 0.861 | 0.952 | 0.872 | 0.913 | 0.913 | - |
| *VR-12 summary component score* |  |  |  |  |  |  |  |  |
| PCS   Mean | 50.484 | 53.545 | 53.048 | 53.740 | 49.960 | 53.179 | 48.929 | - |
| Standard deviation | 7.320 | 4.243 | 5.124 | 4.100 | 2.630 | 7.441 | 4.023 | - |
| Median | 53.075 | 55.521 | 55.090 | 53.879 | 49.775 | 56.824 | 49.009 | - |
| 25th percentile | 48.260 | 53.634 | 49.721 | 52.814 | 48.260 | 47.169 | 45.270 | - |
| 75th percentile | 56.198 | 56.247 | 56.198 | 56.824 | 51.659 | 58.875 | 52.748 | - |
| Minimum | 27.588 | 45.479 | 32.833 | 35.078 | 40.824 | 35.844 | 40.644 | - |
| Maximum | 61.137 | 56.824 | 57.791 | 61.137 | 57.445 | 58.875 | 57.509 | - |
| MCS  Mean | 52.321 | 47.215 | 51.507 | 54.221 | 52.770 | 53.933 | 58.224 | - |
| Standard deviation | 6.335 | 8.738 | 6.649 | 6.254 | 3.574 | 4.600 | 3.292 | - |
| Median | 53.849 | 48.737 | 48.080 | 58.248 | 53.503 | 52.002 | 58.443 | - |
| 25th percentile | 47.365 | 39.767 | 43.908 | 48.935 | 51.508 | 51.505 | 54.250 | - |
| 75th percentile | 58.248 | 56.090 | 57.512 | 59.588 | 55.160 | 59.588 | 60.987 | - |
| Minimum | 31.268 | 36.186 | 31.268 | 38.482 | 44.320 | 48.103 | 50.266 | - |
| Maximum | 64.834 | 59.588 | 62.516 | 61.487 | 62.618 | 59.608 | 64.834 | - |
| *VR-12 domain scores* |  |  |  |  |  |  |  |  |
| PF     Mean | 80.344 | 89.293 | 81.671 | 90.612 | 76.151 | 83.879 | 77.810 | - |
| Standard deviation | 18.133 | 9.081 | 15.860 | 7.080 | 11.822 | 21.451 | 11.073 | - |
| Median | 93.485 | 93.485 | 93.485 | 93.485 | 80.375 | 93.485 | 80.375 | - |
| 25th percentile | 71.400 | 93.485 | 58.290 | 93.485 | 58.290 | 93.485 | 58.290 | - |
| 75th percentile | 93.485 | 93.485 | 93.485 | 93.485 | 93.485 | 93.485 | 93.485 | - |
| Minimum | 29.460 | 71.400 | 36.595 | 58.290 | 58.290 | 29.460 | 58.290 | - |
| Maximum | 93.485 | 93.485 | 93.485 | 93.485 | 93.485 | 93.485 | 93.485 | - |
| RP     Mean | 80.647 | 72.335 | 91.793 | 92.665 | 84.472 | 93.757 | 87.195 | - |
| Standard deviation | 26.137 | 32.819 | 17.667 | 11.482 | 13.171 | 11.391 | 12.419 | - |
| Median | 89.670 | 79.405 | 100.690 | 100.690 | 89.670 | 100.690 | 89.670 | - |
| 25th percentile | 79.405 | 79.405 | 89.670 | 89.670 | 79.405 | 79.405 | 79.405 | - |
| 75th percentile | 100.690 | 89.670 | 100.690 | 100.690 | 100.690 | 100.690 | 100.690 | - |
| Minimum | 0.490 | 9.775 | 9.775 | 44.460 | 44.720 | 79.405 | 44.460 | - |
| Maximum | 100.690 | 100.690 | 100.690 | 100.690 | 100.690 | 100.690 | 100.690 | - |
| BP     Mean | 70.244 | 61.787 | 78.468 | 73.288 | 68.899 | 81.078 | 70.380 | - |
| Standard deviation | 20.123 | 14.263 | 18.298 | 18.446 | 14.830 | 24.010 | 14.152 | - |
| Median | 56.250 | 56.250 | 95.200 | 56.250 | 56.250 | 95.200 | 56.250 | - |
| 25th percentile | 56.250 | 56.250 | 56.250 | 56.250 | 56.250 | 56.250 | 56.250 | - |
| 75th percentile | 95.200 | 56.250 | 95.200 | 95.200 | 95.200 | 95.200 | 95.200 | - |
| Minimum | 31.500 | 56.250 | 31.500 | 31.500 | 31.500 | 31.500 | 56.250 | - |
| Maximum | 95.200 | 95.200 | 95.200 | 95.200 | 95.200 | 95.200 | 95.200 | - |
| GH    Mean | 73.056 | 83.485 | 74.867 | 78.055 | 70.397 | 74.741 | 70.872 | - |
| Standard deviation | 14.970 | 11.740 | 12.598 | 10.685 | 10.453 | 17.318 | 11.629 | - |
| Median | 83.710 | 83.710 | 83.710 | 83.710 | 61.500 | 83.710 | 83.710 | - |
| 25th percentile | 61.500 | 83.710 | 61.500 | 61.500 | 61.500 | 61.500 | 61.500 | - |
| 75th percentile | 83.710 | 83.710 | 83.710 | 83.710 | 83.710 | 83.710 | 83.710 | - |
| Minimum | 37.500 | 37.500 | 37.500 | 61.500 | 37.500 | 37.500 | 37.500 | - |
| Maximum | 101.840 | 101.840 | 83.710 | 101.840 | 83.710 | 83.710 | 83.710 | - |
| VT     Mean | 59.581 | 60.449 | 64.184 | 61.937 | 58.359 | 65.054 | 66.617 | - |
| Standard deviation | 15.964 | 16.005 | 14.550 | 14.334 | 14.748 | 18.875 | 12.299 | - |
| Median | 54.550 | 54.550 | 79.250 | 54.550 | 54.550 | 79.250 | 79.250 | - |
| 25th percentile | 45.900 | 45.900 | 45.900 | 45.900 | 45.900 | 54.550 | 54.550 | - |
| 75th percentile | 79.250 | 79.250 | 79.250 | 79.250 | 79.250 | 79.250 | 79.250 | - |
| Minimum | 1.800 | 45.900 | 31.930 | 31.930 | 1.800 | 31.930 | 31.930 | - |
| Maximum | 79.250 | 79.250 | 79.250 | 79.250 | 79.250 | 79.250 | 79.250 | - |
| SF      Mean | 79.696 | 57.905 | 70.225 | 85.382 | 87.759 | 82.085 | 93.851 | - |
| Standard deviation | 19.205 | 21.826 | 18.421 | 15.754 | 12.509 | 18.493 | 9.180 | - |
| Median | 99.320 | 66.870 | 66.870 | 99.320 | 99.320 | 66.870 | 99.320 | - |
| 25th percentile | 66.870 | 48.870 | 48.870 | 66.870 | 66.870 | 66.870 | 99.320 | - |
| 75th percentile | 99.320 | 66.870 | 99.320 | 99.320 | 99.320 | 99.320 | 99.320 | - |
| Minimum | 24.910 | 24.910 | 24.910 | 48.870 | 48.870 | 66.870 | 66.870 | - |
| Maximum | 99.320 | 99.320 | 99.320 | 99.320 | 99.320 | 99.320 | 99.320 | - |
| MH   Mean | 78.196 | 79.632 | 78.488 | 80.569 | 73.150 | 78.472 | 82.970 | - |
| Standard deviation | 12.638 | 13.544 | 11.323 | 13.137 | 13.420 | 14.645 | 9.967 | - |
| Median | 83.670 | 83.670 | 74.865 | 83.845 | 83.670 | 83.670 | 92.650 | - |
| 25th percentile | 69.490 | 65.505 | 74.865 | 65.505 | 60.685 | 69.490 | 74.485 | - |
| 75th percentile | 92.650 | 92.650 | 83.670 | 92.650 | 83.670 | 92.650 | 92.650 | - |
| Minimum | 21.060 | 65.505 | 21.060 | 41.685 | 41.440 | 41.440 | 60.685 | - |
| Maximum | 98.595 | 98.595 | 92.650 | 98.595 | 98.595 | 92.650 | 98.595 | - |
| RE     Mean | 75.084 | 50.459 | 80.646 | 92.132 | 75.507 | 88.023 | 101.531 | - |
| Standard deviation | 32.075 | 29.975 | 25.219 | 19.344 | 16.598 | 26.456 | 5.405 | - |
| Median | 81.810 | 41.250 | 81.810 | 104.125 | 77.005 | 104.125 | 104.125 | - |
| 25th percentile | 54.690 | 29.335 | 54.690 | 81.810 | 54.690 | 54.690 | 104.125 | - |
| 75th percentile | 104.125 | 54.690 | 104.125 | 104.125 | 104.125 | 104.125 | 104.125 | - |
| Minimum | -13.835 | 13.250 | 2.250 | 41.250 | 54.690 | 54.690 | 81.810 | - |
| Maximum | 104.125 | 104.125 | 104.125 | 104.125 | 104.125 | 104.125 | 104.125 | - |

BP, bodily pain; GH, general health; MCS, mental component summary; MH, mental health; PCS, physical component summary; PF, physical functioning; RE, role emotional; RP, role physical; VT, vitality; SF, social functioning

**SM3 Table 24**. Canadian norms for VR-12 health utility values, summary component scores, and domain scores, by age group – New Brunswick, females only.

|  | All participants (n=83) | 18 to 29 (n=11) | 30 to 39 (n=12) | 40 to 49 (n=7) | 50 to 59 (n=29) | 60 to 69 (n=16) | 70 to 79 (n=7) | 80 and over (n=1) |
| --- | --- | --- | --- | --- | --- | --- | --- | --- |
| *VR-12 health utility values* |  |  |  |  |  |  |  |  |
| Mean | 0.616 | 0.518 | 0.661 | 0.290 | 0.709 | 0.594 | 0.727 | - |
| Standard deviation | 0.205 | 0.255 | 0.127 | 0.227 | 0.127 | 0.276 | 0.134 | - |
| Median | 0.707 | 0.644 | 0.669 | 0.268 | 0.746 | 0.608 | 0.787 | - |
| 25th percentile | 0.519 | 0.213 | 0.508 | 0.106 | 0.644 | 0.519 | 0.444 | - |
| 75th percentile | 0.799 | 0.779 | 0.779 | 0.623 | 0.818 | 0.799 | 0.913 | - |
| Minimum | -0.334 | 0.213 | 0.287 | -0.334 | -0.024 | -0.186 | 0.358 | - |
| Maximum | 0.952 | 0.810 | 0.852 | 0.684 | 0.913 | 0.818 | 0.952 | - |
| *VR-12 summary component score* |  |  |  |  |  |  |  |  |
| PCS   Mean | 47.080 | 54.533 | 52.803 | 38.871 | 46.438 | 43.543 | 40.949 | - |
| Standard deviation | 8.946 | 4.877 | 6.219 | 7.433 | 7.086 | 13.206 | 6.218 | - |
| Median | 51.128 | 54.370 | 55.127 | 40.936 | 49.882 | 38.999 | 43.800 | - |
| 25th percentile | 38.999 | 54.346 | 51.128 | 29.906 | 41.959 | 36.873 | 31.743 | - |
| 75th percentile | 56.229 | 56.337 | 59.170 | 50.858 | 53.297 | 57.299 | 51.038 | - |
| Minimum | 14.977 | 43.180 | 14.977 | 20.710 | 26.211 | 19.202 | 21.529 | - |
| Maximum | 63.474 | 63.474 | 59.336 | 51.362 | 59.471 | 59.623 | 51.990 | - |
| MCS  Mean | 47.491 | 38.263 | 45.062 | 36.804 | 51.905 | 49.093 | 55.992 | - |
| Standard deviation | 8.254 | 8.832 | 5.752 | 3.651 | 5.316 | 9.772 | 3.921 | - |
| Median | 48.022 | 35.517 | 47.492 | 32.798 | 54.413 | 51.093 | 59.656 | - |
| 25th percentile | 41.029 | 30.812 | 40.314 | 31.977 | 47.633 | 46.548 | 51.282 | - |
| 75th percentile | 55.768 | 47.490 | 48.022 | 42.331 | 56.358 | 53.692 | 60.987 | - |
| Minimum | 21.948 | 25.610 | 30.499 | 29.736 | 23.905 | 21.948 | 43.967 | - |
| Maximum | 63.618 | 52.360 | 55.770 | 43.818 | 62.848 | 62.458 | 61.539 | - |
| *VR-12 domain scores* |  |  |  |  |  |  |  |  |
| PF     Mean | 73.038 | 88.399 | 82.310 | 45.743 | 70.542 | 69.659 | 59.165 | - |
| Standard deviation | 21.160 | 10.692 | 15.036 | 13.737 | 18.387 | 30.898 | 19.375 | - |
| Median | 80.375 | 93.485 | 93.485 | 58.290 | 80.375 | 80.375 | 80.375 | - |
| 25th percentile | 58.290 | 93.485 | 80.375 | 29.460 | 58.290 | 58.290 | 29.460 | - |
| 75th percentile | 93.485 | 93.485 | 93.485 | 58.290 | 93.485 | 93.485 | 93.485 | - |
| Minimum | 7.765 | 58.290 | 7.765 | 7.765 | 7.765 | 7.765 | 7.765 | - |
| Maximum | 93.485 | 93.485 | 93.485 | 71.400 | 93.485 | 93.485 | 93.485 | - |
| RP     Mean | 65.564 | 67.960 | 70.396 | 37.104 | 72.545 | 58.508 | 68.421 | - |
| Standard deviation | 30.073 | 26.912 | 24.608 | 30.964 | 25.997 | 42.287 | 25.580 | - |
| Median | 79.405 | 54.725 | 79.405 | 9.775 | 90.425 | 44.460 | 100.690 | - |
| 25th percentile | 44.460 | 54.725 | 45.835 | -3.670 | 44.460 | 44.460 | 9.775 | - |
| 75th percentile | 100.690 | 89.670 | 100.690 | 89.670 | 100.690 | 100.690 | 100.690 | - |
| Minimum | -3.670 | 9.775 | -3.670 | -3.670 | -3.670 | 0.490 | 0.490 | - |
| Maximum | 100.690 | 100.690 | 100.690 | 100.690 | 100.690 | 100.690 | 100.690 | - |
| BP     Mean | 63.556 | 68.559 | 76.904 | 58.468 | 64.507 | 55.652 | 68.151 | - |
| Standard deviation | 20.571 | 19.677 | 17.504 | 21.688 | 16.787 | 28.860 | 14.903 | - |
| Median | 56.250 | 56.250 | 95.200 | 56.250 | 56.250 | 43.890 | 56.250 | - |
| 25th percentile | 43.890 | 56.250 | 56.250 | 31.500 | 56.250 | 31.500 | 43.890 | - |
| 75th percentile | 95.200 | 95.200 | 95.200 | 95.200 | 95.200 | 95.200 | 95.200 | - |
| Minimum | 2.280 | 43.890 | 31.500 | 2.280 | 31.500 | 31.500 | 31.500 | - |
| Maximum | 95.200 | 95.200 | 95.200 | 95.200 | 95.200 | 95.200 | 95.200 | - |
| GH    Mean | 63.527 | 68.690 | 68.319 | 44.160 | 65.227 | 63.673 | 32.963 | - |
| Standard deviation | 17.212 | 14.211 | 11.603 | 11.449 | 12.983 | 26.327 | 15.970 | - |
| Median | 61.500 | 61.500 | 61.500 | 37.500 | 61.500 | 61.500 | 37.500 | - |
| 25th percentile | 61.500 | 61.500 | 61.500 | 37.500 | 61.500 | 61.500 | 0.580 | - |
| 75th percentile | 83.710 | 83.710 | 83.710 | 61.500 | 83.710 | 83.710 | 61.500 | - |
| Minimum | 0.580 | 37.500 | 37.500 | 0.580 | 0.580 | 0.580 | 0.580 | - |
| Maximum | 101.840 | 101.840 | 83.710 | 61.500 | 101.840 | 101.840 | 61.500 | - |
| VT     Mean | 46.476 | 28.936 | 58.067 | 22.627 | 48.374 | 49.758 | 54.127 | - |
| Standard deviation | 20.131 | 27.674 | 20.017 | 10.286 | 12.574 | 22.536 | 13.414 | - |
| Median | 45.900 | 45.900 | 54.550 | 31.930 | 45.900 | 54.550 | 45.900 | - |
| 25th percentile | 31.930 | 1.800 | 45.900 | 1.800 | 31.930 | 45.900 | 31.930 | - |
| 75th percentile | 54.550 | 54.550 | 79.250 | 31.930 | 54.550 | 54.550 | 79.250 | - |
| Minimum | 1.800 | 1.800 | 1.800 | 1.800 | 1.800 | 1.800 | 31.930 | - |
| Maximum | 79.250 | 79.250 | 79.250 | 45.900 | 79.250 | 79.250 | 79.250 | - |
| SF      Mean | 68.693 | 65.567 | 65.210 | 42.534 | 83.281 | 59.533 | 83.166 | - |
| Standard deviation | 25.066 | 21.360 | 16.653 | 12.881 | 15.982 | 41.522 | 15.139 | - |
| Median | 66.870 | 48.870 | 66.870 | 48.870 | 99.320 | 66.870 | 99.320 | - |
| 25th percentile | 48.870 | 48.870 | 48.870 | 24.910 | 66.870 | 24.910 | 66.870 | - |
| 75th percentile | 99.320 | 99.320 | 66.870 | 48.870 | 99.320 | 99.320 | 99.320 | - |
| Minimum | 2.780 | 48.870 | 24.910 | 2.780 | 2.780 | 2.780 | 24.910 | - |
| Maximum | 99.320 | 99.320 | 99.320 | 66.870 | 99.320 | 99.320 | 99.320 | - |
| MH   Mean | 63.272 | 37.490 | 57.526 | 34.174 | 72.647 | 70.935 | 86.029 | - |
| Standard deviation | 20.853 | 27.915 | 13.490 | 8.611 | 14.183 | 19.207 | 8.958 | - |
| Median | 74.485 | 41.685 | 60.685 | 41.440 | 83.670 | 74.485 | 92.650 | - |
| 25th percentile | 51.045 | 6.005 | 40.305 | 26.385 | 60.685 | 65.505 | 83.670 | - |
| 75th percentile | 83.670 | 60.685 | 69.490 | 41.685 | 83.845 | 83.670 | 92.650 | - |
| Minimum | 6.005 | 6.005 | 26.385 | 6.005 | 23.910 | 6.005 | 55.620 | - |
| Maximum | 98.595 | 83.670 | 74.865 | 51.325 | 92.650 | 92.650 | 98.595 | - |
| RE     Mean | 71.214 | 61.708 | 62.748 | 38.603 | 80.774 | 72.721 | 81.211 | - |
| Standard deviation | 26.671 | 30.154 | 19.272 | 20.435 | 22.161 | 35.901 | 19.241 | - |
| Median | 81.810 | 81.810 | 54.690 | 35.050 | 81.810 | 81.810 | 104.125 | - |
| 25th percentile | 54.690 | 41.285 | 54.690 | 29.335 | 68.370 | 68.370 | 41.285 | - |
| 75th percentile | 104.125 | 81.810 | 81.810 | 54.690 | 104.125 | 104.125 | 104.125 | - |
| Minimum | -33.415 | -13.835 | 29.335 | -20.070 | -33.415 | -13.835 | 29.335 | - |
| Maximum | 104.125 | 81.810 | 104.125 | 77.005 | 104.125 | 104.125 | 104.125 | - |

BP, bodily pain; GH, general health; MCS, mental component summary; MH, mental health; PCS, physical component summary; PF, physical functioning; RE, role emotional; RP, role physical; VT, vitality; SF, social functioning

**SM3 Table 25**. Canadian norms for VR-12 health utility values, summary component scores, and domain scores, by age group – Newfoundland.

|  | All participants (n=90) | 18 to 29 (n=11) | 30 to 39 (n=16) | 40 to 49 (n=20) | 50 to 59 (n=19) | 60 to 69 (n=16) | 70 to 79 (n=7) | 80 and over (n=1) |
| --- | --- | --- | --- | --- | --- | --- | --- | --- |
| *VR-12 health utility values* |  |  |  |  |  |  |  |  |
| Mean | 0.756 | 0.670 | 0.747 | 0.692 | 0.781 | 0.860 | 0.532 | - |
| Standard deviation | 0.172 | 0.171 | 0.192 | 0.165 | 0.106 | 0.143 | 0.141 | - |
| Median | 0.810 | 0.706 | 0.818 | 0.727 | 0.810 | 0.869 | 0.568 | - |
| 25th percentile | 0.706 | 0.706 | 0.696 | 0.553 | 0.726 | 0.810 | 0.365 | - |
| 75th percentile | 0.852 | 0.706 | 0.818 | 0.852 | 0.869 | 0.952 | 0.644 | - |
| Minimum | 0.142 | 0.142 | 0.220 | 0.301 | 0.456 | 0.457 | 0.250 | - |
| Maximum | 0.952 | 0.868 | 0.913 | 0.913 | 0.952 | 0.952 | 0.913 | - |
| *VR-12 summary component score* |  |  |  |  |  |  |  |  |
| PCS   Mean | 52.301 | 54.066 | 55.987 | 52.346 | 50.004 | 51.409 | 35.016 | - |
| Standard deviation | 8.352 | 7.545 | 5.914 | 6.544 | 6.998 | 10.647 | 5.318 | - |
| Median | 56.691 | 57.024 | 56.691 | 55.921 | 52.562 | 53.355 | 32.151 | - |
| 25th percentile | 48.250 | 53.033 | 56.691 | 49.094 | 45.804 | 48.167 | 31.667 | - |
| 75th percentile | 57.155 | 57.024 | 58.132 | 58.044 | 55.963 | 57.155 | 37.797 | - |
| Minimum | 24.065 | 32.996 | 38.946 | 24.065 | 28.392 | 24.692 | 26.707 | - |
| Maximum | 61.452 | 61.452 | 61.034 | 60.325 | 58.769 | 58.016 | 54.838 | - |
| MCS  Mean | 51.621 | 40.282 | 48.690 | 51.358 | 52.655 | 59.668 | 49.030 | - |
| Standard deviation | 10.130 | 8.449 | 13.293 | 5.847 | 7.505 | 3.189 | 7.134 | - |
| Median | 54.556 | 35.391 | 54.556 | 50.089 | 55.070 | 60.202 | 53.555 | - |
| 25th percentile | 45.792 | 35.391 | 45.632 | 45.512 | 44.005 | 59.458 | 38.566 | - |
| 75th percentile | 59.603 | 48.242 | 54.556 | 58.376 | 60.217 | 61.487 | 56.482 | - |
| Minimum | 21.542 | 33.908 | 21.542 | 32.789 | 33.091 | 53.439 | 33.534 | - |
| Maximum | 62.865 | 56.581 | 57.666 | 60.330 | 62.031 | 62.865 | 59.608 | - |
| *VR-12 domain scores* |  |  |  |  |  |  |  |  |
| PF     Mean | 83.267 | 82.644 | 89.696 | 77.668 | 83.308 | 84.607 | 57.304 | - |
| Standard deviation | 20.127 | 30.031 | 13.607 | 19.850 | 15.733 | 21.106 | 15.307 | - |
| Median | 93.485 | 93.485 | 93.485 | 93.485 | 93.485 | 93.485 | 58.290 | - |
| 25th percentile | 80.375 | 93.485 | 93.485 | 58.290 | 80.375 | 80.375 | 36.595 | - |
| 75th percentile | 93.485 | 93.485 | 93.485 | 93.485 | 93.485 | 93.485 | 80.375 | - |
| Minimum | 7.765 | 7.765 | 58.290 | 7.765 | 7.765 | 7.765 | 29.460 | - |
| Maximum | 93.485 | 93.485 | 93.485 | 93.485 | 93.485 | 93.485 | 93.485 | - |
| RP     Mean | 85.681 | 91.517 | 94.100 | 88.410 | 74.795 | 87.709 | 15.860 | - |
| Standard deviation | 29.190 | 26.570 | 18.123 | 21.460 | 30.869 | 31.147 | 18.255 | - |
| Median | 100.690 | 100.690 | 100.690 | 100.690 | 89.670 | 100.690 | 9.775 | - |
| 25th percentile | 89.670 | 100.690 | 89.670 | 79.405 | 79.405 | 79.405 | 9.775 | - |
| 75th percentile | 100.690 | 100.690 | 100.690 | 100.690 | 100.690 | 100.690 | 9.775 | - |
| Minimum | 0.490 | 0.490 | 44.460 | 5.020 | 0.490 | 5.020 | 5.020 | - |
| Maximum | 100.690 | 100.690 | 100.690 | 100.690 | 100.690 | 100.690 | 100.690 | - |
| BP     Mean | 73.221 | 50.017 | 86.224 | 74.827 | 66.894 | 79.222 | 41.473 | - |
| Standard deviation | 24.796 | 14.098 | 21.971 | 22.794 | 18.324 | 28.017 | 12.412 | - |
| Median | 95.200 | 43.890 | 95.200 | 95.200 | 56.250 | 95.200 | 43.890 | - |
| 25th percentile | 56.250 | 43.890 | 95.200 | 43.890 | 56.250 | 56.250 | 31.500 | - |
| 75th percentile | 95.200 | 56.250 | 95.200 | 95.200 | 95.200 | 95.200 | 43.890 | - |
| Minimum | 31.500 | 31.500 | 43.890 | 31.500 | 31.500 | 31.500 | 31.500 | - |
| Maximum | 95.200 | 95.200 | 95.200 | 95.200 | 95.200 | 95.200 | 95.200 | - |
| GH    Mean | 79.672 | 81.579 | 77.230 | 82.039 | 73.437 | 85.903 | 55.671 | - |
| Standard deviation | 20.169 | 14.658 | 27.122 | 14.287 | 14.097 | 26.223 | 14.872 | - |
| Median | 83.710 | 83.710 | 83.710 | 83.710 | 83.710 | 83.710 | 61.500 | - |
| 25th percentile | 83.710 | 83.710 | 83.710 | 83.710 | 61.500 | 83.710 | 37.500 | - |
| 75th percentile | 83.710 | 83.710 | 83.710 | 83.710 | 83.710 | 101.840 | 83.710 | - |
| Minimum | 0.580 | 37.500 | 0.580 | 37.500 | 0.580 | 37.500 | 37.500 | - |
| Maximum | 101.840 | 101.840 | 101.840 | 101.840 | 83.710 | 101.840 | 83.710 | - |
| VT     Mean | 56.650 | 56.399 | 47.372 | 59.157 | 60.702 | 64.753 | 39.238 | - |
| Standard deviation | 19.938 | 22.505 | 18.091 | 19.796 | 14.918 | 23.059 | 9.809 | - |
| Median | 54.550 | 45.900 | 45.900 | 54.550 | 54.550 | 79.250 | 31.930 | - |
| 25th percentile | 45.900 | 45.900 | 45.900 | 54.550 | 45.900 | 45.900 | 31.930 | - |
| 75th percentile | 79.250 | 79.250 | 45.900 | 79.250 | 79.250 | 79.250 | 45.900 | - |
| Minimum | 1.800 | 1.800 | 1.800 | 1.800 | 31.930 | 1.800 | 31.930 | - |
| Maximum | 95.340 | 95.340 | 79.250 | 79.250 | 79.250 | 79.250 | 79.250 | - |
| SF      Mean | 79.113 | 52.181 | 79.269 | 77.255 | 77.343 | 95.574 | 55.068 | - |
| Standard deviation | 27.035 | 11.419 | 38.734 | 23.646 | 17.995 | 13.862 | 20.636 | - |
| Median | 99.320 | 48.870 | 99.320 | 99.320 | 66.870 | 99.320 | 66.870 | - |
| 25th percentile | 66.870 | 48.870 | 48.870 | 66.870 | 66.870 | 99.320 | 48.870 | - |
| 75th percentile | 99.320 | 66.870 | 99.320 | 99.320 | 99.320 | 99.320 | 66.870 | - |
| Minimum | 2.780 | 24.910 | 2.780 | 24.910 | 48.870 | 66.870 | 2.780 | - |
| Maximum | 99.320 | 66.870 | 99.320 | 99.320 | 99.320 | 99.320 | 99.320 | - |
| MH   Mean | 75.436 | 56.060 | 71.724 | 71.041 | 75.468 | 92.962 | 61.115 | - |
| Standard deviation | 21.317 | 18.409 | 25.076 | 15.576 | 17.879 | 8.816 | 16.538 | - |
| Median | 83.670 | 51.325 | 83.670 | 74.485 | 74.865 | 92.650 | 69.490 | - |
| 25th percentile | 60.685 | 51.325 | 60.685 | 55.055 | 60.685 | 92.650 | 41.685 | - |
| 75th percentile | 92.650 | 60.685 | 83.670 | 92.650 | 98.595 | 98.595 | 74.865 | - |
| Minimum | 6.005 | 21.060 | 6.005 | 26.385 | 30.945 | 65.505 | 21.060 | - |
| Maximum | 98.595 | 92.650 | 83.845 | 92.650 | 98.595 | 98.595 | 92.650 | - |
| RE     Mean | 82.578 | 42.523 | 82.619 | 83.692 | 85.175 | 100.524 | 78.550 | - |
| Standard deviation | 34.259 | 37.155 | 44.777 | 22.866 | 28.264 | 15.951 | 22.901 | - |
| Median | 104.125 | 29.335 | 104.125 | 104.125 | 104.125 | 104.125 | 104.125 | - |
| 25th percentile | 65.090 | 29.335 | 54.690 | 77.005 | 81.810 | 104.125 | 54.690 | - |
| 75th percentile | 104.125 | 65.090 | 104.125 | 104.125 | 104.125 | 104.125 | 104.125 | - |
| Minimum | -13.835 | -13.835 | -3.985 | 29.335 | 2.250 | 29.335 | 29.335 | - |
| Maximum | 104.125 | 104.125 | 104.125 | 104.125 | 104.125 | 104.125 | 104.125 | - |

BP, bodily pain; GH, general health; MCS, mental component summary; MH, mental health; PCS, physical component summary; PF, physical functioning; RE, role emotional; RP, role physical; VT, vitality; SF, social functioning

**SM3 Table 26**. Canadian norms for VR-12 health utility values, summary component scores, and domain scores, by age group – Newfoundland, males only.

|  | All participants (n=44) | 18 to 29 (n=6) | 30 to 39 (n=10) | 40 to 49 (n=8) | 50 to 59 (n=9) | 60 to 69 (n=5) | 70 to 79 (n=5) | 80 and over (n=1) |
| --- | --- | --- | --- | --- | --- | --- | --- | --- |
| *VR-12 health utility values* |  |  |  |  |  |  |  |  |
| Mean | 0.766 | 0.721 | 0.783 | 0.717 | 0.814 | 0.823 | 0.644 | - |
| Standard deviation | 0.134 | 0.094 | 0.196 | 0.148 | 0.087 | 0.074 | 0.079 | - |
| Median | 0.818 | 0.706 | 0.818 | 0.746 | 0.810 | 0.852 | 0.644 | - |
| 25th percentile | 0.706 | 0.706 | 0.818 | 0.553 | 0.726 | 0.778 | 0.568 | - |
| 75th percentile | 0.852 | 0.706 | 0.818 | 0.852 | 0.912 | 0.869 | 0.664 | - |
| Minimum | 0.220 | 0.569 | 0.220 | 0.421 | 0.535 | 0.703 | 0.568 | - |
| Maximum | 0.952 | 0.868 | 0.913 | 0.913 | 0.952 | 0.869 | 0.913 | - |
| *VR-12 summary component score* |  |  |  |  |  |  |  |  |
| PCS   Mean | 53.193 | 55.474 | 56.823 | 54.173 | 50.839 | 48.629 | 34.540 | - |
| Standard deviation | 8.096 | 5.775 | 4.470 | 6.689 | 6.440 | 9.278 | 5.979 | - |
| Median | 56.691 | 57.024 | 56.691 | 59.739 | 53.618 | 48.167 | 32.151 | - |
| 25th percentile | 50.677 | 57.024 | 56.691 | 42.873 | 45.804 | 40.730 | 31.667 | - |
| 75th percentile | 57.024 | 57.024 | 58.769 | 59.750 | 55.862 | 57.908 | 37.797 | - |
| Minimum | 26.707 | 40.561 | 38.946 | 41.390 | 29.488 | 38.141 | 26.707 | - |
| Maximum | 61.034 | 57.410 | 61.034 | 60.325 | 58.769 | 57.908 | 54.838 | - |
| MCS  Mean | 51.092 | 40.334 | 52.136 | 49.371 | 54.730 | 58.767 | 55.210 | - |
| Standard deviation | 9.507 | 11.086 | 10.064 | 5.224 | 6.942 | 1.710 | 2.224 | - |
| Median | 54.556 | 35.391 | 54.556 | 48.038 | 59.303 | 59.641 | 56.482 | - |
| 25th percentile | 46.958 | 35.391 | 51.433 | 45.005 | 45.792 | 56.980 | 53.555 | - |
| 75th percentile | 56.980 | 48.242 | 54.556 | 55.756 | 60.217 | 59.896 | 58.262 | - |
| Minimum | 23.022 | 35.391 | 23.022 | 38.604 | 43.920 | 56.980 | 50.700 | - |
| Maximum | 62.031 | 56.581 | 57.666 | 57.000 | 62.031 | 60.202 | 59.608 | - |
| *VR-12 domain scores* |  |  |  |  |  |  |  |  |
| PF     Mean | 86.342 | 87.514 | 92.818 | 82.158 | 84.922 | 81.179 | 68.917 | - |
| Standard deviation | 16.639 | 29.217 | 7.037 | 16.092 | 15.483 | 14.317 | 10.105 | - |
| Median | 93.485 | 93.485 | 93.485 | 93.485 | 93.485 | 80.375 | 58.290 | - |
| 25th percentile | 80.375 | 93.485 | 93.485 | 58.290 | 80.375 | 80.375 | 58.290 | - |
| 75th percentile | 93.485 | 93.485 | 93.485 | 93.485 | 93.485 | 93.485 | 80.375 | - |
| Minimum | 7.765 | 7.765 | 58.290 | 49.705 | 7.765 | 58.290 | 58.290 | - |
| Maximum | 93.485 | 93.485 | 93.485 | 93.485 | 93.485 | 93.485 | 93.485 | - |
| RP     Mean | 88.793 | 99.004 | 99.186 | 81.019 | 84.232 | 80.079 | 18.813 | - |
| Standard deviation | 27.507 | 7.626 | 6.299 | 28.868 | 27.376 | 24.063 | 21.999 | - |
| Median | 100.690 | 100.690 | 100.690 | 100.690 | 100.690 | 79.405 | 9.775 | - |
| 25th percentile | 89.670 | 100.690 | 100.690 | 44.720 | 79.405 | 54.725 | 9.775 | - |
| 75th percentile | 100.690 | 100.690 | 100.690 | 100.690 | 100.690 | 100.690 | 9.775 | - |
| Minimum | 5.020 | 79.405 | 79.405 | 9.775 | 5.245 | 54.725 | 5.020 | - |
| Maximum | 100.690 | 100.690 | 100.690 | 100.690 | 100.690 | 100.690 | 100.690 | - |
| BP     Mean | 72.855 | 47.621 | 89.435 | 78.708 | 66.300 | 78.797 | 43.603 | - |
| Standard deviation | 25.990 | 7.983 | 20.912 | 23.427 | 17.982 | 25.660 | 14.554 | - |
| Median | 95.200 | 43.890 | 95.200 | 95.200 | 56.250 | 95.200 | 43.890 | - |
| 25th percentile | 56.250 | 43.890 | 95.200 | 43.890 | 56.250 | 56.250 | 31.500 | - |
| 75th percentile | 95.200 | 56.250 | 95.200 | 95.200 | 95.200 | 95.200 | 43.890 | - |
| Minimum | 31.500 | 43.890 | 43.890 | 31.500 | 43.890 | 56.250 | 31.500 | - |
| Maximum | 95.200 | 56.250 | 95.200 | 95.200 | 95.200 | 95.200 | 95.200 | - |
| GH    Mean | 79.065 | 84.760 | 82.596 | 82.499 | 76.095 | 71.198 | 47.984 | - |
| Standard deviation | 17.179 | 15.165 | 11.017 | 14.506 | 14.782 | 27.398 | 13.741 | - |
| Median | 83.710 | 83.710 | 83.710 | 83.710 | 83.710 | 83.710 | 37.500 | - |
| 25th percentile | 83.710 | 83.710 | 83.710 | 61.500 | 61.500 | 37.500 | 37.500 | - |
| 75th percentile | 83.710 | 83.710 | 83.710 | 101.840 | 83.710 | 83.710 | 61.500 | - |
| Minimum | 0.580 | 61.500 | 61.500 | 61.500 | 0.580 | 37.500 | 37.500 | - |
| Maximum | 101.840 | 101.840 | 101.840 | 101.840 | 83.710 | 83.710 | 83.710 | - |
| VT     Mean | 55.845 | 56.710 | 49.382 | 63.391 | 68.848 | 58.024 | 42.785 | - |
| Standard deviation | 19.132 | 25.842 | 21.465 | 16.545 | 14.461 | 18.694 | 11.019 | - |
| Median | 45.900 | 45.900 | 45.900 | 79.250 | 79.250 | 54.550 | 45.900 | - |
| 25th percentile | 45.900 | 45.900 | 45.900 | 45.900 | 45.900 | 45.900 | 31.930 | - |
| 75th percentile | 79.250 | 79.250 | 45.900 | 79.250 | 79.250 | 79.250 | 45.900 | - |
| Minimum | 1.800 | 45.900 | 1.800 | 31.930 | 45.900 | 45.900 | 31.930 | - |
| Maximum | 95.340 | 95.340 | 79.250 | 79.250 | 79.250 | 79.250 | 79.250 | - |
| SF      Mean | 80.958 | 50.844 | 91.045 | 82.723 | 79.191 | 94.927 | 70.368 | - |
| Standard deviation | 24.839 | 7.913 | 27.039 | 16.853 | 20.333 | 14.814 | 7.765 | - |
| Median | 99.320 | 48.870 | 99.320 | 99.320 | 99.320 | 99.320 | 66.870 | - |
| 25th percentile | 66.870 | 48.870 | 99.320 | 66.870 | 66.870 | 99.320 | 66.870 | - |
| 75th percentile | 99.320 | 48.870 | 99.320 | 99.320 | 99.320 | 99.320 | 66.870 | - |
| Minimum | 24.910 | 48.870 | 24.910 | 48.870 | 48.870 | 66.870 | 66.870 | - |
| Maximum | 99.320 | 66.870 | 99.320 | 99.320 | 99.320 | 99.320 | 99.320 | - |
| MH   Mean | 74.172 | 58.634 | 78.158 | 65.106 | 78.543 | 88.290 | 75.058 | - |
| Standard deviation | 19.412 | 20.045 | 23.697 | 13.778 | 18.851 | 5.614 | 5.061 | - |
| Median | 83.670 | 51.325 | 83.670 | 74.485 | 92.650 | 89.615 | 74.865 | - |
| 25th percentile | 65.505 | 51.325 | 74.865 | 41.440 | 51.325 | 83.670 | 69.490 | - |
| 75th percentile | 83.670 | 60.685 | 83.670 | 74.865 | 92.650 | 92.650 | 74.865 | - |
| Minimum | 6.005 | 51.045 | 6.005 | 41.440 | 51.045 | 83.670 | 69.490 | - |
| Maximum | 98.595 | 92.650 | 83.845 | 83.670 | 98.595 | 92.650 | 92.650 | - |
| RE     Mean | 83.732 | 45.243 | 93.486 | 74.819 | 94.670 | 104.125 | 95.677 | - |
| Standard deviation | 35.267 | 43.743 | 36.255 | 29.525 | 22.313 | 0.000 | 14.359 | - |
| Median | 104.125 | 29.335 | 104.125 | 104.125 | 104.125 | 104.125 | 104.125 | - |
| 25th percentile | 54.690 | 29.335 | 104.125 | 29.335 | 104.125 | 104.125 | 104.125 | - |
| 75th percentile | 104.125 | 29.335 | 104.125 | 104.125 | 104.125 | 104.125 | 104.125 | - |
| Minimum | 14.165 | 14.165 | 14.165 | 29.335 | 29.335 | 104.125 | 54.690 | - |
| Maximum | 104.125 | 104.125 | 104.125 | 104.125 | 104.125 | 104.125 | 104.125 | - |

BP, bodily pain; GH, general health; MCS, mental component summary; MH, mental health; PCS, physical component summary; PF, physical functioning; RE, role emotional; RP, role physical; VT, vitality; SF, social functioning

**SM3 Table 27**. Canadian norms for VR-12 health utility values, summary component scores, and domain scores, by age group – Newfoundland, females only.

|  | All participants (n=46) | 18 to 29 (n=5) | 30 to 39 (n=6) | 40 to 49 (n=12) | 50 to 59 (n=10) | 60 to 69 (n=11) | 70 to 79 (n=2) | 80 and over (n=0) |
| --- | --- | --- | --- | --- | --- | --- | --- | --- |
| *VR-12 health utility values* |  |  |  |  |  |  |  |  |
| Mean | 0.744 | 0.491 | 0.645 | 0.677 | 0.745 | 0.873 | - | - |
| Standard deviation | 0.203 | 0.181 | 0.152 | 0.180 | 0.118 | 0.165 | - | - |
| Median | 0.765 | 0.486 | 0.664 | 0.726 | 0.755 | 0.952 | - | - |
| 25th percentile | 0.644 | 0.456 | 0.644 | 0.600 | 0.644 | 0.810 | - | - |
| 75th percentile | 0.952 | 0.639 | 0.736 | 0.879 | 0.869 | 0.952 | - | - |
| Minimum | 0.142 | 0.142 | 0.423 | 0.301 | 0.456 | 0.457 | - | - |
| Maximum | 0.952 | 0.716 | 0.818 | 0.913 | 0.952 | 0.952 | - | - |
| *VR-12 summary component score* |  |  |  |  |  |  |  |  |
| PCS   Mean | 51.279 | 49.168 | 53.571 | 51.302 | 49.080 | 52.414 | - | - |
| Standard deviation | 8.562 | 8.869 | 7.646 | 6.550 | 7.740 | 11.319 | - | - |
| Median | 55.316 | 52.867 | 57.868 | 53.077 | 48.250 | 57.155 | - | - |
| 25th percentile | 47.758 | 38.932 | 47.758 | 49.094 | 40.898 | 50.994 | - | - |
| 75th percentile | 57.155 | 53.033 | 58.132 | 56.255 | 57.307 | 57.155 | - | - |
| Minimum | 24.065 | 32.996 | 39.905 | 24.065 | 28.392 | 24.692 | - | - |
| Maximum | 61.452 | 61.452 | 59.244 | 58.044 | 57.577 | 58.016 | - | - |
| MCS  Mean | 52.227 | 40.098 | 38.726 | 52.492 | 50.357 | 59.994 | - | - |
| Standard deviation | 10.765 | 4.981 | 12.996 | 6.215 | 7.911 | 3.647 | - | - |
| Median | 55.480 | 37.260 | 39.573 | 51.488 | 49.429 | 61.487 | - | - |
| 25th percentile | 45.632 | 34.036 | 33.350 | 49.726 | 42.460 | 59.576 | - | - |
| 75th percentile | 61.487 | 41.072 | 51.250 | 59.588 | 61.499 | 61.487 | - | - |
| Minimum | 21.542 | 33.908 | 21.542 | 32.789 | 33.091 | 53.439 | - | - |
| Maximum | 62.865 | 49.592 | 52.168 | 60.330 | 61.906 | 62.865 | - | - |
| *VR-12 domain scores* |  |  |  |  |  |  |  |  |
| PF     Mean | 79.746 | 65.703 | 80.665 | 75.103 | 81.519 | 85.847 | - | - |
| Standard deviation | 22.642 | 30.410 | 17.872 | 22.364 | 16.662 | 23.976 | - | - |
| Median | 93.485 | 80.375 | 93.485 | 93.485 | 93.485 | 93.485 | - | - |
| 25th percentile | 71.400 | 29.460 | 58.290 | 58.290 | 58.290 | 80.375 | - | - |
| 75th percentile | 93.485 | 93.485 | 93.485 | 93.485 | 93.485 | 93.485 | - | - |
| Minimum | 7.765 | 7.765 | 58.290 | 7.765 | 29.460 | 7.765 | - | - |
| Maximum | 93.485 | 93.485 | 93.485 | 93.485 | 93.485 | 93.485 | - | - |
| RP     Mean | 82.119 | 65.474 | 79.392 | 92.631 | 64.343 | 90.469 | - | - |
| Standard deviation | 30.622 | 32.727 | 22.895 | 14.924 | 33.014 | 34.164 | - | - |
| Median | 100.690 | 79.405 | 89.670 | 100.690 | 79.405 | 100.690 | - | - |
| 25th percentile | 79.405 | 44.460 | 54.725 | 89.670 | 9.775 | 89.670 | - | - |
| 75th percentile | 100.690 | 89.670 | 90.425 | 100.690 | 100.690 | 100.690 | - | - |
| Minimum | 0.490 | 0.490 | 44.460 | 5.020 | 0.490 | 5.020 | - | - |
| Maximum | 100.690 | 100.690 | 100.690 | 100.690 | 100.690 | 100.690 | - | - |
| BP     Mean | 73.640 | 58.350 | 76.940 | 72.610 | 67.552 | 79.376 | - | - |
| Standard deviation | 23.879 | 18.804 | 22.489 | 23.162 | 19.584 | 30.230 | - | - |
| Median | 95.200 | 56.250 | 95.200 | 95.200 | 56.250 | 95.200 | - | - |
| 25th percentile | 56.250 | 43.890 | 56.250 | 56.250 | 43.890 | 56.250 | - | - |
| 75th percentile | 95.200 | 56.250 | 95.200 | 95.200 | 95.200 | 95.200 | - | - |
| Minimum | 31.500 | 31.500 | 56.250 | 31.500 | 31.500 | 31.500 | - | - |
| Maximum | 95.200 | 95.200 | 95.200 | 95.200 | 95.200 | 95.200 | - | - |
| GH    Mean | 80.367 | 70.514 | 61.715 | 81.777 | 70.493 | 91.222 | - | - |
| Standard deviation | 22.838 | 11.730 | 39.428 | 14.781 | 13.833 | 22.837 | - | - |
| Median | 83.710 | 61.500 | 83.710 | 83.710 | 83.710 | 101.840 | - | - |
| 25th percentile | 61.500 | 61.500 | 61.500 | 83.710 | 61.500 | 83.710 | - | - |
| 75th percentile | 101.840 | 83.710 | 83.710 | 83.710 | 83.710 | 101.840 | - | - |
| Minimum | 0.580 | 37.500 | 0.580 | 37.500 | 37.500 | 37.500 | - | - |
| Maximum | 101.840 | 83.710 | 83.710 | 101.840 | 83.710 | 101.840 | - | - |
| VT     Mean | 57.571 | 55.320 | 41.560 | 56.738 | 51.680 | 67.188 | - | - |
| Standard deviation | 20.852 | 20.744 | 9.572 | 22.108 | 12.152 | 24.776 | - | - |
| Median | 54.550 | 45.900 | 45.900 | 54.550 | 54.550 | 79.250 | - | - |
| 25th percentile | 45.900 | 31.930 | 31.930 | 54.550 | 45.900 | 54.550 | - | - |
| 75th percentile | 79.250 | 79.250 | 45.900 | 79.250 | 54.550 | 79.250 | - | - |
| Minimum | 1.800 | 1.800 | 31.930 | 1.800 | 31.930 | 1.800 | - | - |
| Maximum | 79.250 | 79.250 | 54.550 | 79.250 | 79.250 | 79.250 | - | - |
| SF      Mean | 77.000 | 56.833 | 45.216 | 74.132 | 75.296 | 95.809 | - | - |
| Standard deviation | 29.109 | 15.097 | 33.100 | 27.600 | 16.565 | 14.143 | - | - |
| Median | 99.320 | 66.870 | 48.870 | 99.320 | 66.870 | 99.320 | - | - |
| 25th percentile | 66.870 | 66.870 | 48.870 | 66.870 | 66.870 | 99.320 | - | - |
| 75th percentile | 99.320 | 66.870 | 48.870 | 99.320 | 99.320 | 99.320 | - | - |
| Minimum | 2.780 | 24.910 | 2.780 | 24.910 | 48.870 | 66.870 | - | - |
| Maximum | 99.320 | 66.870 | 99.320 | 99.320 | 99.320 | 99.320 | - | - |
| MH   Mean | 76.883 | 47.108 | 53.121 | 74.430 | 72.064 | 94.652 | - | - |
| Standard deviation | 23.122 | 16.478 | 15.878 | 16.460 | 17.537 | 9.100 | - | - |
| Median | 83.845 | 40.305 | 51.325 | 74.865 | 65.505 | 98.595 | - | - |
| 25th percentile | 60.685 | 31.800 | 41.685 | 65.505 | 60.685 | 92.650 | - | - |
| 75th percentile | 98.595 | 55.620 | 60.685 | 92.650 | 98.595 | 98.595 | - | - |
| Minimum | 21.060 | 21.060 | 41.440 | 26.385 | 30.945 | 65.505 | - | - |
| Maximum | 98.595 | 74.865 | 83.670 | 92.650 | 98.595 | 98.595 | - | - |
| RE     Mean | 81.257 | 33.062 | 51.193 | 88.760 | 74.658 | 99.221 | - | - |
| Standard deviation | 33.607 | 31.263 | 43.240 | 16.949 | 31.681 | 19.211 | - | - |
| Median | 104.125 | 13.250 | 54.690 | 104.125 | 104.125 | 104.125 | - | - |
| 25th percentile | 77.005 | 2.250 | 29.335 | 77.005 | 29.335 | 104.125 | - | - |
| 75th percentile | 104.125 | 65.090 | 81.810 | 104.125 | 104.125 | 104.125 | - | - |
| Minimum | -13.835 | -13.835 | -3.985 | 41.250 | 2.250 | 29.335 | - | - |
| Maximum | 104.125 | 77.005 | 104.125 | 104.125 | 104.125 | 104.125 | - | - |

BP, bodily pain; GH, general health; MCS, mental component summary; MH, mental health; PCS, physical component summary; PF, physical functioning; RE, role emotional; RP, role physical; VT, vitality; SF, social functioning

**SM3 Table 28**. Canadian norms for VR-12 health utility values, summary component scores, and domain scores, by age group – Prince Edward Island.

|  | All participants (n=41) | 18 to 29 (n=9) | 30 to 39 (n=5) | 40 to 49 (n=3) | 50 to 59 (n=9) | 60 to 69 (n=7) | 70 to 79 (n=8) | 80 and over (n=) |
| --- | --- | --- | --- | --- | --- | --- | --- | --- |
| *VR-12 health utility values* |  |  |  |  |  |  |  |  |
| Mean | 0.703 | 0.570 | 0.608 | - | 0.530 | 0.812 | 0.800 | - |
| Standard deviation | 0.203 | 0.163 | 0.220 | - | 0.264 | 0.175 | 0.054 | - |
| Median | 0.768 | 0.564 | 0.696 | - | 0.726 | 0.852 | 0.777 | - |
| 25th percentile | 0.696 | 0.346 | 0.696 | - | 0.269 | 0.852 | 0.768 | - |
| 75th percentile | 0.852 | 0.746 | 0.721 | - | 0.818 | 0.852 | 0.827 | - |
| Minimum | -0.590 | 0.319 | -0.132 | - | -0.590 | 0.365 | 0.644 | - |
| Maximum | 0.913 | 0.812 | 0.746 | - | 0.827 | 0.913 | 0.913 | - |
| *VR-12 summary component score* |  |  |  |  |  |  |  |  |
| PCS   Mean | 51.448 | 52.833 | 50.163 | - | 45.514 | 54.632 | 44.856 | - |
| Standard deviation | 8.507 | 10.129 | 6.924 | - | 7.510 | 11.104 | 4.695 | - |
| Median | 55.623 | 56.560 | 52.716 | - | 46.663 | 57.908 | 47.670 | - |
| 25th percentile | 46.721 | 47.718 | 52.716 | - | 44.091 | 57.908 | 42.267 | - |
| 75th percentile | 57.908 | 60.323 | 53.770 | - | 56.057 | 57.908 | 50.925 | - |
| Minimum | 21.716 | 34.776 | 27.651 | - | 21.716 | 31.203 | 33.880 | - |
| Maximum | 66.263 | 66.263 | 55.623 | - | 57.763 | 57.908 | 53.493 | - |
| MCS  Mean | 50.025 | 38.741 | 47.022 | - | 45.139 | 55.212 | 59.147 | - |
| Standard deviation | 9.776 | 9.377 | 7.309 | - | 9.337 | 8.750 | 3.157 | - |
| Median | 55.497 | 36.138 | 48.677 | - | 49.668 | 56.980 | 59.628 | - |
| 25th percentile | 45.457 | 28.652 | 45.748 | - | 31.391 | 56.980 | 57.016 | - |
| 75th percentile | 56.980 | 49.441 | 52.738 | - | 54.872 | 56.980 | 60.007 | - |
| Minimum | 20.625 | 22.357 | 21.914 | - | 20.625 | 34.704 | 45.320 | - |
| Maximum | 67.549 | 55.590 | 52.738 | - | 62.388 | 60.306 | 67.549 | - |
| *VR-12 domain scores* |  |  |  |  |  |  |  |  |
| PF     Mean | 81.888 | 82.828 | 83.554 | - | 75.584 | 87.253 | 62.008 | - |
| Standard deviation | 17.822 | 13.807 | 15.825 | - | 14.744 | 25.745 | 17.594 | - |
| Median | 93.485 | 93.485 | 93.485 | - | 80.375 | 93.485 | 71.400 | - |
| 25th percentile | 80.375 | 71.400 | 80.375 | - | 71.400 | 93.485 | 29.460 | - |
| 75th percentile | 93.485 | 93.485 | 93.485 | - | 93.485 | 93.485 | 80.375 | - |
| Minimum | 7.765 | 58.290 | 36.595 | - | 7.765 | 29.460 | 29.460 | - |
| Maximum | 93.485 | 93.485 | 93.485 | - | 93.485 | 93.485 | 93.485 | - |
| RP     Mean | 80.955 | 71.881 | 71.442 | - | 67.607 | 91.100 | 84.397 | - |
| Standard deviation | 31.577 | 40.728 | 29.203 | - | 28.943 | 42.318 | 14.375 | - |
| Median | 100.690 | 100.690 | 89.670 | - | 100.690 | 100.690 | 89.670 | - |
| 25th percentile | 79.405 | 9.775 | 45.835 | - | 44.460 | 100.690 | 79.405 | - |
| 75th percentile | 100.690 | 100.690 | 100.690 | - | 100.690 | 100.690 | 100.690 | - |
| Minimum | -3.670 | -3.645 | 0.490 | - | -3.670 | 0.490 | 44.460 | - |
| Maximum | 100.690 | 100.690 | 100.690 | - | 100.690 | 100.690 | 100.690 | - |
| BP     Mean | 74.130 | 64.894 | 67.387 | - | 51.960 | 89.134 | 67.707 | - |
| Standard deviation | 22.940 | 20.062 | 26.323 | - | 22.576 | 22.004 | 13.003 | - |
| Median | 95.200 | 56.250 | 56.250 | - | 56.250 | 95.200 | 56.250 | - |
| 25th percentile | 56.250 | 43.890 | 56.250 | - | 31.500 | 95.200 | 56.250 | - |
| 75th percentile | 95.200 | 95.200 | 95.200 | - | 95.200 | 95.200 | 95.200 | - |
| Minimum | 2.280 | 43.890 | 2.280 | - | 2.280 | 43.890 | 56.250 | - |
| Maximum | 95.200 | 95.200 | 95.200 | - | 95.200 | 95.200 | 95.200 | - |
| GH    Mean | 70.669 | 69.903 | 72.656 | - | 65.914 | 74.341 | 61.932 | - |
| Standard deviation | 18.550 | 21.325 | 15.740 | - | 15.051 | 29.337 | 13.498 | - |
| Median | 83.710 | 83.710 | 83.710 | - | 61.500 | 83.710 | 61.500 | - |
| 25th percentile | 61.500 | 37.500 | 61.500 | - | 61.500 | 83.710 | 37.500 | - |
| 75th percentile | 83.710 | 83.710 | 83.710 | - | 83.710 | 83.710 | 83.710 | - |
| Minimum | 0.580 | 37.500 | 37.500 | - | 0.580 | 0.580 | 37.500 | - |
| Maximum | 101.840 | 101.840 | 83.710 | - | 83.710 | 83.710 | 83.710 | - |
| VT     Mean | 55.580 | 47.923 | 54.583 | - | 31.227 | 68.983 | 55.032 | - |
| Standard deviation | 22.163 | 17.102 | 19.505 | - | 15.876 | 32.987 | 14.806 | - |
| Median | 54.550 | 45.900 | 45.900 | - | 31.930 | 79.250 | 45.900 | - |
| 25th percentile | 31.930 | 31.930 | 45.900 | - | 1.800 | 79.250 | 45.900 | - |
| 75th percentile | 79.250 | 54.550 | 79.250 | - | 54.550 | 79.250 | 54.550 | - |
| Minimum | 1.800 | 1.800 | 1.800 | - | 1.800 | 1.800 | 31.930 | - |
| Maximum | 95.340 | 79.250 | 79.250 | - | 79.250 | 79.250 | 95.340 | - |
| SF      Mean | 75.349 | 51.597 | 58.108 | - | 58.151 | 90.315 | 97.750 | - |
| Standard deviation | 28.148 | 26.735 | 28.908 | - | 25.006 | 31.969 | 6.418 | - |
| Median | 99.320 | 48.870 | 48.870 | - | 66.870 | 99.320 | 99.320 | - |
| 25th percentile | 48.870 | 24.910 | 24.910 | - | 24.910 | 99.320 | 99.320 | - |
| 75th percentile | 99.320 | 66.870 | 99.320 | - | 99.320 | 99.320 | 99.320 | - |
| Minimum | 2.780 | 2.780 | 24.910 | - | 2.780 | 24.910 | 48.870 | - |
| Maximum | 99.320 | 99.320 | 99.320 | - | 99.320 | 99.320 | 99.320 | - |
| MH   Mean | 70.677 | 45.511 | 70.777 | - | 60.285 | 81.387 | 87.329 | - |
| Standard deviation | 20.219 | 19.050 | 16.838 | - | 20.852 | 15.414 | 5.373 | - |
| Median | 83.670 | 31.800 | 74.865 | - | 74.865 | 83.670 | 83.845 | - |
| 25th percentile | 60.685 | 26.385 | 74.865 | - | 31.800 | 83.670 | 83.670 | - |
| 75th percentile | 83.670 | 74.865 | 83.845 | - | 74.865 | 83.670 | 92.650 | - |
| Minimum | -1.030 | 21.060 | 14.025 | - | -1.030 | 41.440 | 60.685 | - |
| Maximum | 98.595 | 83.670 | 83.845 | - | 98.595 | 92.650 | 98.595 | - |
| RE     Mean | 80.763 | 47.428 | 62.223 | - | 71.790 | 97.909 | 99.146 | - |
| Standard deviation | 32.219 | 30.446 | 31.723 | - | 29.279 | 33.746 | 9.292 | - |
| Median | 104.125 | 29.335 | 54.690 | - | 77.005 | 104.125 | 104.125 | - |
| 25th percentile | 54.690 | 14.165 | 54.690 | - | 41.250 | 104.125 | 104.125 | - |
| 75th percentile | 104.125 | 81.810 | 104.125 | - | 104.125 | 104.125 | 104.125 | - |
| Minimum | -20.070 | 2.250 | -0.095 | - | -20.070 | 2.250 | 42.775 | - |
| Maximum | 104.125 | 104.125 | 104.125 | - | 104.125 | 104.125 | 104.125 | - |

BP, bodily pain; GH, general health; MCS, mental component summary; MH, mental health; PCS, physical component summary; PF, physical functioning; RE, role emotional; RP, role physical; VT, vitality; SF, social functioning

**SM3 Table 29**. Canadian norms for VR-12 health utility values, summary component scores, and domain scores, by age group – Prince Edward Island, males only.

|  | All participants (n=23) | 18 to 29 (n=5) | 30 to 39 (n=3) | 40 to 49 (n=1) | 50 to 59 (n=4) | 60 to 69 (n=3) | 70 to 79 (n=7) | 80 and over (n=) |
| --- | --- | --- | --- | --- | --- | --- | --- | --- |
| *VR-12 health utility values* |  |  |  |  |  |  |  |  |
| Mean | 0.698 | 0.486 | - | - | - | - | 0.817 | - |
| Standard deviation | 0.129 | 0.140 | - | - | - | - | 0.049 | - |
| Median | 0.746 | 0.346 | - | - | - | - | 0.777 | - |
| 25th percentile | 0.696 | 0.346 | - | - | - | - | 0.768 | - |
| 75th percentile | 0.777 | 0.706 | - | - | - | - | 0.908 | - |
| Minimum | 0.275 | 0.346 | - | - | - | - | 0.644 | - |
| Maximum | 0.913 | 0.765 | - | - | - | - | 0.913 | - |
| *VR-12 summary component score* |  |  |  |  |  |  |  |  |
| PCS   Mean | 48.370 | 44.376 | - | - | - | - | 45.002 | - |
| Standard deviation | 6.372 | 8.868 | - | - | - | - | 5.063 | - |
| Median | 51.266 | 34.776 | - | - | - | - | 47.670 | - |
| 25th percentile | 42.825 | 34.776 | - | - | - | - | 42.267 | - |
| 75th percentile | 54.587 | 57.405 | - | - | - | - | 50.925 | - |
| Minimum | 31.203 | 34.776 | - | - | - | - | 33.880 | - |
| Maximum | 57.960 | 57.960 | - | - | - | - | 53.493 | - |
| MCS  Mean | 49.628 | 36.925 | - | - | - | - | 59.724 | - |
| Standard deviation | 7.282 | 3.125 | - | - | - | - | 3.213 | - |
| Median | 51.508 | 36.138 | - | - | - | - | 59.946 | - |
| 25th percentile | 45.320 | 36.138 | - | - | - | - | 57.016 | - |
| 75th percentile | 57.016 | 36.918 | - | - | - | - | 60.007 | - |
| Minimum | 25.529 | 25.529 | - | - | - | - | 45.320 | - |
| Maximum | 67.549 | 47.135 | - | - | - | - | 67.549 | - |
| *VR-12 domain scores* |  |  |  |  |  |  |  |  |
| PF     Mean | 77.420 | 71.435 | - | - | - | - | 60.509 | - |
| Standard deviation | 15.757 | 13.546 | - | - | - | - | 18.770 | - |
| Median | 93.485 | 58.290 | - | - | - | - | 71.400 | - |
| 25th percentile | 58.290 | 58.290 | - | - | - | - | 29.460 | - |
| 75th percentile | 93.485 | 93.485 | - | - | - | - | 80.375 | - |
| Minimum | 29.460 | 58.290 | - | - | - | - | 29.460 | - |
| Maximum | 93.485 | 93.485 | - | - | - | - | 93.485 | - |
| RP     Mean | 74.651 | 43.860 | - | - | - | - | 90.768 | - |
| Standard deviation | 29.800 | 45.608 | - | - | - | - | 9.042 | - |
| Median | 100.690 | -3.645 | - | - | - | - | 89.670 | - |
| 25th percentile | 45.835 | -3.645 | - | - | - | - | 79.405 | - |
| 75th percentile | 100.690 | 100.690 | - | - | - | - | 100.690 | - |
| Minimum | -3.645 | -3.645 | - | - | - | - | 44.720 | - |
| Maximum | 100.690 | 100.690 | - | - | - | - | 100.690 | - |
| BP     Mean | 68.725 | 48.698 | - | - | - | - | 69.535 | - |
| Standard deviation | 16.677 | 10.082 | - | - | - | - | 13.570 | - |
| Median | 56.250 | 43.890 | - | - | - | - | 56.250 | - |
| 25th percentile | 56.250 | 43.890 | - | - | - | - | 56.250 | - |
| 75th percentile | 95.200 | 43.890 | - | - | - | - | 95.200 | - |
| Minimum | 43.890 | 43.890 | - | - | - | - | 56.250 | - |
| Maximum | 95.200 | 95.200 | - | - | - | - | 95.200 | - |
| GH    Mean | 63.304 | 52.869 | - | - | - | - | 62.001 | - |
| Standard deviation | 14.070 | 16.473 | - | - | - | - | 14.579 | - |
| Median | 61.500 | 37.500 | - | - | - | - | 61.500 | - |
| 25th percentile | 37.500 | 37.500 | - | - | - | - | 37.500 | - |
| 75th percentile | 83.710 | 61.500 | - | - | - | - | 83.710 | - |
| Minimum | 37.500 | 37.500 | - | - | - | - | 37.500 | - |
| Maximum | 83.710 | 83.710 | - | - | - | - | 83.710 | - |
| VT     Mean | 48.324 | 34.438 | - | - | - | - | 56.488 | - |
| Standard deviation | 14.590 | 8.909 | - | - | - | - | 15.730 | - |
| Median | 45.900 | 31.930 | - | - | - | - | 54.550 | - |
| 25th percentile | 31.930 | 31.930 | - | - | - | - | 45.900 | - |
| 75th percentile | 54.550 | 45.900 | - | - | - | - | 79.250 | - |
| Minimum | 1.800 | 1.800 | - | - | - | - | 31.930 | - |
| Maximum | 95.340 | 54.550 | - | - | - | - | 95.340 | - |
| SF      Mean | 72.285 | 47.587 | - | - | - | - | 97.500 | - |
| Standard deviation | 21.621 | 13.167 | - | - | - | - | 6.915 | - |
| Median | 66.870 | 48.870 | - | - | - | - | 99.320 | - |
| 25th percentile | 48.870 | 48.870 | - | - | - | - | 99.320 | - |
| 75th percentile | 99.320 | 66.870 | - | - | - | - | 99.320 | - |
| Minimum | 24.910 | 24.910 | - | - | - | - | 48.870 | - |
| Maximum | 99.320 | 66.870 | - | - | - | - | 99.320 | - |
| MH   Mean | 69.214 | 38.125 | - | - | - | - | 87.913 | - |
| Standard deviation | 16.997 | 12.467 | - | - | - | - | 5.688 | - |
| Median | 74.865 | 26.385 | - | - | - | - | 83.845 | - |
| 25th percentile | 60.685 | 26.385 | - | - | - | - | 83.670 | - |
| 75th percentile | 83.845 | 41.685 | - | - | - | - | 92.650 | - |
| Minimum | 21.060 | 26.385 | - | - | - | - | 60.685 | - |
| Maximum | 98.595 | 60.685 | - | - | - | - | 98.595 | - |
| RE     Mean | 76.822 | 30.065 | - | - | - | - | 101.911 | - |
| Standard deviation | 27.356 | 17.991 | - | - | - | - | 8.409 | - |
| Median | 104.125 | 14.165 | - | - | - | - | 104.125 | - |
| 25th percentile | 54.690 | 14.165 | - | - | - | - | 104.125 | - |
| 75th percentile | 104.125 | 41.250 | - | - | - | - | 104.125 | - |
| Minimum | -13.835 | 2.250 | - | - | - | - | 42.775 | - |
| Maximum | 104.125 | 77.005 | - | - | - | - | 104.125 | - |

BP, bodily pain; GH, general health; MCS, mental component summary; MH, mental health; PCS, physical component summary; PF, physical functioning; RE, role emotional; RP, role physical; VT, vitality; SF, social functioning

**SM3 Table 30**. Canadian norms for VR-12 health utility values, summary component scores, and domain scores, by age group – Prince Edward Island, females only.

|  | All participants (n=18) | 18 to 29 (n=4) | 30 to 39 (n=2) | 40 to 49 (n=2) | 50 to 59 (n=5) | 60 to 69 (n=4) | 70 to 79 (n=1) | 80 and over (n=) |
| --- | --- | --- | --- | --- | --- | --- | --- | --- |
| *VR-12 health utility values* |  |  |  |  |  |  |  |  |
| Mean | 0.707 | - | - | - | 0.382 | - | - | - |
| Standard deviation | 0.274 | - | - | - | 0.310 | - | - | - |
| Median | 0.839 | - | - | - | 0.269 | - | - | - |
| 25th percentile | 0.696 | - | - | - | 0.269 | - | - | - |
| 75th percentile | 0.852 | - | - | - | 0.827 | - | - | - |
| Minimum | -0.590 | - | - | - | -0.590 | - | - | - |
| Maximum | 0.852 | - | - | - | 0.827 | - | - | - |
| *VR-12 summary component score* |  |  |  |  |  |  |  |  |
| PCS   Mean | 53.454 | - | - | - | 39.665 | - | - | - |
| Standard deviation | 10.277 | - | - | - | 7.230 | - | - | - |
| Median | 57.908 | - | - | - | 44.091 | - | - | - |
| 25th percentile | 46.721 | - | - | - | 26.017 | - | - | - |
| 75th percentile | 57.908 | - | - | - | 46.663 | - | - | - |
| Minimum | 21.716 | - | - | - | 21.716 | - | - | - |
| Maximum | 66.263 | - | - | - | 46.663 | - | - | - |
| MCS  Mean | 50.283 | - | - | - | 43.876 | - | - | - |
| Standard deviation | 12.492 | - | - | - | 11.970 | - | - | - |
| Median | 56.980 | - | - | - | 31.391 | - | - | - |
| 25th percentile | 45.457 | - | - | - | 31.391 | - | - | - |
| 75th percentile | 56.980 | - | - | - | 62.388 | - | - | - |
| Minimum | 20.625 | - | - | - | 20.625 | - | - | - |
| Maximum | 62.388 | - | - | - | 62.388 | - | - | - |
| *VR-12 domain scores* |  |  |  |  |  |  |  |  |
| PF     Mean | 84.800 | - | - | - | 67.955 | - | - | - |
| Standard deviation | 20.013 | - | - | - | 17.075 | - | - | - |
| Median | 93.485 | - | - | - | 71.400 | - | - | - |
| 25th percentile | 80.375 | - | - | - | 58.290 | - | - | - |
| 75th percentile | 93.485 | - | - | - | 80.375 | - | - | - |
| Minimum | 7.765 | - | - | - | 7.765 | - | - | - |
| Maximum | 93.485 | - | - | - | 93.485 | - | - | - |
| RP     Mean | 85.064 | - | - | - | 50.787 | - | - | - |
| Standard deviation | 33.852 | - | - | - | 27.960 | - | - | - |
| Median | 100.690 | - | - | - | 44.460 | - | - | - |
| 25th percentile | 100.690 | - | - | - | 9.775 | - | - | - |
| 75th percentile | 100.690 | - | - | - | 100.690 | - | - | - |
| Minimum | -3.670 | - | - | - | -3.670 | - | - | - |
| Maximum | 100.690 | - | - | - | 100.690 | - | - | - |
| BP     Mean | 77.652 | - | - | - | 30.981 | - | - | - |
| Standard deviation | 28.999 | - | - | - | 15.479 | - | - | - |
| Median | 95.200 | - | - | - | 31.500 | - | - | - |
| 25th percentile | 56.250 | - | - | - | 2.280 | - | - | - |
| 75th percentile | 95.200 | - | - | - | 56.250 | - | - | - |
| Minimum | 2.280 | - | - | - | 2.280 | - | - | - |
| Maximum | 95.200 | - | - | - | 56.250 | - | - | - |
| GH    Mean | 75.469 | - | - | - | 64.575 | - | - | - |
| Standard deviation | 22.003 | - | - | - | 18.431 | - | - | - |
| Median | 83.710 | - | - | - | 61.500 | - | - | - |
| 25th percentile | 61.500 | - | - | - | 61.500 | - | - | - |
| 75th percentile | 83.710 | - | - | - | 83.710 | - | - | - |
| Minimum | 0.580 | - | - | - | 0.580 | - | - | - |
| Maximum | 101.840 | - | - | - | 83.710 | - | - | - |
| VT     Mean | 60.309 | - | - | - | 27.728 | - | - | - |
| Standard deviation | 28.513 | - | - | - | 21.302 | - | - | - |
| Median | 79.250 | - | - | - | 1.800 | - | - | - |
| 25th percentile | 54.550 | - | - | - | 1.800 | - | - | - |
| 75th percentile | 79.250 | - | - | - | 54.550 | - | - | - |
| Minimum | 1.800 | - | - | - | 1.800 | - | - | - |
| Maximum | 79.250 | - | - | - | 79.250 | - | - | - |
| SF      Mean | 77.345 | - | - | - | 44.777 | - | - | - |
| Standard deviation | 35.318 | - | - | - | 27.066 | - | - | - |
| Median | 99.320 | - | - | - | 24.910 | - | - | - |
| 25th percentile | 66.870 | - | - | - | 24.910 | - | - | - |
| 75th percentile | 99.320 | - | - | - | 99.320 | - | - | - |
| Minimum | 2.780 | - | - | - | 2.780 | - | - | - |
| Maximum | 99.320 | - | - | - | 99.320 | - | - | - |
| MH   Mean | 71.629 | - | - | - | 57.334 | - | - | - |
| Standard deviation | 24.192 | - | - | - | 26.796 | - | - | - |
| Median | 83.670 | - | - | - | 31.800 | - | - | - |
| 25th percentile | 74.865 | - | - | - | 31.800 | - | - | - |
| 75th percentile | 83.670 | - | - | - | 98.595 | - | - | - |
| Minimum | -1.030 | - | - | - | -1.030 | - | - | - |
| Maximum | 98.595 | - | - | - | 98.595 | - | - | - |
| RE     Mean | 83.331 | - | - | - | 65.957 | - | - | - |
| Standard deviation | 38.134 | - | - | - | 32.056 | - | - | - |
| Median | 104.125 | - | - | - | 41.250 | - | - | - |
| 25th percentile | 41.250 | - | - | - | 41.250 | - | - | - |
| 75th percentile | 104.125 | - | - | - | 104.125 | - | - | - |
| Minimum | -20.070 | - | - | - | -20.070 | - | - | - |
| Maximum | 104.125 | - | - | - | 104.125 | - | - | - |

BP, bodily pain; GH, general health; MCS, mental component summary; MH, mental health; PCS, physical component summary; PF, physical functioning; RE, role emotional; RP, role physical; VT, vitality; SF, social functioning

**SM3 Table 31**. Canadian norms for VR-12 health utility values, summary component scores, and domain scores, by age group – Northwest Territories.

|  | All participants (n=10) | 18 to 29 | 30 to 39 | 40 to 49 | 50 to 59 | 60 to 69 | 70 to 79 | 80 and over |
| --- | --- | --- | --- | --- | --- | --- | --- | --- |
| *VR-12 health utility values* |  |  |  |  |  |  |  |  |
| Mean | 0.654 | - | - | - | - | - | - | - |
| Standard deviation | 0.203 | - | - | - | - | - | - | - |
| Median | 0.696 | - | - | - | - | - | - | - |
| 25th percentile | 0.615 | - | - | - | - | - | - | - |
| 75th percentile | 0.818 | - | - | - | - | - | - | - |
| Minimum | 0.111 | - | - | - | - | - | - | - |
| Maximum | 0.900 | - | - | - | - | - | - | - |
| *VR-12 summary component score* |  |  |  |  |  |  |  |  |
| PCS   Mean | 50.428 | - | - | - | - | - | - | - |
| Standard deviation | 8.551 | - | - | - | - | - | - | - |
| Median | 55.196 | - | - | - | - | - | - | - |
| 25th percentile | 40.409 | - | - | - | - | - | - | - |
| 75th percentile | 58.491 | - | - | - | - | - | - | - |
| Minimum | 33.480 | - | - | - | - | - | - | - |
| Maximum | 60.792 | - | - | - | - | - | - | - |
| MCS  Mean | 47.429 | - | - | - | - | - | - | - |
| Standard deviation | 8.490 | - | - | - | - | - | - | - |
| Median | 51.401 | - | - | - | - | - | - | - |
| 25th percentile | 38.032 | - | - | - | - | - | - | - |
| 75th percentile | 55.572 | - | - | - | - | - | - | - |
| Minimum | 29.808 | - | - | - | - | - | - | - |
| Maximum | 57.355 | - | - | - | - | - | - | - |
| *VR-12 domain scores* |  |  |  |  |  |  |  |  |
| PF     Mean | 75.133 | - | - | - | - | - | - | - |
| Standard deviation | 21.114 | - | - | - | - | - | - | - |
| Median | 93.485 | - | - | - | - | - | - | - |
| 25th percentile | 58.290 | - | - | - | - | - | - | - |
| 75th percentile | 93.485 | - | - | - | - | - | - | - |
| Minimum | 29.460 | - | - | - | - | - | - | - |
| Maximum | 93.485 | - | - | - | - | - | - | - |
| RP     Mean | 69.775 | - | - | - | - | - | - | - |
| Standard deviation | 33.646 | - | - | - | - | - | - | - |
| Median | 100.690 | - | - | - | - | - | - | - |
| 25th percentile | 9.775 | - | - | - | - | - | - | - |
| 75th percentile | 100.690 | - | - | - | - | - | - | - |
| Minimum | 9.775 | - | - | - | - | - | - | - |
| Maximum | 100.690 | - | - | - | - | - | - | - |
| BP     Mean | 69.601 | - | - | - | - | - | - | - |
| Standard deviation | 21.628 | - | - | - | - | - | - | - |
| Median | 56.250 | - | - | - | - | - | - | - |
| 25th percentile | 43.890 | - | - | - | - | - | - | - |
| 75th percentile | 95.200 | - | - | - | - | - | - | - |
| Minimum | 31.500 | - | - | - | - | - | - | - |
| Maximum | 95.200 | - | - | - | - | - | - | - |
| GH    Mean | 75.583 | - | - | - | - | - | - | - |
| Standard deviation | 17.976 | - | - | - | - | - | - | - |
| Median | 61.500 | - | - | - | - | - | - | - |
| 25th percentile | 61.500 | - | - | - | - | - | - | - |
| 75th percentile | 101.840 | - | - | - | - | - | - | - |
| Minimum | 37.500 | - | - | - | - | - | - | - |
| Maximum | 101.840 | - | - | - | - | - | - | - |
| VT     Mean | 52.727 | - | - | - | - | - | - | - |
| Standard deviation | 19.317 | - | - | - | - | - | - | - |
| Median | 45.900 | - | - | - | - | - | - | - |
| 25th percentile | 31.930 | - | - | - | - | - | - | - |
| 75th percentile | 54.550 | - | - | - | - | - | - | - |
| Minimum | 31.930 | - | - | - | - | - | - | - |
| Maximum | 95.340 | - | - | - | - | - | - | - |
| SF      Mean | 72.541 | - | - | - | - | - | - | - |
| Standard deviation | 20.849 | - | - | - | - | - | - | - |
| Median | 66.870 | - | - | - | - | - | - | - |
| 25th percentile | 48.870 | - | - | - | - | - | - | - |
| 75th percentile | 99.320 | - | - | - | - | - | - | - |
| Minimum | 24.910 | - | - | - | - | - | - | - |
| Maximum | 99.320 | - | - | - | - | - | - | - |
| MH   Mean | 57.856 | - | - | - | - | - | - | - |
| Standard deviation | 21.063 | - | - | - | - | - | - | - |
| Median | 65.505 | - | - | - | - | - | - | - |
| 25th percentile | 41.440 | - | - | - | - | - | - | - |
| 75th percentile | 74.865 | - | - | - | - | - | - | - |
| Minimum | 6.005 | - | - | - | - | - | - | - |
| Maximum | 83.670 | - | - | - | - | - | - | - |
| RE     Mean | 78.296 | - | - | - | - | - | - | - |
| Standard deviation | 23.850 | - | - | - | - | - | - | - |
| Median | 81.810 | - | - | - | - | - | - | - |
| 25th percentile | 54.690 | - | - | - | - | - | - | - |
| 75th percentile | 104.125 | - | - | - | - | - | - | - |
| Minimum | 29.335 | - | - | - | - | - | - | - |
| Maximum | 104.125 | - | - | - | - | - | - | - |

BP, bodily pain; GH, general health; MCS, mental component summary; MH, mental health; PCS, physical component summary; PF, physical functioning; RE, role emotional; RP, role physical; VT, vitality; SF, social functioning

**SM3 Table 32**. Canadian norms for VR-12 health utility values, summary component scores, and domain scores, by age group – Northwest Territories, males only.

|  | All participants  (n=6) | 18 to 29 | 30 to 39 | 40 to 49 | 50 to 59 | 60 to 69 | 70 to 79 | 80 and over |
| --- | --- | --- | --- | --- | --- | --- | --- | --- |
| *VR-12 health utility values* |  |  |  |  |  |  |  |  |
| Mean | 0.640 | - | - | - | - | - | - | - |
| Standard deviation | 0.245 | - | - | - | - | - | - | - |
| Median | 0.644 | - | - | - | - | - | - | - |
| 25th percentile | 0.615 | - | - | - | - | - | - | - |
| 75th percentile | 0.900 | - | - | - | - | - | - | - |
| Minimum | 0.111 | - | - | - | - | - | - | - |
| Maximum | 0.900 | - | - | - | - | - | - | - |
| *VR-12 summary component score* |  |  |  |  |  |  |  |  |
| PCS   Mean | 48.727 | - | - | - | - | - | - | - |
| Standard deviation | 10.501 | - | - | - | - | - | - | - |
| Median | 47.251 | - | - | - | - | - | - | - |
| 25th percentile | 40.409 | - | - | - | - | - | - | - |
| 75th percentile | 60.792 | - | - | - | - | - | - | - |
| Minimum | 33.480 | - | - | - | - | - | - | - |
| Maximum | 60.792 | - | - | - | - | - | - | - |
| MCS  Mean | 46.848 | - | - | - | - | - | - | - |
| Standard deviation | 9.538 | - | - | - | - | - | - | - |
| Median | 51.401 | - | - | - | - | - | - | - |
| 25th percentile | 38.032 | - | - | - | - | - | - | - |
| 75th percentile | 55.572 | - | - | - | - | - | - | - |
| Minimum | 30.797 | - | - | - | - | - | - | - |
| Maximum | 57.355 | - | - | - | - | - | - | - |
| *VR-12 domain scores* |  |  |  |  |  |  |  |  |
| PF     Mean | 69.352 | - | - | - | - | - | - | - |
| Standard deviation | 25.481 | - | - | - | - | - | - | - |
| Median | 58.290 | - | - | - | - | - | - | - |
| 25th percentile | 58.290 | - | - | - | - | - | - | - |
| 75th percentile | 93.485 | - | - | - | - | - | - | - |
| Minimum | 29.460 | - | - | - | - | - | - | - |
| Maximum | 93.485 | - | - | - | - | - | - | - |
| RP     Mean | 61.453 | - | - | - | - | - | - | - |
| Standard deviation | 41.135 | - | - | - | - | - | - | - |
| Median | 89.670 | - | - | - | - | - | - | - |
| 25th percentile | 9.775 | - | - | - | - | - | - | - |
| 75th percentile | 100.690 | - | - | - | - | - | - | - |
| Minimum | 9.775 | - | - | - | - | - | - | - |
| Maximum | 100.690 | - | - | - | - | - | - | - |
| BP     Mean | 64.422 | - | - | - | - | - | - | - |
| Standard deviation | 24.676 | - | - | - | - | - | - | - |
| Median | 56.250 | - | - | - | - | - | - | - |
| 25th percentile | 43.890 | - | - | - | - | - | - | - |
| 75th percentile | 95.200 | - | - | - | - | - | - | - |
| Minimum | 31.500 | - | - | - | - | - | - | - |
| Maximum | 95.200 | - | - | - | - | - | - | - |
| GH    Mean | 75.884 | - | - | - | - | - | - | - |
| Standard deviation | 17.026 | - | - | - | - | - | - | - |
| Median | 61.500 | - | - | - | - | - | - | - |
| 25th percentile | 61.500 | - | - | - | - | - | - | - |
| 75th percentile | 101.840 | - | - | - | - | - | - | - |
| Minimum | 61.500 | - | - | - | - | - | - | - |
| Maximum | 101.840 | - | - | - | - | - | - | - |
| VT     Mean | 54.491 | - | - | - | - | - | - | - |
| Standard deviation | 24.962 | - | - | - | - | - | - | - |
| Median | 45.900 | - | - | - | - | - | - | - |
| 25th percentile | 31.930 | - | - | - | - | - | - | - |
| 75th percentile | 95.340 | - | - | - | - | - | - | - |
| Minimum | 31.930 | - | - | - | - | - | - | - |
| Maximum | 95.340 | - | - | - | - | - | - | - |
| SF      Mean | 72.179 | - | - | - | - | - | - | - |
| Standard deviation | 21.782 | - | - | - | - | - | - | - |
| Median | 66.870 | - | - | - | - | - | - | - |
| 25th percentile | 48.870 | - | - | - | - | - | - | - |
| 75th percentile | 99.320 | - | - | - | - | - | - | - |
| Minimum | 48.870 | - | - | - | - | - | - | - |
| Maximum | 99.320 | - | - | - | - | - | - | - |
| MH   Mean | 53.960 | - | - | - | - | - | - | - |
| Standard deviation | 22.520 | - | - | - | - | - | - | - |
| Median | 65.505 | - | - | - | - | - | - | - |
| 25th percentile | 41.440 | - | - | - | - | - | - | - |
| 75th percentile | 69.490 | - | - | - | - | - | - | - |
| Minimum | 6.005 | - | - | - | - | - | - | - |
| Maximum | 74.865 | - | - | - | - | - | - | - |
| RE     Mean | 73.205 | - | - | - | - | - | - | - |
| Standard deviation | 27.974 | - | - | - | - | - | - | - |
| Median | 81.810 | - | - | - | - | - | - | - |
| 25th percentile | 54.690 | - | - | - | - | - | - | - |
| 75th percentile | 104.125 | - | - | - | - | - | - | - |
| Minimum | 29.335 | - | - | - | - | - | - | - |
| Maximum | 104.125 | - | - | - | - | - | - | - |

BP, bodily pain; GH, general health; MCS, mental component summary; MH, mental health; PCS, physical component summary; PF, physical functioning; RE, role emotional; RP, role physical; VT, vitality; SF, social functioning

**SM4 Table 1**. Canadian norms for VR-12 health utility values and summary component scores, across seven self-reported health conditions (anemia or blood disease, back pain, cancer, depression, diabetes, heart disease, high blood pressure). Values are means (standard deviations).

|  | Anemia or blood disease (n=294) | Back pain (n=1929) | Cancer  (n=174) | Depression (n=1265) | Diabetes  (n=739) | Heart disease (n=237) | High blood pressure (n=1440) |
| --- | --- | --- | --- | --- | --- | --- | --- |
| VR-12 health utility values | 0.579 (0.242) | 0.607 (0.250) | 0.619 (0.278) | 0.493 (0.252) | 0.652 (0.237) | 0.634 (0.238) | 0.682 (0.212) |
| VR-12 summary component score |  |  |  |  |  |  |  |
| Physical component summary | 46.594 (9.365) | 44.487 (10.832) | 42.060 (10.485) | 47.776 (10.167) | 42.946 (10.848) | 39.912 (11.813) | 44.572 (10.330) |
| Mental component summary | 45.499 (10.810) | 48.664 (10.215) | 50.604 (10.187) | 38.867 (9.699) | 51.770 (8.962) | 52.158 (9.177) | 52.174 (8.592) |
| VR-12 domain scores |  |  |  |  |  |  |  |
| Physical functioning | 69.259 (23.559) | 65.651 (26.779) | 59.575 (28.139) | 67.913 (24.805) | 61.821 (27.690) | 55.632 (27.983) | 66.347 (26.384) |
| Role physical | 63.281 (33.559) | 59.736 (38.541) | 53.880 (42.818) | 58.894 (35.619) | 59.864 (38.686) | 52.348 (41.876) | 65.919 (36.538) |
| Bodily pain | 58.446 (23.769) | 54.891 (22.566) | 57.772 (30.579) | 59.035 (23.785) | 61.583 (25.268) | 59.852 (27.823) | 63.098 (24.415) |
| General health | 64.869 (19.564) | 67.618 (22.344) | 60.327 (27.960) | 63.453 (21.957) | 60.397 (23.178) | 54.216 (25.249) | 63.339 (21.492) |
| Vitality | 47.501 (20.130) | 49.585 (21.471) | 50.286 (24.632) | 42.069 (21.423) | 51.058 (22.423) | 46.361 (26.452) | 51.190 (20.770) |
| Social functioning | 63.704 (28.880) | 68.549 (28.121) | 73.008 (29.213) | 48.577 (25.113) | 74.042 (26.372) | 72.722 (28.461) | 76.446 (25.493) |
| Mental health | 61.385 (22.291) | 66.384 (22.191) | 71.139 (22.630) | 48.666 (21.653) | 72.610 (20.732) | 74.800 (19.824) | 74.142 (19.380) |
| Role emotional | 57.638 (38.704) | 66.307 (38.163) | 65.358 (42.845) | 39.736 (35.370) | 74.033 (35.822) | 70.153 (36.538) | 77.054 (32.878) |

**SM4 Table 2.** Canadian norms for VR-12 health utility values and summary component scores, across six self-reported health conditions (kidney disease, liver disease, lung disease, osteoarthritis, rheumatoid arthritis, ulcer or stomach disease). Values are means (standard deviations).

|  | Kidney disease (n=71) | Liver disease (n=64) | Lung disease (n=146) | Osteoarthritis (n=723) | Rheumatoid arthritis (n=281) | Ulcer or stomach disease (n=187) |
| --- | --- | --- | --- | --- | --- | --- |
| VR-12 health utility values | 0.584 (0.251) | 0.607 (0.189) | 0.576 (0.233) | 0.596 (0.245) | 0.554 (0.237) | 0.579 (0.221) |
| VR-12 summary component score |  |  |  |  |  |  |
| Physical component summary | 38.956 (9.595) | 45.600 (9.634) | 36.684 (10.582) | 39.502 (10.573) | 40.440 (10.833) | 43.552 (9.679) |
| Mental component summary | 49.914 (8.866) | 46.759 (9.708) | 50.257 (8.526) | 50.616 (9.298) | 48.661 (8.795) | 46.218 (8.545) |
| VR-12 domain scores |  |  |  |  |  |  |
| Physical functioning | 52.693 (24.264) | 64.339 (22.307) | 44.541 (27.966) | 54.989 (26.731) | 54.779 (27.128) | 66.531 (22.424) |
| Role physical | 49.922 (34.363) | 62.506 (32.938) | 37.677 (39.793) | 48.303 (38.255) | 52.198 (37.711) | 52.726 (34.437) |
| Bodily pain | 56.474 (22.528) | 64.814 (21.857) | 55.288 (27.469) | 50.284 (22.167) | 50.067 (21.403) | 53.934 (19.737) |
| General health | 53.376 (19.525) | 63.993 (19.593) | 53.225 (20.318) | 61.682 (22.662) | 60.382 (22.106) | 59.389 (22.960) |
| Vitality | 40.098 (19.748) | 49.535 (20.190) | 41.532 (26.835) | 44.459 (20.676) | 48.575 (20.634) | 44.829 (18.864) |
| Social functioning | 66.157 (26.347) | 60.349 (23.614) | 64.625 (25.310) | 70.782 (27.021) | 63.007 (25.297) | 61.066 (24.762) |
| Mental health | 70.803 (19.148) | 64.875 (20.798) | 69.740 (19.827) | 69.186 (20.976) | 66.308 (20.181) | 62.808 (19.512) |
| Role emotional | 64.932 (34.667) | 56.341 (30.907) | 63.206 (36.667) | 68.227 (36.900) | 61.078 (36.413) | 58.260 (31.481) |

**SM4 Table 3**. Canadian norms for VR-12 health utility values, summary component scores, and domain scores, across seven self-reported health conditions (anemia or blood disease, back pain, cancer, depression, diabetes, heart disease, and high blood pressure), by gender.

|  | Anemia or blood disease | | Back pain | | Cancer | | Depression | | Diabetes | | Heart disease | | High blood pressure | |
| --- | --- | --- | --- | --- | --- | --- | --- | --- | --- | --- | --- | --- | --- | --- |
|  | Male (n=56) | Female (n=238) | Male (n=912) | Female (n=1017) | Male (n=92) | Female (n=82) | Male (n=518) | Female (n=747) | Male (n=458) | Female (n=281) | Male (n=160) | Female (n=77) | Male (n=834) | Female (n=606) |
| *VR-12 health utility values* |  |  |  |  |  |  |  |  |  |  |  |  |  |  |
| Mean | 0.637 | 0.564 | 0.658 | 0.564 | 0.677 | 0.557 | 0.528 | 0.477 | 0.684 | 0.601 | 0.667 | 0.545 | 0.708 | 0.648 |
| Standard deviation | 0.231 | 0.242 | 0.205 | 0.277 | 0.210 | 0.327 | 0.230 | 0.263 | 0.218 | 0.257 | 0.235 | 0.228 | 0.191 | 0.235 |
| Median | 0.704 | 0.644 | 0.705 | 0.664 | 0.704 | 0.644 | 0.606 | 0.553 | 0.737 | 0.664 | 0.713 | 0.547 | 0.758 | 0.706 |
| 25th percentile | 0.606 | 0.374 | 0.570 | 0.427 | 0.570 | 0.456 | 0.388 | 0.311 | 0.610 | 0.456 | 0.548 | 0.438 | 0.644 | 0.547 |
| 75th percentile | 0.789 | 0.765 | 0.802 | 0.768 | 0.839 | 0.777 | 0.716 | 0.696 | 0.829 | 0.778 | 0.830 | 0.741 | 0.829 | 0.818 |
| Minimum | -0.489 | -0.390 | -0.590 | -0.590 | -0.263 | -0.249 | -0.590 | -0.590 | -0.590 | -0.393 | -0.211 | -0.174 | -0.590 | -0.590 |
| Maximum | 0.903 | 1.000 | 0.960 | 1.000 | 1.000 | 1.000 | 0.952 | 1.000 | 1.000 | 1.000 | 0.952 | 0.913 | 1.000 | 1.000 |
| *VR-12 summary component score* |  |  |  |  |  |  |  |  |  |  |  |  |  |  |
| PCS    Mean | 43.882 | 47.331 | 45.363 | 43.693 | 41.874 | 42.258 | 49.325 | 46.868 | 43.753 | 41.632 | 41.135 | 36.618 | 45.274 | 43.672 |
| Standard deviation | 9.112 | 9.349 | 10.375 | 11.135 | 10.528 | 10.497 | 9.700 | 10.343 | 10.564 | 11.201 | 11.602 | 11.793 | 9.820 | 10.949 |
| Median | 41.863 | 48.400 | 47.500 | 44.881 | 40.686 | 39.253 | 52.403 | 47.855 | 44.553 | 42.459 | 41.784 | 37.515 | 46.097 | 43.998 |
| 25th percentile | 39.896 | 40.641 | 38.457 | 36.585 | 35.991 | 34.796 | 41.869 | 39.361 | 37.209 | 33.579 | 32.956 | 28.200 | 38.074 | 36.205 |
| 75th percentile | 48.891 | 54.838 | 54.277 | 53.263 | 50.700 | 52.218 | 57.761 | 55.653 | 53.542 | 52.385 | 50.011 | 45.019 | 54.838 | 53.978 |
| Minimum | 19.864 | 10.489 | 10.492 | 5.715 | 21.238 | 14.648 | 18.462 | 12.998 | 10.494 | 5.715 | 10.844 | 4.409 | 10.492 | 4.409 |
| Maximum | 60.230 | 64.776 | 63.573 | 66.246 | 60.155 | 61.522 | 70.847 | 70.390 | 64.732 | 61.412 | 62.450 | 62.714 | 65.528 | 65.783 |
| MCS   Mean | 51.250 | 44.021 | 50.717 | 46.969 | 52.924 | 48.121 | 39.687 | 38.615 | 52.880 | 49.961 | 53.040 | 49.781 | 53.074 | 51.079 |
| Standard deviation | 10.320 | 10.438 | 9.042 | 10.758 | 8.639 | 11.140 | 9.213 | 9.861 | 8.570 | 9.326 | 9.390 | 8.393 | 8.005 | 9.194 |
| Median | 58.059 | 45.563 | 52.307 | 49.552 | 54.047 | 47.463 | 39.746 | 39.390 | 55.937 | 52.491 | 55.110 | 51.709 | 55.218 | 53.160 |
| 25th percentile | 42.009 | 35.642 | 44.966 | 39.790 | 48.222 | 39.625 | 32.551 | 30.489 | 47.648 | 43.836 | 46.134 | 42.236 | 49.194 | 44.780 |
| 75th percentile | 60.091 | 53.325 | 58.062 | 55.308 | 60.287 | 56.297 | 47.365 | 46.284 | 59.588 | 57.047 | 61.141 | 56.652 | 59.516 | 58.396 |
| Minimum | 18.311 | 17.620 | 15.291 | 12.946 | 22.375 | 14.330 | 10.612 | 8.848 | 16.950 | 18.523 | 25.443 | 24.854 | 18.311 | 18.832 |
| Maximum | 67.596 | 68.956 | 70.517 | 67.974 | 65.494 | 65.941 | 64.013 | 65.767 | 67.596 | 65.941 | 64.985 | 63.154 | 70.087 | 67.974 |
| *VR-12 domain scores* |  |  |  |  |  |  |  |  |  |  |  |  |  |  |
| PF     Mean | 68.260 | 69.480 | 69.336 | 62.411 | 59.862 | 59.267 | 72.404 | 65.229 | 63.640 | 58.856 | 59.630 | 44.862 | 69.429 | 62.350 |
| Standard deviation | 25.123 | 23.266 | 24.997 | 27.924 | 29.577 | 26.611 | 23.759 | 25.214 | 26.680 | 29.088 | 27.828 | 26.013 | 24.077 | 28.841 |
| Median | 71.400 | 80.375 | 71.400 | 58.290 | 58.290 | 58.290 | 93.485 | 71.400 | 58.290 | 58.290 | 58.290 | 51.545 | 80.375 | 58.290 |
| 25th percentile | 58.290 | 58.290 | 58.290 | 36.595 | 29.460 | 36.595 | 58.290 | 58.290 | 58.290 | 29.460 | 36.595 | 29.460 | 58.290 | 36.595 |
| 75th percentile | 93.485 | 93.485 | 93.485 | 93.485 | 80.375 | 80.375 | 93.485 | 93.485 | 93.485 | 93.485 | 80.375 | 58.290 | 93.485 | 93.485 |
| Minimum | 7.765 | 7.765 | 7.765 | 7.765 | 7.765 | 7.765 | 7.765 | 7.765 | 7.765 | 7.765 | 7.765 | 7.765 | 7.765 | 7.765 |
| Maximum | 93.485 | 93.485 | 93.485 | 93.485 | 93.485 | 93.485 | 93.485 | 93.485 | 93.485 | 93.485 | 93.485 | 93.485 | 93.485 | 93.485 |
| RP     Mean | 57.090 | 65.271 | 64.841 | 55.290 | 59.299 | 48.083 | 63.168 | 56.960 | 62.311 | 55.876 | 55.872 | 42.851 | 68.760 | 62.486 |
| Standard deviation | 32.121 | 33.647 | 36.784 | 39.546 | 41.011 | 44.177 | 33.902 | 36.290 | 38.201 | 39.234 | 42.654 | 39.170 | 34.453 | 38.886 |
| Median | 51.185 | 79.405 | 79.405 | 79.405 | 79.405 | 44.460 | 79.405 | 79.405 | 79.405 | 79.405 | 79.405 | 9.775 | 79.405 | 79.405 |
| 25th percentile | 39.705 | 40.190 | 9.775 | 9.775 | 9.775 | 9.775 | 9.775 | 9.775 | 9.775 | 5.245 | 9.775 | 5.020 | 44.460 | 9.775 |
| 75th percentile | 79.405 | 100.690 | 100.690 | 100.690 | 90.425 | 89.670 | 100.690 | 89.670 | 100.690 | 90.425 | 90.425 | 79.405 | 100.690 | 100.690 |
| Minimum | -3.670 | -3.670 | -3.670 | -3.670 | -3.645 | -3.670 | -3.670 | -3.670 | -3.670 | -3.670 | -3.670 | -3.670 | -3.670 | -3.670 |
| Maximum | 100.690 | 100.690 | 100.690 | 100.690 | 100.690 | 100.690 | 100.690 | 100.690 | 100.690 | 100.690 | 100.690 | 100.690 | 100.690 | 100.690 |
| BP     Mean | 56.324 | 58.853 | 56.922 | 53.134 | 60.522 | 54.829 | 61.972 | 57.373 | 65.135 | 55.794 | 63.756 | 49.333 | 65.176 | 60.508 |
| Standard deviation | 22.189 | 24.083 | 21.145 | 23.656 | 28.606 | 32.536 | 22.830 | 24.295 | 25.213 | 24.408 | 28.177 | 24.786 | 23.563 | 25.333 |
| Median | 56.250 | 56.250 | 56.250 | 56.250 | 56.250 | 56.250 | 56.250 | 56.250 | 56.250 | 56.250 | 56.250 | 56.250 | 56.250 | 56.250 |
| 25th percentile | 56.250 | 43.890 | 43.890 | 43.890 | 43.890 | 31.500 | 43.890 | 43.890 | 43.890 | 43.890 | 43.890 | 31.500 | 43.890 | 43.890 |
| 75th percentile | 56.250 | 95.200 | 56.250 | 56.250 | 95.200 | 95.200 | 95.200 | 95.200 | 95.200 | 56.250 | 95.200 | 56.250 | 95.200 | 95.200 |
| Minimum | 2.280 | 2.280 | 2.280 | 2.280 | 2.280 | 2.280 | 2.280 | 2.280 | 2.280 | 2.280 | 2.280 | 2.280 | 2.280 | 2.280 |
| Maximum | 95.200 | 95.200 | 95.200 | 95.200 | 95.200 | 95.200 | 95.200 | 95.200 | 95.200 | 95.200 | 95.200 | 95.200 | 95.200 | 95.200 |
| GH    Mean | 66.816 | 64.465 | 68.343 | 67.024 | 58.365 | 62.426 | 64.062 | 63.657 | 61.011 | 59.396 | 54.224 | 54.195 | 62.672 | 64.200 |
| Standard deviation | 23.291 | 18.571 | 22.441 | 22.202 | 31.024 | 24.054 | 22.431 | 20.950 | 22.199 | 24.703 | 25.413 | 25.070 | 21.579 | 21.378 |
| Median | 61.500 | 61.500 | 83.710 | 61.500 | 61.500 | 61.500 | 61.500 | 61.500 | 61.500 | 61.500 | 61.500 | 61.500 | 61.500 | 61.500 |
| 25th percentile | 61.500 | 61.500 | 61.500 | 61.500 | 37.500 | 61.500 | 61.500 | 61.500 | 37.500 | 37.500 | 37.500 | 37.500 | 37.500 | 61.500 |
| 75th percentile | 83.710 | 83.710 | 83.710 | 83.710 | 83.710 | 83.710 | 83.710 | 83.710 | 83.710 | 83.710 | 83.710 | 83.710 | 83.710 | 83.710 |
| Minimum | 0.580 | 0.580 | 0.580 | 0.580 | 0.580 | 0.580 | 0.580 | 0.580 | 0.580 | 0.580 | 0.580 | 0.580 | 0.580 | 0.580 |
| Maximum | 101.840 | 101.840 | 101.840 | 101.840 | 101.840 | 83.710 | 101.840 | 101.840 | 101.840 | 101.840 | 101.840 | 101.840 | 101.840 | 101.840 |
| VT     Mean | 53.887 | 45.962 | 53.777 | 45.982 | 51.296 | 49.205 | 47.357 | 39.295 | 53.019 | 47.862 | 47.842 | 42.371 | 53.043 | 48.837 |
| Standard deviation | 23.606 | 18.808 | 20.773 | 21.451 | 25.185 | 24.097 | 21.385 | 20.708 | 21.461 | 23.630 | 27.950 | 22.803 | 19.937 | 21.684 |
| Median | 45.900 | 45.900 | 54.550 | 45.900 | 54.550 | 54.550 | 45.900 | 45.900 | 54.550 | 45.900 | 45.900 | 31.930 | 54.550 | 45.900 |
| 25th percentile | 45.900 | 31.930 | 45.900 | 31.930 | 31.930 | 45.900 | 31.930 | 31.930 | 31.930 | 31.930 | 31.930 | 31.930 | 45.900 | 31.930 |
| 75th percentile | 79.250 | 54.550 | 79.250 | 54.550 | 79.250 | 54.550 | 54.550 | 54.550 | 79.250 | 54.550 | 79.250 | 54.550 | 79.250 | 54.550 |
| Minimum | 1.800 | 1.800 | 1.800 | 1.800 | 1.800 | 1.800 | 1.800 | 1.800 | 1.800 | 1.800 | 1.800 | 1.800 | 1.800 | 1.800 |
| Maximum | 95.340 | 95.340 | 95.340 | 95.340 | 95.340 | 95.340 | 95.340 | 95.340 | 95.340 | 95.340 | 95.340 | 95.340 | 95.340 | 95.340 |
| SF      Mean | 69.423 | 62.423 | 71.492 | 66.192 | 78.474 | 67.160 | 49.000 | 48.605 | 77.447 | 68.493 | 76.782 | 61.782 | 78.381 | 74.069 |
| Standard deviation | 33.535 | 27.387 | 26.071 | 29.478 | 26.047 | 31.378 | 24.144 | 25.683 | 24.906 | 27.843 | 26.876 | 29.416 | 23.912 | 27.336 |
| Median | 66.870 | 66.870 | 66.870 | 66.870 | 99.320 | 66.870 | 48.870 | 48.870 | 99.320 | 66.870 | 99.320 | 66.870 | 99.320 | 66.870 |
| 25th percentile | 48.870 | 48.870 | 48.870 | 48.870 | 66.870 | 48.870 | 24.910 | 24.910 | 66.870 | 48.870 | 48.870 | 48.870 | 66.870 | 48.870 |
| 75th percentile | 99.320 | 99.320 | 99.320 | 99.320 | 99.320 | 99.320 | 66.870 | 66.870 | 99.320 | 99.320 | 99.320 | 99.320 | 99.320 | 99.320 |
| Minimum | 2.780 | 2.780 | 2.780 | 2.780 | 2.780 | 2.780 | 2.780 | 2.780 | 2.780 | 2.780 | 2.780 | 2.780 | 2.780 | 2.780 |
| Maximum | 99.320 | 99.320 | 99.320 | 99.320 | 99.320 | 99.320 | 99.320 | 99.320 | 99.320 | 99.320 | 99.320 | 99.320 | 99.320 | 99.320 |
| MH   Mean | 71.917 | 58.676 | 71.786 | 61.827 | 75.249 | 66.742 | 50.107 | 48.302 | 75.571 | 67.786 | 76.486 | 70.257 | 76.877 | 70.793 |
| Standard deviation | 20.717 | 21.887 | 19.663 | 23.175 | 19.692 | 24.805 | 20.199 | 22.395 | 19.648 | 21.644 | 20.729 | 17.245 | 18.032 | 20.516 |
| Median | 83.670 | 60.685 | 74.865 | 65.505 | 74.865 | 69.490 | 51.325 | 51.045 | 83.670 | 74.865 | 83.670 | 74.485 | 83.670 | 74.865 |
| 25th percentile | 59.850 | 41.685 | 60.685 | 48.020 | 65.505 | 49.110 | 31.800 | 30.945 | 65.505 | 51.325 | 65.505 | 51.325 | 65.505 | 55.620 |
| 75th percentile | 83.670 | 83.670 | 83.845 | 83.670 | 92.650 | 83.670 | 69.490 | 65.505 | 92.650 | 83.670 | 92.650 | 83.845 | 92.650 | 92.650 |
| Minimum | -1.030 | -1.030 | -1.030 | -1.030 | -1.030 | -1.030 | -1.030 | -1.030 | -1.030 | -1.030 | 6.005 | 6.005 | -1.030 | -1.030 |
| Maximum | 92.650 | 98.595 | 98.595 | 98.595 | 98.595 | 98.595 | 98.595 | 98.595 | 98.595 | 98.595 | 98.595 | 98.595 | 98.595 | 98.595 |
| RE     Mean | 74.453 | 53.211 | 71.370 | 62.154 | 72.500 | 57.715 | 44.697 | 37.387 | 76.024 | 70.789 | 73.099 | 62.218 | 79.493 | 74.063 |
| Standard deviation | 32.721 | 39.010 | 34.804 | 40.253 | 38.445 | 46.159 | 33.452 | 36.132 | 35.117 | 36.795 | 35.701 | 37.494 | 31.124 | 34.920 |
| Median | 77.005 | 54.690 | 81.810 | 68.370 | 81.810 | 68.370 | 41.285 | 41.250 | 104.125 | 81.810 | 77.005 | 68.370 | 104.125 | 81.810 |
| 25th percentile | 54.690 | 14.165 | 42.775 | 29.335 | 41.250 | 29.335 | 14.165 | 7.930 | 54.690 | 41.285 | 41.285 | 35.050 | 54.690 | 54.690 |
| 75th percentile | 104.125 | 104.125 | 104.125 | 104.125 | 104.125 | 104.125 | 77.005 | 68.370 | 104.125 | 104.125 | 104.125 | 104.125 | 104.125 | 104.125 |
| Minimum | -33.415 | -33.415 | -33.415 | -33.415 | -27.180 | -33.415 | -33.415 | -33.415 | -33.415 | -33.415 | -33.415 | -33.415 | -33.415 | -33.415 |
| Maximum | 104.125 | 104.125 | 104.125 | 104.125 | 104.125 | 104.125 | 104.125 | 104.125 | 104.125 | 104.125 | 104.125 | 104.125 | 104.125 | 104.125 |

BP, bodily pain; GH, general health; MCS, mental component summary; MH, mental health; PCS, physical component summary; PF, physical functioning; RE, role emotional; RP, role physical; VT, vitality; SF, social functioning

**SM4 Table 4**. Canadian norms for VR-12 health utility values, summary component scores, and domain scores, across six self-reported health conditions (kidney disease, liver disease, lung disease, osteoarthritis, rheumatoid arthritis, ulcer or stomach disease), by gender.

|  | Kidney disease | | Liver disease | | Lung disease | | Osteoarthritis | | Rheumatoid  arthritis | | Ulcer or stomach  disease | |
| --- | --- | --- | --- | --- | --- | --- | --- | --- | --- | --- | --- | --- |
|  | Male (n=36) | Female (n=35) | Male (n=34) | Female (n=30) | Male (n=75) | Female (n=71) | Male (n=248) | Female (n=475) | Male (n=124) | Female (n=157) | Male (n=80) | Female (n=107) |
| *VR-12 health utility values* |  |  |  |  |  |  |  |  |  |  |  |  |
| Mean | 0.589 | 0.578 | 0.669 | 0.468 | 0.600 | 0.549 | 0.637 | 0.573 | 0.579 | 0.534 | 0.634 | 0.533 |
| Standard deviation | 0.251 | 0.254 | 0.169 | 0.178 | 0.235 | 0.231 | 0.225 | 0.252 | 0.232 | 0.240 | 0.195 | 0.233 |
| Median | 0.678 | 0.696 | 0.644 | 0.445 | 0.696 | 0.595 | 0.707 | 0.644 | 0.644 | 0.608 | 0.678 | 0.616 |
| 25th percentile | 0.388 | 0.438 | 0.644 | 0.260 | 0.406 | 0.388 | 0.553 | 0.459 | 0.438 | 0.388 | 0.557 | 0.406 |
| 75th percentile | 0.808 | 0.829 | 0.746 | 0.596 | 0.758 | 0.717 | 0.778 | 0.758 | 0.758 | 0.716 | 0.818 | 0.746 |
| Minimum | -0.489 | -0.358 | -0.324 | -0.024 | -0.263 | -0.193 | -0.513 | -0.590 | -0.211 | -0.393 | -0.168 | -0.484 |
| Maximum | 0.913 | 0.919 | 1.000 | 0.913 | 0.952 | 0.952 | 0.952 | 0.960 | 0.952 | 0.913 | 0.913 | 0.912 |
| *VR-12 summary component score* |  |  |  |  |  |  |  |  |  |  |  |  |
| PCS   Mean | 38.427 | 39.667 | 48.632 | 38.841 | 36.483 | 36.904 | 40.558 | 38.948 | 39.885 | 40.892 | 42.026 | 44.869 |
| Standard deviation | 10.709 | 8.422 | 8.982 | 8.825 | 10.735 | 10.490 | 10.910 | 10.375 | 10.340 | 11.224 | 9.695 | 9.619 |
| Median | 41.333 | 42.018 | 48.021 | 40.464 | 36.506 | 37.346 | 41.239 | 40.441 | 42.267 | 41.536 | 43.044 | 48.209 |
| 25th percentile | 27.523 | 32.550 | 44.827 | 28.514 | 27.157 | 27.361 | 33.983 | 31.611 | 30.926 | 33.081 | 39.465 | 38.696 |
| 75th percentile | 48.063 | 46.250 | 56.824 | 49.721 | 45.377 | 46.727 | 49.420 | 46.735 | 48.202 | 49.721 | 49.324 | 53.914 |
| Minimum | 18.755 | 15.591 | 15.287 | 19.202 | 16.295 | 18.292 | 10.494 | 4.409 | 10.492 | 5.715 | 15.290 | 13.883 |
| Maximum | 56.741 | 55.696 | 60.733 | 61.447 | 59.244 | 58.377 | 63.207 | 61.531 | 58.912 | 64.537 | 61.940 | 63.802 |
| MCS  Mean | 51.855 | 47.306 | 48.148 | 43.662 | 51.412 | 48.993 | 52.116 | 49.780 | 50.109 | 47.482 | 48.919 | 44.005 |
| Standard deviation | 8.465 | 8.994 | 10.366 | 8.703 | 9.311 | 7.467 | 8.699 | 9.506 | 8.763 | 8.704 | 8.181 | 8.359 |
| Median | 55.131 | 46.990 | 47.132 | 42.701 | 51.505 | 50.220 | 54.091 | 51.441 | 51.560 | 47.634 | 50.081 | 45.691 |
| 25th percentile | 45.242 | 41.424 | 44.783 | 31.852 | 43.166 | 43.836 | 45.995 | 42.884 | 44.328 | 41.486 | 40.951 | 40.514 |
| 75th percentile | 58.222 | 56.507 | 57.788 | 53.657 | 59.588 | 53.776 | 59.576 | 57.268 | 57.204 | 55.746 | 58.082 | 50.351 |
| Minimum | 18.311 | 17.768 | 26.685 | 20.037 | 20.559 | 30.501 | 21.468 | 17.739 | 22.041 | 19.683 | 28.730 | 18.545 |
| Maximum | 62.385 | 62.516 | 62.711 | 63.670 | 65.118 | 64.353 | 70.517 | 67.832 | 67.596 | 64.581 | 61.565 | 62.871 |
| *VR-12 domain scores* |  |  |  |  |  |  |  |  |  |  |  |  |
| PF     Mean | 51.535 | 54.250 | 70.913 | 49.686 | 46.235 | 42.685 | 60.087 | 52.257 | 55.641 | 54.078 | 66.468 | 66.645 |
| Standard deviation | 27.449 | 20.833 | 19.232 | 22.582 | 27.819 | 28.199 | 26.663 | 26.453 | 25.969 | 28.076 | 22.358 | 22.674 |
| Median | 58.290 | 58.290 | 71.400 | 58.290 | 58.290 | 36.595 | 58.290 | 58.290 | 58.290 | 58.290 | 71.400 | 71.400 |
| 25th percentile | 29.460 | 29.460 | 58.290 | 7.765 | 29.460 | 7.765 | 36.595 | 29.460 | 29.460 | 29.460 | 58.290 | 58.290 |
| 75th percentile | 80.375 | 80.375 | 93.485 | 80.375 | 58.290 | 58.290 | 80.375 | 80.375 | 80.375 | 80.375 | 80.375 | 93.485 |
| Minimum | 7.765 | 7.765 | 7.765 | 7.765 | 7.765 | 7.765 | 7.765 | 7.765 | 7.765 | 7.765 | 7.765 | 7.765 |
| Maximum | 93.485 | 93.485 | 93.485 | 93.485 | 93.485 | 93.485 | 93.485 | 93.485 | 93.485 | 93.485 | 93.485 | 93.485 |
| RP     Mean | 52.991 | 45.796 | 71.182 | 43.169 | 38.941 | 36.294 | 52.034 | 46.455 | 48.629 | 55.101 | 47.374 | 57.626 |
| Standard deviation | 37.003 | 31.679 | 32.371 | 30.051 | 41.363 | 38.310 | 39.374 | 37.540 | 38.007 | 37.392 | 33.762 | 34.608 |
| Median | 79.405 | 44.460 | 79.405 | 9.775 | 9.775 | 9.775 | 79.405 | 44.460 | 44.720 | 79.405 | 39.705 | 79.405 |
| 25th percentile | 5.245 | 5.020 | 45.835 | 0.490 | 5.020 | 5.245 | 9.775 | 9.775 | 5.020 | 9.775 | 9.775 | 9.775 |
| 75th percentile | 89.670 | 79.405 | 100.690 | 90.425 | 79.405 | 79.405 | 89.670 | 79.405 | 89.670 | 89.670 | 79.405 | 100.690 |
| Minimum | -3.670 | -3.670 | -3.670 | -3.670 | -3.670 | -3.670 | -3.670 | -3.670 | -3.670 | -3.670 | -3.670 | -3.670 |
| Maximum | 100.690 | 100.690 | 100.690 | 100.690 | 100.690 | 100.690 | 100.690 | 100.690 | 100.690 | 100.690 | 100.690 | 100.690 |
| BP     Mean | 57.748 | 54.762 | 69.656 | 54.022 | 56.859 | 53.568 | 52.731 | 48.956 | 49.798 | 50.286 | 53.858 | 53.982 |
| Standard deviation | 25.386 | 19.452 | 22.813 | 19.083 | 28.347 | 26.602 | 22.222 | 22.078 | 20.349 | 22.263 | 18.562 | 20.745 |
| Median | 56.250 | 56.250 | 56.250 | 43.890 | 56.250 | 43.890 | 56.250 | 43.890 | 56.250 | 56.250 | 56.250 | 56.250 |
| 25th percentile | 31.500 | 43.890 | 56.250 | 31.500 | 31.500 | 31.500 | 43.890 | 31.500 | 31.500 | 31.500 | 43.890 | 31.500 |
| 75th percentile | 95.200 | 56.250 | 95.200 | 95.200 | 95.200 | 56.250 | 56.250 | 56.250 | 56.250 | 56.250 | 56.250 | 56.250 |
| Minimum | 2.280 | 2.280 | 2.280 | 2.280 | 2.280 | 2.280 | 2.280 | 2.280 | 2.280 | 2.280 | 2.280 | 2.280 |
| Maximum | 95.200 | 95.200 | 95.200 | 95.200 | 95.200 | 95.200 | 95.200 | 95.200 | 95.200 | 95.200 | 95.200 | 95.200 |
| GH    Mean | 52.488 | 54.571 | 70.825 | 48.766 | 50.747 | 55.938 | 63.612 | 60.653 | 61.158 | 59.749 | 54.729 | 63.542 |
| Standard deviation | 18.638 | 20.635 | 20.606 | 13.736 | 21.340 | 18.951 | 23.739 | 22.065 | 22.177 | 22.105 | 25.427 | 20.486 |
| Median | 61.500 | 61.500 | 83.710 | 61.500 | 37.500 | 61.500 | 61.500 | 61.500 | 61.500 | 61.500 | 61.500 | 61.500 |
| 25th percentile | 37.500 | 37.500 | 61.500 | 37.500 | 37.500 | 37.500 | 61.500 | 61.500 | 61.500 | 37.500 | 37.500 | 61.500 |
| 75th percentile | 61.500 | 83.710 | 83.710 | 61.500 | 61.500 | 61.500 | 83.710 | 83.710 | 83.710 | 83.710 | 83.710 | 83.710 |
| Minimum | 0.580 | 0.580 | 0.580 | 0.580 | 0.580 | 0.580 | 0.580 | 0.580 | 0.580 | 0.580 | 0.580 | 0.580 |
| Maximum | 83.710 | 83.710 | 101.840 | 83.710 | 83.710 | 83.710 | 101.840 | 101.840 | 101.840 | 101.840 | 83.710 | 101.840 |
| VT     Mean | 43.624 | 35.356 | 56.584 | 33.823 | 39.305 | 43.972 | 47.097 | 43.046 | 49.630 | 47.718 | 48.332 | 42.145 |
| Standard deviation | 21.250 | 17.730 | 19.012 | 17.369 | 27.470 | 26.118 | 20.628 | 20.617 | 20.803 | 20.533 | 17.193 | 19.617 |
| Median | 45.900 | 31.930 | 54.550 | 31.930 | 45.900 | 45.900 | 45.900 | 45.900 | 45.900 | 45.900 | 45.900 | 45.900 |
| 25th percentile | 31.930 | 31.930 | 45.900 | 1.800 | 31.930 | 31.930 | 31.930 | 31.930 | 31.930 | 31.930 | 31.930 | 31.930 |
| 75th percentile | 54.550 | 54.550 | 54.550 | 45.900 | 54.550 | 54.550 | 54.550 | 54.550 | 54.550 | 54.550 | 54.550 | 54.550 |
| Minimum | 1.800 | 1.800 | 1.800 | 1.800 | 1.800 | 1.800 | 1.800 | 1.800 | 1.800 | 1.800 | 1.800 | 1.800 |
| Maximum | 79.250 | 79.250 | 95.340 | 79.250 | 95.340 | 95.340 | 95.340 | 95.340 | 95.340 | 95.340 | 79.250 | 79.250 |
| SF      Mean | 66.992 | 65.036 | 64.188 | 51.790 | 67.278 | 61.721 | 72.738 | 69.632 | 65.279 | 61.159 | 67.135 | 56.134 |
| Standard deviation | 25.621 | 27.425 | 25.564 | 20.409 | 25.445 | 25.017 | 26.302 | 27.336 | 24.269 | 26.039 | 24.237 | 24.425 |
| Median | 66.870 | 66.870 | 66.870 | 48.870 | 66.870 | 66.870 | 66.870 | 66.870 | 66.870 | 66.870 | 66.870 | 48.870 |
| 25th percentile | 48.870 | 48.870 | 48.870 | 24.910 | 48.870 | 48.870 | 48.870 | 48.870 | 48.870 | 48.870 | 48.870 | 24.910 |
| 75th percentile | 99.320 | 99.320 | 99.320 | 66.870 | 99.320 | 66.870 | 99.320 | 99.320 | 99.320 | 99.320 | 99.320 | 66.870 |
| Minimum | 2.780 | 2.780 | 2.780 | 2.780 | 2.780 | 2.780 | 2.780 | 2.780 | 2.780 | 2.780 | 2.780 | 2.780 |
| Maximum | 99.320 | 99.320 | 99.320 | 99.320 | 99.320 | 99.320 | 99.320 | 99.320 | 99.320 | 99.320 | 99.320 | 99.320 |
| MH   Mean | 73.841 | 66.718 | 67.839 | 58.269 | 73.394 | 65.740 | 73.282 | 66.946 | 68.964 | 64.146 | 69.419 | 57.231 |
| Standard deviation | 19.781 | 18.288 | 21.162 | 19.989 | 20.138 | 18.814 | 19.091 | 21.625 | 20.283 | 19.952 | 19.342 | 18.508 |
| Median | 83.670 | 74.485 | 60.685 | 55.620 | 74.865 | 69.490 | 74.865 | 74.485 | 74.865 | 65.505 | 74.865 | 60.685 |
| 25th percentile | 60.685 | 51.325 | 60.685 | 40.305 | 65.505 | 51.045 | 59.850 | 51.325 | 51.325 | 49.545 | 51.325 | 41.685 |
| 75th percentile | 92.650 | 83.845 | 83.670 | 92.650 | 92.650 | 83.670 | 92.650 | 83.670 | 83.845 | 83.670 | 83.845 | 74.485 |
| Minimum | -1.030 | -1.030 | 26.385 | 6.005 | -1.030 | 6.005 | 6.005 | -1.030 | 6.005 | 6.005 | 21.060 | 6.005 |
| Maximum | 92.650 | 98.595 | 98.595 | 98.595 | 98.595 | 98.595 | 98.595 | 98.595 | 98.595 | 98.595 | 92.650 | 98.595 |
| RE     Mean | 71.260 | 56.422 | 60.888 | 46.207 | 64.727 | 61.541 | 75.201 | 64.373 | 65.061 | 57.837 | 60.271 | 56.949 |
| Standard deviation | 32.375 | 36.317 | 32.455 | 28.368 | 40.396 | 32.472 | 36.568 | 36.578 | 36.251 | 36.393 | 27.976 | 33.833 |
| Median | 77.005 | 54.690 | 42.775 | 42.775 | 77.005 | 54.690 | 104.125 | 68.370 | 81.810 | 54.690 | 68.370 | 54.690 |
| 25th percentile | 54.690 | 29.335 | 42.775 | 13.250 | 29.335 | 35.050 | 42.775 | 41.250 | 41.250 | 29.335 | 29.335 | 29.335 |
| 75th percentile | 104.125 | 104.125 | 104.125 | 81.810 | 104.125 | 104.125 | 104.125 | 104.125 | 104.125 | 104.125 | 81.810 | 104.125 |
| Minimum | -33.415 | -33.415 | -33.415 | -33.415 | -33.415 | -33.415 | -33.415 | -33.415 | -33.415 | -33.415 | -27.180 | -33.415 |
| Maximum | 104.125 | 104.125 | 104.125 | 104.125 | 104.125 | 104.125 | 104.125 | 104.125 | 104.125 | 104.125 | 104.125 | 104.125 |

BP, bodily pain; GH, general health; MCS, mental component summary; MH, mental health; PCS, physical component summary; PF, physical functioning; RE, role emotional; RP, role physical; VT, vitality; SF, social functioning
